# High performance p-type molecular electron donors for OPV applications via alkylthiophene catenation chromophore extension

**DOI:** 10.3762/bjoc.12.223

**Published:** 2016-11-02

**Authors:** Paul B Geraghty, Calvin Lee, Jegadesan Subbiah, Wallace W H Wong, James L Banal, Mohammed A Jameel, Trevor A Smith, David J Jones

**Affiliations:** 1 School of Chemistry, Bio21 Institute, University of Melbourne, Parkville Vic 3010, Melbourne, Australia; 2 School of Chemistry, University of Melbourne, Parkville Vic 3010, Melbourne, Australia

**Keywords:** molecular materials, nematic liquid crystal, organic solar cells, organic synthesis, p-type organic semiconductors, small molecule

## Abstract

The synthesis of key 4-alkyl-substituted 5-(trimethylsilyl)thiophene-2-boronic acid pinacol esters **3** allowed a simplified alkylthiophene catenation process to access bis-, ter-, quater-, and quinquethiophene π-bridges for the synthesis of acceptor–π-bridge-donor– π-bridge-acceptor (A–π-D–π-A) electron donor molecules. Based on the known benzodithiophene-terthiophene-rhodanine (**BTR**) material, the **BXR** series of materials, **BMR** (X = M, monothiophene), **BBR** (X = B, bithiophene), known **BTR** (X = T, terthiophene), **BQR** (X = Q, quaterthiophene), and **BPR** (X = P(penta), quinquethiophene) were synthesised to examine the influence of chromophore extension on the device performance and stability for OPV applications. The **BT^x^R** (x = 4, butyl, and x = 8, octyl) series of materials were synthesised by varying the oligothiophene π-bridge alkyl substituent to examine structure–property relationships in OPV device performance. The devices assembled using electron donors with an extended chromophore (**BQR** and **BPR**) are shown to be more thermally stable than the **BTR** containing devices, with un-optimized efficiencies up to 9.0% PCE. **BQR** has been incorporated as a secondary donor in ternary blend devices with **PTB7-Th** resulting in high-performance OPV devices with up to 10.7% PCE.

## Introduction

Bulk heterojunction (BHJ) organic solar cells (OSC), a blend of p-type and n-type conjugated polymers or molecular materials (MM), have attracted significant attention as alternative solar cell technologies as they are light-weight, low-cost and offer the opportunity of cheaper manufacturing employing roll-to-roll printing processes [1–3]. Recent advances in materials synthesis and device architecture has pushed OSC power conversion efficiencies (PCEs) to 11.5% [4–5]. Further materials design and device optimizations have been proposed to deliver OSCs with PCEs up to 15% [6–7]. Although the field has been dominated by polymeric conjugated organic semiconductors, there has been a rapid advance in the development of MMs with PCEs over 10% now reported [8–9]. The switch to MMs has in part been due to their discrete structure and relative ease of purification, which offers significant advantages, especially reduced batch-to-batch variation [10–12].

We recently used side-chain engineering, through regioregular placement of hexyl side chains on a thiophene π-bridge [13], to generate a MM with a planar core structure and enhanced device performance, up to 9.3% power conversion efficiency (PCE) [14]. This material, built from three key building blocks benzodithiophene-terthiophene-rhodanine (**BTR**), has been shown to have intriguing materials behaviour and excellent device performance when combined with [6,6]-phenyl C_71_ butyric acid methyl ester (**PC_71_BM**). Maximum PCEs of 9.3% for OSCs containing **BTR** are achieved after solvent vapor annealing, for devices with an active layer up to 310 nm thick. In this case fill-factors (FF) remain above 70%. However, OSC devices containing **BTR** are not stable to thermal annealing, a requirement for scale up using common printing processes, where temperatures >80 °C are required for drying or annealing of printed layers [15]. **BTR** has extremely interesting properties worth further study and leads to three key questions;

1. Synthesis: Can we simplify the synthesis of **BTR** removing some chromatographic purification steps and use of toxic tin containing Stille condensation reactions?
2. Scale-up: Can we develop a multi-gram synthesis route to facilitate translation to printing programs?
3. Structure–property relationships: Can we modify the **BTR** chromophore length or alkyl side-chain length thereby improving device thermal stability and device performance?

**Figure 1:**
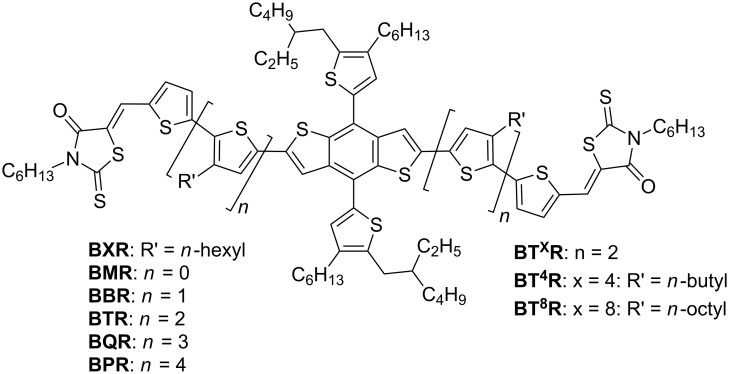
Chemical structures of molecular materials with the following variations; **BT^x^R**, alkyl side chains of the terthiophene bridging arm and **BXR**, oligothiophene bridging arm.

We report here a simplified synthetic route to a series of **BTR** analogues (Figure 1), where we have varied the chromophore length through the **BXR** series, where X = monothiophene (M), bithiophene (B), the known terthiophene (T), quaterthiophene (Q), and quinquethiophene (P), respectively and allowing isolation of products on the multigram scale. The simplified synthesis was translated to a second series of products where the oligothiophene sidechain length for the parent (**BTR**) was systematically varied, i.e. **BT^x^R**, where x = 4 (butyl), or 8 (octyl). Incorporation of the **BXR** series in devices with **PC_71_BM** has demonstrated that with increasing chromophore length, the thermal stability of the OSC devices increases giving a PCE of 8.9% for **BQR** after thermal annealing at 120 °C for 10 minutes. We also report an initial result of PCE of 10.7% for ternary blends of **BQR** with the commercially available **PTB7-Th** as the donor and **PC_71_BM** as the acceptor.

## Results and Discussion

**Scheme 1:**
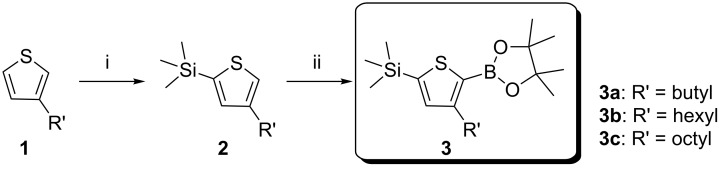
Synthesis of the key intermediates TMS-T_x_-BPin (3), i) diisopropylamine (DIA), THF, *n*-BuLi, −78 °C TMS-Cl, ii) *n*-BuLi, iPrOBPin, THF, −78 °C.

**Synthesis**: Our modified synthesis of **BTR** and its analogues starts with the lithiation of 3-alkylthiophene **1a–c** by lithium diisopropylamide formed in situ from the reaction of *n*-butyllithium with diisopropylamine (DIA) in the presence of the alkylthiophene, followed by quenching with trimethylsilyl chloride to generate the previously unreported 4-alkyl-2-(trimethylsilyl)thiophenes **2a–c**, which could be purified by distillation to ensure removal of unreacted 3-alkylthiophene, Scheme 1. Deprotonation of **2** with *n*-butyllithium and reaction with 2-isopropoxy-4,4,5,5-tetramethyl-1,3,2-dioxaborolane (iPrOBPin) resulted in formation of the key intermediates **3a–c**, after distillation, in high yield of 60–70% (see refs [16–18] for recent similar chemistry). Intermediate **3b** has been scaled to the mole scale with no issues noted.

**Scheme 2:**
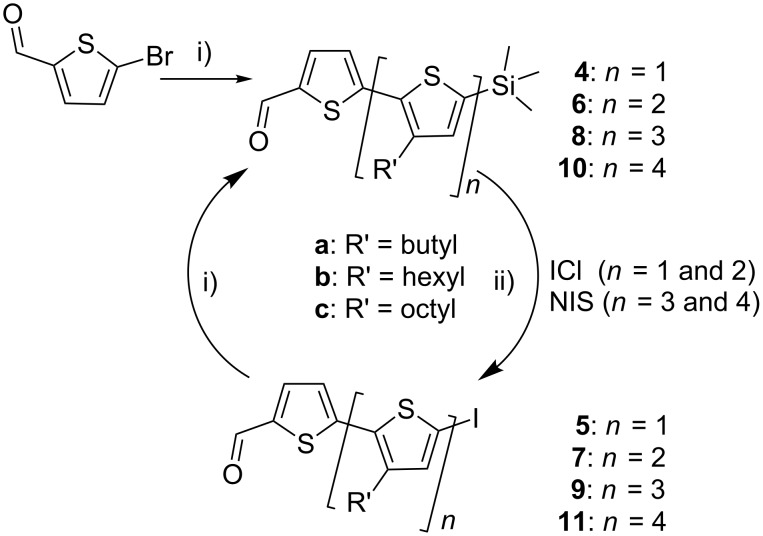
Oligothiophenes **4**–**11** synthesised through reaction of the commercially available 5-bromo-2-thiophenecarboxaldehye with **3a–c**, i) 3, cat. Pd_2_dba_3_.4[*t*-Bu_3_PH]BF_4_, THF, K_3_PO_4_ (2 M), 80 °C, 16 h, ii) ICl, DCM, 0 °C or NIS, 50:50 CHCl_3_/CH_3_CO_2_H, rt, 2 h.

With **3a–c** in hand, synthesis of the required series of alkyl substituted oligothiophene π-bridges by simple Suzuki–Miyaura cross-coupling reactions could be completed. Starting with commercially available 5-bromothiophene-2-carboxaldehyde and then reaction with **3a–c** to generate the required bithiophenes, then terthiophenes, while further catenation with **3b** resulted in synthesis of the hexyl-substituted quater- and quinquethiophenes **8b** and **10b**, Scheme 2. Conversion of the intermediate TMS-protected oligothiophenes to the iodo-oligothiophenes (*n* =1 and 2) was achieved using iodine monochloride (ICl), however for *n* = 3 and 4 a number of side reactions leading to unidentified side products significantly reduced the yield. For the quater- and quinquethiophenes *N*-iodosuccinimide (NIS) was used to give a clean product.

**Scheme 3:**
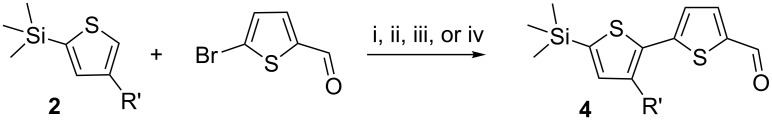
Synthesis of the bithiophene through palladium catalyzed direct arylation, a) i) Pd(OAc)_2_, PCy_3_, PivOH, K_2_CO_3_, toluene 100 °C, 4 h, 1%, ii) Pd(OAc)_2_, PPh_3_, K_2_CO_3_, DMF 120 °C, 6 h, 10%, iii) Pd(OAc)_2_, dppp, KOAc, DMAc 120 °C, 5 h, 32%, iv) Pd(OAc)_2_, dppb, KOAc, DMAc 120 °C, 5 h, 32%.

Ideally, the synthesis could be further simplified by direct palladium-catalyzed CH-activation, arylation of **2** followed by reaction with the commercially available 5-bromothiophene-2-carboxaldehyde to generate the bithiophene **4**, Scheme 3. It has previously been reported that direct coupling of 2-(trimethylsilyl)thiophene with aryl halides proceeds in good yield with protodesilylation being the major side reaction under the reaction conditions, even at short reaction times [19]. An initial reaction screening, investigating ligand, base and solvent variation, showed positive results with up to 32% yield of the required bithiophene **4**. We are currently examining catalyst optimization to improve the yields of this simplified route to the required oligothiophenes.

**Scheme 4:**
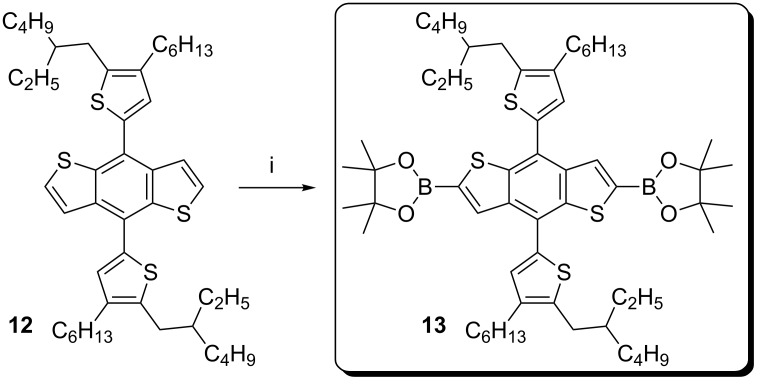
Synthesis of the key bis-borylated BDT core **13**, i) 1.5 equiv B_2_Pin_2_, 0.025 equiv [Ir(COD)OMe]_2_, 0.05 equiv *t*-Bu_2_bipy, DME, 80 °C, 2 h.

To avoid large scale use of tin reagents we required the key bis-borylated benzodithiophene (BDT) core **13**, which was synthesised from the known BDT core **12** using iridium catalyzed borylation via CH-activation. The bis-borylated product was isolated by precipitation on addition of isopropanol (IPA), and an analytically pure material isolated by filtration in excellent yields >90%, Scheme 4. This simplified purification is in direct contrast with reported procedures for the bis-iodinated or bis-stannylated analogues [20–21].

**Scheme 5:**
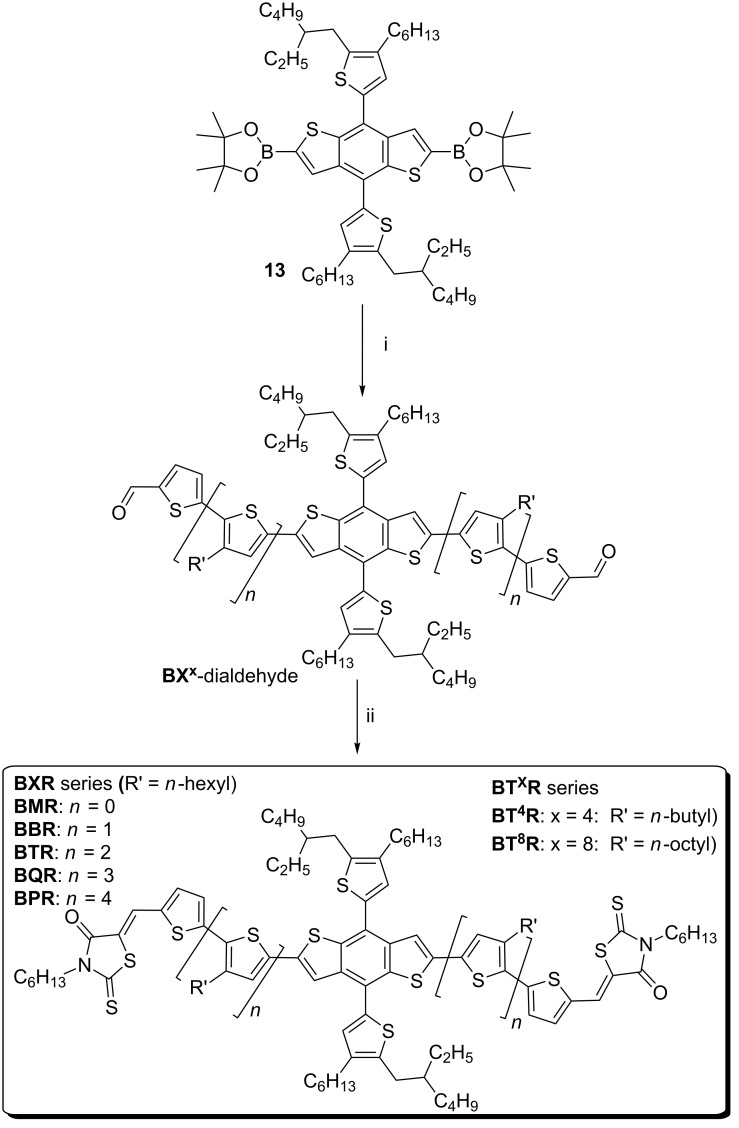
Synthesis of the **BXR** and **BT^X^R** series of materials, i) 5-bromothiophene carboxaldehyde, **5b**, **7a–c**, **9b**, or **11b**, cat. Pd_2_dba_3_·4*t-*Bu_3_P[HBF_4_], THF, K_3_PO_4_ (2M), 80 °C, 16 h, and ii) *N*-hexylrhodanine (10 equiv), CHCl_3_, cat DBU.

A simple Suzuki–Miyaura cross coupling of **5**, **7**, **9** or **11** with **13** gave the required **BX^x^**-dialdehydes **14**, Scheme 5, which were purified by a combination of silica chromatography and size exclusion chromatography (SEC). A final Knoevenagel condensation coupling the **BX^x^**-dialdehydes with *N*-hexyl-rhodanine resulted in the required series of products, both **BXR** and **BT^x^R**. The new materials have been fully characterized by NMR, IR, UV–vis, TGA, DSC, electrochemistry, photoelectron spectroscopy in air (PESA), and have satisfactory mass spectra and microanalysis results. Full experimental details are described in Supporting Information File 1.

**Figure 2:**
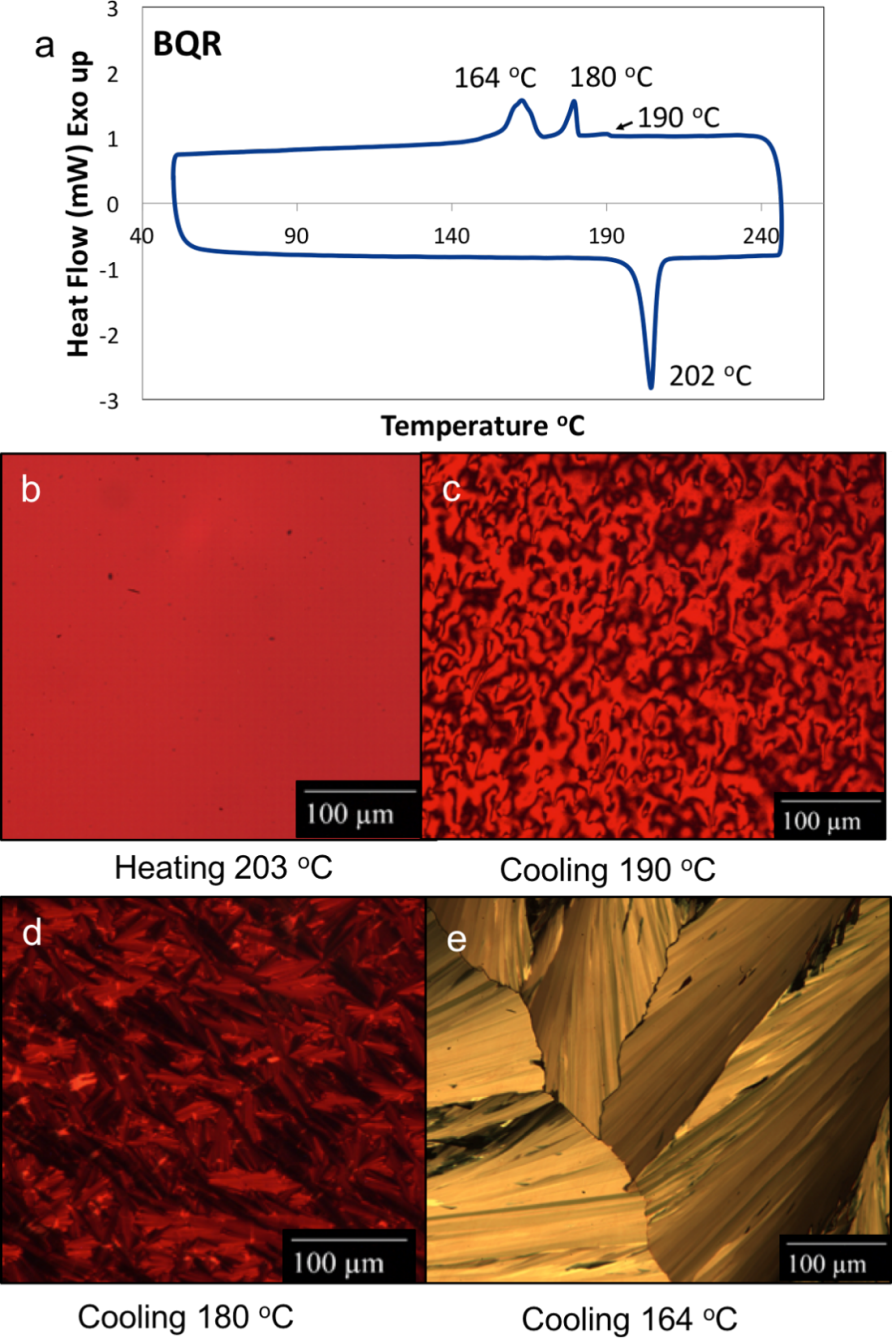
**BQR** thermal and POM properties. a) DSC thermogram of **BQR** under nitrogen at a ramp rate of 10 °C min^–1^. b) **BQR** thin film sandwiched between two glass slides observed under a polarized optical microscope (POM) at 203 °C after heating. c) POM image of the same film when the stage temperature is lowered to 190 °C with distinct Schlieren texture for a NLC, d) POM image when the stage temperature is lowered to 180 °C, and e) POM image when the stage temperature is lowered to 164 °C where a distinct transmitted colour change is observed.

**Thermal behaviour: TGA and DSC**. The thermal behaviour of the **BXR** and **BT^x^R** series has been studied by TGA and DSC. All of the materials show good thermal stability with <5% weight loss below 390 °C, see Supporting Information File 1, Figure S5.1. The phase behaviour was examined by DSC (see Supporting Information File 1, Figure S6.1 for full details) with a single melt temperature recorded for **BMR**, **BBR** and **BPR** at 179 °C, 215 °C and 196 °C, respectively. Subsequent crystallization was observed at 154 °C, 175 °C and 174 °C for **BMR**, **BBR** and **BPR**, respectively. The phase behaviour for **BTR** has been previously reported and shows three phase transitions on heating and cooling with the formation of a high-temperature nematic liquid crystalline (NLC) phase change at 186 °C, with a change to the isotropic phase at 196 °C [14]. **BQR** can then be compared to **BTR** where, surprisingly, a single phase change is seen on heating, while three phase changes are observed on cooling, Figure 2a. Even on slowing the heating rate to 0.1 °C per minute no change in the single phase change on heating was observed. The three phase changes at 190 °C, 180 °C, and 164 °C on cooling appear to be analogous to that seen in **BTR**.

Modification of the **BTR** oligothiophene alkyl chain lengths in the **BT^x^R** series results in an intuitive change in the temperatures of the relevant phase transitions, with an inverse correlation observed between alkyl chain length and the specific phase change temperatures. Interestingly, as with **BQR** the DSC traces of the **BT^x^R** analogues reveal markedly different phase behaviour relative to that of **BTR**. **BT^4^R** has a single endothermic (206 °C) and a single exothermic peak (199 °C) that are higher than the phase transitions in **BTR**. Two exothermic peaks at 148 °C and 182 °C are observed in **BT^8^R**, and two endothermic peaks are recorded at 100 °C and 166 °C. As can be observed, even these small changes in alkyl chain length result in a significant impact on the phase change behaviour. However, unfortunately no correlation can be made at this stage between subsequent thermal stability of OPV devices and the phase transition of the **BXR** and **BT^x^R** materials.

**Polarized optical microscopy (POM)**. POM was utilized in conjunction with a heating platform to directly observe these phase transitions and elucidate thin film structure. On heating at 10 °C·min^−1^ **BQR** shows a single phase transition to the isotropic melt at 202 °C, while on cooling we have identified an initial transition to a high-temperature NLC phase at 190 °C, and then a crystallisation at 180 °C. On further cooling a thermochromic phase change is observed at 164 °C, see Figure 2 (and UV–vis discussion below). Even with the much slower cooling rates used for POM studies we did not observe more than the single phase change on heating the **BQR** sample. We have repeated POM studies on the new batches of **BTR** and they are identical to those reported; see Supporting Information File 1, Figure S8.2 [14].

**Figure 3:**
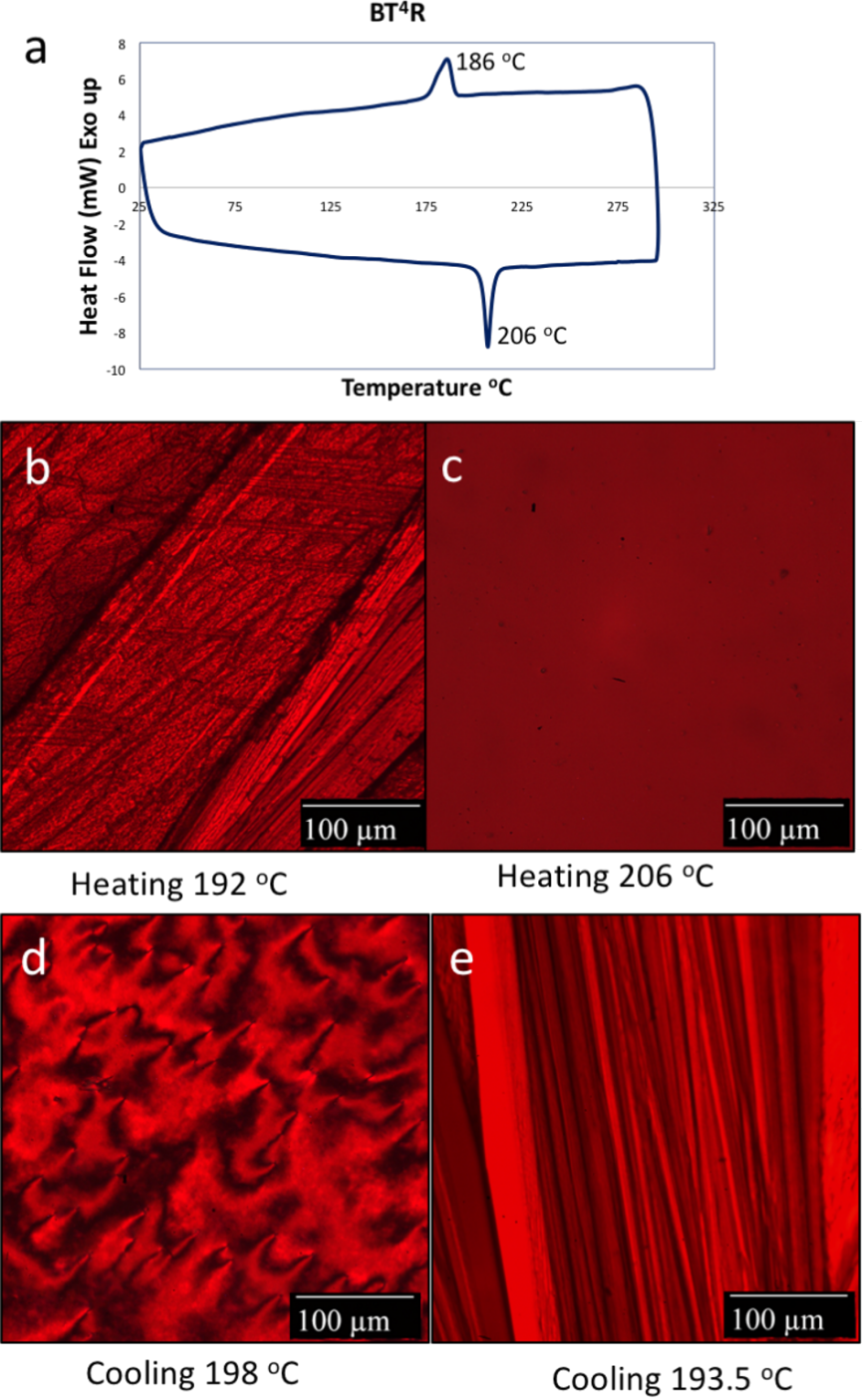
**BT^4^R** thermal and POM images. a) DSC thermogram of **BT^4^R** under nitrogen at a ramp rate of 10 °C min^−1^. b) **BT^4^R** thin film sandwiched between two glass slides observed under a POM at 192 °C on heating. c) POM image of the same film when the stage temperature is at 206 °C, d) POM image when the stage temperature is lowered to 198 °C with distinct Schlieren texture for a NLC, and e) POM image when the stage temperature is lowered to 193.5 °C.

Upon examination of **BT^4^R** with POM a single phase change on heating was observed, with a highly crystalline state below 205 °C giving way to an isotropic melt at 206 °C. Surprisingly, when the sample was cooled a characteristic NLC was observed at 199 °C, with a change to its crystalline state at 193 °C, Figure 3. This NLC transition was not observed in the DSC, even with a slowed cooling rate.

The thermal transitions in **BT^8^R** are not as defined as in the other cases with two broad endothermic (148 °C and 182 °C) and exothermic transitions (166 °C and 100 °C) (Supporting Information File 1, Figure S8.5). No high temperature NLC phase was observed when examined under POM.

The dramatic changes in the thermal behaviour and phase change properties for the **BX^x^R** series materials are induced by either changes to the chromophore length, or by altering the side chain length on the oligothiophene. These changes have a dramatic impact on the presence or absence of a NLC phase in these materials. All, however, show the appearance of long needle like crystal forms in the POM images obtained.

**Figure 4:**
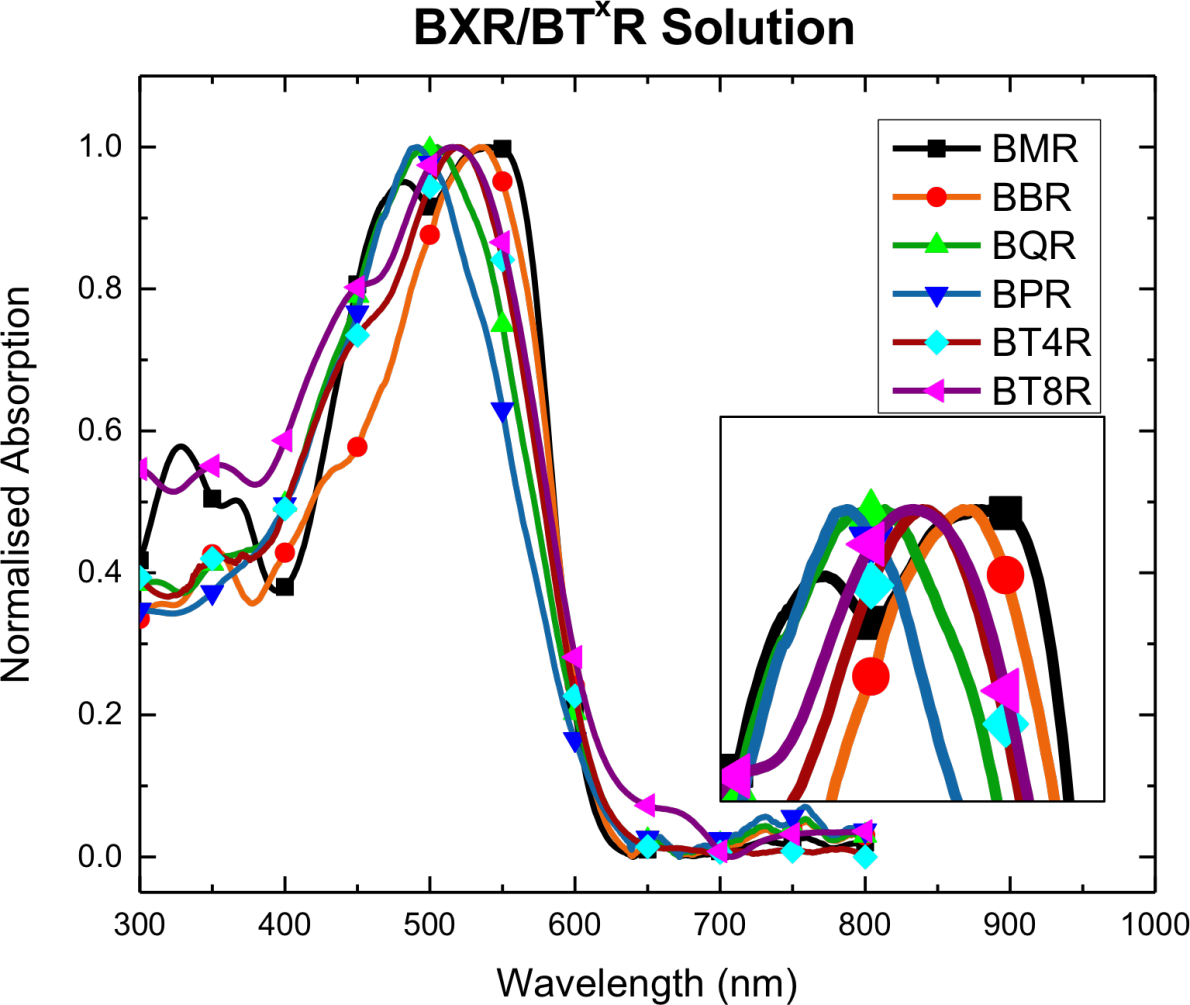
UV–vis absorption spectra of the **BX^X^R** series in CHCl_3_. An expansion of the peak area is shown in the inset.

**Figure 5:**
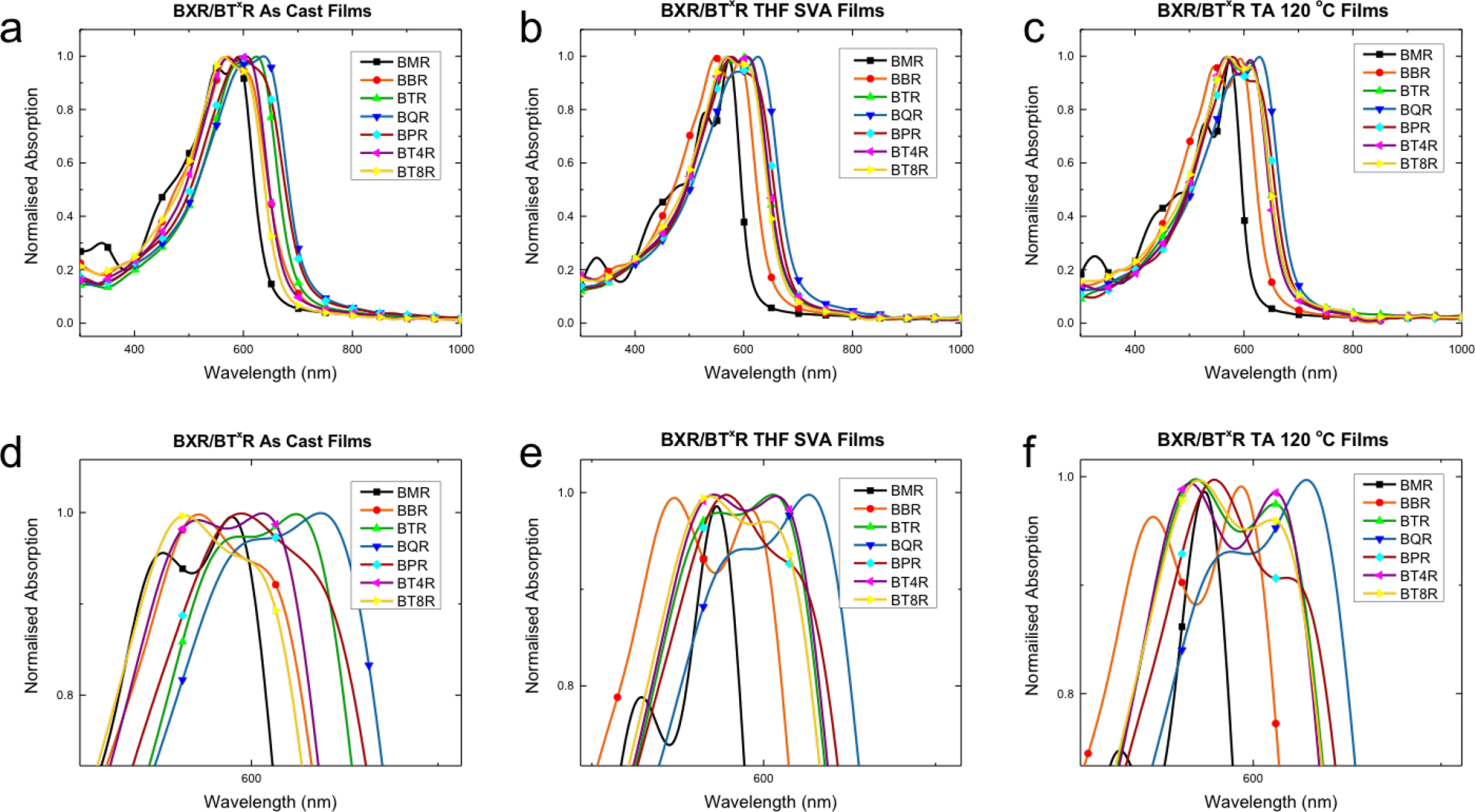
Normalised thin film UV–vis absorption profiles for the **BX^x^R** series for, a) as-cast films (from CDCl_3_), b) after solvent vapor annealing (SVA with THF for 10 seconds, and c) thermally annealed (TA) at 120 °C for 10 minutes. Expansions of the peak areas in a), b), and c) are shown in d), e), and f).

**UV–vis and fluorescence spectroscopy**. Solution and thin film UV–vis absorption profiles of the **BX^x^R** are shown in Figure 4 and Figure 5, respectively, with selected data collected in Table 1 (all spectra can be found in the Supporting Information File 1). The members of the series all exhibit absorption maxima between 450–600 nm in chloroform. In solution it is evident that, although all the materials have a similar onset of absorption at around 600 nm, the peak maxima progress in the reverse order to that expected with **BPR** having a maximum absorption at 490 nm, while **BMR** has a maximum absorbance at 541 nm, Table 1. While the **BPR** and **BQR** spectra show broad featureless peaks, the spectra for **BMR** (and **BBR**) show more complicated structure indicating possible association in solution with the development of strong aggregates.

**Table 1:** Extracted UV–vis absorption peak positions for the **BX^x^R** series. (λ_max_ in bold).

	Solution		ε	As cast		SVA		TA 120 °C		TA 200 °C		TA 220 °C

	λ (nm)		(M^-1^cm^-1^)	λ (nm)		λ (nm)		λ (nm)		λ (nm)		λ (nm)

**BMR**	480	**541**	95,500	552	**590**	528	**572**	529	**574**			
**BBR**		**535**	62,500	**571**	602	**548**	593	545	**594**			
**BTR**		**523**	110,000^a^	**592**	624	573	**605**	**569**	610			
**BQR**		**500**	92,500	600	**634**	587	**626**	588	**628**	584	**634**	**690**
**BPR**		**490**	104,000	**594**	630	**578**	612	**579**	618			
**BT^4^R**		**520**	88,500	571	**605**	**570**	607	**566**	612			
**BT^8^R**		**516**	69,100	**564**	595	**568**	602	**570**	610			

^a^Taken from ref. [14].

The UV–vis spectra for thin films of the **BX^x^R** series, cast from chloroform and subject to both solvent vapour annealing (SVA, THF 20 s) or thermal annealing (TA, 120 °C 10 min, N_2_), are shown in Figure 5. On increasing the conjugation length from **BMR** to **BPR** the expected red-shift in the absorption peaks is now evident (Figure 5a). However, λ_max_ is dependent on the degree of formation of π–π stacking and development of the lowest energy transition with two clear sharp peaks at 552 nm and 590 nm (λ_max_) for **BMR**, while for **BPR** λ_max_ is at 594 nm with a shoulder at around 630 nm indicating poor formation of the aggregates (Figure 5d). Side-chain modification from butyl to octyl in the **BT^4^R**, **BTR** and **BT^8^R** series also impacts on the thin film formation as seen in as-cast films with poor development of the crystalline order in **BT^4^R** and **BT^8^R**, as well as a λ_max_ blue-shift of both **BT^X^R** analogues, when compared to **BTR**, Figure 5a and d.

Crystalline order develops for all films after SVA or TA, (Figure 5b and c). While after annealing λ_max_ corresponds to the lowest energy band for most of the thin films, or the two peaks are close in intensity, **BPR** is the exception where the long wavelength absorption is a shoulder that is not well resolved and λ_max_ corresponds to the higher energy band. In both cases **BQR** shows well-ordered films with the largest red shift and a λ_max_ at 625–630 nm. After annealing the absorption profiles of **BT^4^R**, **BTR** and **BT^8^R** are almost identical with only a small change in the intensity of the peak at around 570 nm, therefore indicating that the underlying packing structures are not significantly altered through side-chain substitution.

**Figure 6:**
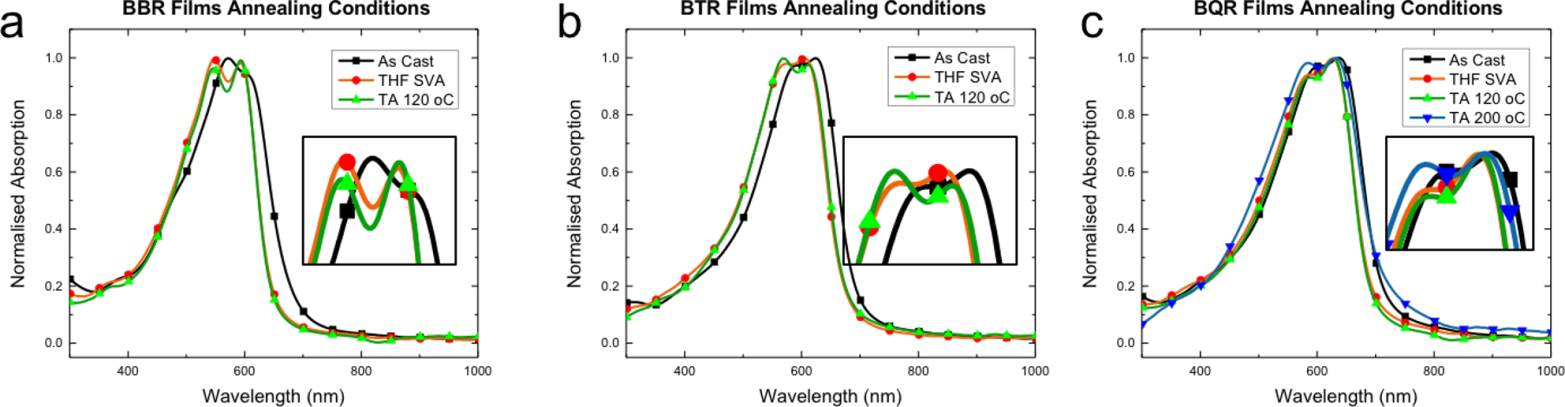
Normalised thin film UV–vis absorption profiles for a) **BBR**, b) **BTR** and c) **BQR** showing as-cast (black, square), SVA with THF for 10 s (orange, circle) and thermally annealed (TA) at 120 °C for 10 minutes (green, triangle). Insets show expansion of the main peak area.

To better understand the annealing process UV–vis spectra have been replotted for each material, see Figure 6 (and Supporting Information File 1, Figures S9.1 and 2). As each material is annealed a small blue shift, 10–20 nm, is seen in most spectra with a concomitant increase in the prominence of the low energy peak. The shift is smallest in **BPR** (Figure 6c), and no significant change is seen for **BT^4^R** (Supporting Information File 1, Figure S9.2f). It is evident that subtle changes in molecular orientation and packing, with a tendency to H-aggregate formation, are present, however, further work is being undertaken to better understand the underlying processes leading to these changes.

**Figure 7:**
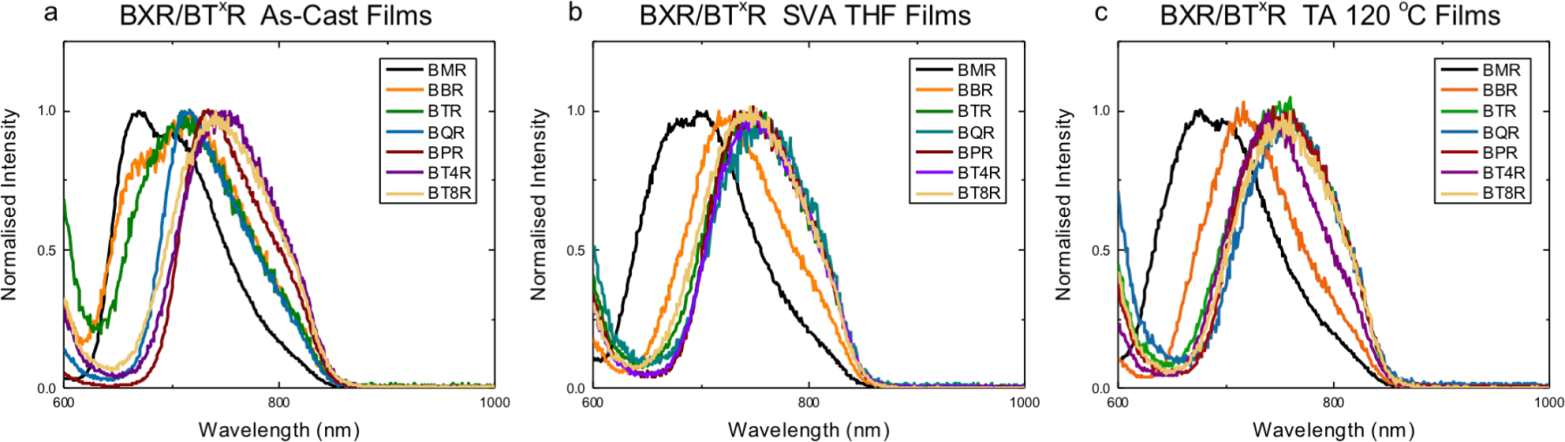
Normalised thin film fluorescence emission profiles for **BX^x^R** series after excitation at 580 nm, a) as-cast films (from CDCl_3_), b) after solvent vapor annealing (SVA with THF for 10 seconds), and c) thermally annealed (TA) at 120 °C for 10 minutes.

Fluorescence emission spectra were collected using the same films as those used to collect the UV–vis spectra above, with selected graphs shown in Figure 7, and extracted data in Table 2. The full spectra are presented in Supporting Information File 1, Figure S9.3. The as-cast films do not contain simple symmetric emission bands, indicating a significant level of structural complexity in the as-cast thin films. **BMR** shows two emission peaks at 670 nm and 704 nm with a long tail at around 800 nm. A number of absorption peaks are evident for **BMR**, Figure 5a and d, and these may represent the multiple environments for emission. **BBR**, **BTR** and **BQR** show surprisingly asymmetric peaks with long linear tails from a peak maximum located at around 715 nm. Following SVA a broad, more symmetric emission band is seen for all materials, except **BMR** and **BBR**, located at around 750 nm with a broad shoulder indicating a secondary emission located at around 810–825 nm. For TA films, there is little change in the emission from that seen for SVA films indicating that under these conditions a similar underlying structure is formed after solvent vapour or thermal annealing.

**Table 2:** Extracted fluorescence emission peak positions for the **BX^x^R** materials (λ_excit_ 580 nm).

	As cast		SVA	TA 120 °C	TA 200 °C	TA 220 °C (fast cool)	TA 220 °C (slow cool)

	λ (nm)		λ (nm)	λ (nm)	λ (nm)	λ (nm)	λ (nm)

**BMR**	669	697	705	677			
**BBR**	678	718	716	719			
**BTR**		712	752	735			
**BQR**		716	756	763	760	730	709
**BPR**		732	736	762			
**BT^4^R**		746	749	739			
**BT^8^R**		741	740	759			

**Figure 8:**
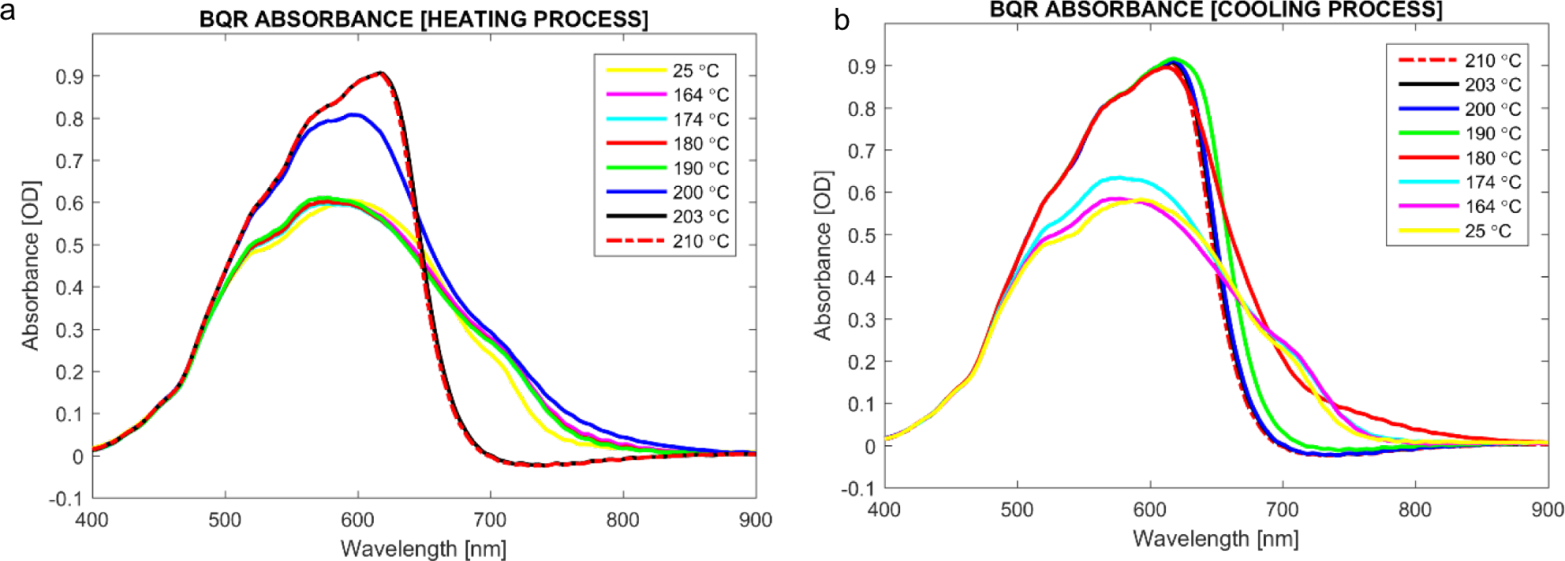
Variable temperature thin film UV–vis absorption profiles for **BQR**, collected using the transmission lamp of the POM and fibre-optic spectrometer.

**Variable temperature UV–vis and fluorescence measurements**. POM measurements of **BQR** indicated a change in the transmitted spectrum during the phase change recorded at 164 °C, Figure 2, suggestive of significant structural rearrangements occurring during crystallization. The POM/heating stage apparatus was coupled to a fibre-optic based spectrometer to enable collection of variable temperature UV–vis and fluorescence spectral data from **BTR** and **BQR**. The absorption spectra recorded for **BQR** are shown in Figure 8, while the those for **BTR** are included in the supplementary material (Figure S8.1). The absorption spectrum of **BQR** collected on the POM heated stage shows and extra shoulder located at around 730 nm that was not present in the as-cast films of **BQR** or the thermally annealed films (measured at room temperature). The **BQR** thin films were annealed up to 220 °C to probe the effect on the UV–vis spectrum (spectrum collected at room temperature after cooling) of cycling the **BQR** thin film up to the NLC phase change temperatures (Figure 10) but there is no appearance of the new shoulder, however as the films are rapidly cooled there may be a kinetic effect (see below).

**Figure 9:**
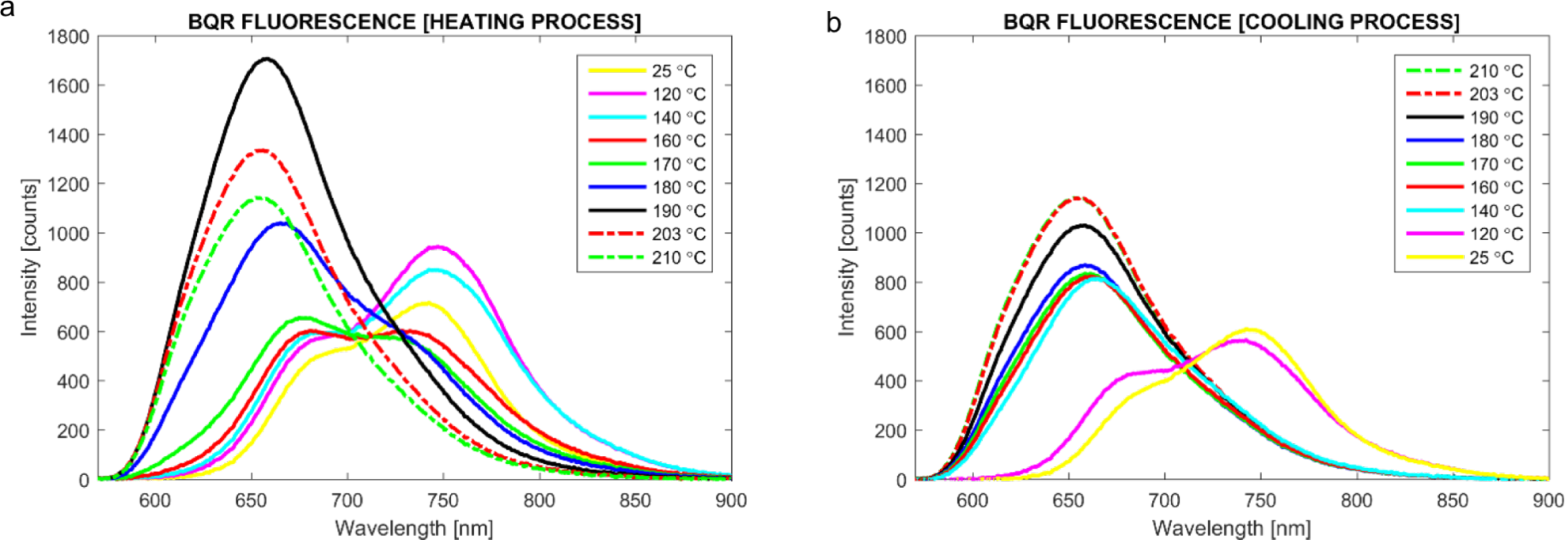
Variable temperature thin-film fluorescence emission profiles for **BQR**, a) heating and b) cooling, collected using the same apparatus as Figure 8 but with an Hg lamp as the excitation source.

Variable temperature fluorescence emission spectra were recorded on **BTR** and **BQR** (Figure 9) using a similar setup as for the UV–vis measurements (Figure 8), but employing the Hg fluorescence excitation lamp of the microscope. However, the lamp used introduced a significant thermal load on the sample (approx. 18 °C) and therefore the apparent phase change temperatures are offset relative to the absorption data for this experiment. The data for **BQR** are shown below, while the data for **BTR** are included in Supporting Information File 1. At room temperature (after cycling once) **BQR** has two emission peaks at around 750 nm and 690 nm. On heating the low energy peak reduces in intensity with a concomitant increase in the peak at 690 nm and a blue shift to 655 nm at the sample melting point to the isotropic phase (Figure 9a). A similar shift is observed on cooling the sample, Figure 9b. The emission peaks appear to reflect the two absorption peaks observed in the variable temperature UV–vis absorption spectra, however the underlying structural changes remain unclear and are the subject of further structural studies on **BTR** and **BQR** thin films.

**Figure 10:**
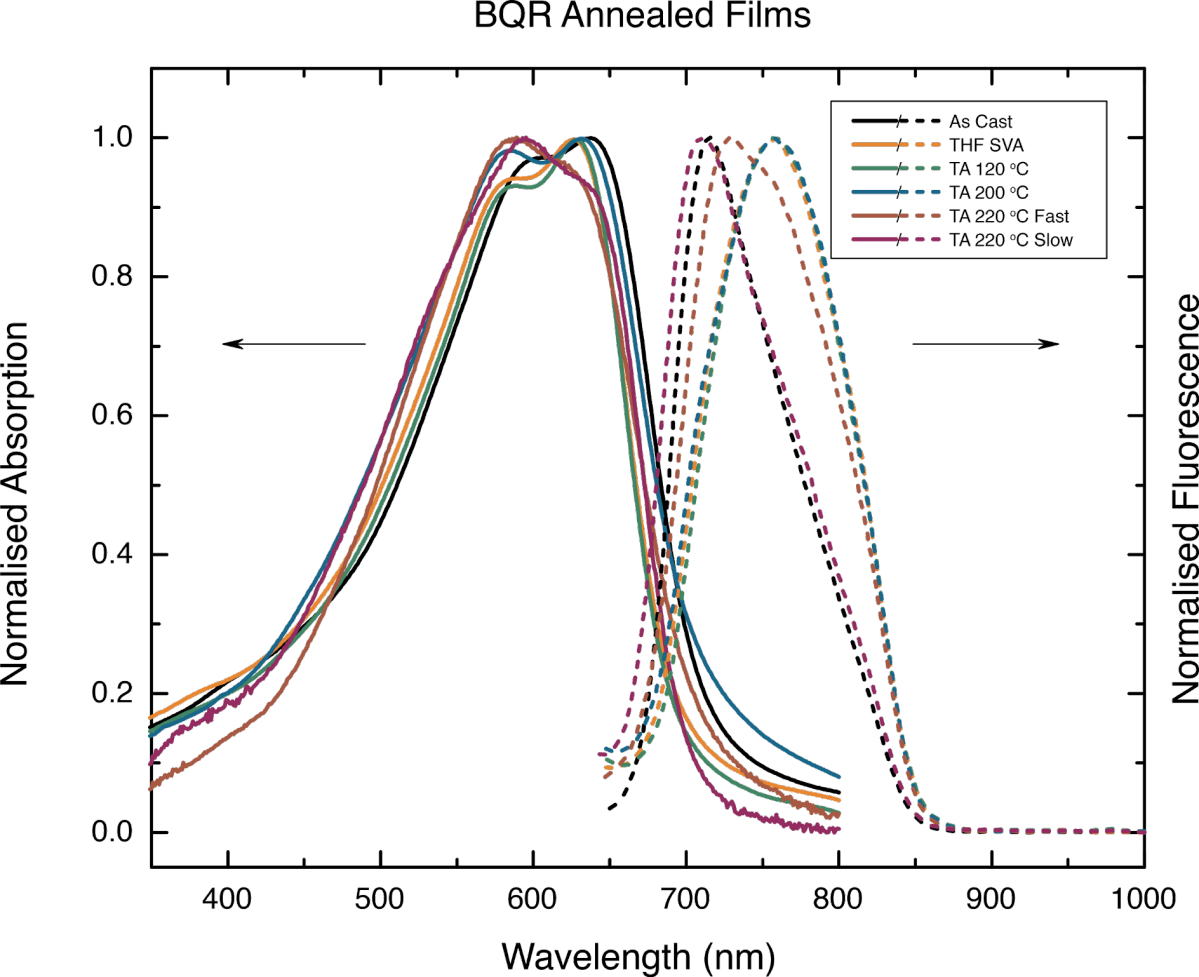
**BQR** thin film UV–vis (solid lines) and fluorescence emission spectra (dashed lines) collected at rt, after SVA and thermal annealing at 120 °C, 200 °C, and 220 °C (above the NLC phase change temperature). Films heated to 220 °C were either removed from the hotplate and cooled (fast) or left on the hotplate to cool (slow).

The appearance of the second fluorescence emission peak in variable temperature spectra on the POM stage, again not seen on the thin films (Figure 7c), has been examined in more detail. The thin films used to obtain the UV–vis spectra, were heated to above the NLC phase change temperature, and collected fluorescence emission spectra are shown in Figure 10. It is clear that when the thin films are heated to 220 °C, i.e., above the NLC phase change temperature, a second blue shifted peak appears at 730 nm, however this is not at the same position recorded on films heated on the POM stage (690 nm). The thin films for these tests were heated to the annealing temperature and after the set time the microscope slide was removed, causing rapid cooling (220 °C fast in Figure 10). It is possible that the rate of heating/cooling impacts on the crystallization of the thin films, with the slower rates used for the fully enclosed, temperature ramped POM stage, leading to equilibrium phases, while rapid thermal quenching of isolated thin films on glass slides gives different results. To further probe this effect, the cooling rate for the thin film heated to 220 °C was modified by turning off the hotplate and allowing the thin film to cool slowly (220 °C slow in Figure 10). When the film is left to cool at a slow rate (220 °C to room temperature over 45 min) the emission spectrum is an almost perfect replica as for the as-cast film emission. Detailed variable temperature X-ray analysis of **BQR** thin films is currently underway to better understand these changes.

**CV and PESA**: The electrochemical properties of the **BXR** series of materials have been examined by cyclic voltammetry (CV), photoelectron spectroscopy in air (PESA), and UV–vis to determine approximate energy HOMO–LUMO energy levels, and the data are summarized in Table 3. From the UV–vis absorption onsets, determined from the as-cast thin films, we determined the *E*_g(opt)_ levels, which demonstrate a clear trend in the reduction of *E*_g(opt)_ on increasing the conjugation length in the **BXR** series dropping from 1.92 eV to 1.74 eV. Pseudo reversible oxidation potentials in the CVs have been recorded on thin films for each of the materials and the data are listed in Table 3, (see Figure S7.1 Supporting Information File 1 for the cyclic voltammograms). The CV data show the expected gradual increase in HOMO levels as we increase the conjugation length and the expected general downward trend in energy gap from 2.40 eV to 1.77 eV for the **BXR** series. The first reduction potential could also be measured allowing an estimation of the LUMO levels for the **BXR** series and therefore an electrochemical energy gap (*E*_g(CV)_), and these match well to the *E*_g(opt)_ values reported above and listed in Table 3.

**Table 3:** Extracted UV–vis absorption peak position for **BX^x^R**.

	UV−vis	Cyclic voltammetry			PESA	DFT Calculations		

**BXR**	*E*_g(opt)_ (eV)	HOMO (eV)	LUMO (eV)	*E*_g(CV)_ (eV)	HOMO (eV)	HOMO (eV)	LUMO (eV)	*E*_g(Theory)_ (eV)

**BMR**	1.92	−5.79	−3.39	2.40	−5.51	−5.67	−3.14	2.53
**BBR**	1.83	−5.55	−3.45	2.10	−5.39	−5.50	−3.01	2.49
**BTR**	1.80	−5.43	−3.46	1.97	−5.20	−5.36	−2.93	2.43
**BQR**	1.74	−5.33	−3.53	1.80	−5.09	−5.26	−2.88	2.38
**BPR**	1.73	−5.29	−3.52	1.77	−5.02	−5.19	−2.85	2.34
**BT^4^R**	1.73	−5.44	−3.46	1.98	−5.21	−	−	−
**BT^8^R**	1.72	−5.49	−3.48	2.01	−5.14	−	−	−

Ionisation potentials have also been measured by photoelectron spectroscopy in air (PESA), and give a direct measure of the HOMO level which rises from −5.51 eV for **BMR** through to −5.02 eV for **BPR** (see Figures S10.1–S10.5). There is a good correlation between the HOMO energy levels measured by CV and PESA.

**Figure 11:**
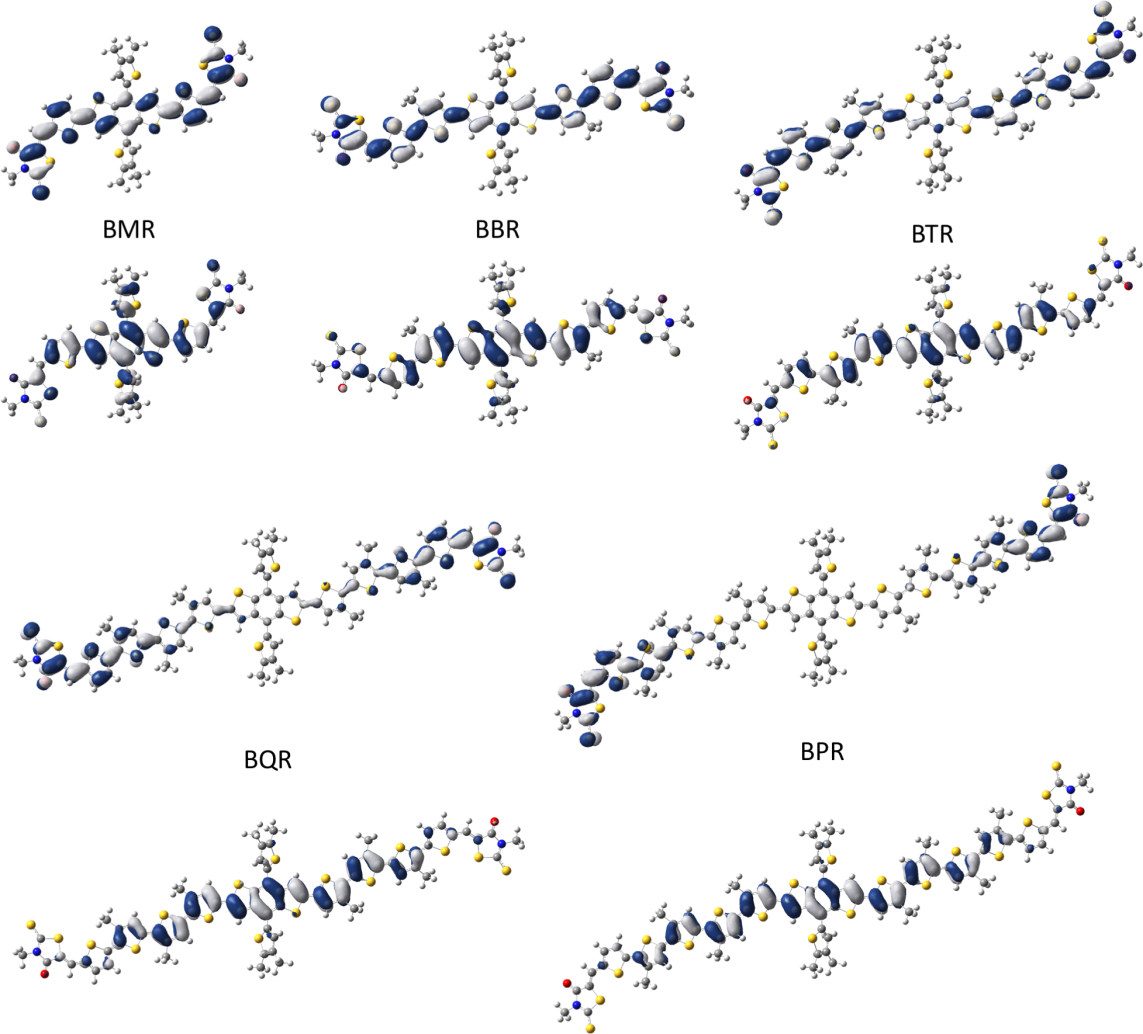
Optimized geometry and molecular orbital surfaces of HOMO (bottom) and LUMO (top) for the **BXR** series. Calculated at the B3LYP-D2/6-311G(d,p)//PBE0/def2-TZVP level. Alkyl chains have been substituted by methyl groups.

**DFT calculations**. To further understand the impact on varying the conjugation length of the oligothiophene bridging arm on the distribution of the HOMO/LUMO energy levels and overlap, density functional theory (DFT) calculations were performed. Geometry optimization and molecular orbital surfaces were determined and are shown in Figure 11. Geometries of the **BXR** series were obtained at the D2 dispersion corrected B3LYP/6-311G(d,p) level of theory. Subsequent time-dependent DFT (TD-DFT) calculations were carried out on the optimized structures with PBE0/def2-TZVP level of theory based on our benchmark calculations (Supporting Information File 1, chapter S11). It is apparent in Figure 11 that as the **BXR** molecular materials increase in size the overlap of the HOMO and LUMO decreases. The HOMO of the **BXR** series extends as the number of the thiophene rings increases. In contrast, the LUMO becomes more localized towards the *N*-hexylrhodamine acceptor moiety as the conjugation length increases. The calculated HOMO values and HOMO–LUMO energy difference follows the same trend as the observed values determined by CV and PESA.

**Figure 12:**
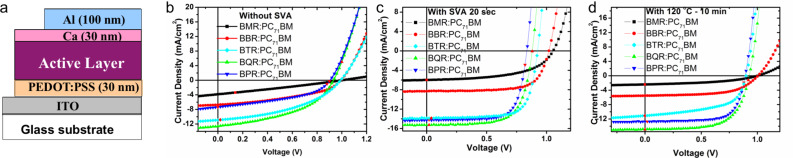
*J–V* characteristics of **BXR**:**PC_71_BM** BHJ solar cells. (a) device structure, (b) *J–V* curves for as-cast, (c) *J–V* curves for SVA with THF, (d) and *J–V* curves for TA at 120 °C for 10 min.

**Photovoltaic performances**. The **BX^x^R** series of materials were incorporated into bulk heterojunction devices with a conventional architecture, i.e. ITO/PEDOT:PSS/active layer/Ca/Al (Figure 12a). The active layer composition was held at 1:1 **BX^x^R**:**PC_71_BM** (by weight) and deposited from CHCl_3_. The active layer was ≈250 nm thick and Ca/Al was used as the back cathode.

We report here preliminary BHJ device data to indicate the impact of small structural variations on the device performance. Further device optimization is currently being completed and will be reported at a later date. Devices assembled with as-cast films (Table 4, entries 1–5 and *J–V* curves Figure 12b) show acceptable device performance without annealing, with **BQR** delivering the best device performance at 5.3%. All devices show high open circuit voltages (*V*_oc_) above 0.90 V, but low fill factors (FF) <45%.

Using previously optimized SVA conditions for **BTR**, THF for 10 seconds, devices based on the **BXR** series were fabricated and device data collected, see Table 4 (entries 6–10) and *J–V* curves in Figure 12c. The *V*_oc_ decreases from 1.04 V for **BMR** to 0.82 V for **BPR** as the conjugation length increases. The *V*_oc_ drop reflects the measured increase in the HOMO level across the series from −5.51 eV for **BMR** to –5.02 for **BPR**. The measured device data for **BTR** with *J*_sc_ = 13.9 mA cm^−2^, *V*_oc_ = 0.92 V, FF of 72% and PCE of 9.3% are almost identical to those previously reported at *J*_sc_ = 13.9 mA cm^−2^, *V*_oc_ = 0.90 V, FF 74.1% and PCE of 9.3% [14], showing the batch to batch reproducibility in device data for molecular materials.

Except for **BMR**, the FFs for SVA devices lie above 70%, indicating excellent morphology development. The best device contains **BQR** with a PCE of 9.4% and a *J*_sc_ = 15.3 mA cm^−2^. **BPR** shows promise with a high FF (74%), however a lower *V*_oc_ (0.82 V) and a reduced *J*_sc_ (14.3 mA cm^−2^) reduce the PCE to 8.7%. UV–vis data indicate that under these SVA conditions the π–π stacking is not fully developed indicating that optimizing SVA conditions may lead to improved light harvesting.

**Table 4:** Photovoltaic performances of **BXR**:**PC_71_BM** BHJ solar cells fabricated under different annealing conditions.

	Active layer	Annealing conditions	*J*_sc_ (mA/cm^2^)	*V*_oc_ (V)	FF (%)	PCE (%)

1	**BMR**:**PC_71_BM**	as cast	3.9	1.00	26	1.0
2	**BBR**:**PC_71_BM**	as cast	6.7	0.98	43	2.8
3	**BTR**:**PC_71_BM**	as cast	10.8	0.98	43	4.6
**4**	**BQR:PC_71_BM**	**as cast**	**12.6**	**0.94**	**45**	**5.3**
5	**BPR**:**PC_71_BM**	as cast	7.5	0.90	45	3.0

6	**BMR**:**PC_71_BM**	SVA 20 s	6.0	1.04	56	3.5
7	**BBR**:**PC_71_BM**	SVA 20 s	8.4	1.00	71	6.0
8	**BTR**:**PC_71_BM**	SVA 20 s	13.9	0.92	72	9.3
**9**	**BQR:PC_71_BM**	**SVA 20 s**	**15.3**	**0.88**	**70**	**9.4**
10	**BPR**:**PC_71_BM**	SVA 20 s	14.3	0.82	74	8.7

11	**BMR**:**PC_71_BM**	TA 120 °C, 10 min	2.5	1.00	44	1.1
12	**BBR**:**PC_71_BM**	TA 120 °C, 10 min	5.5	1.00	60	3.3
13	**BTR**:**PC_71_BM**	TA 120 °C, 10 min	11.0	0.88	58	5.7
**14**	**BQR:PC_71_BM**	**TA 120 °C, 10 min**	**14.9**	**0.92**	**65**	**8.9**
15	**BPR**:**PC_71_BM**	TA 120 °C, 10 min	12.8	0.88	71	8.1

It is important for commercialization of printed BHJ devices that any active layer can withstand the requirements of a printing process, which normally requires a drying or curing step for printed electrodes of >80 °C. To evaluate our new materials for possible translation to a printing process devices incorporating the **BXR** series of donors were assembled and the active layer thermally annealed at 120 °C for 10 minutes before electrode deposition, data collected from the devices are listed in Table 4 (entries 11–15) and *J–V* curves are shown in Figure 12d. The thermally annealed devices do not show as clear a trend as seen for SVA annealed devices with, e.g., no clear systematic decrease in the *V*_oc_s on going from **BMR** through to **BPR**. Also, apart from **BPR**, device FFs remain below 70%. This suggests that further device optimization is required. The device performances of **BMR** (PCE 1.1%), **BBR** (PCE 3.3%), and **BTR** (PCE 5.7%) are significantly lower than the SVA devices, primarily due to lower FFs and *J*_sc_ values.

Both **BQR** (PCE 8.9%) and **BPR** (PCE 8.1%) do not show significant performance loss after thermal annealing, maintaining good FF’s, *J*_sc_ values and *V*_oc_’s. The drop in performance compared to the SVA devices indicates that further optimization may be required.

It is evident that modification of the chromophore length has a large impact on the device stability and performance. **BQR** as a molecular electron donor is the stand-out performer with the best initial results under all device assembly conditions, and shows thermal stability compatible with printing processes.

**Figure 13:**
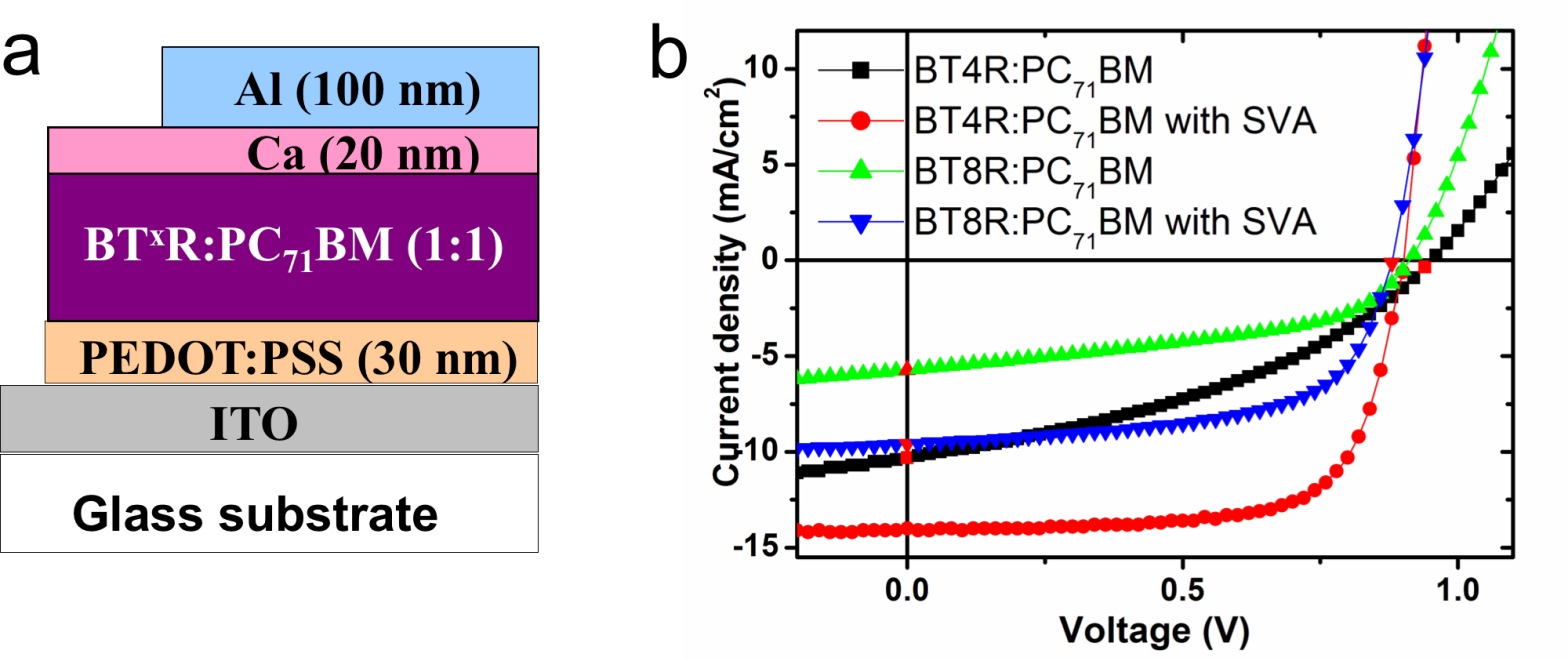
*J–V* characteristics of **BT^x^R**:**PC_71_BM** ternary BHJ solar cells, a) device architecture, and b) *J–V* curve for **BT^x^R** containing devices.

The influence on the oligo-thiophene alkyl chain length on molecular packing, and thereby device performance, was examined in the **BT^x^R** series. BHJ devices using **BT^4^R** and **BT^8^R** were assembled using the same device architecture described above. The collected device data are summarized in Table 5, and the *J–V* curves are shown in Figure 13. Examination of the **BT^x^R** UV–vis data for as-cast films (Figure 5d) indicates that **BT^8^R** does not have a well-developed π–π stacking peak in as-cast films, unlike **BTR**. Also, both **BT^4^R** and **BT^8^R** are blue-shifted in comparison to **BTR**, by 18 nm and 26 nm respectively for **BT^4^R** and **BT^8^R**. As it is not expected that modifications of the oligothiophene bridge side-chain length should significantly impact the chromophore energy levels, variations in measured properties will be due to impacts of side-chain variation on intra-/intermolecular interactions. The differences are reflected in the performance of **BT^4^R** and **BT^8^R** containing devices, Table 5, entries 1–3 and the *J–V* curves reproduced in Figure 13b, where the device efficiency for **BTR** at 4.6% PCE remains above that for **BT^4^R** (3.8% PCE) and **BT^8^R** (2.4% PCE). The major change is a significant drop in short circuit current for **BT^8^R** down to 5.7 mA cm^−2^, from over 10.3 mA cm^−2^ for **BTR** and **BT^4^R**. The open circuit voltage is also lower for both **BT^4^R** and **BT^8^R** in comparison to **BTR**, however there is no obvious trend.

**Table 5:** Photovoltaic performances of **BT^X^R:PC_71_BM** BHJ solar cells fabricated under different annealing conditions.

	Active layer	Annealing conditions	*J*_sc_ (mA/cm^2^)	*V*_oc_ (V)	FF (%)	PCE (%)

1	**BT^4^R**:**PC_71_BM**	as cast	10.3	0.94	39	3.8
2^a^	**BTR**:**PC_71_BM**	as cast	10.8	0.98	43	4.6
3	**BT^8^R**:**PC_71_BM**	as cast	5.7	0.92	46	2.4

4	**BT^4^R**:**PC_71_BM**	SVA 20 s	14.0	0.88	73	9.0
5^a^	**BTR**:**PC_71_BM**	SVA 20 s	13.9	0.92	72	9.3
6	**BT^8^R**:**PC_71_BM**	SVA 20 s	9.6	0.88	62	5.2

^a^**BTR** data from Table 4 has been reproduced to aid in data interpretation.

After SVA the UV–vis spectra for **BT^4^R** and **BT^8^R** match more closely that for **BTR**, however the π–π stacking peak remains poorly resolved for **BT^8^R**. Again this is reflected in the lower device performance for **BT^8^R** (5.2% PCE) in comparison to **BTR** (9.3% PCE) and **BT^4^R** (9.0% PCE). In fact, the device parameters for **BT^4^R** are almost identical to those for **BTR**, except for a significant drop in *V*_oc_ to 0.88 V from 0.92 V. One can only speculate on the cause of the *V*_oc_ drop until further structural characterisation of the thin films is completed. The poor *J*_sc_ and FF for the **BT^8^R** devices indicates a poor development of morphology and indicates that devices optimization is still required.

**Figure 14:**
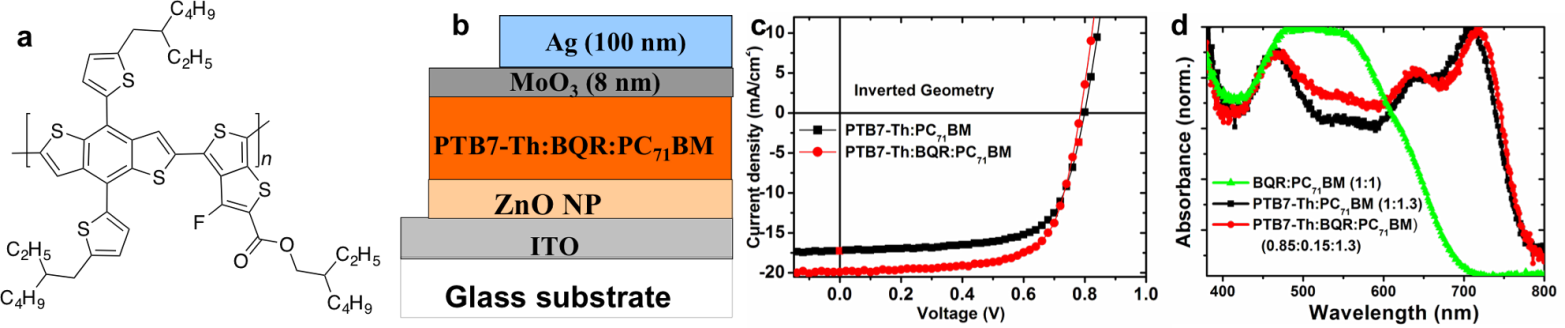
*J–V* characteristics of **PTB7-Th**:**BQR**:**PC_71_BM** ternary BHJ solar cells. (a) **PTB7-Th** chemical structure, (b) inverted device architecture, and (c) *J–V* curve for **BQR** containing ternary device, and (d) UV–vis absorption spectra of ternary and binary blend active layers **BQR**:**PC_71_BM**, **PTB7-Th**:**PC_71_BM** and **PTB7-Th**:**BQR**:**PC_71_BM**.

The performance of **BQR** as a molecular electron donor and the stability of **BQR** containing BHJ devices encouraged the examination of **BQR** in ternary BHJ devices. It has been reported that addition of a small percentage of a molecular electron donor to polymer:fullerene BHJ devices leads to an improvement in overall device performance [22]. The underlying reason for the improved performance in these ternary devices is not yet clear with a combination of favourable morphology, energy level cascading and recombination in the ternary blend being suggested [23–24], however the performance enhancement is real and reproducible. Ternary blend devices containing **BQR** have been assembled using poly[4,8-bis(5-(2-ethylhexyl)thiophen-2-yl)benzo[1,2-*b*:4,5-*b*′]dithiophene-*alt*-3-fluorothieno[3,4-*b*]thiophene-2-carboxylate] (**PTB7-Th**) as the polymeric electron donor, Figure 14a, as we expected the similarities of the BDT cores to allow better interaction between **BQR** and **PTB7-Th**, if this is important. Inverted devices with a structure ITO/ZnO/**PTB7-Th**:**BQR**:**PC_71_BM**/MoO_3_/Ag were assembled, and device data are collected in Table 6 and *J–V* curves are shown in Figure 14c. Devices were spun cast from chlorobenzene containing 3% diiodooctane as a processing additive. When **PTB7-Th** was as the polymeric donor with our standard inverted device architecture and processing conditions, we were able to assemble BHJ devices with a PCE of 9.6%. For these **PTB7-Th** only devices we achieved a good *J*_sc_ =17.2 mA cm^−2^ and a FF of 69% with the expected *V*_oc_ of 0.80 V for devices containing **PTB7-Th** (Table 6, entry 1). These results compare very well with previously reported devices containing **PTB7-Th**:**PC_71_BM** as the active layer, with a similar simple device architecture (see for example ref [25], *J*_sc_ =17.23 mA cm^−2,^ FF 63.42%, *V*_oc_ of 0.793 V, and a PCE 8.81%, 1:1.5 **PTB7-Th**:**PC_71_BM**).

**Table 6:** Photovoltaic Performances of **PTB7-Th**:**BQR**:**PC_71_BM** ternary BHJ solar cells.

	Active layer	Ratio	*J*_sc_ (mA cm^−2^)	*V*_oc_ (V)	FF (%)	PCE (%)

1	**PTB7-Th**:**PC_71_BM**	1:1.3	17.2	0.80	69	9.6
2	**PTB7-Th**:**BQR**:**PC_71_BM**	0.85:0.15:1.3	19.8	0.80	68	10.7

Inclusion of 15 wt % of **BQR** in the donor phase resulted in a significantly enhanced efficiency from 9.6% to 10.7% PCE, with increase in the device *J*_sc_ to 19.8 mA cm^–2^, while the *V*_oc_ and FF remain effectively unchanged. To investigate the enhanced performance due to addition of **BQR**, we have studied the absorption spectra (Figure 14d) of the ternary and binary blend films. The normalized absorption intensity of ternary blend (**PTB7-Th**:**BQR**:**PC_71_BM**) active layer shows the enhanced absorption intensities in the wavelength range between 500–600 nm comparing to the binary blend (**PTB7-Th**:**PC_71_BM**). This enhanced absorption in the ternary blend is due to the inclusion of **BQR** as confirmed from the absorption spectrum of **BQR**: **PC_71_BM**, which shows the absorption maximum in the wavelength range 500–600 nm. Further device optimization and active layer morphology investigation for enhanced performance of ternary blend OPV devices are being explored currently and the results will be communicated elsewhere.

## Conclusion

We have reported a simplified synthesis of alkylsubstituted oligothiophenes used as π-bridges in A–π-D–π-A molecular electron donors via chain extension catenation of alkylthiophenes. We have used commercially available 3-butyl-, 3-hexyl- and 3-octylthiophene to form the key intermediate TMS-alkylthiophene boronic acid pinacol esters (**3**) in high yield on a large scale and in high purity as they can be purified by distillation. Access to the mono-, bis-, ter-, quater-, and quinquethiophene π-bridge oligothiophenes by alkylthiophene catenation has allowed the synthesis of chromophore extended versions of the previously reported **BTR**, the **BXR** series of materials, that is **BMR** (X = M, monothiophene), **BBR** (X = B, bithiophene), the known **BTR** (X = T, terthiophene), **BQR** (X = Q, quaterthiphene), and the **BPR** (X = P, quinquethiophene). The impact of the oligothiophene alkyl side-chain on OPV device performance was studied using the 3-butyl and 3-octylthiophene starting materials to generate the **BT^X^R** analogues with butyl- and octyl-substituted oligothiophene π-bridges, the **BT^x^R** series of materials, where x = 4 (butyl) and x = 8 (octyl). Thin films of the pure materials have been analysed by UV–vis absorption spectroscopy which indicated that extension of the **BXR** chromophore through oligothiophene extension and side-chain variation impacts significantly on the development of highly π–π stacked materials. Shorter chromophore length leads to good stacking in thin films with dominant absorption transitions even in as-cast films for **BMR**, **BTR** and **BQR**. Molecular organization is improved in all films with SVA, except for the longest chromophore (**BPR**), where good π–π stacking is not observed, even on extended thermal annealing.

Small changes in the molecular structures lead to larger impacts on the thermal behaviour of the materials. DSC thermograms for materials indicate that short and long chromophores (**BMR**, **BBR**, and **BPR**) show single phase changes, while **BTR** and **BQR** show a number of phase changes and high temperature NLC phases. Surprisingly when **BT^4^R** was examined using DSC a single exothermic and endothermic peak were recorded, however when the materials were examined by POM a high temperature NLC phase was observed indicating a rich and more complicated phase space than indicated by simple thermal analysis. For **BQR**, variable temperature UV–vis spectroscopy mapped the transmitted light variations that accompany the phase change noted in the POM studies of **BQR**. We are currently studying the structural changes occurring in thin films of our **BX^x^R** materials to better understand the changes observed and these will be reported in due course.

All the new materials have been tested as electron donors in OPV devices with **PC_71_BM** as the acceptor with thick active layers (approx. 250 nm). The preliminary results show interesting patterns with good OPV device performance for both solvent vapour and thermally annealed devices, up to 9.4% PCE. Device performance improved with chromophore extension in SVA devices increasing from 3.5% PCE for **BMR** containing devices through to 9.4% PCE for **BQR**. The results indicated an improved performance for **BQR** over that for **BTR** previously reported, 9.3% PCE, also reproduced for materials made in this study with our modified procedure. Further extension of the chromophore length in these preliminary studies, for **BPR**, results in a lower PCE of 8.7%, mainly due to a lower *J*_sc_ and *V*_oc_. However, again with a FF of 74% there is scope for device improvements through more optimization.

Device performance improved with chromophore extension in TA devices, increasing from 1.1% PCE for **BMR** through to 8.9% PCE for **BQR**. The results indicated an improved thermal stability for OPV devices containing **BQR** over that for **BTR** previously reported. Incorporating **BPR** in OPV devices, with the longest chromophore length in this study, also resulted in thermally stable devices, but with a lower PCE of 8.1%, mainly due to a lower *J*_sc_ and *V*_oc_. However, with a FF of 71%, the highest in this thermally annealed series, there is again scope for device improvements through more optimization.

In an extension of these studies, we have used the best material (**BQR**) as a secondary donor in ternary blend devices with commercially available **PTB7-Th** as the main polymeric donor. In initial studies using these ternary blends we have recorded OPV device efficiencies of up to 10.7% PCE. The improved efficiency in these devices is a result of a significantly higher *J*_sc_, rising from 17.2 to 19.8 mA cm^−2^, with no significant change in *V*_oc_ or FF.

Therefore, we have shown using a simplified synthesis that chain extended chromophores can be accessed, and thereby the thermal stability of OPV devices containing these new materials can be improved. We are currently examining **BQR** in printed solar cells.

In all cases in our structure–property relationship studies, devices incorporating materials that exhibited a high temperature NLC phase gave the best results. The role of the high temperature NLC behaviour in device performance remains unclear, and as we do not anneal to temperatures where the NLC phase change temperature is reached, its presence is unlikely. However, it may be that structural properties leading to a high temperature NLC phase may help to pre-organise the donor material into a morphology best suited for OPV devices. We are currently probing the structure of these materials in thin films, and these results will be published in the near future, along with device optimization studies and translation to large area devices.

In summary, we have developed a simplified synthetic route to afford a range of MMs analogues of **BTR**. This simplified route has allowed large-scale synthesis of intermediate building blocks and of a multi-gram synthesis of the required MMs. Detailed structure–property studies have identified **BQR** and **BPR** as excellent materials for further optimization with an improved performance over **BTR**. OPV devices containing **BQR** or **BPR** show a good thermal stability at 120 °C for 10 min, maintaining a high PCE (**BQR**, 8.9% and **BPR**, 8.1%) and FF (**BQR**, 65% and **BPR**, 71%). These are promising results for high performance OPV devices and the translation to large area and printed OPV devices.

## Supporting Information

File 1 Synthetic procedures, NMR spectra, MALDI, TGA, DSC, CVs, POM methods and images, UV–vis, fluorescence, and DFT cartesian coordinates.
