# Supplementary material for: High performance p-type molecular electron donors for OPV applications via alkylthiophene catenation chromophore extension
Source: Beilstein J Org Chem. 2016 Nov 2;12:2298–314. doi: 10.3762/bjoc.12.223 (PMC5238583; doi:10.3762/bjoc.12.223)
Supplement: File 1 — Synthetic procedures, NMR spectra, MALDI, TGA, DSC, CVs, POM methods and images, UV–vis, fluorescence, and DFT cartesian coordinates. [file Beilstein_J_Org_Chem-12-2298-s001.pdf]

Supporting Information
for

High performance p-type molecular donors for OPV via alkylthiophene catenation chromophore extension.

Paul B. Geraghty,[1] Calvin Lee,[1] Jegadesan Subbiah,[1] Wallace W. H. Wong,[1] James L. Banal,[1] Mohammed A. Jameel,[2] Trevor A. Smith,[2] and David J. Jones*[1]

Address: [1]School of Chemistry, Bio21 Institute, University of Melbourne, Parkville, Melbourne, Australia, 3010 and [2]School of Chemistry, University of Melbourne, Parkville, Melbourne, Australia, 3010.

Email: David J. Jones* - djjones@unimelb.edu.au
*Corresponding author

Synthetic procedures, NMR spectra, MALDI, TGA, DSC, CVs, POM methods and images, UV–vis, fluorescence, and DFT cartesian coordinates

S1 Materials and methods S1

S2 Synthesis S2

S2.1 Synthesis of 4-alkyl-5-(4,4,5,5-tetramethyl-1,3,2-dioxaborolan-2-yl)thiophen-2-yl)trimethylsilanes S2

S2.1.1 Synthesis of (4-butylthiophen-2-yl)trimethylsilane (**2a**) S2

S2.1.2 Synthesis of (4-hexylthiophen-2-yl)trimethylsilane (**2b**) S3

S2.1.3 Synthesis of (4-octylthiophen-2-yl)trimethylsilane (**2c**) S3

S2.1.4 Synthesis of (4-butyl-5-(4,4,5,5-tetramethyl-1,3,2-dioxaborolan-2-yl)thiophen-2-yl)trimethylsilane (**3a**) S4

S2.1.5 Synthesis of (4-hexyl-5-(4,4,5,5-tetramethyl-1,3,2-dioxaborolan-2-yl)thiophen-2-yl)trimethylsilane (**3b**) S4

S2.1.6 Synthesis of (4-octyl-5-(4,4,5,5-tetramethyl-1,3,2-dioxaborolan-2-yl)thiophen-2-yl)trimethylsilane (**3c**) S5

S2.2 Synthesis of oligothiophenes 4-11 S5

S2.2.1 Synthesis of 3'-butyl-5'-(trimethylsilyl)-[2,2'-bithiophene]-5-carbaldehyde (**4a**) S5

S2.2.2 Synthesis of 3'-hexyl-5'-(trimethylsilyl)-[2,2'-bithiophene]-5-carbaldehyde (**4b**) S6

S2.2.3 Synthesis of 3'-octyl-5'-(trimethylsilyl)-[2,2'-bithiophene]-5-carbaldehyde (**4c**) S7

S2.2.4 Synthesis of 3'-butyl-5'-iodo-[2,2'-bithiophene]-5-carbaldehyde (**5a**) S7

S2.2.5 Synthesis of 3'-hexyl-5'-iodo-[2,2'-bithiophene]-5-carbaldehyde (**5b**) S8

S2.2.6 Synthesis of 3'-octyl-5'-iodo-[2,2'-bithiophene]-5-carbaldehyde (**5c**) S8

S2.2.7 Synthesis of 3',3''-dibutyl-5''-(trimethylsilyl)-[2,2':5',2''-terthiophene]-5-carbaldehyde (**6a**) S9

S2.2.8 Synthesis of 3',3''-dihexyl-5''-(trimethylsilyl)-[2,2':5',2''-terthiophene]-5-carbaldehyde (**6b**) S9

S2.2.9 Synthesis of 3',3''-dioctyl-5''-(trimethylsilyl)-[2,2':5',2''-terthiophene]-5-carbaldehyde (**6c**) S10

S2.2.10 Synthesis of 3',3''-dibutyl-5''-iodo-[2,2':5',2''-terthiophene]-5-carbaldehyde (**7a**) S10

S2.2.11 Synthesis of 3',3''-dihexyl-5''-iodo-[2,2':5',2''-terthiophene]-5-carbaldehyde (**7b**) S11

S2.2.12 Synthesis of 3',3''-dioctyl-5''-iodo-[2,2':5',2''-terthiophene]-5-carbaldehyde (**7c**) S11

S2.2.13 Synthesis of 3',3'',3'''-trihexyl-5'''-(trimethylsilyl)-[2,2':5',2'':5'',2'''-quaterthiophene]-5-carbaldehyde (**8b**) S12

S2.2.14 Synthesis of 3',3'',3'''-trihexyl-5'''-iodo-[2,2':5',2'':5'',2'''-quaterthiophene]-5-carbaldehyde (**9b**) S12

S2.2.15 Synthesis of 3',3'',3''',3''''-tetrahexyl-5''''-(trimethylsilyl)-[2,2':5',2'':5'',2''':5''',2''''-quinquethiophene]-5-carbaldehyde (**10b**) S13

S2.2.16 Synthesis of 3',3'',3''',3''''-tetrahexyl-5''''-iodo-[2,2':5',2'':5'',2''': 5''',2''''-quinquethiophene]-5-carbaldehyde (**11b**) S13

S2.3 Synthesis of BX^x-dialdehyde core molecule S15

S2.3.1 Synthesis of 2,2'-(4,8-bis(5-(2-ethylhexyl)-4-hexylthiophen-2-yl)benzo[1,2-b:4,5-b']dithiophene-2,6-diyl)bis(4,4,5,5-tetramethyl-1,3,2-dioxaborolane) (**13**) S15

S2.3.2 Synthesis of 5,5'-(4,8-bis(5-(2-ethylhexyl)-4-hexylthiophen-2-yl)benzo[1,2-b:4,5-b']dithiophene-2,6-diyl)bis(thiophene-2-carbaldehyde)) (**BM**-dialdehyde) S15

S2.3.3 Synthesis of 5',5'''-(4,8-bis(5-(2-ethylhexyl)-4-hexylthiophen-2-yl)benzo[1,2-b:4,5-b']dithiophene-2,6-diyl)bis(3'-hexyl-[2,2'-bithiophene]-5-carbaldehyde) (**BB**-dialdehyde) S16

S2.3.4 Synthesis of 5'',5''''-(4,8-bis(5-(2-ethylhexyl)-4-hexylthiophen-2-yl)benzo[1,2-b:4,5-b']dithiophene-2,6-diyl)bis(3',3''-dibutyl-[2,2':5',2''-terthiophene]-5-carbaldehyde) (**BT**^{4}-dialdehyde) S16

S2.3.5 Synthesis of 5'',5''''-(4,8-bis(5-(2-ethylhexyl)-4-hexylthiophen-2-yl)benzo[1,2-b:4,5-b']dithiophene-2,6-diyl)bis(3',3''-dihexyl-[2,2':5',2''-terthiophene]-5-carbaldehyde) (**BT**-dialdehyde) S17

S2.3.6 Synthesis of 5'',5''''-(4,8-bis(5-(2-ethylhexyl)-4-hexylthiophen-2-yl)benzo[1,2-b:4,5-b']dithiophene-2,6-diyl)bis(3',3''-dioctyl-[2,2':5',2''-terthiophene]-5-carbaldehyde) (**BT**^{8}-dialdehyde) S18

S2.3.7 Synthesis of 5''',5''''''-(4,8-bis(5-(2-ethylhexyl)-4-hexylthiophen-2-yl)benzo[1,2-b:4,5-b']dithiophene-2,6-diyl)bis(3',3'',3'''-trihexyl-[2,2':5',2'':5'',2'''-quaterthiophene]-5-carbaldehyde) (**BQ**-dialdehyde) S18

S2.3.8 Synthesis of 5'''',5''''''''-(4,8-bis(5-(2-ethylhexyl)-4-hexylthiophen-2-yl)benzo[1,2-*b*:4,5-*b'*]dithiophene-2,6-diyl)bis(3',3'',3''',3''''-tetrahexyl-[2,2':5',2'':5'',2''':5''',2''''-quinquethiophene]-5-carbaldehyde) (**BP**-dialdehyde) S19

S2.4 Synthesis of BXR and BT^{x}R molecules S20

S2.4.1 Synthesis of **BMR** molecule (5z,5'z)-5,5'-((5,5'-(4,8-bis(5-(2-ethylhexyl)-4-hexylthiophen-2-yl)benzo[1,2-b:4,5-b']dithiophene-2,6-diyl)bis(thiophene-5,2-diyl))bis(methanylylidene))bis(3-hexyl-2-thioxothiazolidin-4-one) S20

S2.4.2 Synthesis of **BBR** molecule (5z,5'z)-5,5'-((5',5'''-(4,8-bis(5-(2-ethylhexyl)-4-hexylthiophen-2-yl)benzo[1,2-b:4,5-b']dithiophene-2,6-diyl)bis(3'-hexyl-[2,2'-bithiophene]-5',5-diyl))bis(methanylylidene))bis(3-hexyl-2-thioxothiazolidin-4-one) S20

S2.4.3 Synthesis of **BT**^{4}**R** molecule 5'',5''''-(4,8-bis(5-(2-ethylhexyl)-4-hexylthiophen-2-yl)benzo[1,2-b:4,5-b']dithiophene-2,6-diyl)bis(3',3''-dibutyl-[2,2':5',2''-terthiophene]-5-hexylrhodanine) S21

S2.4.4 Synthesis of **BTR**, (5*z*,5'*z*)-5,5'-((5'',5''''-(4,8-bis(5-(2-ethylhexyl)-4-hexylthiophen-2-yl)benzo[1,2-*b*:4,5-*b'*]dithiophene-2,6-diyl)bis(3',3''-dihexyl-[2,2':5',2''-terthiophene]-5'',5-diyl))bis(methanylylidene))bis(3-hexyl-2-thioxothiazolidin-4-one) S21

S2.4.5 Synthesis of **BT**^{8}**R** molecule 5'',5''''-(4,8-bis(5-(2-ethylhexyl)-4-hexylthiophen-2-yl)benzo[1,2-b:4,5-b']dithiophene-2,6-diyl)bis(3',3''-dioctyl-[2,2':5',2''-terthiophene]-5-hexylrhodanine) () S22

S2.4.6 Synthesis of **BQR** molecule, (5z,5'z)-5,5'-((5''',5''''''-(4,8-bis(5-(2-ethylhexyl)-4-hexylthiophen-2-yl)benzo[1,2-b:4,5-b']dithiophene-2,6-diyl)bis(3',3'',3'''-trihexyl-[2,2':5',2'':5'',2'''-quaterthiophene]-5''',5-diyl))bis(methanylylidene))bis(3-hexyl-2-thioxothiazolidin-4-one) S23

S2.4.7 Synthesis of **BPR** molecule (5z,5'z)-5,5'-((5'''',5''''''''-(4,8-bis(5-(2-ethylhexyl)-4-hexylthiophen-2-yl)benzo[1,2-b:4,5-b']dithiophene-2,6-diyl)bis(3',3'',3''',3''''-tetrahexyl-[2,2':5',2'':5'',2''':5''',2''''-quinquethiophene]-5'''',5-diyl))bis(methanylylidene))bis(3-hexyl-2-thioxothiazolidin-4-one) S23

S3 ^{1}H and ^{13}C spectra of all new compounds. S25

S4 Maldi spectra for the dialdehydes BX^x-dialdehyde and the BX^xR materials S62

S5 TGA thermograms S69

S6 DSC thermograms for the BX^xR materials S70

S7 Cyclic voltammograms for the BX^xR materials S71

S8 Polarising optical microscopy (POM) method and images S72

S9 UV–vis spectra for the BXR and BT^xR series of materials S79

S10 Photoelectron spectroscopy in air (PESA) measurements for the BXR series of materials S84

S11 DFT calculations S88

Section s11.1 **cartesian coordinates** **S88**

Section s11.2 **BMR** **S88**

Section s11.3 **BBR** **S90**

Section s11.4 **BTR** **S92**

Section s11.5 **BQR** **S95**

Section s11.6 **BPR** **S98**

Section s11.7 **TD-DFT energy level calculation** **S101**

S1 Materials and Methods

Unless noted, all materials were reagent grade and used as received without further purification. Anhydrous solvents were prepared by drying HPLC grade solvents using freshly activated molecular sieves. Chromatographic separations were performed using standard column methods with silica gel (Merck 9385 Kieselgel 60). Thin layer chromatography was performed on Merck Kieselgel 60 silica gel on glass (0.25 mm thick).

IR spectra were obtained on a Perkin Elmer Spectrum One FT-IR spectrometer and UV–vis spectra were recorded using a Cary 50 UV–vis spectrometer, a Perkin Elmer Lambda 1050 UV–vis-NIR spectrophotometer, or an Agilent 8453 spectrophotometer. Photoluminescence was measured with a Varian Cary Eclipse fluorimeter. ^1H NMR and ^{13}C NMR spectra were carried out on either a Varian 400 MHz spectrometer, an Agilent 500 MHz spectrometer, or a Varian 600 MHz spectrometer. All NMR data was referenced to the chloroform signal, unless stated otherwise, and peak multiplicity was reported as follows: s = singlet, d = doublet, t = triplet, q = quartet, p = pentet, dd = doublets of doublets, m = multiplet, br = broad). MALDI–TOF mass spectrometry was performed on a Bruker microflex instrument, using chloroform as solvent and dithranol as the assisted matrix. ESI mass spectrometry was performed on a Thermo Scientific Q Exactive Plus Orbitrap LC-MS/MS instrument, using 50% acetonitrile/ 0.1% formic acid as the solvent.

Elemental analyses were obtained commercially through Chemical & Analytical Services Pty. Ltd. (Australia) an Exeter Analytical CE–440 elemental analyzer. Thermal gravimetric analysis (TGA) experiments were carried out with a Mettler Toledo TGA/SDTA851e and differential scanning calorimetry (DSC) experiments were performed on a Perkin-Elmer Sapphire DSC at a ramp rate of 10 °C min^{-1} unless otherwise noted.

Similarly as described in [2], cyclic voltammetry (CV) experiments were performed at a sweep rate of 100 mV/s. CVs were carried out in a three-electrode cell consisting of a glassy carbon working electrode, a platinum wire auxiliary electrode, and a Ag/Ag^+ pseudo-reference electrode. The supporting electrolyte was 0.10 M tetrabutylammonium hexafluorophosphate (Bu_4NPF_6) in DCM. The solutions were deoxygenated by sparging with argon prior to each scan and blanketed with argon during the scans. The glassy carbon working electrode was prepared by polishing with 5 mm alumina and washed and dried before the polymer was drop-casted on the electrode from chlorobenzene solution to form a film. Ferrocene/ferrocenium redox couple was used as the internal standard. The HOMO energy level was calculated from the onset of the oxidation potential of the donor materials using the following: $E_{HOMO} = -(4.8 + E_{ox}$ onset) eV.

Device fabrication and characterization:

Organic photovoltaic devices were processed on pre-patterned indium tin oxide (ITO) coated glass substrates with a sheet resistance of 15 Ω per square. The conventional devices were fabricated with a device geometry of glass/ITO/PEDOT:PSS/active layer/Ca/Al. The ITO-coated glass substrates were cleaned by ultrasonic treatment in acetone and isopropyl alcohol for 15 minutes each and subsequently treated in UV–Ozone for 15 minutes. A thin layer (30 nm) of PEDOT:PSS (Clevios P VP AI 4083, filtered at 0.45 µm) was spin-coated onto the ITO surface. After baked at 150 °C for 10 minutes, the substrates were transferred into a nitrogen-filled glove box. Subsequently, the active layer was spin-coated from blend chloroform solutions (32 mg/mL) with weight ratio of BXR and PC71BM at 1:1 and the active layer thicknesses of all films were

measured as 210–260 nm. Then, the substrates were placed in a glass Petri dish containing 1 mL THF for solvent vapour annealing (SVA). After SVA treatment, the films were transferred to a metal evaporation chamber and a bilayer cathode consisted of Ca (30 nm) capped with Al (100 nm) was deposited through a shadow mask (active area was 0.1 cm^2) at approximately 1 $\times 10^{-6}$ Torr.

For inverted devices, a thin layer of ZnO nanopaticle (NP) was deposited on cleaned ITO substrate by spin-coating (3000 rpm) to form 30 nm of a ZnO layer, followed by backing on a hot plate at 150 °C for 5 min. An active layer of the device was deposited by spin coating with a chlorobenzene solution (1 mL) containing 8.5 mg of PTB7-Th, 1.5 mg of BQR and 13 mg of $PC_{71}BM$ along with 3% of 1,8-diiodooctane (DIO). The thickness of the active layer was measured as 90 nm. The films were then transferred to a metal evaporation chamber and MoO_3 (10 nm) and silver (100 nm) were thermally deposited through a shadow mask (active area was 0.1 cm^2) at approximately 1 × 10^{-6} Torr. Film thickness was determined by a Veeco Dektak 150+Surface Profiler. The current density-voltage measurements of the devices were carried out using a 1 kW Oriel solar simulator with an AM 1.5G filter as the light source in conjunction with a Keithley 2400 source measurement unit. Solar measurements were carried out under 1000 W/m^2 AM 1.5G illumination conditions. For accurate measurement, the light intensity was calibrated using a reference silicon solar cell (PVmeasurements Inc.) certified by the National Renewable Energy Laboratory. Device fabrication and characterizations were performed in an inert environment without any encapsulation.

UV–vis absorption and PESA measurements:

The ionization potential for the organic thin films (BXR and BTXR) on glass was measured by photoelectron spectroscopy in air (PESA) under constant dry air flow using a Riken Keiki AC-2 spectrometer. UV–vis spectra were measured by Cary 50 UV–vis spectrometer.

S2 Synthesis

S2.1 Synthesis of 4-alkyl-5-(4,4,5,5-tetramethyl-1,3,2-dioxaborolan-2-yl)thiophen-2-yl)trimethylsilanes

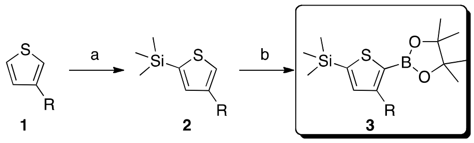

3a: R = butyl
3b: R = hexyl
3c: R = octyl

S2.1.1 Synthesis of (4-butylthiophen-2-yl)trimethylsilane (2a)

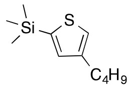

Reagents 3-butylthiophene (**1a**) and diisopropylamine were dried by passage through an alumina column then degassed by bubbling dinitrogen through the reagents for 30 minutes. A solution of *n*-BuLi (100 mL, 2.5 M, 250 mmol) was added drop wise over 1 hour to a solution of 3-butylthiophene (31.5 g, 225 mmol) and diisopropylamine (34.15 g, 337.5 mmol) in 250 mL of THF at −78 °C. The reaction mixture was allowed to warm to rt, then cooled to −78 °C when

chlorotrimethylsilane (28.93 g, 266 mmol) was slowly added. The reaction mixture was allowed to warm to rt and stirred for 1 hour. The reaction was quenched by addition of 250 mL of water. The THF was removed under rotary evaporation and was followed by the addition of 200 mL of diethyl ether. The organic phases were separated, washed with water (2 × 100 mL) then dried over $MgSO_4$. The slurry was filtered to remove the $MgSO_4$, the solvent removed under vacuum and the crude product distilled at 90 °C at 700 mTorr to yield the product as a clear oil (36.1 g, 170 mmol, 77%). IR (neat) ν = 2956, 2929, 2859, 1248, 1006, 820 cm^{-1}. ^1H NMR (600 MHz, $CDCl_3$) δ = 7.17 (s, 1 H), 7.08 (s, 1 H), 2.65 (t, J = 7.8 Hz, 2 H), 1.68-1.59 (m, 2 H), 1.40-1.37 (m, 2 H), 0.95 (t, J = 6.7 Hz, 3 H), 0.33 (s, 9 H). ^{13}C NMR (100 MHz, $CDCl_3$) δ = 144.6, 140.0, 135.5, 125.3, 32.9, 29.7, 22.5, 13.9, 0.01. MS (ESI) m/z $[M+H]^+$ = 212.1494. Elemental analysis calculated for $C_{11}H_{20}SSi$: Calculated: C, 62.19; H, 9.49; S, 15.09; Si, 13.22; found: C, 62.12; H, 9.64; S, 14.92

S2.1.2 Synthesis of (4-hexylthiophen-2-yl)trimethylsilane (2b)

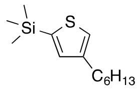

Reagents **1b** (3-hexylthiophene) and diisopropylamine were dried by passage through an alumina column then degassed by bubbling dinitrogen through the reagents for 30 minutes. A solution of *n*-BuLi (97 mL, 2.5 M, 243 mmol) was added drop wise over 1 hour to a solution of 3-hexylthiophene (36.7 g, 218 mmol) and diisopropylamine (33.3 g, 327 mmol) in 250 mL of THF at −78 °C. The reaction mixture was allowed to warm to rt, then cooled to −78 °C when chlorotrimethylsilane (27.72 g, 32.4 mL, 258 mmol) was slowly added. The reaction mixture was allowed to warm to rt and stirred for 1 hour. The reaction was quenched by addition of 250 mL of water. The THF was removed under vacuum (RotoVap), 200 mL of diethyl ether added and the organic phases separated, washed with water (2 × 100 mL) then dried over $MgSO_4$. The slurry was filtered to remove the $MgSO_4$, the solvent removed under vacuum and the crude product distilled. The unreacted hexylthiophene removed and the product then collected which included some 2,5-bis-trimethylsilyl-3-hexylthiophene which could be carried forward without impacting the next reaction. Yield 43.2 g (180 mmol, 82.4%) Analytically pure material could be isolated by careful distillation at 92–94 °C at 220-225 mTorr. IR (neat cm^{-1}) 2956, 2927, 2857, 1249, 1007, 833, 756, 696. ^1H NMR (400 MHz, $CDCl_3$) δ = 7.162 (s, 1 H), 7.066 (s, 1 H), 2.635 (t, J = 7.8 Hz, 2 H), 1.65-1.62 (m, 2 H), 1.36-1.30 (m, 6 H), 0.900 (t, J = 7.8 Hz, 3 H), 0.322 (s, 9 H); ^{13}C NMR (100 MHz, $CDCl_3$) 144.7, 140.0, 135.5, 125.2, 31.9, 30.7, 30.0, 29.1, 22.6, 14.1, -0.03; MS (ESI) m/z $[M+CH_3]^+$ = 255.133. Elemental Analysis calculated for $C_{13}H_{24}SSi$: Calculated C, 64.93; H, 10.06; S, 13.33; Si, 11.68. Found C, 64.95; H, 10.12; S, 13.20.[1]

S2.1.3 Synthesis of (4-octylthiophen-2-yl)trimethylsilane (2c)

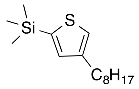

Reagents 3-octylthiophene (**1c**) and diisopropylamine were dried by passage through an alumina column then degassed by bubbling dinitrogen through the reagents for 30 minutes. A

[1] Spivey, A.; Turner, D.; Turner, M.; Yeates, S. *Org. Lett.* **2002**, *4*, 1899–902.

solution of *n*-BuLi (100 mL, 2.5 M, 250 mmol) was added drop wise over 1 hour to a solution of **1c** (44.2 g, 225 mmol) and diisopropylamine (34.15 g, 337.5 mmol) in 250 mL of THF at −78 °C. The reaction mixture was allowed to warm to rt, then cooled to −78 °C when chlorotrimethylsilane (28.93 g, 266 mmol) was slowly added. The reaction mixture was allowed to warm to rt and stirred for 1 hour. The reaction was quenched by addition of 250 mL of water. The THF was removed under rotary evaporation and was followed by the addition of 200 mL of diethyl ether. The organic phases were separated, washed with water (2 × 100 mL) then dried over $MgSO_4$. The slurry was filtered to remove the $MgSO_4$, the solvent removed under vacuum and the crude product distilled at 102 °C at 610 mTorr to yield the product as a clear oil (50.9 g, 180 mmol, 84%). IR (neat cm^{-1}) 2956, 2924, 2854, 1248, 1006, 820; ^{1}H NMR (600 MHz, $CDCl_3$) δ = 7.17 (s, 1 H), 7.08 (s, 1 H), 2.65 (t, J = 7.8 Hz, 2 H), 1.68-1.59 (m, 2 H), 1.40-1.24 (m, 6 H), 0.91 (t, J = 6.7 Hz, 3 H), 0.33 (s, 9 H). ^{13}C NMR (100 MHz, $CDCl_3$) δ = 144.7, 140.0, 135.5, 125.3, 31.9, 30.7, 30.3, 29.5, 29.4, 29.3, 2276, 14.1, -0.01. MS (ESI) m/z $[M+H]^+$ = 269.1751. Elemental analysis calculated for $C_{15}H_{28}SSi$: Calculated: C, 67.09; H, 10.51; S, 11.94; Si, 10.46, found: C: 67.03; H, 10.63

S2.1.4 Synthesis of (4-butyl-5-(4,4,5,5-tetramethyl-1,3,2-dioxaborolan-2-yl)thiophen-2-yl)trimethylsilane (3a)

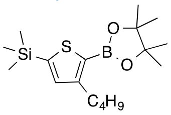

A solution of *n*-BuLi (64 mL, 2.5 M, 160 mmol) was added drop wise over 1 hour to a solution of (4-butylthiophen-2-yl)trimethylsilane (30 g, 141 mmol) in THF (100 mL) at −78 °C. The mixture was allowed to react for 1 hour then 2-isopropoxy-4,4,5,5-tetramethyl-1,3,2-dioxaborolane (62.6 g, 160 mmol) was slowly added. The reaction mixture was allowed to warm to rt and stirred for 1 hour. The reaction was quenched by addition of 100 mL of water. The THF was removed under rotary evaporation and diethyl ether (100 mL) was added with the organic phases separated, washed with water (2 × 100 mL) then dried over $MgSO_4$. The slurry was filtered to remove the $MgSO_4$, the solvent removed under vacuum and the crude product distilled at 150 °C at 700 mTorr to yield a clear, viscous oil (26.53 g, 78 mmol, 56%). IR (neat) ν = 2977, 2956, 2929, 2859, 1523, 1436,1330, 1249, 1142, 1006, 1006, 996, 820, 775 cm^{-1}; ^{1}H NMR (600 MHz, $CDCl_3$) δ = 7.13 (s, 1 H), 2.88 (t, J = 6 Hz, 2 H), 1.58-1.56 (m, 2 H), 1.34-1.32 (m, 2 H), 1.32 (s, 12H) 0.93 (t, J = 7 Hz, 3 H), 0.30 (s, 9 H). ^{13}C NMR (100 MHz, $CDCl_3$) δ = 155.5, 147.1, 137.17, 83.5, 34.1, 29.6, 24.8, 22.5 13.92, 0.01. MS (ESI) m/z $[M+H]^+$ = 339.1977. Elemental analysis calculated for $C_{17}H_{31}BO_2SSi$: Calculated: C, 60.34; H, 9.23; B, 3.19; O, 9.46; S, 9.48; Si, 8.30; found: C, 60.20; H, 9.42; S, 9.29

S2.1.5 Synthesis of (4-hexyl-5-(4,4,5,5-tetramethyl-1,3,2-dioxaborolan-2-yl)thiophen-2-yl)trimethylsilane (3b)

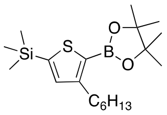

A solution of *n*-BuLi (160 mL, 2.5 M, 400 mmol) was added drop wise over 1 hour to a solution of (4-hexylthiophen-2-yl)trimethylsilane (86 g, 358 mmol) in 250 mL of THF at −78 °C. The mixture was allowed to react for 1 hour then 2-isopropoxy-4,4,5,5-tetramethyl-1,3,2-

dioxaborolane (74.4 g, 81.6 mL, 400 mmol) was slowly added. The reaction mixture was allowed to warm to rt and stirred for 1 hour. The reaction was quenched by addition of 250 mL of water. The THF was removed under vacuum (RotoVap), 200 mL of diethyl ether added and the organic phases separated, washed with water (2 × 100 mL) then dried over $MgSO_4$. The slurry was filtered to remove the $MgSO_4$, the solvent removed under vacuum and the crude product distilled. The unreacted (4-hexylthiophen-2-yl)trimethylsilane was removed and the product then collected which included some 2,5-bis-trimethylsilyl-3-hexylthiophene which could be carried forward without impacting the next reaction. Yield 88.5 g (62%) Analytically pure material could be isolated by careful distillation at 132–135 °C at 440-445 mTorr. ^1H NMR (400 MHz, $CDCl_3$) δ = 7.134 (s, 1 H), 2.876 (t, J = 7.8 Hz, 2 H), 1.65-1.55 (m, 2 H), 1.36-1.28 (m, 18 H), 0.892 (t, J = 7.8 Hz, 3 H), 0.303 (s, 9 H); ^{13}C NMR (100 MHz, $CDCl_3$) δ = 155.6, 147.1, 137.2, 83.459, 31.9, 31.6, 29.9, 29.1, 24.8, 22.6, 14.1, -0.12; MS (ESI) m/z $[M+H]^+$ = 367.2294^+. Elemental Analysis calculated for $C_{19}H_{35}BO_2SSi$: Calculated C, 62.28; H, 9.63; S, 8.75. Found C, 62.47; H, 9.86; S, 8.80.

S2.1.6 Synthesis of (4-octyl-5-(4,4,5,5-tetramethyl-1,3,2-dioxaborolan-2-yl)thiophen-2-yl)trimethylsilane (3c)

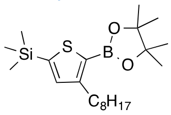

A solution of *n*-BuLi (76 mL, 2.5 M, 190 mmol) was added drop wise over 1 hour to a solution of (4-octylthiophen-2-yl)trimethylsilane (45 g, 167.6 mmol) in THF (125 mL) at −78 °C. The mixture was allowed to react for 1 hour then 2-isopropoxy-4,4,5,5-tetramethyl-1,3,2-dioxaborolane (35.4 g, 190 mmol) was slowly added. The reaction mixture was allowed to warm to rt and stirred for 1 hour. The reaction was quenched by addition of 250 mL of water. The THF was removed under rotary evaporation, diethyl ether (200 mL) was added and the organic phases separated, washed with water (2 × 100 mL) then dried over $MgSO_4$. The slurry was filtered to remove the $MgSO_4$, the solvent removed under vacuum and the crude product distilled at 150 °C at 730 mTorr to yield a clear, viscous oil (38.08 g, 96.59 mmol, 58%). IR (neat) ν = 2956, 2924, 2855, 1330, 1249, 1142, 996, 820 cm^{-1}; ^1H NMR (600 MHz, $CDCl_3$) δ = 7.13 (s, 1 H), 2.87 (t, J = 6 Hz, 2 H), 1.61-1.57 (m, 2 H), 1.32 (s, 12 H), 1.29-1.23 (m, 10H) 0.89 (t, J = 7 Hz, 3 H), 0.30 (s, 9 H). ^{13}C NMR (100 MHz, $CDCl_3$) δ = 155.6, 147.1, 137.18, 83.5, 31.9, 30.0, 29.5, 29.4, 29.3, 24.8, 14.1, -0.01. MS (ESI) m/z $[M+Ag(I)]^+$ = 501.1575. Elemental analysis calculated for $C_{21}H_{38}SSiBO_2$: Calculated: C, 63.94; H, 9.96; B, 2.74; O, 8.11; S, 8.13; Si, 7.12, found: C: 64.21 H: 9.98.

S2.2 Synthesis of oligothiophenes 4-11

S2.2.1 Synthesis of 3'-butyl-5'-(trimethylsilyl)-[2,2'-bithiophene]-5-carbaldehyde (4a)

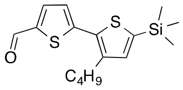

A mixture of **3a** (20 g, 59.14 mmol), 5-bromothiophen-2-ylcarboxaldehyde (11.3 g, 59.14 mmol), THF (100 mL) and 2.0 M K_3PO_4 (89 mL) was degassed by bubbling dinitrogen through for 30 minutes. The catalyst, $Pd_2(dba)_3$ (680 mg, 0.74 mmol) and P(*o*-tol)$_3$ (900 mg, 2.95 mmol) was added and the reaction mixture was stirred at 60 °C for 18 h. The reaction mixture was filtered through celite filter aid and the THF removed under vacuum. Diethyl ether (100 mL) was added and the organic phase separated, washed with water (2 × 100 mL) and dried over $MgSO_4$ and filtered. After removing the solvent the crude product was purified twice by silica gel column

chromatography (petroleum spirit: dichloromethane = 1:1.5, then petroleum spirits:ethyl acetate = 5:1) to give the pure product as a brown oil (9.06 g, 28.12 mmol, 48%).

*From **2a** by direct arylation*

A mixture of **2a** (300 mg, 1.41 mmol), $Pd(OAc)_2$ (5.8 mg, 0.028 mmol), diphenylphosphinobutane (11.9 mg, 0.028 mmol) and KOAc (137 mg, 1.4 mmol) were degassed under dinitrogen for 30 minutes. A solution of 5-bromothiophen-2-ylcarboxaldehyde (534 mg, 2.8 mmol) in DMAc (5 mL) was stirred, degassed under dinitrogen for 30 minutes and introduced to the other reagents. The reaction mixture was then stirred for 5 hours at 120 °C. The reaction mixture was filtered through celite filter aid and the solvent removed under vacuum. Diethyl ether (20 mL) was added and the organic phase separated, washed with water (2 × 20 mL) and dried over $MgSO_4$ and filtered. After removing the solvent the crude product was purified twice by silica gel column chromatography (petroleum spirit: dichloromethane = 1:1.5, then petroleum spirit:ethyl acetate = 5:1) to give the pure product as a brown oil (0.15 g, 0.45 mmol, 32%).IR (neat) ν = 2955, 2930, 1664, 1510, 1446, 1417, 1249, 1224, 1011, 820, 754 cm^{-1}; ^{1}H NMR (600 MHz, $CDCl_3$) δ = 9.88 (s, 1 H), 7.70 (d, J = 3.9 Hz, 1 H), 7.23 (d, J = 3.9 Hz, 1 H), 7.07 (s, 1 H), 2.81 (t, J = 7.9 Hz, 2 H), 1.66-1.64 (m, 2 H), 1.44-1.40 (m, 2H), 0.95 (t, J = 7.08 Hz, 3 H), 0.33 (s, 9H). ^{13}C NMR (100 MHz, $CDCl_3$) δ = 182.7, 146.8, 143.1, 142.2, 141.5, 137.5, 136.8, 134.5, 126.0, 32.7, 29.2, 22.7, 13.9, -0.22. MS (ESI) m/z $[M+H]^+$ = 323.0953. Elemental analysis calculated for $C_{16}H_{22}OS_2Si$: Calculated: C, 59.58; H, 6.87; O, 4.96; S, 19.88; Si, 8.71; found: C, 59.79; H, 6.95; S, 19.92

S2.2.2 Synthesis of 3'-hexyl-5'-(trimethylsilyl)-[2,2'-bithiophene]-5-carbaldehyde (4b)

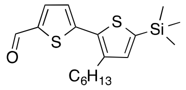

A 500 mL flask containing compound **3b** (20.0 g, 54.6 mmol), 5-bromothiophen-2-ylcarboxaldehyde (10.4 g, 54.6 mmol), 100 mL of THF and 75mL of 2.0 M K_3PO_4 was degassed by bubbling dinitrogen through for 30 minutes. The catalyst, $Pd_2(dba)_3$ (626 mg, 0.68 mmol) and P(*o*-tol)$_3$ (830 mg, 2.73 mmol) was added and the reaction mixture was stirred at 60 °C for 12 h. The reaction mixture was filtered through celite filter aid and the THF removed under vacuum. Diethyl ether (200 mL) was added and the organic phase separated, washed with water (2 × 100 mL) and dried over $MgSO_4$ and filtered. After removing the solvent the crude product was purified by silica gel column chromatography (petroleum spirit : dichloromethane = 1:1) to give the crude product as a pale yellow liquid. Unreacted 4 was removed under high vacuum and the product purified by silica gel column chromatography (petroleum spirit : ethyl acetate = 9:1) to give the product as a pale yellow liquid. R_f 0.65 (dichloromethane/petroleum spirits 1:1); IR (neat cm^{-1}) 2954, 2928, 2856, 1651, 1445, 1416, 1249, 1223, 1053, 1001, 835, 801, 753, 667; ^{1}H NMR (400 MHz, $CDCl_3$) δ = 9.872 (s, 1 H), 7.690 (d, J = 4.0 Hz, 1 H), 7.218 (d, J = 4.0 Hz, 1 H), 7.074 (s, 1 H), 2.804 (t, J = 8.0 Hz, 2 H), 1.60-1.75 (m, 2 H), 1.36-1.45 (m, 2 H), 1.35-1.28 (m, 4 H), 0.893 (t, J = 9.0 Hz, 3 H), 0.335 (s, 9 H); ^{13}C NMR (100 MHz, $CDCl_3$) δ = 182.6, 146.7, 143.1, 142.2, 141.5, 137.5, 136.8, 134.5, 126.0, 31.6, 30.5, 29.5, 29.3, 22.6, 14.1, -0.21; MS (ESI) m/z $[M+H]^+$ = 351.1270. Elemental Analysis calculated for: C, 61.66; H, 7.47; S, 18.29. Found C, 61.89; H, 7.62; S, 18.35.

S2.2.3 Synthesis of 3'-octyl-5'-(trimethylsilyl)-[2,2'-bithiophene]-5-carbaldehyde (4c)

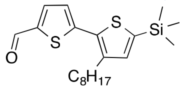

A mixture of **3c** (35 g, 88.2 mmol), 5-bromothiophen-2-ylcarboxaldehyde (16.8 g, 88.2 mmol), THF (350 mL) and 2.0 M K_3PO_4 (122 mL) was degassed by bubbling dinitrogen through for 30 minutes. The catalyst, $Pd_2(dba)_3$ (1 g, 1.12 mmol) and P(*o*-tol)$_3$ (1.35 g, 4.2 mmol) was added and the reaction mixture was stirred at 60 ^oC for 18 h. The reaction mixture was filtered through celite filter aid and the THF removed under vacuum. Diethyl ether (300 mL) was added and the organic phase separated, washed with water (2 × 150 mL) and dried over $MgSO_4$ and filtered. After removing the solvent the crude product was purified twice by silica gel column chromatography (petroleum spirit: dichloromethane = 1:1, then petroleum spirit:ethyl acetate = 10:1) to give the pure product as a brown oil (26.34 g, 69.68 mmol, 79%).

From ***2c*** *by direct arylation*

A mixture of **2c** (379 mg, 1.41 mmol), $Pd(OAc)_2$ (5.8 mg, 0.028 mmol), bis-(diphenylphosphino)butane (11.9 mg, 0.028 mmol) and KOAc (137 mg, 1.4 mmol) were degassed under dinitrogen for 30 minutes. A solution of 5-bromothiophen-2-ylcarboxaldehyde (534 mg, 2.8 mmol) in dimethylacetamide (DMAc) (5 mL) was stirred, degassed under dinitrogen for 30 minutes and introduced to the other reagents. The reaction mixture was then stirred for 5 hours at 120 °C. The reaction mixture was filtered through celite filter aid and the solvent removed under vacuum. Diethyl ether (20 mL) was added and the organic phase separated, washed with water (2 × 20 mL) and dried over $MgSO_4$ and filtered. After removing the solvent the crude product was purified twice by silica gel column chromatography (petroleum spirit: dichloromethane = 1:1.5, then petroleum spirit:ethyl acetate = 5:1) to give the pure product as a brown oil (0.18 g, 0.47 mmol, 34%).

IR (neat) ν = 2954, 2925, 2855, 1668, 1446, 1250, 1225, 820 cm^{-1}; ^1H NMR (600 MHz, $CDCl_3$) δ = 9.88 (s, 1 H), 7.69 (d, J = 3.9 Hz, 1 H), 7.23 (d, J = 3.9 Hz, 1 H), 7.07 (s, 1 H), 2.80 (t, J = 7.9 Hz, 2 H), 1.69-1.56 (m, 2 H), 1.38-1.34 (m, 2H), 1.34-1.26 (m, 8H) 0.875 (t, J = 7.08 Hz, 3 H), 0.33 (s, 9H). ^{13}C NMR (100 MHz, $CDCl_3$) δ = 182.7, 146.8, 143.1, 142.2, 141.5, 137.5, 136.8, 134.5, 126.0, 31.8, 30.5, 29.6, 29.5, 29.4, 29.2, 22.7, 14.1, -0.22. MS (ESI) m/z $[M+H]^+$ = 379.1586. Elemental analysis calculated for $C_{20}H_{30}SiOS_2$: Calculated: C, 63.44; H, 7.99; S, 16.94. Found: C, 63.68; H, 8.03; S, 16.71

S2.2.4 Synthesis of 3'-butyl-5'-iodo-[2,2'-bithiophene]-5-carbaldehyde (5a)

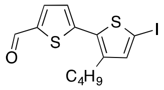

A solution of ICl (3.8 g, 23.3 mmol) in DCM (25 mL) was added slowly to a solution of **4a** (7.5 g, 23.3 mmol) in DCM (100 mL) under dinitrogen at −15 °C (ice/acetone). The solution was allowed to react for one hour then quenched by addition of 2 M K_2CO_3 then a 1 M solution of Na_2SO_3. The biphasic reaction mix was filtered through celite, and then the organic phase separated, washed with water (2 × 100 mL) and then dried over $MgSO_4$. The solvent was removed from the filtered solution and a crude product obtained by silica gel column chromatography (petroleum spirit: dichloromethane = 1:1) as a pale yellow liquid. The pure product was obtained by silica gel column chromatography (petroleum spirit : ethyl acetate = 7:3) to give the product as a brown oil which crystallized as yellow, needle-like crystals under vacuum (7.4 g, 19.67 mmol,

84%). IR (neat) ν = 2956, 2926, 1666, 1443, 1223, 1057, 801 cm^{-1}; ^{1}H NMR (600 MHz, $CDCl_3$) δ = 9.88 (s, 1 H), 7.69 (d, J = 3.95 Hz, 1 H), 7.14 (d, J = 3.27 Hz, 1 H), 7.12 (s, 1 H), 2.75 (t, J = 7.8 Hz, 2 H), 1.64-1.59 (m, 2 H), 1.38-1.33 (m, 2H), 1.31-1.25 (m, 8H) 0.88 (t, J = 7.08 Hz, 3 H). ^{13}C NMR (100 MHz, $CDCl_3$) δ = 182.6, 144.8, 143.6, 142.7, 140.4, 136.6, 135.5, 126.5, 74.5, 32.5, 28.9, 22.5, 13.9. MS (ESI) m/z $[M+H]^+$ = 376.9526. Elemental analysis calculated for $C_{13}H_{13}OS_2I$: Calculated: C, 41.50; H, 3.48; I, 33.73; O, 4.25; S, 17.04; found: C, 41.42; H, 3.53; S, 17.28

S2.2.5 Synthesis of 3'-hexyl-5'-iodo-[2,2'-bithiophene]-5-carbaldehyde (5b)

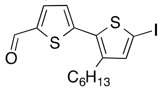

A solution of ICl (6.1 g, 37.4 mmol) in 50 mL of DCM was added slowly to a solution of **4b** (13.1 g, 37.4 mmol) in DCM (250 mL) under dinitrogen at −15 °C (ice/acetone). The solution was allowed to react for one hour then quenched by addition of 2 M K_2CO_3 then a 1 M solution of Na_2SO_3. The biphasic reaction mix was filtered through celite, and then the organic phase separated, washed with water (2 × 100 mL) and then dried over $MgSO_4$. The solvent was removed from the filtered solution and a crude product obtained by silica gel column chromatography (petroleum spirit : dichloromethane = 1:1) as a pale yellow liquid. The pure product was obtained by silica gel column chromatography (petroleum spirit : ethyl acetate = 9:1) to give the product as a pale yellow liquid. Yield 13.62 g (33.7 mmol, 90%). R_f 0.57 (dichloromethane/petroleum spirit 1:1); IR (neat cm^{-1}) 2953, 2924, 2804, 1659, 1438, 1219, 1054, 798, 753, 665; ^{1}H NMR (400 MHz, $CDCl_3$) δ = 9.876 (s, 1 H), 7.680 (d, J = 4.0 Hz, 1 H), 7.136 (d, J = 4.0 Hz, 1 H), 7.104 (s, 1 H), 2.744 (t, J = 7.8 Hz, 2 H), 1.68-1.55 (m, 4 H), 1.39-1.25 (m, 6H), 0.875 (t, J = 7.0 Hz, 6 H); ^{13}C NMR (100 MHz, $CDCl_3$) δ = 182.6, 144.8, 143.6, 142.7, 140.3, 136.6, 135.5, 126.5, 74.5, 31.6, 30.3, 29.2, 29.1, 22.5, 14.1; MS (ESI) m/z $[M+H]^+$ = 404.9839. Elemental Analysis calculated for $C_{15}H_{17}IOS_2$: Calculated C, 44.56; H, 4.24; I, 31.39; O, 3.96; S, 15.86. Found C, 44.75; H, 4.31; S, 15.92.

S2.2.6 Synthesis of 3'-octyl-5'-iodo-[2,2'-bithiophene]-5-carbaldehyde (5c)

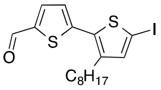

A solution of ICl (6.47 g, 39.67 mmol) in DCM (50 mL) was added slowly to a solution of **4c** (15 g, 39.67 mmol) in DCM (250 mL) under dinitrogen at −15 °C (ice/acetone). The solution was allowed to react for one hour then quenched by addition of 2 M K_2CO_3 then a 1 M solution of Na_2SO_3. The biphasic reaction mix was filtered through celite, and then the organic phase separated, washed with water (2 × 100 mL) and then dried over $MgSO_4$. The solvent was removed from the filtered solution and a crude product obtained by silica gel column chromatography (petroleum spirit: dichloromethane = 1:1) as a pale yellow liquid. The pure product was obtained by silica gel column chromatography (petroleum spirit : ethyl acetate = 9:1) to give the product as yellow, needle-like crystals (14.05 g, 32.53 mmol, 82%). IR (neat) ν = 2924, 2853, 1668, 1443, 1222 cm^{-1}; ^{1}H NMR (600 MHz, $CDCl_3$) δ = 9.88 (s, 1 H), 7.69 (d, J = 3.95 Hz, 1 H), 7.14 (d, J = 3.27 Hz, 1 H), 7.12 (s, 1 H), 2.75 (t, J = 7.8 Hz, 2 H), 1.64-1.59 (m, 2 H), 1.38-1.33 (m, 2H), 1.31-1.25 (m, 8H) 0.88 (t, J = 7.08 Hz, 3 H). ^{13}C NMR (100 MHz, $CDCl_3$) δ = 182.6, 144.8, 143.7, 142.7, 140.3, 136.6, 135.5, 126.5, 74.5, 31.8, 30.3, 29.4, 29.3, 29.2, 29.1, 22.6, 14.1. MS (ESI) m/z

$[M+H]^+$ = 433.0137. Elemental analysis calculated for $C_{17}H_{21}IOS_2$: Calculated: C, 47.22; H, 4.90; I, 29.35; O, 3.70; S, 14.83; found: C: 46.92, H: 4.91.

S2.2.7 Synthesis of 3',3''-dibutyl-5''-(trimethylsilyl)-[2,2':5',2''-terthiophene]-5-carbaldehyde (6a)

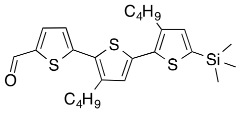

A mixture of **3a** (5.7 g, 15.32 mmol), **5a** (5.8 g, 15.32mmol), THF (100 mL) and 2.0 M K_3PO_4 (35 mL) was degassed by bubbling dinitrogen through for 30 minutes. The catalyst, $Pd_2(dba)_3$ (340 mg, 0.38 mmol) and P(*o*-tol)$_3$ (466 mg, 1.53 mmol) was added and the reaction mixture was stirred at 60 °C for 18 h. The reaction mixture was filtered through celite filter aid and the THF removed under vacuum. Diethyl ether (100 mL) was added and the organic phase separated, washed with water (2 × 50 mL) and dried over $MgSO_4$ and filtered. After removing the solvent the crude product was purified twice by silica gel column chromatography (petroleum spirit: dichloromethane = 1:1, then petroleum spirit: ethyl acetate = 7:3) to give the pure product as a brown oil which crystallized into yellow needles under high pressure (5.6 g, 12 mmol, 80%). IR (neat) ν = 2955, 2924, 1666, 1449, 1453, 1251, 1221, 1057, 1005, 837, 827 cm^{-1}; ^1H NMR (600 MHz, $CDCl_3$) δ = 9.88 (s, 1 H), 7.70 (d, J = 4.0, 1 H), 7.23 (d, J = 4.0 Hz, 1 H), 7.04 (s, 1H), 6.99 (s, 1 H), 2.82 (t, J = 7.8 Hz, 2 H), 2.78 (t, J = 7.8 Hz, 2 H), 1.71-1.62 (m, 4 H), 1.46-1.41 (m, 4 H), 0.96 (m, 6 H), 0.33 (s, 9H). ^{13}C NMR (100 MHz, $CDCl_3$) δ = 182.6, 146.4, 142.4, 142.0, 141.4, 139.5, 137.3, 136.9, 136.7, 135.0, 129.1, 128.9, 125.7, 32.8, 32.4, 29.5, 29.0 22.8, 22.6, 13.9, -0.16. MS (ESI) m/z $[M+H]^+$ = 461.1456. Elemental analysis calculated for $C_{24}H_{32}OSiS_3$: Calculated: C, 62.56; H, 7.00; O, 3.47; S, 20.88; Si, 6.10; found: C, 62.48, H, 70.12

S2.2.8 Synthesis of 3',3''-dihexyl-5''-(trimethylsilyl)-[2,2':5',2''-terthiophene]-5-carbaldehyde (6b)

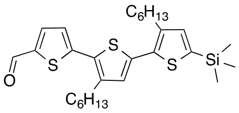

The synthesis of **6b** was completed by reacting **5b** (13.62 g, 33.7 mmol), with **3b** (12.34 g, 33.7 mmol) in 250 mL THF and 75 mL of 2 M K_3PO_4 under palladium catalysis {Pd_2dba_3 (770 mg, 0.84 mmol), P(*o*-tolyl)$_3$ (1.03 g, 3.37 mmol)}. The reaction was stirred at 60 °C for 18 hours. The reaction mixture was filtered through celite and the THF removed under vacuum. Diethyl ether (200 mL) was added and the organic phase separated, washed with water (2 × 100 mL) and dried over $MgSO_4$ and filtered. After removing the solvent the crude product was purified by silica gel column chromatography (petroleum spirit : dichloromethane = 1:1) to give the crude product as a pale yellow liquid. The crude product was further purified by silica gel column chromatography (petroleum spirit : ethyl acetate = 9:1) to give the product as a pale yellow liquid. Yield 13.9 g (26.9 mmol, 79.8%). R_f 0.65 (dichloromethane/petroleum spirit 1:1); IR (neat cm^{-1}) 2955, 2926, 2856, 1663, 1663, 1433, 1249, 1224, 1056, 1000, 835, 798, 754, 667; ^1H NMR (400 MHz, $CDCl_3$) δ = 9.880 (d, J = 0.2 Hz, 1 H), 7.701 (dd, J = 4.0 & 0.5 Hz, 1 H), 7.055 (dd, J = 4.0 & 0.5 Hz, 1 H), 6.994 (s, 1 H), 2.819 (t, J = 7.8 Hz, 1 H), 2.779 (t, J = 7.8 Hz, 1 H), 1.692-1.644 (m, 4 H), 1.42-1.28 (m, 12 H), 0.897 (brt, J = 7.0 Hz, 6 H), 0.332(s, 9H); ^{13}C NMR (100 MHz, $CDCl_3$) δ = 182.5,

146.4, 142.5, 142.0, 141.5, 139.5, 137.3, 136.9, 136.7, 134.9, 129.1, 128.9, 125.7, 31.65, 31.63, 30.7, 30.2, 29.8, 29.3, 29.2, 22.6, 22.6, 14.08, 14.06, -0.1; MS (ESI) m/z $[M+H]^+$ = 517.2090. Elemental Analysis calculated for $C_{28}H_{40}OS_3Si$: Calculated C, 65.06; H, 7.80; S, 18.61. Found C, 65.12; H, 7.85; S, 18.84.

S2.2.9 Synthesis of 3',3''-dioctyl-5''-(trimethylsilyl)-[2,2':5',2''-terthiophene]-5-carbaldehyde (6c)

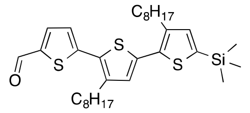

A mixture of **3c** (7.3 g, 18.51 mmol), **5c** (8 g, 18.51 mmol), THF (125 mL) and 2.0 M K_3PO_4 (42 mL) was degassed by bubbling dinitrogen through for 30 minutes. The catalyst, $Pd_2(dba)_3$ (421 mg, 0.46 mmol) and P(*o*-tol)$_3$ (570 mg, 1.85 mmol) was added and the reaction mixture was stirred at 60 °C for 18 h. The reaction mixture was filtered through celite filter aid and the THF removed under vacuum. Diethyl ether (200 mL) was added and the organic phase separated, washed with water (2 × 50 mL) and dried over $MgSO_4$ and filtered. After removing the solvent the crude product was purified twice by silica gel column chromatography (petroleum spirit: dichloromethane = 1:1, then petroleum spirit: ethyl acetate = 10:1) to give the pure product as a brown oil (9.1 g, 15.91 mmol, 86%). IR (neat) ν = 2954, 2924, 2854, 1665, 1444, 1249, 1225, 1003, 820 cm^{-1}; ^1H NMR (600 MHz, $CDCl_3$) δ = 9.88 (s, 1 H), 7.70 (d, J = 4.0, 1 H), 7.23 (d, J = 4.0 Hz, 1 H), 7.05 (s, 1H), 6.99 (s, 1 H), 2.82 (t, J = 7.8 Hz, 2 H), 2.78 (t, J = 7.8 Hz, 2 H), 1.71-1.63 (m, 4 H), 1.42-1.36 (m, 4 H), 1.36-1.23 (m, 16H), 0.88 (dd, J = 7.0 Hz, 2.3 Hz, 6 H), 0.33 (s, 9H). ^{13}C NMR (100 MHz, $CDCl_3$) δ = 182.5, 146.4, 142.5, 142.0, 141.5, 139.5, 137.3, 136.9, 136.7, 135.0, 129.1, 128.9, 125.7, 31.9, 31.8, 30.7, 30.2, 29.8, 29.6, 29.5, 29.4, 29.3, 29.2, 22.6, 14.09, -0.1. MS (ESI) m/z $[M+H]^+$ = 573.2683. Elemental analysis calculated for $C_{32}H_{48}SiOS_3$: Calculated: C, 67.08; H, 8.44; O, 2.79; S, 16.79; Si, 4.90; found: C, 67.12; H, 8.58; S, 16.54

S2.2.10 Synthesis of 3',3''-dibutyl-5''-iodo-[2,2':5',2''-terthiophene]-5-carbaldehyde (7a)

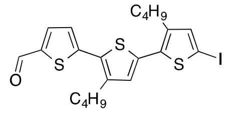

A solution of ICl (1.7 g, 10.5 mmol) in DCM (20 mL) was added slowly to a solution of **6a** (4.8 g, 10.5 mmol) in DCM (100 mL) under dinitrogen at −15 °C (ice/acetone). The solution was allowed to react for one hour then quenched by addition of 2 M K_2CO_3 then a 1 M solution of Na_2SO_3. The biphasic reaction mix was filtered through celite, and then the organic phase separated, washed with water (2 × 100 mL) and then dried over $MgSO_4$. The solvent was removed from the filtered solution and purified twice by silica gel column chromatography (petroleum spirit: dichloromethane = 1:1, then petroleum spirit: ethyl acetate = 4:1) to give the mixed iodinated/deiodinated terthiophene as yellow needles. This was used without further purification.

An alternative iodination reaction is as follows:

A mixture of **6a** (0.5 g, 1.08 mmol), n-iodosuccinimide (0.36 g, 1.63 mmol) and sodium acetate (0.5 g, 3 mmol) was dissolved in a solution of chloroform (15 mL) and acetic acid (15 mL), then cooled to 0 °C. The mixture was stirred for 1 hour, then poured into H_2O (50 mL). The mixture was then extracted with DCM (2 × 50 mL) and the combined organic phase was washed with saturated $NaHCO_3$ (2 × 50 mL) before being dried over $MgSO_4$. The solvent was removed from the filtered solution and purified twice by silica gel column chromatography (petroleum spirit:

dichloromethane = 1:1, then petroleum spirit: ethyl acetate = 4:1) to give the product as yellow needles (0.43 g, 0.83 mmol, 77%). IR (neat) ν = 2925, 2854, 1666, 1456, 1434, 1260, 1225, 764 cm^{-1}; ^1H NMR (600 MHz, $CDCl_3$) δ = 9.88 (s, 1 H), 7.71 (d, J = 4.0, 1 H), 7.23 (d, J = 4.0 Hz, 1 H), 7.08 (s, 1H), 6.92 (s, 1 H),), 2.78 (t, J = 7.8 Hz, 2 H), 1.71-1.62 (m, 4 H), 1.46-1.41 (m, 4 H), 0.96 (m, 6 H). ^{13}C NMR (100 MHz, $CDCl_3$) δ = 182.6, 145.9, 142.4, 142.3, 142.0, 140.0, 136.8, 135.8, 135.0, 129.7, 129.5, 126.0, 72.4, 32.7, 32.4, 29.4, 28.7, 22.6, 22.5, 13.9, 13.9.MS (ESI) m/z $[M+H]^+$ = 515.0028Elemental analysis calculated for $C_{21}H_{23}OIS_3$: Calculated: C, 49.02; H, 4.51; I, 24.67; O, 3.11; S, 18.70; found: C, 49.06; H, 4.77; S, 18.54

S2.2.11 Synthesis of 3',3''-dihexyl-5''-iodo-[2,2':5',2''-terthiophene]-5-carbaldehyde (7b)

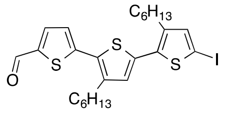

A solution of ICl (4.32 g, 26.6 mmol) in 50 mL of DCM was added slowly to a solution of **6b** 13.9 g, 26.59 mmol) in DCM (250 mL) under dinitrogen at −15 °C (ice/acetone). The solution was allowed to react for one hour then quenched by addition of 2 M K_2CO_3 then of a 1 M solution of Na_2SO_3. The biphasic reaction mix was filtered through celite, and then the organic phase separated, washed with water (2 × 100 mL) and then dried over $MgSO_4$. The solvent was removed from the filtered solution and a crude product obtained by silica gel column chromatography (petroleum spirit : dichloromethane = 1:1) as a pale yellow liquid. The pure product was obtained by silica gel column chromatography (petroleum spirit : ethyl acetate = 9:1) to give the product as a pale yellow liquid. Yield 14.0 g (24.55 mmol, 92%). R_f 0.63 (dichloromethane/petroleum spirit 1:1); IR (neat) ν = CO 1659 cm^{-1}; ^1H NMR (400 MHz, $CDCl_3$) δ = 9.869 (s, 1 H), 7.691 (d, J = 3.9 Hz, 1 H), 7.206 (d, J = 3.9 Hz, 1 H), 7.063 (s, 1 H), 6.900 (s, 1 H), 2.786 (t, J = 7.8 Hz, 2 H), 2.708 (t, J = 7.8 Hz, 2 H), 1.68-1.57 (m, 4 H), 1.38-1.26 (m, 12 H), 0.867 (m, 6 H); ^{13}C NMR (100 MHz, $CDCl_3$) δ = 182.5, 142.3, 142.1, 139.9, 136.8, 135.8, 134.9, 129.4, 126.0, 31.59, 31.56, 30.5, 30.2, 29.7, 29.2, 29.1, 29.0, 22.5, 14.0; MS (ESI): m/z calculated for $C_{25}H_{31}IOS_3$ 571.06545; found 571.06539 $[M+H^+]$. Micro calculated for $C_{25}H_{31}IOS_3$ C, 52.62; H, 5.48; S, 16.86. Found C, 52.77; H, 5.68; S, 16.60.

S2.2.12 Synthesis of 3',3''-dioctyl-5''-iodo-[2,2':5',2''-terthiophene]-5-carbaldehyde (7c)

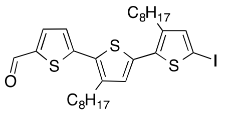

A solution of ICl (1.7 g, 10.5 mmol) in DCM (20 mL) was added slowly to a solution of **6c** (6 g, 10.5 mmol) in DCM (100 mL) under dinitrogen at −15 °C (ice/acetone). The solution was allowed to react for one hour then quenched by addition of 2 M K_2CO_3 then a 1 M solution of Na_2SO_3. The biphasic reaction mix was filtered through celite, and then the organic phase separated, washed with water (2 × 100 mL) and then dried over $MgSO_4$. The solvent was removed from the filtered solution and purified twice by silica gel column chromatography (petroleum spirit: dichloromethane = 1:1, then petroleum spirit: ethyl acetate = 10:1) to give the pure product as a brown, viscous oil (5.4 g, 8.61 mmol, 82%). IR (neat) ν = 2925, 2854, 1666, 1456, 1434, 1260, 1225, 764 cm^{-1}; ^1H NMR (600 MHz, $CDCl_3$) δ = 9.88 (s, 1 H), 7.71 (d, J = 4.0, 1 H), 7.23 (d, J =

4.0 Hz, 1 H), 7.08 (s, 1H), 6.92 (s, 1 H), 2.82 (t, *J* = 7.8 Hz, 2 H), 2.78 (t, *J* = 7.8 Hz, 2 H), 1.71-1.63 (m, 4 H), 1.42-1.36 (m, 4 H), 1.36-1.23 (m, 16H), 0.88 (dd, *J* = 7.0 Hz, 2.3 Hz, 6 H). ^{13}C NMR (400MHz, $CDCl_3$) δ =182.55, 145.89, 142.40, 142.32, 142.07, 139.94, 136.77, 135.81, 134.96, 129.74, 129.45, 126.00, 72.36, 31.86, 31.84, 30.53, 30.25, 29.69, 29.52, 29.42, 29.40, 29.35, 29.22, 28.98, 22.66, 14.10. MS (ESI) m/z $[M+H]^+$ = 627.1286. Elemental analysis calculated for $C_{29}H_{39}IOS_3$: Calculated: C, 55.58; H, 6.27; S, 15.35; Found: C, 57.60; H, 6.64; S, 14.64

S2.2.13 Synthesis of 3',3'',3'''-trihexyl-5'''-(trimethylsilyl)-[2,2':5',2'':5'',2'''-quaterthiophene]-5-carbaldehyde (8b)

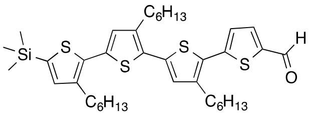

The synthesis of **8b** was completed by reacting **7b** (5.67 g, 10.0 mmol), with **3b** (3.66 g, 10.0 mmol) in 100 mL THF and 50 mL of 2 M K_3PO_4 under palladium catalysis {Pd_2dba_3 (228 mg, 0.25 mmol), P(*o*-tolyl)$_3$ (300 mg, 1.00 mmol)}. The reaction mixture was filtered through celite filter aid and the THF removed under vacuum. Diethyl ether (200 mL) was added and the organic phase separated, washed with water (2 × 100 mL) and dried over $MgSO_4$ and filtered. After removing the solvent the crude product was purified by silica gel column chromatography (petroleum spirit : dichloromethane = 1:1) to give the crude product as a sticky yellow liquid. The product purified by silica gel column chromatography (petroleum spirit : ethyl acetate = 9:1) to give the product as a pale yellow liquid. Yield 5.06 g (7.3 mmol, 73%). R_f 0.65 (dichloromethane/petroleum spirits 1:1); IR (neat) ν = CO 1663 cm^{-1}; ^{1}H NMR (400 MHz, $CDCl_3$) δ = 9.869 (s, 1 H), 7.695 (d, *J* = 4.0 Hz, 1 H), 7.220 (d, *J* = 4.0 Hz, 1 H), 7.023 (s, 1 H), 6.979 (s, 1 H), 6.941 (s, 1 H), 2.808 (t, *J* = 7.9 Hz, 2 H), 2.78-2.874 (m, 4 H), 1.69-1.60 (m, 6 H), 1.41-1.28 (m, 28 H), 0.879 (t, *J* = 6.8 Hz, 9 H), 0.306 (s, 9 H); ^{13}C-NMR (101 MHz; $CDCl_3$) δ = 182.5, 145.9, 142.39, 142.30, 142.0, 139.9, 136.80, 135.8, 135.0, 130.3, 129.7, 129.4, 129.02, 128.2, 125.98, 125.3, 124.3, 72.4, 31.64, 31.60, 30.5, 30.2, 29.7, 29.22, 29.14, 29.03, 22.6, 14. MS (MALDI): m/z calculated for $C_{38}H_{54}OS_4Si$ 682.28995; found 683.28727 $[M+H^+]$. Micro calculated C, 52.62; H, 5.48; S, 16.86. Found C, 52.77; H, 5.68; S, 16.86.

S2.2.14 Synthesis of 3',3'',3'''-trihexyl-5'''-iodo-[2,2':5',2'':5'',2'''-quaterthiophene]-5-carbaldehyde (9b)

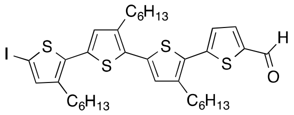

A solution of ICl (5.9 mL, 1 M in DCM, 5.9 mmol) was added slowly to a solution of **8b** (3.71 g, 5.42 mmol) in DCM (50 mL) under dinitrogen at −15 °C (ice/acetone). The solution was allowed to react for one hour then quenched by addition of a 1 M solution of Na_2SO_3. The biphasic reaction mix was filtered through celite, and then the organic phase separated, washed with water (2 × 50 mL) and then dried over $MgSO_4$. The solvent was removed from the filtered solution and a crude product obtained by silica gel column chromatography (petroleum spirit : dichloromethane = 1:1) as a red oil. The pure product was obtained by silica gel column chromatography (petroleum spirit : ethyl acetate = 9:1) to give the product as a pale yellow liquid. Yield 3.10 g (4.2 mmol, 78%). R_f 0.5 (dichloromethane/petroleum spirits 1:1); IR (neat) ν = CO 1660 cm^{-1}; ^{1}H NMR (400 MHz, $CDCl_3$) δ = 9.88 (s, 1H), 7.70 (d, *J* = 4.0 Hz, 1H), 7.23 (d, *J* = 4.0 Hz, 1H), 7.06 (s, 1H), 6.99

(s, 1H), 6.88 (s, 1H), 2.85-2.70 (m, 6H), 1.72-1.60 (m, 6H), 1.43-1.31 (m, 18H), 0.90 (brm, 9H); ^{13}C NMR (100 MHz, $CDCl_3$) δ = 182.5, 146.1, 142.5, 142.2, 141.6, 140.5, 139.9, 136.8, 136.3, 135.9, 133.3, 130.2, 129.4, 129.15, 129.00, 125.8, 71.8, 31.65, 31.61, 30.53, 30.42, 30.23, 29.8, 29.5, 29.25, 29.23, 29.13, 28.98, 22.63, 22.61, 14.11, 14.09; MS (SIMS) m/z calculated for $C_{35}H_{45}IOS_4$ 736.1398, found 736.13847 [M^+]. Elemental Analysis calculated for $C_{35}H_{45}IOS_4$: Calculated C, 57.05; H, 6.16; S, 17.41. Found C, 57.07; H, 6.23; S, 17.52.

S2.2.15 Synthesis of 3',3'',3''',3''''-tetrahexyl-5''''-(trimethylsilyl)-[2,2':5',2'':5'',2''':5''',2''''-quinquethiophene]-5-carbaldehyde (10b)

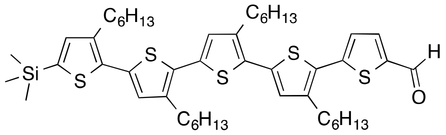

The synthesis of **10b** was completed by reacting **9b** (0.335 g, 0.45 mmol), with **3b** (0.183 g, 0.50 mmol) in 10 mL THF and 10 mL of 2 M K_3PO_4 under palladium catalysis {Pd_2dba_3 (11 mg, 0.011 mmol), *t*-$Bu_3PH.BF_4$ (14 mg, 0.045 mmol)}. The aqueous phase was removed and the solvent removed under vacuum. After removing the solvent the crude product was purified by silica gel column chromatography (petroleum spirit : dichloromethane = 1:1) to give the product as a red oil which crystallised on standing, R_f = 0.59. Yield 0.291 g (64%). IR (neat) ν = CO 1665 cm^{-1}; ^{1}H NMR (400 MHz, $CDCl_3$) δ = 9.889(s, 1H C*H*O), 7.711(d, *J* = 4.0 Hz, 1H), 7.242(d, *J* = 4.0 Hz, 1H), 7.050(s, 1H), 7.012(s, 1H), 6.977(s, 1H), 6.964(s, 1H), 2.84-2.77(m, 8H α-CH_2), 1.71-1.67(m, 8H β-CH_2), 1.44-1.34(m, 24H CH_2), 0.909(t, *J* = 7.0 Hz, 12H alkyl-CH_3), 0.335(s, 9H SiMe's); ^{13}C NMR (100 MHz, $CDCl_3$) δ =182.5, 146.2, 142.5, 142.1, 140.8, 140.6, 140.0, 138.7, 137.2, 136.8, 136.2, 135.6, 134.6, 134.5, 130.0, 129.5, 129.2, 128.8, 128.5, 125.8, 31.68, 31.65, 30.7, 30.4, 30.2, 29.8, 29.53, 29.47, 29.35, 29.30, 29.25, 29.23, 22.63, 22.60, 14.11, 14.09, 14.07, -0.1; MS (MALDI) m/z calculated for $C_{48}H_{68}OS_5Si$ 848.3643, found 848.36205 [M^+]. Elemental Analysis calculated for $C_{48}H_{68}OS_5Si$: Calculated C, 67.87; H, 8.07; S, 18.87. Found C, 67.99; H, 8.04; S, 18.99.

S2.2.16 Synthesis of 3',3'',3''',3''''-tetrahexyl-5''''-iodo-[2,2':5',2'':5'',2''': 5''',2''''-quinquethiophene]-5-carbaldehyde (11b)

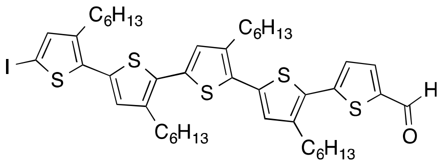

To a solution of **10b** (0.247 g, 0.291 mmol) in 50/50 $CHCl_3/CH_3COOH$ (50 mL) under dinitrogen at room temperature was added NIS (0.224 g, 1.0 mmol). The solution was allowed to react for one hour then quenched by addition of 1M K_2CO_3 and the aqueous phase removed. Excess iodine was removed by washing with 1 M solution of Na_2SO_3. The organic phase separated, washed with water (2 × 50 mL) and then dried over $MgSO_4$. The solvent was removed from the filtered solution and a pure product obtained by silica gel column chromatography (petroleum spirit : dichloromethane = 1:1) as a red oil which crystallised on standing. Yield 0.217 g (0.241 mmol, 83%). R_f 0.44 (DCM/petroleum spirits 1:1); IR (neat) ν = CO 1660 cm^{-1}; ^{1}H NMR (400 MHz, $CDCl_3$) δ = 9.89 (s, 1H), 7.71 (d, *J* = 4.0 Hz, 1H), 7.24 (d, *J* = 4.0 Hz, 1H), 7.06 (s, 1H), 7.01 (s,

1H), 6.97 (s, 1H), 6.88 (s, 1H), 2.78 (m, 8H), 1.73-1.60 (m, 8H), 1.45-1.26 (m, 24H), 0.91 (bt, *J* = 6.5 Hz, 12H); ^{13}C NMR (100 MHz, $CDCl_3$) δ = 182.5, 146.2, 142.5, 142.1, 141.4, 140.6, 140.03, 139.83, 136.8, 136.4, 136.1, 134.2, 132.8, 130.7, 129.9, 129.29, 129.12, 128.89, 128.76, 125.8, 71.6, 31.66, 31.62, 30.55, 30.44, 30.2, 29.8, 29.53, 29.41, 29.25, 29.22, 29.13, 28.97, 22.63, 22.61, 14.11, 14.08; MS (EIS) m/z calculated for $C_{45}H_{59}IOS_5$ 902.2214, found 902.22240 [M^+]. Elemental analysis calculated C, 59.84; H, 6.58; S, 17.75. Found C, 59.98; H, 6.73; S, 17.61.

S2.3 Synthesis of BTR-dialdehyde core molecule

S2.3.1 Synthesis of 2,2'-(4,8-bis(5-(2-ethylhexyl)-4-hexylthiophen-2-yl)benzo[1,2-b:4,5-b']dithiophene-2,6-diyl)bis(4,4,5,5-tetramethyl-1,3,2-dioxaborolane) (13)

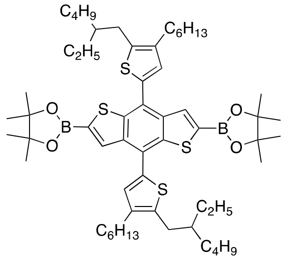

To a 50 mL Schlenk flask **11**, 4,8-bis(5-(2-ethylhexyl)-4-hexylthiophen-2-yl)benzo[1,2-*b*:4,5-*b'*]dithiophene (4.00 g, 5.36 mmol) and B_2Pin_2 (2.04 g, 8.04 mmol), and the flask heated under vacuum for 20 mins at 80 ^oC. Dry, degassed DME (20 mL) was added and set to stir at 80 °C. The following were added in the order, i) $[Ir(COD)_2OMe]_2$ (178 mg, 0.268 mmol), and ii) 4,4'-Dinonyl-2,2'-bipyridine (219 mg, 0.536 mmol) and the reaction stirred for 3 hours. The reaction vessel was cooled to rt and IPA (15 mL) added and the DME removed under vacuum. A further 20 mL of IPA were added and the product allowed to precipitate overnight. The crude product was collected by filtration, washed with IPA, dried under vacuum and used without further purification. Yield 4.86 g (4.87 mmol, 91%). ^{1}H NMR (400 MHz, $CDCl_3$) δ = 8.164 (s, 2 H), 7.181 (s, 2 H), 2.735 (dd, *J* = 7.1 & 1.9 Hz, 4 H), 2.588 (t, *J* = 7.7 Hz, 4 H), 1.63-1.59 (m, 6 H), 1.45-1.30 (m, 52 H), 0.96-0.65 (m, 18 H); ^{13}C NMR (100 MHz, $CDCl_3$) δ = 142.5, 139.3, 138.8, 138.1, 135.0, 133.8, 129.8, 124.9, 84.4, 41.6, 32.6, 32.2, 31.8, 30.8, 29.3, 28.9, 28.4, 25.8, 24.7, 23.0, 22.6, 14.2, 14.1, 10.9; MS (ESI +) m/z 998.58614 [M^+]. Elemental analysis calculated for $C_{58}H_{88}B_2O_4S_4$: C, 69.72; H, 8.88; S, 12.84. Found C, 69.74; H, 8.90; S, 12.90.

S2.3.2 Synthesis of 5,5'-(4,8-bis(5-(2-ethylhexyl)-4-hexylthiophen-2-yl)benzo[1,2-b:4,5-b']dithiophene-2,6-diyl)bis(thiophene-2-carbaldehyde)) (BM-dialdehyde)

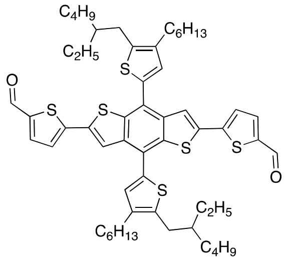

To a Schlenk flask containing **13** (0.500 g, 0.5 mmol), 5-bromo-2-thiophenecarboxaldehyde (0.48 g, 2.5 mmol), P(*o*-tolyl)$_3$ (0.00202 g, 0.001 mmol), K_3PO_4 (2M, 10 mL) and THF (20 mL) were combined in a 100 mL Schlenk tube and degassed with N_2 for 20 mins at 60 °C. To this $Pd_2(dba)_3$ (46 mg, 0.05 mmol) was added to the reaction mixture was and continued to stir at 60 °C for 20 hours under N_2. The reaction mixture was cooled to room temperature and the THF was removed under vacuum and the organic fraction was extracted with DCM and water and filtered through celite. The organic fraction was isolated and dried over $MgSO_4$ and the DCM was removed under vacuum to give the crude product. Column chromatography on silica with DCM:PET, 1:1 (R_f 0.4) was performed and the product was isolated pure and dried under high vacuum. Yield 0.176 g, (36%).%). ^{1}H-NMR (600 MHz; $Cl_2CDCDCl_2$): δ 9.88 (d, *J* = 0.7 Hz, 2H), 7.95 (s, 2H), 7.74 (d, *J* = 3.2 Hz, 2H), 7.40 (d, *J* = 3.2 Hz, 2H), 7.28 (s, 2H), 2.80 (7, *J* = 8.0 Hz, 4H), 2.65 (t, *J* = 7.5 Hz, 4H), 1.71-1.63 (m, 12H), 1.48-1.37 (m, 31H), 0.99-0.92 (m, 18H). ^{13}C-NMR (151 MHz; $Cl_2CDCDCl_2$): δ = 182.6, 146.5, 142.6, 140.1, 139.16, 139.12, 137.29, 137.12, 136.7, 133.7, 130.0, 125.9, 124.4, 122.3, 41.5, 32.5, 32.1, 31.7, 30.6, 29.1, 28.8, 28.3, 25.9, 23.0, 22.7, 14.20, 14.17, 11.0. IR (neat) ν = 2955, 2923, 2855, 1660, 1450, 1215, 1051, 793 cm^{-1}. MS (MALDI) m/z

Theoretical 966.37, Found 966.616 $[M]^{•+}$. Elemental analysis calculated for $C_{56}H_{70}O_2S_6$: Calculated C, 69.52; H, 7.29; O, 3.31; S, 19.88; Found: C, 69.60; H, 7.25; S, 19.84.

S2.3.3 Synthesis of 5',5'''-(4,8-bis(5-(2-ethylhexyl)-4-hexylthiophen-2-yl)benzo[1,2-b:4,5-b']dithiophene-2,6-diyl)bis(3'-hexyl-[2,2'-bithiophene]-5-carbaldehyde) (BB-dialdehyde)

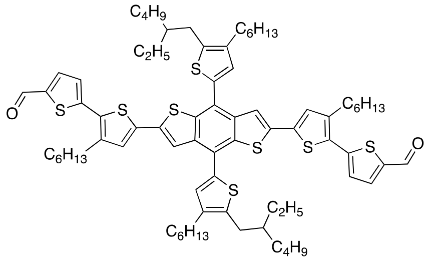

A Schlenk flask containing **13** (0.775 g, 0.5 mmol), **5b** (0.785 g, 1.94 mmol), P(*o*-tolyl)$_3$ (47 mg, 0.155 mmol), K_3PO_4 (2M, 10 mL) and THF (20 mL) were combined and degassed with N_2 for 20 mins at 60 ^oC. To this $Pd_2(dba)_3$ (35.5 mg, 0.039 mmol) was added to the reaction mixture was and continued to stir at 60 ^oC for 20 hours under N_2. The reaction mixture was cooled to room temperature and the THF was removed under vacuum and the organic fraction was extracted with DCM and water and filtered through celite. The organic fraction was isolated and dried over $MgSO_4$ and the DCM was removed under vacuum to give the crude product. Column chromatography on silica with DCM:PET, 1: (R_f 0.4) was performed and the product was isolated as a red solid. This was washed with IPA to give the pure product. Yield 0.533 g, (53%). ^{1}H-NMR (500 MHz; $CDCl_3$): δ 9.89 (s, 2H), 7.72-7.71 (m, 4H), 7.23 (m, J = 2.0 Hz, 4H), 7.15 (s, 2H), 2.82-2.77 (m, 9H), 2.65 (t, J = 7.6 Hz, 4H), 1.70-1.67 (m, 11H), 1.38-1.32 (m, 46H), 0.98 (t, J = 7.4 Hz, 7H), 0.91 (ddd, J = 9.7, 7.1, 2.6 Hz, 20H. ^{13}C-NMR (125 MHz; $CDCl_3$): δ = 182.52, 182.51, 145.9, 143.1, 142.4, 139.7, 139.1, 138.7, 137.37, 137.27, 137.0, 136.7, 134.6, 129.87, 129.77, 128.5, 126.1, 123.7, 120.1, 41.7, 32.7, 32.4, 31.9, 31.6, 30.8, 30.2, 29.9, 29.23, 29.22, 29.0, 28.4, 26.0, 23.1, 22.71, 22.57, 14.21, 14.16, 14.05, 11.0. IR (neat) ν = 2955, 2924, 2855, 1660, 1453, 1432, 1220, 1056, 821, 789 cm^{-1}. MS (MALDI) m/z Theoretical 1298.53, Found 1298.893 $[M]^{•+}$. Elemental analysis calculated for : $C_{76}H_{98}O_2S_8$: Calculated: C, 70.21; H, 7.60; S, 19.73; Found: C, 70.29; H, 7.60; S, 19.81.

S2.3.4 Synthesis of 5'',5''''-(4,8-bis(5-(2-ethylhexyl)-4-hexylthiophen-2-yl)benzo[1,2-b:4,5-b']dithiophene-2,6-diyl)bis(3',3''-dibutyl-[2,2':5',2''-terthiophene]-5-carbaldehyde) (BT^4-dialdehyde)

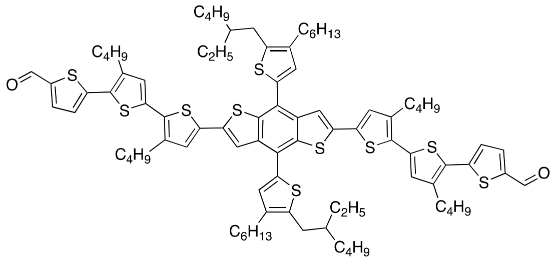

A mixture of **13** (0.5 g, 0.5 mmol), **7a** (0.62 g, 1.2 mmol), THF (30 mL) and 2.0 M K_3PO_4 (15 mL) was degassed by bubbling dinitrogen through for 30 minutes. The catalyst, $Pd_2(dba)_3$ (23 mg, 0.025 mmol) and P(*o*-tol)$_3$ (30.5 mg, 0.1 mmol) was added and the reaction mixture was stirred at 60 °C for 12 h. The reaction mixture was filtered through celite filter aid and the THF removed under vacuum. Diethyl ether (40 mL) was added and the organic phase separated, washed with water (2 × 20 mL) and dried over $MgSO_4$ and filtered. After removing the solvent the crude product was purified by silica gel column chromatography (petroleum spirit: dichloromethane = 1:1) to give the pure product as a red solid (0.51 g, 0.28 mmol, 59%). IR (neat) ν = 2957, 2926, 1646, 1434,

1264, 1230, 1058, 754, 703cm^{-1}. ^{1}H NMR (600 MHz, $CDCl_3$) δ = 9.89 (s, 2H), 7.72 (d, J = 4.0, 2H), 7.68 (s, 2H), 7.24 (d, J = 4.0, 2H), 7.23 (s, 2H), 7.12 (s, 2H), 7.02 (s, 2H), 2.80 (m, 12H), 2.65 (m, 4H), 1.69 (m, 16H), 1.47-1.37 (m, 32H), 1.00-0.88 (m, 30H). ^{13}C NMR (400MHz, $CDCl_3$) δ = 182.55, 142.47, 141.12, 139.54, 138.97, 138.58, 137.24, 136.84, 135.99, 135.74, 134.78, 129.78, 129.09, 128.31, 125.88, 123.51, 119.50, 41.69, 32.67, 32.54, 32.37, 31.84, 30.76, 29.47, 29.33, 29.19, 28.95, 28.44, 26.05, 23.09, 22.68, 22.64, 14.21, 14.15, 13.93, 11.05. MS (MALDI) calculated for $C_{88}H_{110}O_2S_{10}$ m/z = 1518.571, Found 1520.262 [M+H$^+$] Elemental analysis calculated for $C_{88}H_{110}O_2S_{10}$: Calculated: C, 69.51; H, 7.29; O, 2.10; S, 21.09; found: C, 69.90; H. 7.49; S, 20.88.

S2.3.5 Synthesis of 5'',5''''-(4,8-bis(5-(2-ethylhexyl)-4-hexylthiophen-2-yl)benzo[1,2-b:4,5-b']dithiophene-2,6-diyl)bis(3',3''-dihexyl-[2,2':5',2''-terthiophene]-5-carbaldehyde) (BT-dialdehyde)

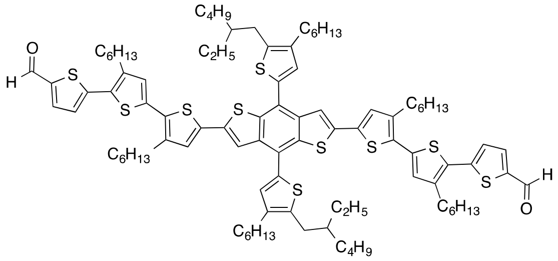

The synthesis of **BT**-dialdehyde was completed reacting **7b** (0.528 g, 0.925 mmol), with **13** (0.462 g, 0.46 mmol) in 20 mL THF and 10 mL of 2 M K_3PO_4 under palladium catalysis {Pd_2dba_3 (21.2 mg, 0.023 mmol), P(*o*-tolyl)$_3$ (28.1 mg, 0.092 mmol)}. The reaction mixture was filtered through celite filter aid and the THF removed under vacuum. Diethyl ether (40 mL) was added and the organic phase separated, washed with water (2 × 20 mL) and dried over $MgSO_4$ and filtered. After removing the solvent the crude product was purified by silica gel column chromatography (petroleum spirit : dichloromethane = 1:1) to give the crude product as a brown/orange solid. The product was purified further by washing with IPA to give the pure product as a brown / orange solid. Yield 5.06 g (7.3 mmol, 73%). R_f 0.30 (dichloromethane/petroleum spirits 1:1); ^{1}H NMR (400 MHz, $CDCl_3$) δ = 9.878 (s, 2 H), 7.703 (d, J = 4.0 Hz, 1 H), 7.668 (s, 2 H), 7.222 (m, 4 H), 7.107 (s, 2 H), 7.7004 (s, 2 H), 2.85-2.76(m, 12 H), 2.640 (t, J = 7.6 Hz, 4 H), 1.75-1.65 (m, 14 H), 1.42-1.32 (m, 58 H), 0.977 (t, J = 6.8 Hz, 6 H), 0.93-0.88 (m, 24 H); ^{13}C NMR (101 MHz; $CDCl_3$): δ = 182.5, 146.1, 142.5, 142.2, 141.2, 139.5, 139.0, 138.6, 137.25, 137.22, 136.8, 136.0, 135.7, 134.8, 130.2, 129.8, 129.5, 129.0, 128.3, 125.8, 123.5, 119.5, 77.2, 41.7, 32.7, 32.3, 31.8, 31.63, 31.62, 30.8, 30.38, 30.21, 29.77, 29.63, 29.23, 29.19, 28.9, 28.4, 26.0, 23.1, 22.69, 22.58, 14.21, 14.15, 14.07, 14.06, 11.0. MS (MALDI) m/z theoretical 1630.70, Found 161.059 [M+H$^+$]. Elemental analysis calculated for $C_{96}H_{126}O_2S_{10}$: Calculated C, 70.62; H, 7.78; S, 19.64. Found C, 70.90; H, 7.81; S, 19.42. Identical to literature.[2]

---

[2] Sun, K.; Xiao, Z.; Lu, S.; Zajaczkowski, W.; Pisula, W.; Hanssen, E.; White, J. M.; Williamson, R. M.; Subbiah, J.; Ouyang, J.; Holmes, A. B.; Wong, W. W.; Jones, D. J. *Nat. Commun.* **2015**, *6*, No. 6013. doi:10.1038/ncomms7013

S2.3.6 Synthesis of 5'',5'''''-(4,8-bis(5-(2-ethylhexyl)-4-hexylthiophen-2-yl)benzo[1,2-b:4,5-b']dithiophene-2,6-diyl)bis(3',3''-dioctyl-[2,2':5',2''-terthiophene]-5-carbaldehyde) (BT8-dialdehyde)

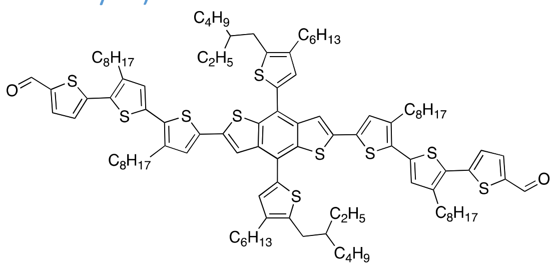

A mixture of **13** (0.5 g, 0.5 mmol), **7c** (0.63 g, 1 mmol), THF (30 mL) and 2.0 M K_3PO_4 (15 mL) was degassed by bubbling dinitrogen through for 30 minutes. The catalyst, $Pd_2(dba)_3$ (23 mg, 0.025 mmol) and P(*o*-tol)$_3$ (30.5 mg, 0.1 mmol) was added and the reaction mixture was stirred at 60 °C for 12 h. The reaction mixture was filtered through celite filter aid and the THF removed under vacuum. Diethyl ether (40 mL) was added and the organic phase separated, washed with water (2 × 20 mL) and dried over $MgSO_4$ and filtered. After removing the solvent the crude product was purified by silica gel column chromatography (petroleum spirit: dichloromethane = 1:1) to give the pure product as a red solid (0.51 g, 0.28 mmol, 59%). IR (neat) ν = 2955, 2930, 1664, 1510, 1446, 1417, 1249, 1224, 1011, 820, 754 cm^{-1}; ^{1}H NMR (600 MHz, $CDCl_3$) δ = 9.89 (s, 2H), 7.72 (d, J = 4.0, 2H), 7.68 (s, 2H), 7.24 (d, J = 4.0, 2H), 7.23 (s, 2H), 7.12 (s, 2H), 7.01 (s, 2H), 2.80 (m, 14H), 2.65 (m, 4H), 1.69 (m, 18H), 1.38-1.27 (m, 62H), 1.00-0.88 (m, 30H). ^{13}C NMR (100 MHz, $CDCl_3$) δ = 182.55, 142.47, 141.12, 139.54, 138.97, 138.58, 137.24, 136.84, 135.99, 135.74, 134.78, 129.78, 129.09, 128.31, 125.88, 123.51, 119.50, 41.69, 32.66, 32.35, 31.85, 30.75, 30.43, 30.25, 29.76, 29.56, 29.41, 29.23, 28.95, 28.43, 26.04, 23.09, 22.66, 14.22, 14.15, 14.10, 11.04. MS (MALDI) calculated for $C_{104}H_{142}O_2S_{10}$ m/z = 1742.822, found 1744.288 [M+H]$^+$. Elemental analysis calculated for $C_{104}H_{142}O_2S_{10}$: Calculated: C, 71.59; H, 8.20; O, 1.83; S, 18.38; found: C, 71.81; H, 8.34; S, 18.42.

S2.3.7 Synthesis of 5''',5'''''''-(4,8-bis(5-(2-ethylhexyl)-4-hexylthiophen-2-yl)benzo[1,2-b:4,5-b']dithiophene-2,6-diyl)bis(3',3'',3'''-trihexyl-[2,2':5',2'':5'',2'''-quaterthiophene]-5-carbaldehyde) (BQ-dialdehyde)

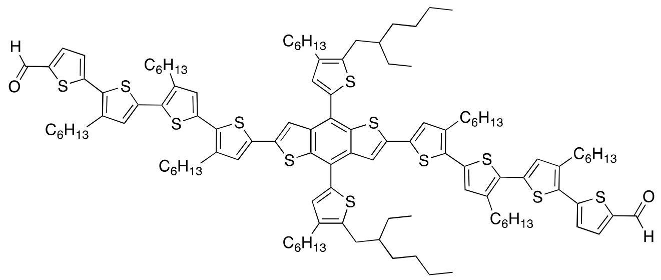

The synthesis of **BQ**-dialdehyde was completed by reacting **9b** (1.00 g, 1.36 mmol), with **13** (0.68 g, 0.68 mmol) in 30 mL THF and 15 mL of 2 M K_3PO_4 under palladium catalysis {Pd_2dba_3 (21.2 mg, 0.023 mmol), P(*o*-tolyl)$_3$ (28.1 mg, 0.092 mmol)}. The reaction mixture was filtered through celite filter aid and the THF removed under vacuum. Toluene (60 mL) was added and the organic phase separated, washed with water (2 × 20 mL) and dried over $MgSO_4$ and filtered. After removing the solvent the crude product was purified by silica gel column chromatography (gradient petroleum spirit : dichloromethane = 6:4 through to 4:6) to give the crude product as a

brown/orange solid. The product was purified further by washing with IPA to give the pure product as a brown / orange solid. Yield 0.65 g (0.33 mmol, 49%). R_f 0.20 (dichloromethane/petroleum spirits 1:1); IR (neat cm^{-1}) 2955, 2923, 2855, 1657, 1432, 1228, 1053, 818; ^1H NMR (400 MHz, $CDCl_3$) δ = δ 9.89 (d, J = 0.5 Hz, 2H), 7.71 (d, J = 4.0 Hz, 2H), 7.67 (s, 2H), 7.25 (d, J = 4.0 Hz, 2H), 7.23 (s, 2H), 7.11 (s, 2H), 7.01 (s, 2H), 6.98 (s, 2H), 2.80 (m, 16H), 2.65 (t, J = 7.6 Hz, 4H), 1.70 (m, 18H), 1.38 (m, 64H), 0.99-0.89 (m, 36H); ^{13}C NMR (100 MHz, $CDCl_3$) δ = 182.5, 146.2, 142.5, 142.1, 140.7, 140.6, 139.5, 138.9, 138.5, 137.4, 137.2, 136.8, 136.1, 135.3, 134.9, 134.3, 130.7, 129.9, 129.8, 129.3, 128.9, 128.8, 128.2, 125.8, 123.4, 119.3, 41.7, 32.7, 32.4, 31.8, 31.6, 30.8, 30.4, 30.4, 30.2, 29.8, 29.6, 29.5, 29.25, 29.20, 29.0, 28.5, 26.0, 23.1, 22.7, 22.6, 14.2, 14.2, 14.09, 14.07, 11.0. MS (MALDI) m/z = calculated 1962.86, found 1963.221 $[M+H]^+$. Elemental analysis calculated for $C_{116}H_{154}O_2S_{12}$: Calculated C, 70.89; H, 7.90; S, 19.58. Found C, 71.13; H, 7.87; S, 19.60.

S2.3.8 Synthesis of 5'''',5''''''''-(4,8-bis(5-(2-ethylhexyl)-4-hexylthiophen-2-yl)benzo[1,2-*b*:4,5-*b'*]dithiophene-2,6-diyl)bis(3',3'',3''',3''''-tetrahexyl-[2,2':5',2'':5'',2''':5''',2''''-quinquethiophene]-5-carbaldehyde) (BP-dialdehyde)

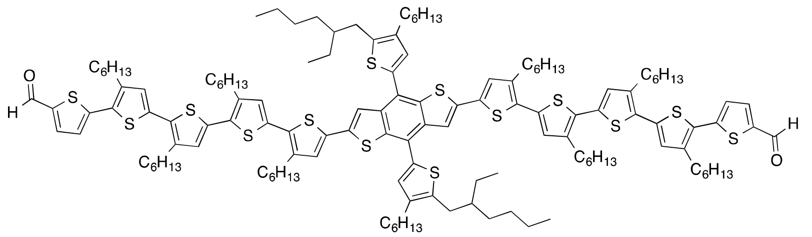

The synthesis of **BP**-dialdehyde was completed by reacting **11b** (0.20 g, 0.221 mmol), with **13** (0.111 g, 0.111 mmol) in 10 mL THF and 5 mL of 2 M K_3PO_4 under palladium catalysis {$Pd_2dba_3.4^tBu_3PHBF_4$ (6.0 mg, 0.0028 mmol)}. The reaction mixture was filtered through celite filter aid and the THF removed under vacuum. Toluene (60 mL) was added and the organic phase separated, washed with water (2 × 20 mL) and dried over $MgSO_4$ and filtered. After removing the solvent the crude product was purified by silica gel column chromatography (toulene) to give the crude product as a brown/orange solid. The product was further purified by passage through a SEC column (THF), IPA added and the THF removed under vaccum. The product collected by filtration and was purified further by washing with IPA to give the pure product as a brown / orange solid. Yield 0.189 g (0.0824 mmol, 74%). R_f 0.44 (toluene); IR (neat cm^{-1}) 2956, 2927, 2857, 1662, 1433, 1228, 1056, 819. ^1H-NMR (400 MHz; $CDCl_3$): δ 9.89 (s, 2H), 7.71 (d, J = 3.9 Hz, 2H), 7.67 (s, 2H), 7.26-7.24 (m, 4H, overlapping $CDCl_3$), 7.11 (s, 2H), 7.01 (s, 2H), 6.98 (s, 4H), 2.86-2.76 (m, 20H), 2.66 (t, J = 7.6 Hz, 4H), 1.71-1.67 (m, 22H), 1.46-1.34 (m, 76H), 1.01-0.90 (m, 42H). ^{13}C-NMR (101 MHz; $CDCl_3$): δ = 182.5, 146.2, 142.5, 142.1, 140.7, 140.4, 140.1, 139.4, 138.9, 138.5, 137.39, 137.21, 136.8, 136.2, 135.1, 134.9, 134.4, 133.8, 130.9, 130.4, 129.8, 129.3, 128.87, 128.72, 128.66, 128.2, 125.8, 123.4, 119.2, 41.7, 32.7, 32.4, 31.85, 31.65, 30.8, 30.43, 30.23, 29.8, 29.58, 29.53, 29.47, 29.25, 29.21, 29.0, 28.5, 26.0, 23.1, 22.71, 22.63, 14.23, 14.17, 14.11, 14.07, 11.0. MS (MALDI) m/z calculated for $C_{136}H_{182}O_2S_{14}$ 2295.0230, found 2295.455 $[M^+]$. Elemental analysis calculated for $C_{136}H_{182}O_2S_{14}$: Calculated C, 71.09; H, 7.98; S, 19.53. Found C, 71.20; H, 7.95; S, 19.64.

Synthesis of BXR and BT^xR molecules

S2.3.9 Synthesis of BMR molecule (5Z,5'Z)-5,5'-((5,5'-(4,8-bis(5-(2-ethylhexyl)-4-hexylthiophen-2-yl)benzo[1,2-b:4,5-b']dithiophene-2,6-diyl)bis(thiophene-5,2-diyl))bis(methanylylidene))bis(3-hexyl-2-thioxothiazolidin-4-one) (BMR)

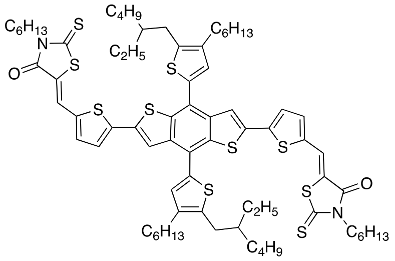

Compound **BM**-dialdehyde (158 mg, 0.163 mmol), *N*-hexylrhodanine (355 mg, 1.63 mmol) and $CHCl_3$ (2 ml) were combined in a µw vial and degassed with N_2 for 10 mins. To this DBU (one drop) was added to the reaction mixture and the reaction mixture continued to stir at room temperature for five hours. The $CHCl_3$ was removed under vacuum and the crude product was purified via column chromatography on silica with a gradient of DCM:PET 2:3 to 1:1 (R_f 0.5). ^{1}H-NMR (500 MHz; $Cl_2CDCDCl_2$): δ 7.89 (s, 2H), 7.82 (s, 2H), 7.38 (d, J = 1.2 Hz, 4H), 7.29 (s, 2H), 4.10 (t, J = 7.7 Hz, 4H), 2.83-2.81 (m, 4H), 2.69-2.66 (m, 4H), 1.73-1.70 (m, 11H), 1.49-1.39 (m, 46H), 1.00 (t, J = 7.4 Hz, 7H), 0.96-0.89 (m, 21H). ^{13}C-NMR (126 MHz; $Cl_2CDCDCl_2$): δ 191.74, 191.72, 167.2, 144.8, 140.0, 139.15, 139.02, 137.8, 137.2, 136.7, 135.1, 133.8, 129.9, 126.7, 124.6, 124.1, 121.4, 121.1, 44.8, 41.5, 32.6, 32.2, 31.7, 31.2, 30.6, 29.1, 28.8, 28.3, 26.8, 26.3, 25.9, 23.0, 22.7, 22.4, 14.23, 14.18, 14.02, 11.1. IR (neat) ν = 2954, 2923, 2855, 1701, 1581,, 1541, 1317, 1237, 1179, 1138, 794 cm^{-1} MS (ESI) m/z calculated for $C_{74}H_{96}N_2O_2S_{10}$ 1364.47, found 1364.4767 $[M]^{\bullet+}$, MS (MALDI) m/z 1364.843 $[M]^{\bullet+}$. Elemental analysis calculated for $C_{74}H_{96}N_2O_2S_{10}$: Calculated: C, 65.05; H, 7.08; N, 2.05; O, 2.34; S, 23.47; Found: C, 65.24; H, 7.09; N, 1.78; S, 23.49.

S2.3.10 Synthesis of BBR molecule (5Z,5'Z)-5,5'-((5',5'''-(4,8-bis(5-(2-ethylhexyl)-4-hexylthiophen-2-yl)benzo[1,2-b:4,5-b']dithiophene-2,6-diyl)bis(3'-hexyl-[2,2'-bithiophene]-5',5-diyl))bis(methanylylidene))bis(3-hexyl-2-thioxothiazolidin-4-one)

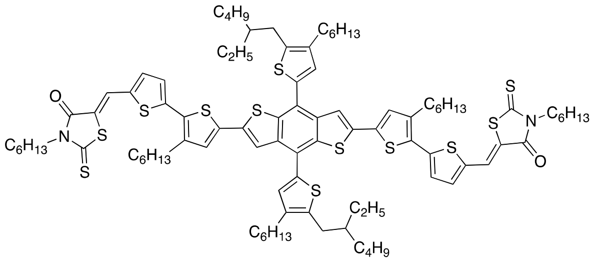

Compound **BB**-dialdehyde (300 mg, 0.231 mmol), *N*-hexylrhodanine (502 mg, 2.31 mmol) and $CHCl_3$ (5 ml) were combined in a µw vial and degassed with N_2 for 10 mins. To this DBU (one drop) was added to the reaction mixture and the reaction mixture continued to stir at room temperature for three hours. The $CHCl_3$ was removed under vacuum and the crude product was purified via column chromatography on silica with toluene (R_f 0.8). Further purification was performed with SEC and Toluene. Finally, the product was isolated and precipitated from $CHCl_3$ with IPA to give the pure product. Yield 183 mg (47%). ^{1}H-NMR (500 MHz; $CDCl_3$): δ 7.83 (d, J = 0.5 Hz, 2H), 7.70 (s, 2H), 7.35 (dd, J = 4.1, 0.4 Hz, 2H), 7.23 (s, 2H), 7.21 (d, J = 4.0 Hz, 2H), 7.13 (s, 2H), 4.11 (t, J = 7.7 Hz, 4H), 2.82-2.78 (m, 8H), 2.66 (t, J = 7.6 Hz, 4H), 1.73-1.67 (m, 14H), 1.48-1.43 (m, 17H), 1.35 (ddt, J = 14.5, 7.1, 3.5 Hz, 40H), 0.99 (t, J = 7.4 Hz, 6H), 0.95-0.86 (m,

28H). ^{13}C-NMR (126 MHz; $CDCl_3$): δ = 192.3, 167.7, 144.2, 142.6, 139.8, 139.2, 138.9, 137.46, 137.45, 137.18, 137.14, 134.82, 134.71, 130.18, 130.03, 128.8, 126.9, 125.0, 123.8, 120.6, 120.2, 45.0, 41.9, 32.9, 32.5, 32.0, 31.8, 31.5, 30.9, 30.5, 30.1, 29.46, 29.39, 29.1, 28.6, 27.1, 26.6, 26.2, 23.3, 22.88, 22.79, 22.66, 14.40, 14.34, 14.27, 14.16, 11.2. IR (neat) ν = 2954, 2923, 2855, 1701, 1577, 1424, 1318, 1231, 1180, 1136, 791 cm^{-1}. MS (ESI) m/z calculated for $C_{94}H_{124}N_2O_2S_{12}$ 1696.63, found 1697.6349 $[M+H]^+$. MS (MALDI) m/z 1697.839 $[M+H]^+$. Elemental analysis calculated for $C_{94}H_{124}N_2O_2S_{12}$: Calculated: C, 66.46; H, 7.36; N, 1.65; S, 22.65; Found: C, 66.32; H, 7.25; N, 1.49; S, 22.49.

S2.3.11 Synthesis of 5'',5'''''-(4,8-bis(5-(2-ethylhexyl)-4-hexylthiophen-2-yl)benzo[1,2-b:4,5-b']dithiophene-2,6-diyl)bis(3',3''-dibutyl-[2,2':5',2''-terthiophene]-5-hexylrhodanine) (BT^4R)

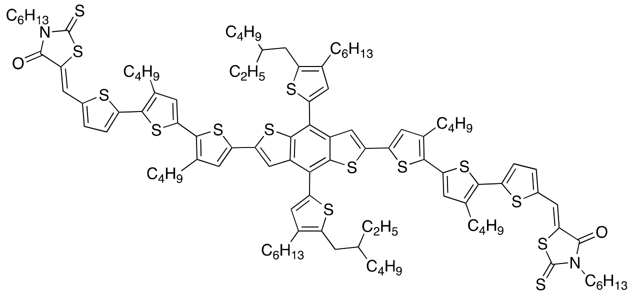

A solution of $\mathbf{BT^4}$-dialdehyde (0.3 g, 0.2 mmol), *N*-hexylrhodanine (0.434 g, 2 mmol) and 1 drop of DBU was stirred in chloroform (5 mL). The reaction mixture was then stirred at room temperature for 3 hours. The solvent was then removed via rotary evaporation and the crude product purified via column chromatography (petroleum spirit: dichloromethane = 1:1) and then washed with isopropanol to yield a red powder. This was then further purified via size-exclusion chromatography (toluene, BioBeads 100) to yield a purple solid (170 mg, 0.08 mmol, 40%). IR (neat) ν = 2953, 2928, 2856, 1700, 1578, 1412, 1328, 1184, 820 cm^{-1}; ^1H NMR (600 MHz, $CDCl_3$) δ = 7.80 (s, 2H), 7.63 (s, 2H), 7.32 (d, J = 4.0, 2H), 7.24 (s, 2H), 7.17 (d, J = 4.0, 2H), 7.08 (s, 2H), 6.98 (s, 2H), 4.08, (t, J = 7.8, 4.0, 4H), 2.80 (m, 12H), 2.65 (m, 4H), 1.70 (m, 20H), 1.47-1.37 (m, 46H), 1.00-0.88 (m, 36H). ^{13}C NMR (400MHz, $CDCl_3$) δ = 192.16, 167.42, 144.24, 141.77, 140.88, 139.46, 138.95, 138.50, 137.18, 136.96, 135.62, 135.57, 134.85, 134.56, 130.45, 129.82, 129.67, 128.96, 128.23, 126.45, 124.94, 123.39, 120.09, 119.39, 44.83, 41.71, 32.71, 32.47, 32.37, 31.88, 31.32, 30.81, 29.55, 29.44, 29.24, 28.98, 28.49, 26.91, 26.45, 26.06, 23.14, 22.73, 22.71, 22.51, 14.26, 14.19, 14.00, 13.97, 11.08. MS (MALDI) m/z = 1919.429. Elemental analysis calculated for $C_{106}H_{136}N_2O_2S_{14}$: Calculated: C, 66.34; H, 7.14; N, 1.46; S, 23.39; found: C, 66.19; H, 7.13; N, 1.40; S, 23.28

S2.3.12 Synthesis of BTR, (5*Z*,5'*Z*)-5,5'-((5'',5'''''-(4,8-bis(5-(2-ethylhexyl)-4-hexylthiophen-2-yl)benzo[1,2-*b*:4,5-*b'*]dithiophene-2,6-diyl)bis(3',3''-dihexyl-[2,2':5',2''-terthiophene]-5'',5-diyl))bis(methanylylidene))bis(3-hexyl-2-thioxothiazolidin-4-one) (BTR)

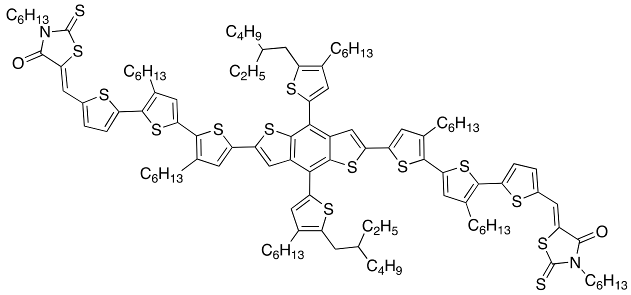

To a solution of compound **BT**-dialdehyde (327 mg, 0.2 mmol) in dry chloroform (20 mL) was added *n*-hexylrhodanine (434 mg, 2.0 mmol) followed by one drop of DBU. The reaction

mixture was then kept stirring at room temperature for 3 h. Reaction solvent was removed by rotary evaporator and the crude product was purified through silica gel column chromatography and then washed with acetone (petroleum spirit : dichloromethane = 2:1) to give the product as purple solid (310 mg, 76%). R_f 0.8 (dichloromethane/petroleum spirits 1:1); mp 178-180 ^oC; IR (neat) ν 2928, 2857, 1702, 1579, 1425, 1328, 1243, 1185, 821 cm^{-1}; ^1H NMR (400 MHz, $CDCl_3$) δ = 7.83 (s, 2 H), 7.65 (s, 2 H), 7.35 (d, J = 4.0 Hz, 2 H), 7.23 (s, 2 H), 7.20 (d, J = 4.0 Hz, 2 H), 7.10 (s, 2 H), 7.00 (s, 2 H), 4.09 (t, J = 7.6 Hz, 4 H), 2.81 (m, 12 H), 2.78 (m, 4 H), 1.60-1.75 (m, 18 H), 1.26-1.45 (m, 64 H), 0.88 (m, 36 H); ^{13}C NMR (100 MHz, $CDCl_3$) δ = 192.1, 167.4, 144.2, 141.8, 140.9, 139.4, 138.9, 138.5, 137.2, 136.9, 135.6, 135.5, 134.9, 134.5, 130.4, 129.8, 129.6, 128.9, 128.2, 126.3, 124.9, 123.3, 120.0, 119.4, 44.8, 41.7, 32.7, 32.4, 31.9, 31.72, 31.67, 31.3, 30.8, 30.30, 30.25, 29.9, 29.7, 29.34, 29.32, 29.2, 29.0, 28.5, 26.9, 26.4, 26.0, 23.1, 22.73, 22.67, 22.6, 22.5, 14.3, 14.2, 14.1, 14.0, 11.1; MS (MALDI) m/z 2028.748, found 2029.394 [M+H]. Micro Calc. for $C_{114}H_{152}N_2O_2S_{14}$: C, 67.41; H, 7.54; N, 1.38; S, 22.10. Found C, 67.57; H, 7.57; N, 1.25; S, 22.04. Identical to literature.[3]

S2.3.13 Synthesis of 5'',5''''-(4,8-bis(5-(2-ethylhexyl)-4-hexylthiophen-2-yl)benzo[1,2-b:4,5-b']dithiophene-2,6-diyl)bis(3',3''-dioctyl-[2,2':5',2''-terthiophene]-5-hexylrhodanine) (BT^8R)

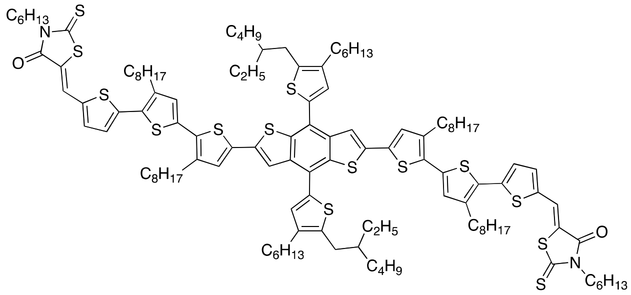

A solution of $\mathbf{BT^8}$-dialdehyde (0.349 g, 0.2 mmol), *N*-hexylrhodanine (0.434 g, 2 mmol) and 1 drop of DBU was stirred in chloroform (20 mL). The reaction mixture was then stirred at room temperature for 3 hours. The solvent was then removed via rotary evaporation and the crude product purified via column chromatography (petroleum spirit: dichloromethane = 1:1) and then washed with isopropanol to yield a red powder. This was then further purified via size-exclusion chromatography (toluene, BioBeads 100) to yield a purple solid (170 mg, 0.08 mmol, 40%). IR (neat) ν = 2957, 2870, 1698, 1579, 1460, 1425, 1378, 1185, 1019, 888, 824 cm^{-1}; ^1H NMR (600 MHz, $CDCl_3$) δ = 7.80 (s, 2H), 7.63 (s, 2H), 7.32 (d, J = 4.0, 2H), 7.24 (s, 2H), 7.17 (d, J = 4.0, 2H), 7.08 (s, 2H), 6.98 (s, 2H), 4.08, (t, J = 7.8, 4.0, 4H), 2.80 (m, 14H), 2.65 (m, 4H), 1.69 (m, 22H), 1.38-1.27 (m, 74H), 1.00-0.88 (m, 36H). ^{13}C NMR (100 MHz, $CDCl_3$) δ = 192.16, 167.42, 144.24, 141.77, 140.88, 139.46, 138.95, 138.50, 137.18, 136.96, 135.62, 135.57, 134.85, 134.56, 130.45, 129.82, 129.67, 128.96, 128.23, 126.45, 124.94, 123.39, 120.09, 119.39, 44.83, 41.69, 32.67, 32.36, 31.88, 31.33, 30.76, 30.40, 29.80, 29.61, 29.40, 29.25, 28.95, 28.45, 26.94, 26.44, 26.05, 23.10, 22.68, 22.49, 14.11, 13.99, 11.04. MS (MALDI) m/z $[M+H]^+$ = 2143.229. Elemental analysis calculated for $C_{122}H_{168}N_2O_2S_{14}$: Calculated: C, 68.36; H, 7.90; N, 1.31; S, 20.94; found: C, 67.54; H, 7.89; N, 1.21; S, 19.72.

---

[3] Sun, K.; et al. *Nat. Commun.* **2015**, *6*, 6013. doi:10.1038/ncomms7013

S2.3.14 Synthesis of BQR molecule, (5Z,5'Z)-5,5'-((5''',5''''''-(4,8-bis(5-(2-ethylhexyl)-4-hexylthiophen-2-yl)benzo[1,2-b:4,5-b']dithiophene-2,6-diyl)bis(3',3'',3'''-trihexyl-[2,2':5',2'':5'',2'''-quaterthiophene]-5''',5-diyl))bis(methanylylidene))bis(3-hexyl-2-thioxothiazolidin-4-one) (BQR)

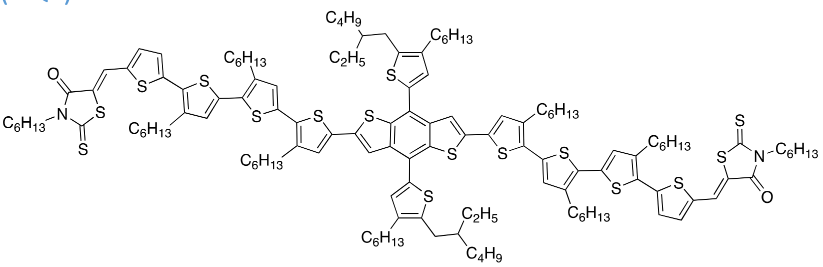

The solution of compound **BQ**-dialdehyde (115 mg, 0.058 mmol) in dry chloroform (5 mL) was added *N*-hexylrhodanine (127 mg, 0.585 mmol) followed by one drop of DBU. The reaction mixture was then kept stirring at room temperature for 3 h. The product was precipitated by addition of IPA (20 mL) and recovered by filtration. The crude product was purified through silica gel column chromatography and then washed with acetone (petroleum spirit : dichloromethane = 6:4) to give the product as purple solid (99 mg, 72%). R_f 0.83 (dichloromethane/petroleum spirits 1:1); IR (neat) ν 2927, 2857, 1695, 1578, 1421, 1329, 1184, 822 cm^{-1}; ^{1}H NMR (400 MHz, $CDCl_3$) δ = δ 7.83 (s, 2H), 7.65 (s, 2H), 7.35 (d, J = 4.0 Hz, 2H), 7.23 (s, 2H), 7.21 (d, J = 4.0 Hz, 2H), 7.09 (s, 2H), 6.99 (s, 2H), 6.97 (s, 2H), 4.10 (t, J = 7.7 Hz, 4H), 2.84-2.75 (m, 16H), 2.66 (t, J = 7.6 Hz, 4H), 1.76-1.65 (m, 20H), 1.48-1.33 (m, 78H), 1.01-0.89 (m, 42H); ^{13}C NMR (100 MHz, $CDCl_3$) δ = 192.2, 167.5, 144.4, 141.9, 140.5, 139.4, 138.9, 138.5, 137.3, 137.2, 137.0, 135.7, 135.2, 134.9, 134.7, 134.2, 130.8, 130.0, 129.8, 129.5, 128.9, 128.7, 128.2, 126.5, 125.0, 123.4, 120.1, 119.3, 44.8, 41.7, 32.7, 32.4, 31.9, 31.67, 31.65, 31.3, 30.8, 30.4, 30.3, 29.8, 29.61, 29.56, 29.30, 29.26, 29.2, 29.0, 26.9, 26.4, 26.0, 23.1, 22.71, 22.65, 22.6, 22.5, 14.23, 14.17, 14.1, 14.0, 11.0. MS (MALDI) calculated m/z 2360.96, found 2361.516 [$M+H^+$]. Micro Calc. for $C_{134}H_{180}N_2O_2S_{16}$: C, 68.08; H, 7.68; N, 1.19; S, 21.70. Found C, 68.10; H, 7.66; N, 1.04; S, 21.72.

S2.3.15 Synthesis of BPR molecule (5Z,5'Z)-5,5'-((5'''',5''''''''-(4,8-bis(5-(2-ethylhexyl)-4-hexylthiophen-2-yl)benzo[1,2-b:4,5-b']dithiophene-2,6-diyl)bis(3',3'',3''',3''''-tetrahexyl-[2,2':5',2'':5'',2''':5''',2''''-quinquethiophene]-5'''',5-diyl))bis(methanylylidene))bis(3-hexyl-2-thioxothiazolidin-4-one) (BPR)

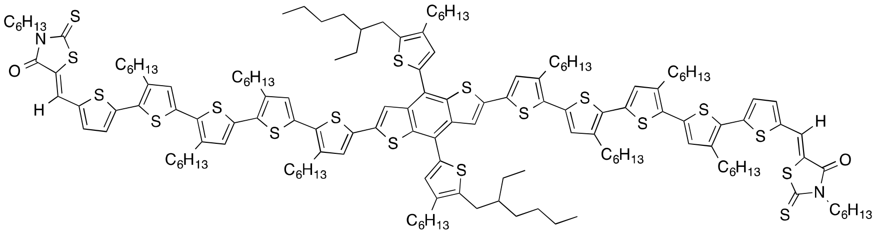

To a solution of **BP**-dialdehyde (0.170 g, 0.074 mmol) and *N*-hexylrhodanine (0.161 g, 0.74 mmol) in 10 mL of $CHCl_3$ under nitrogen was added 2 drops of DBU. The reaction mixture changed colour immediately. The reaction was allowed to react for 16 hours. IPA (30 mL) was added to precipitate the product, which was collected by filtration, washed with IPA, ethyl acetate and then acetone. The crude product was dissolved in $CHCl_3$, filtered through celite and then the solvent removed under vacuum. The product was purified by column chromatography using 50:50 toluene:cyclohexane, R_f = 0.73. The product was collected then dissolved in a minimum THF and then purified further using SEC. To the THF solution collected was added 50 mL of IPA and the THF removed on a rotovap leaving a dark precipitate in IPA, which was collected by filtration, washed with IPA, ethyl acetate and the acetone before being dried in air. The pure product was then dried at 50°C under vacuum overnight. Yield 0.144 g (0.053 mmol, 72%). IR (neat) ν = CO 1700 cm^{-1}; ^{1}H-NMR (400 MHz; $CDCl_3$): δ = 7.83 (s, 2H), 7.65 (s, 2H), 7.34 (d, J = 4.0 Hz, 2H), 7.24 (s, 2H), 7.20 (d, J = 4.0 Hz, 2H), 7.09 (s, 2H), 7.00 (s, 2H), 6.97 (s, 4H), 4.09 (t, J = 7.6 Hz, 4H), 2.85-

2.75 (m, 20H), 2.66 (t, *J* = 7.5 Hz, 4H), 1.70 (d, *J* = 6.1 Hz, 26H), 1.46-1.34 (m, 88H), 1.02-0.90 (m, 48H); ^{13}C NMR (101 MHz; $CDCl_3$): δ 192.1, 167.5, 144.4, 141.9, 140.53, 140.36, 140.0, 139.4, 138.9, 138.5, 137.36, 137.19, 137.0, 135.8, 135.05, 134.91, 134.7, 134.3, 133.8, 130.9, 130.4, 129.87, 129.77, 129.4, 128.85, 128.67, 128.61, 128.2, 126.4, 125.0, 123.3, 120.1, 119.2, 44.8, 41.7, 32.7, 32.4, 31.86, 31.69, 31.67, 31.3, 30.8, 30.43, 30.40, 30.31, 29.9, 29.61, 29.57, 29.50, 29.31, 29.27, 29.22, 29.0, 28.5, 26.9, 26.4, 26.0, 23.1, 22.72, 22.67, 22.50, 14.23, 14.17, 14.00, 11.0. MS (MALDI) m/z calculated for $C_{154}H_{208}N_2O_2S_{18}$ 2693.1209, found 2693.617 [M^+]. Elemental Analysis calculated for $C_{154}H_{208}N_2O_2S_{18}$: C, 68.60; H, 7.78; N, 1.04; S, 21.40. Found C, 69.00; H, 7.79; N, 0.85; S, 21.27.

S3 ^{1}H and ^{13}C spectra of all new compounds.

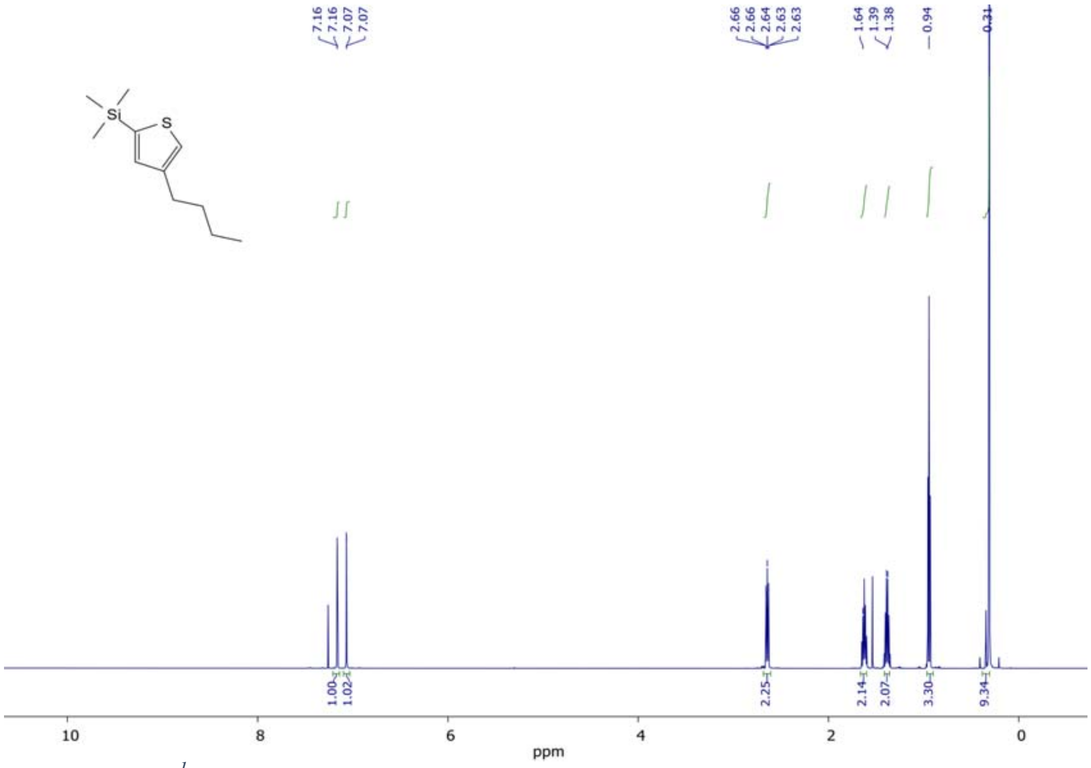

Figure S3.1. ^{1}*H-NMR of (4-butylthiophen-2-yl)trimethylsilane (2a)*

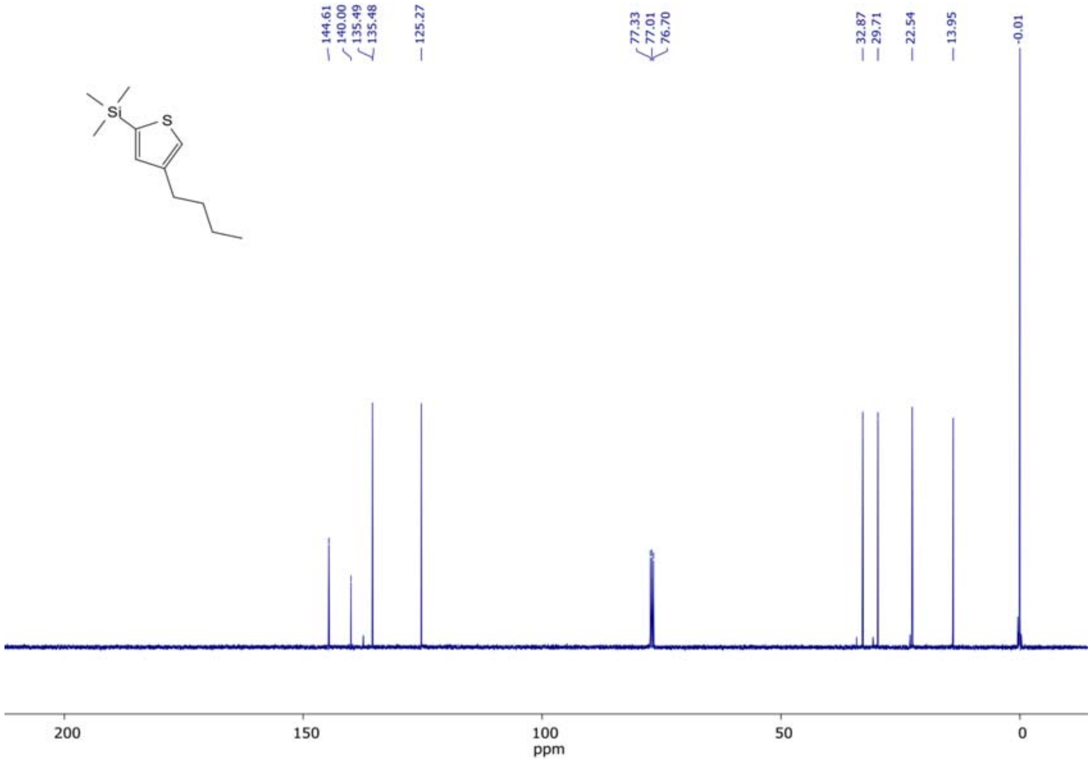

Figure S3.2. ^{13}*C-NMR of (4-butylthiophen-2-yl)trimethylsilane (2a)*

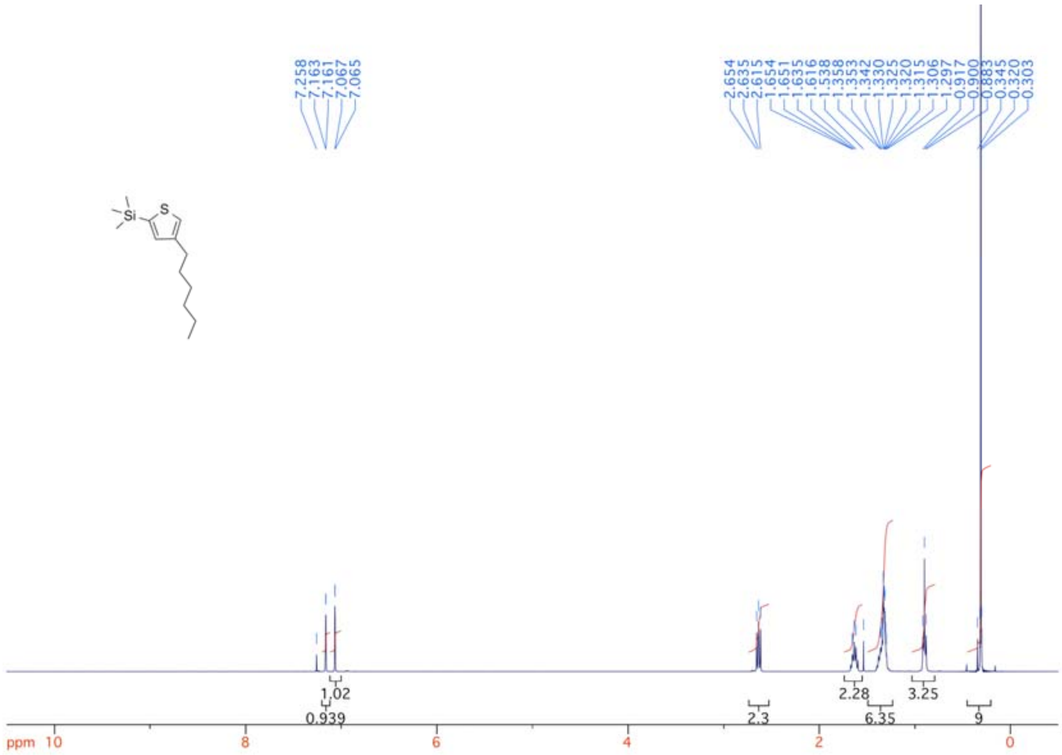

Figure S3.3. *^{1}H-NMR of (4-hexylthiophen-2-yl)trimethylsilane (2b)*

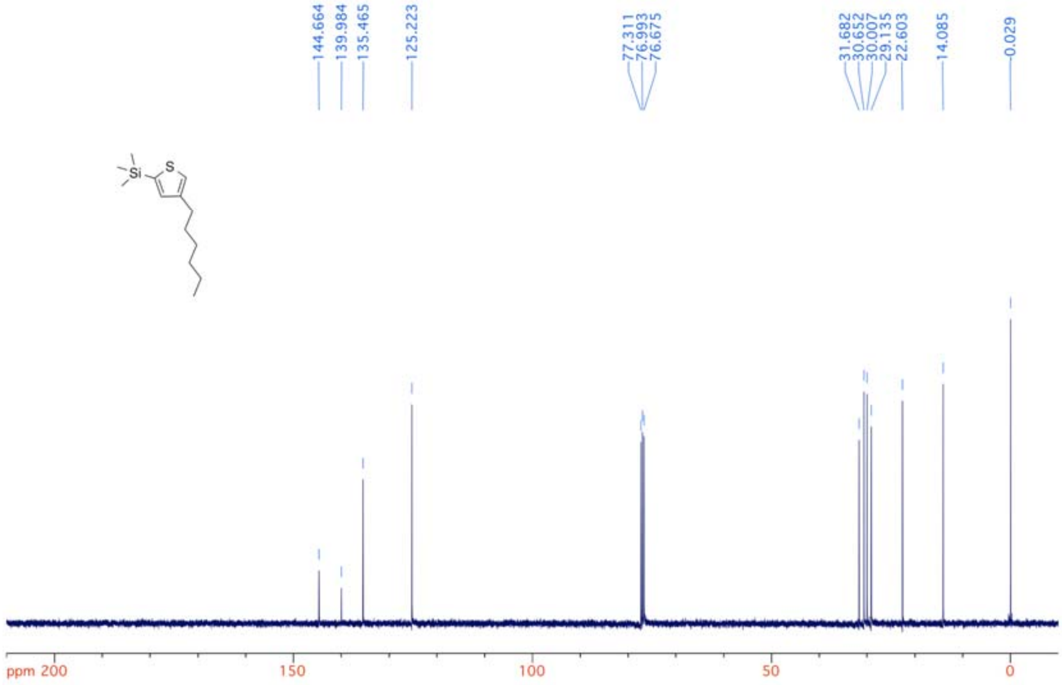

Figure S3.4. *^{13}C-NMR of (4-hexylthiophen-2-yl)trimethylsilane (2b)*

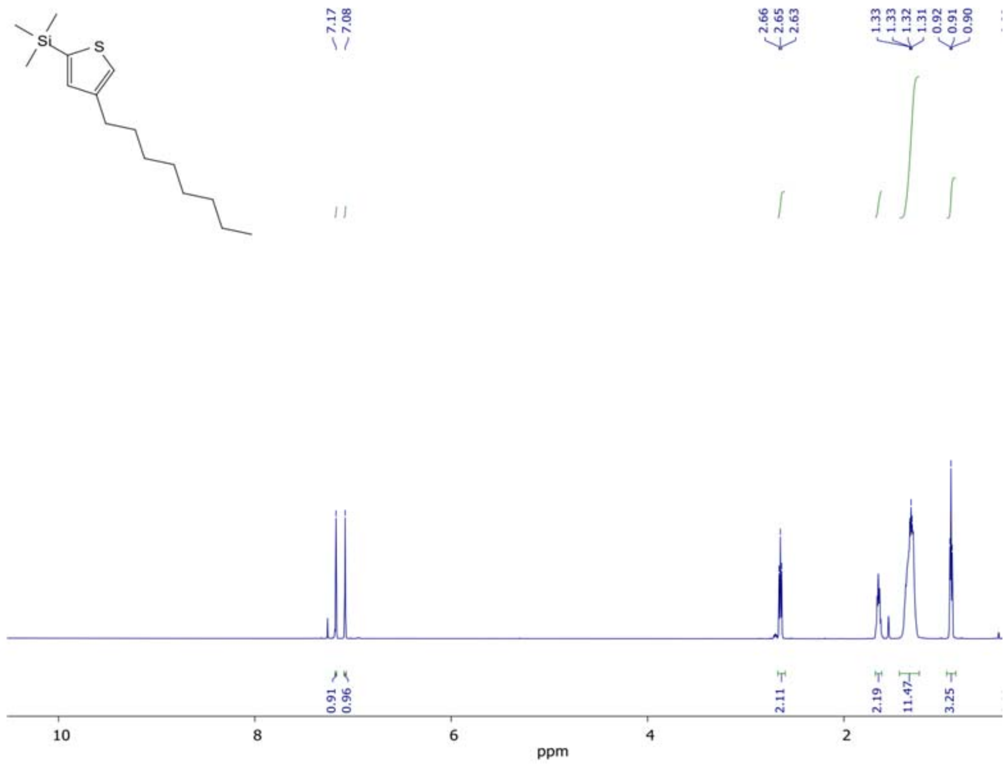

Figure S3.5. [1]*H-NMR of (4-octylthiophen-2-yl)trimethylsilane (2c)*

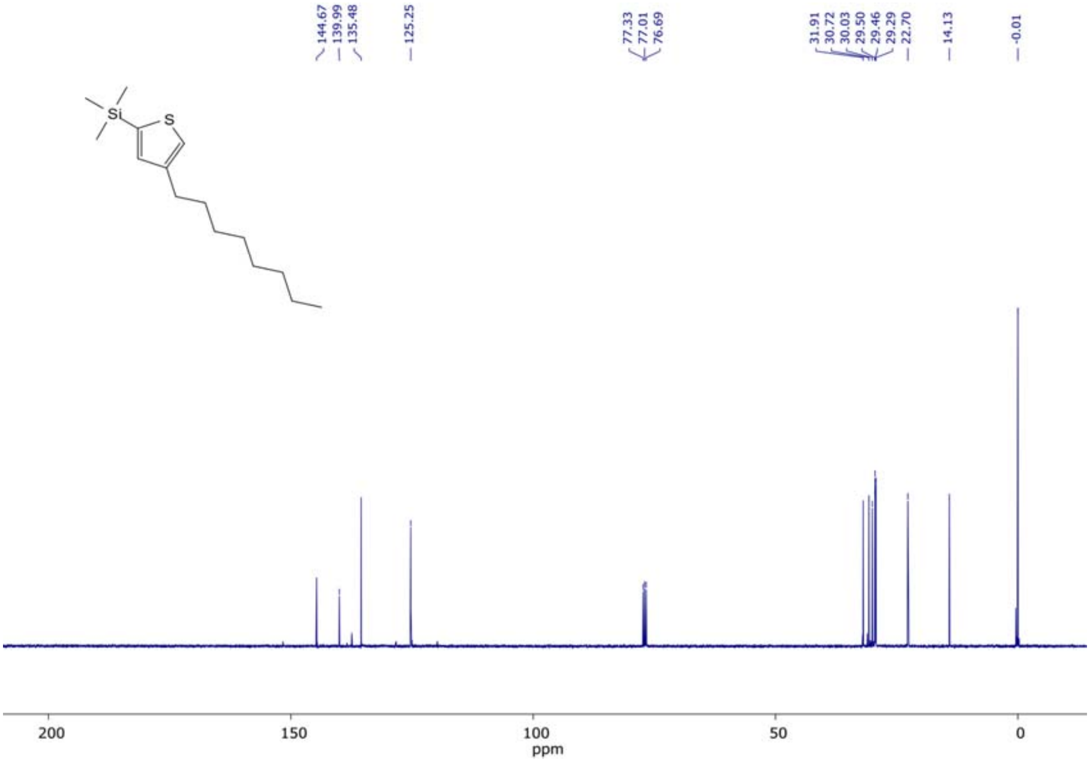

Figure S3.6. [13]*C-NMR of (4-octylthiophen-2-yl)trimethylsilane (2c)*

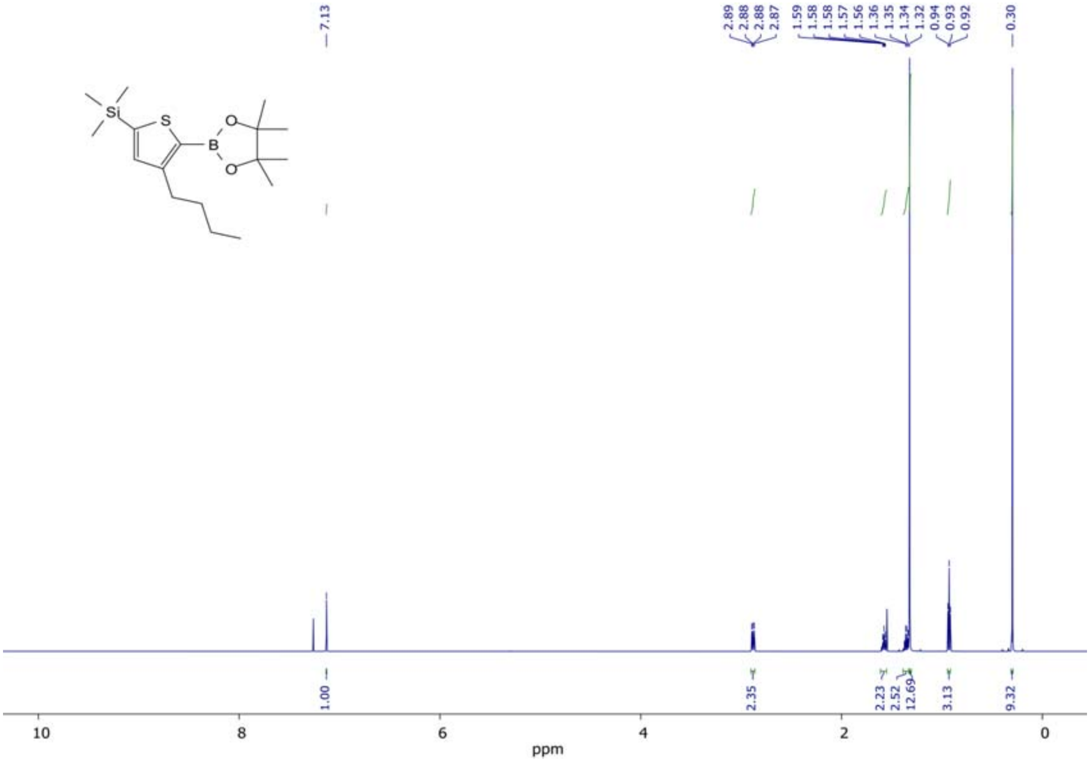

Figure S3.7. *^{1}H-NMR of (4-butyl-5-(4,4,5,5-tetramethyl-1,3,2-dioxaborolan-2-yl)thiophen-2-yl)trimethylsilane (3a)*

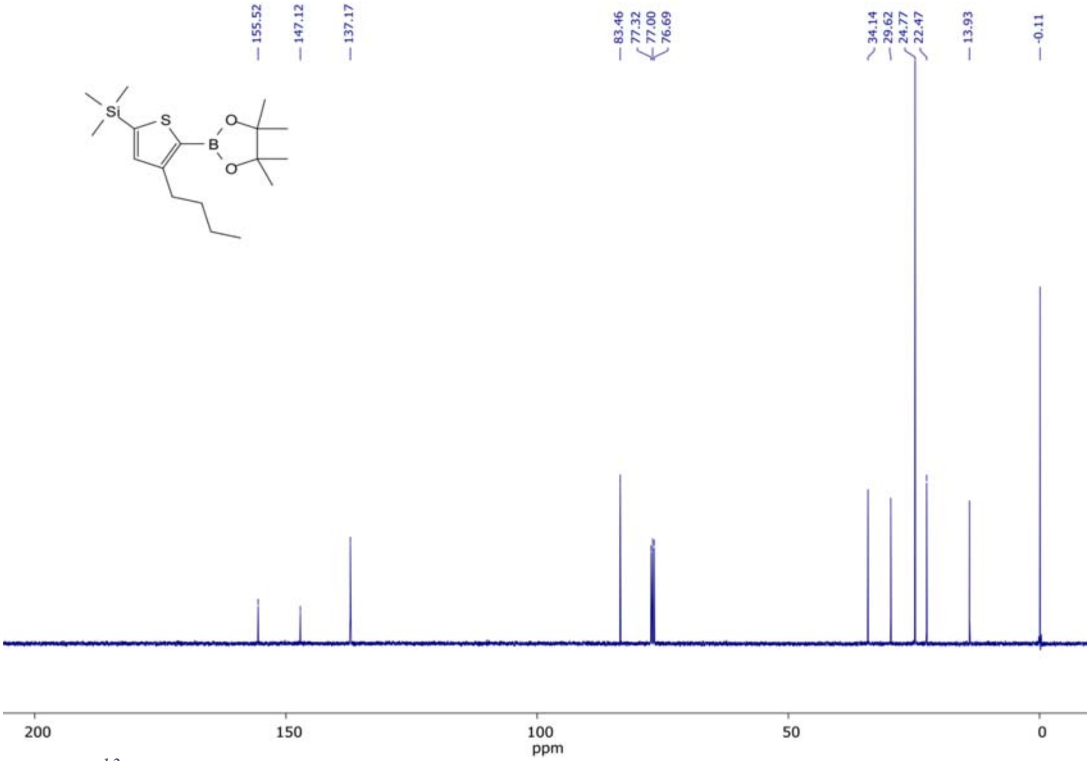

Figure S3.8. *^{13}C-NMR of (4-butyl-5-(4,4,5,5-tetramethyl-1,3,2-dioxaborolan-2-yl)thiophen-2-yl)trimethylsilane (3a)*

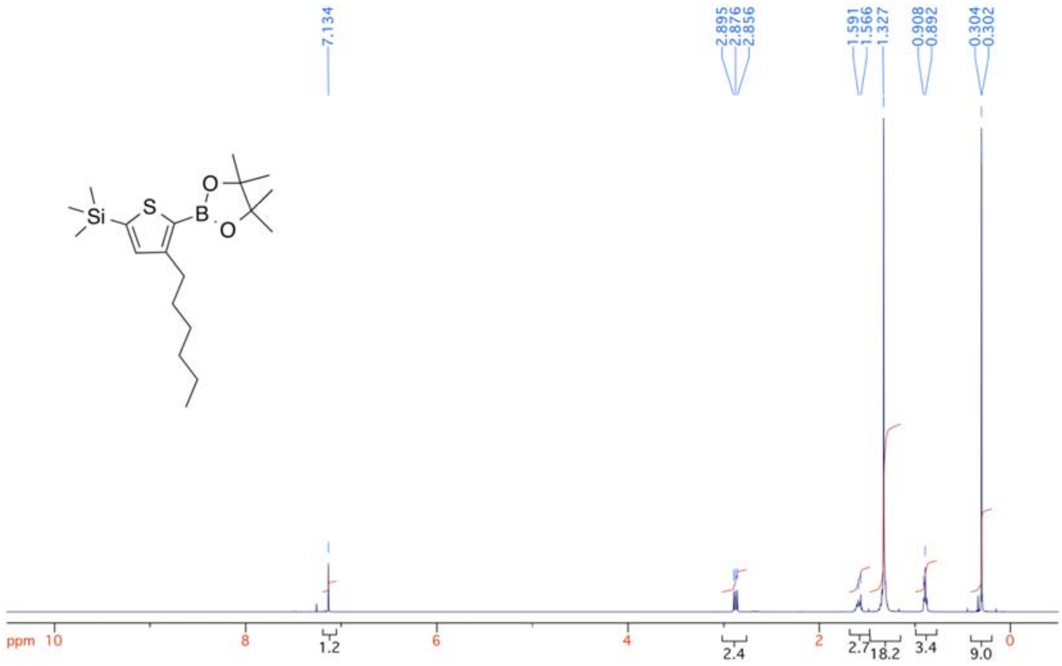

Figure S3.9. [1]*H-NMR of (4-hexyl-5-(4,4,5,5-tetramethyl-1,3,2-dioxaborolan-2-yl)thiophen-2-yl)trimethylsilane (3b)*

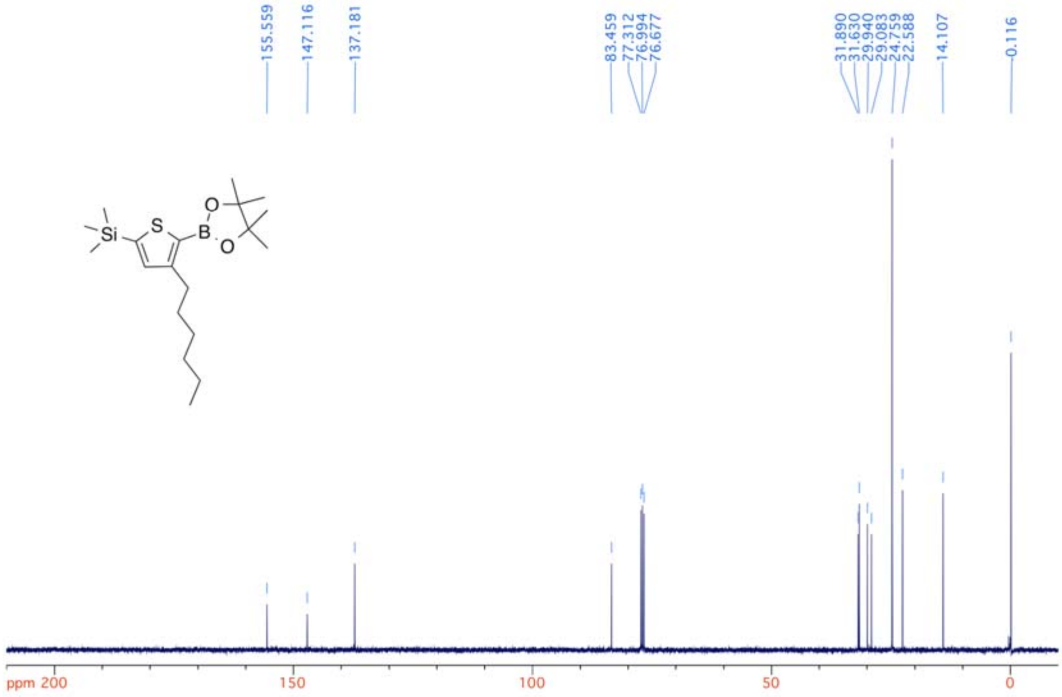

Figure S3.10. [13]*C-NMR of (4-hexyl-5-(4,4,5,5-tetramethyl-1,3,2-dioxaborolan-2-yl)thiophen-2-yl)trimethylsilane (3b)*

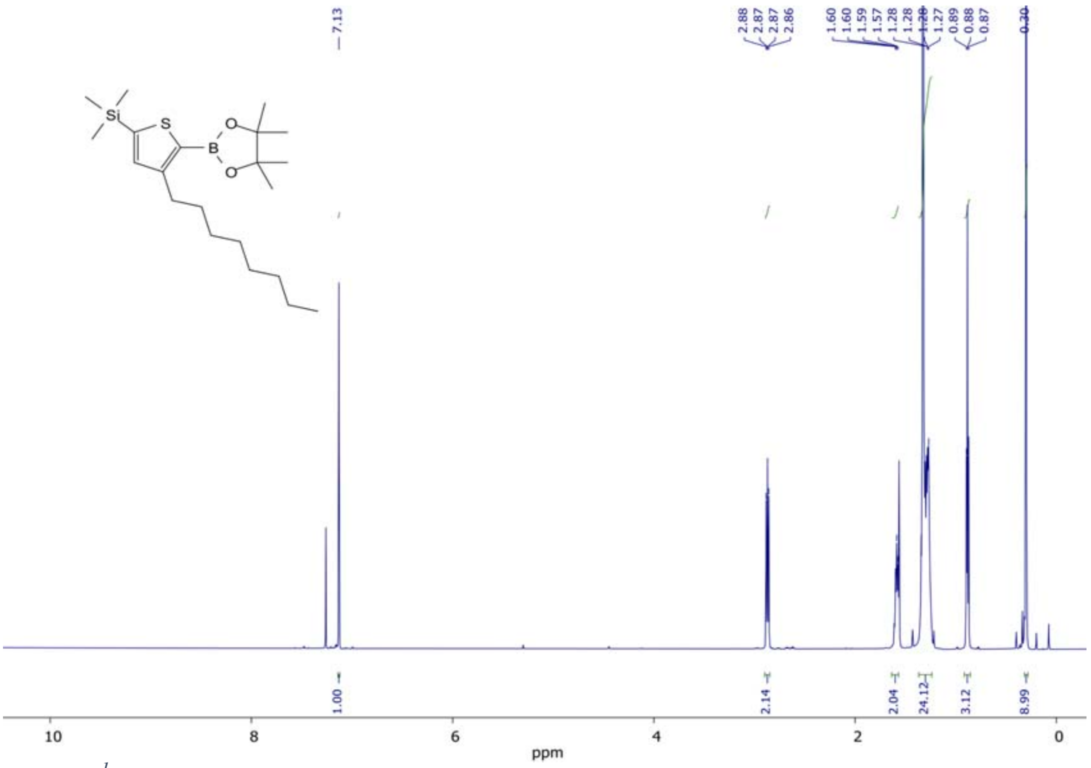

Figure S3.11. [1]*H-NMR of (4-octyl-5-(4,4,5,5-tetramethyl-1,3,2-dioxaborolan-2-yl)thiophen-2-yl)trimethylsilane (3c)*

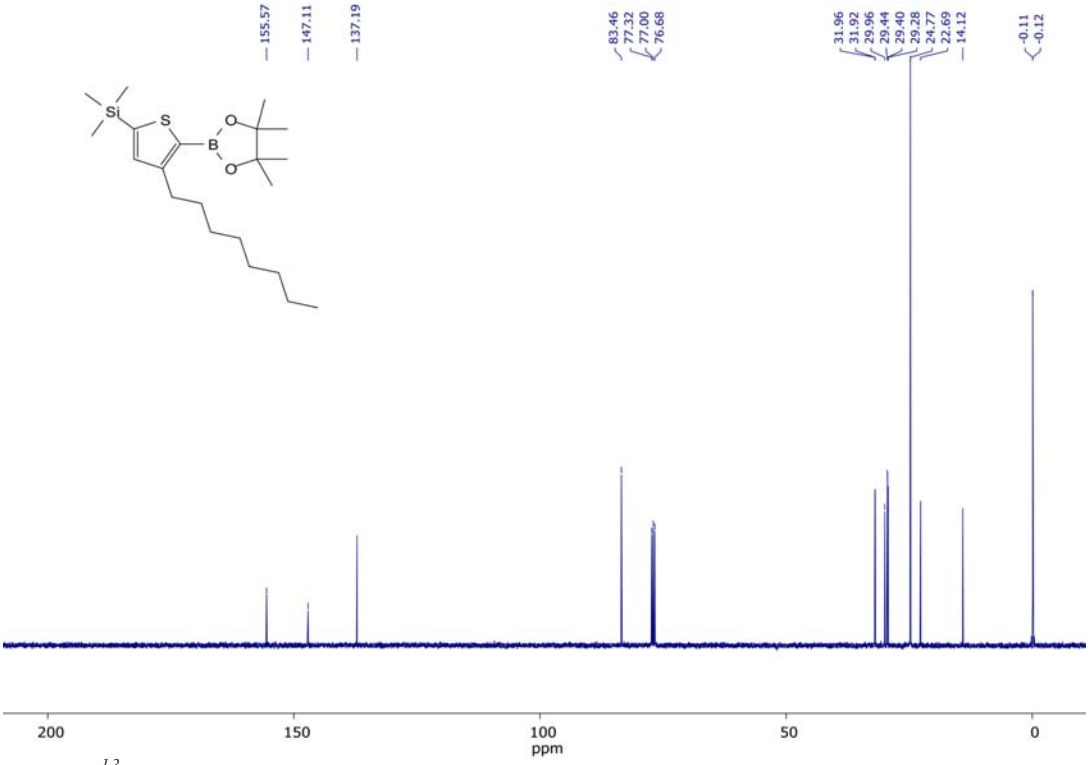

Figure S3.12. [13]*C-NMR of (4-octyl-5-(4,4,5,5-tetramethyl-1,3,2-dioxaborolan-2-yl)thiophen-2-yl)trimethylsilane (3c)*

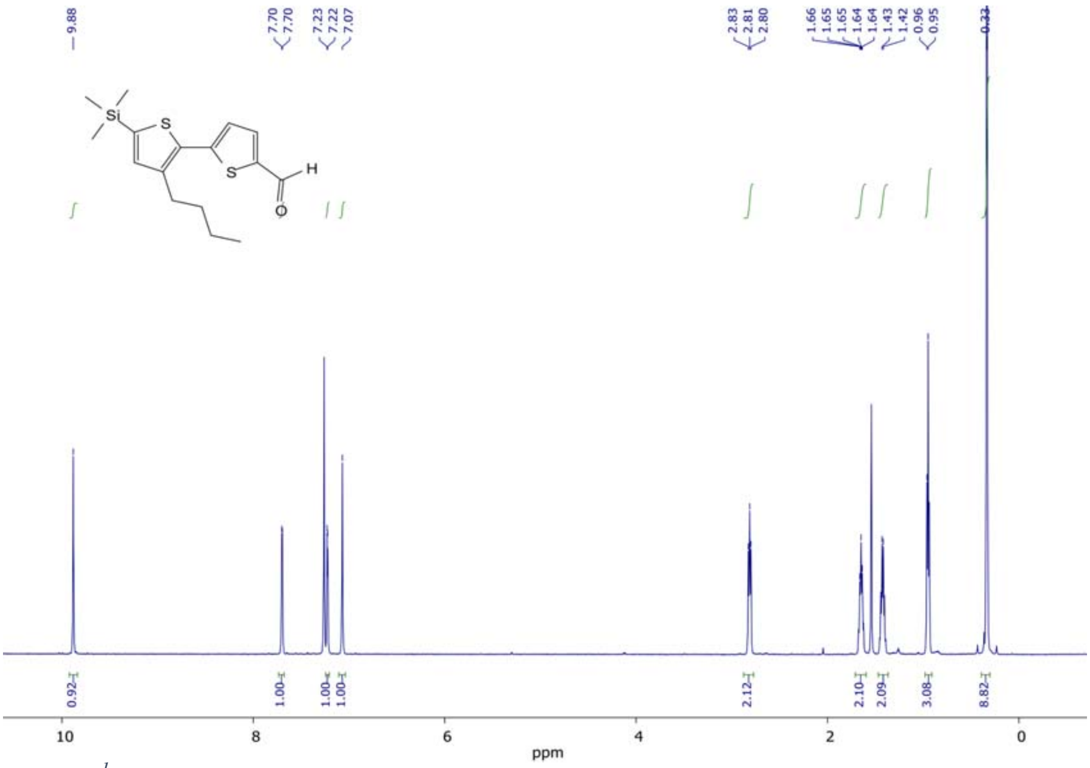

Figure S3.13. [1]*H-NMR of 3'-butyl-5'-(trimethylsilyl)-[2,2'-bithiophene]-5-carbaldehyde (4a)*

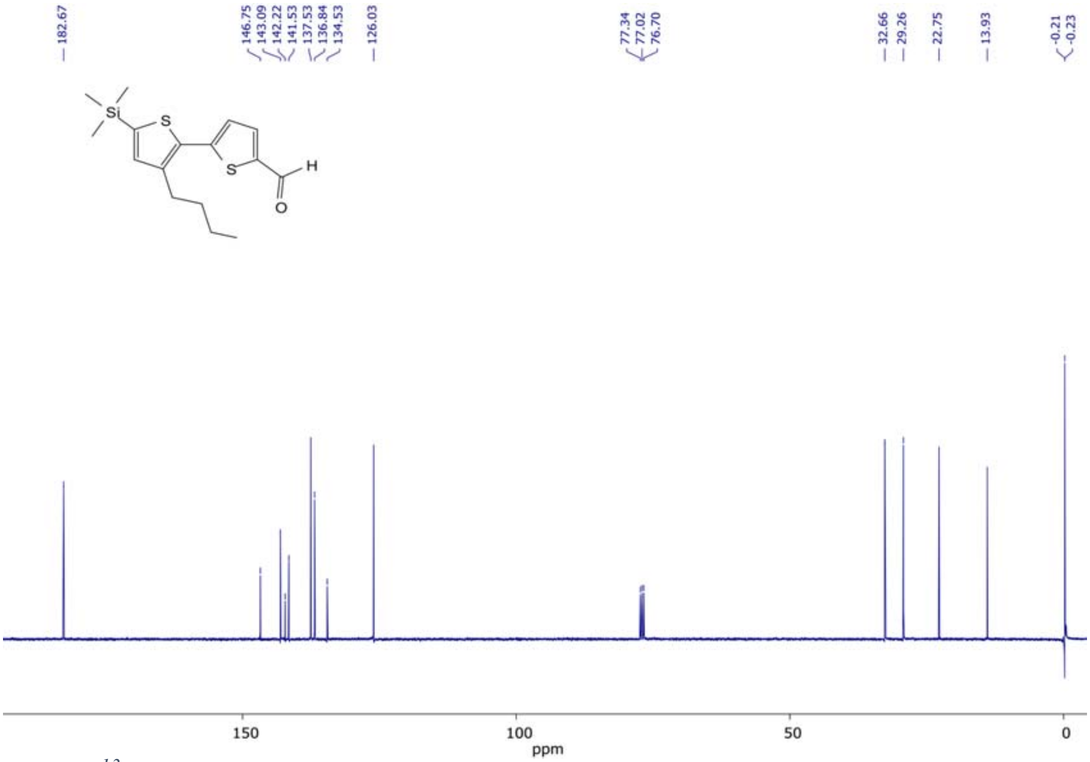

Figure S3.14. [13]*C-NMR of 3'-butyl-5'-(trimethylsilyl)-[2,2'-bithiophene]-5-carbaldehyde (4a)*

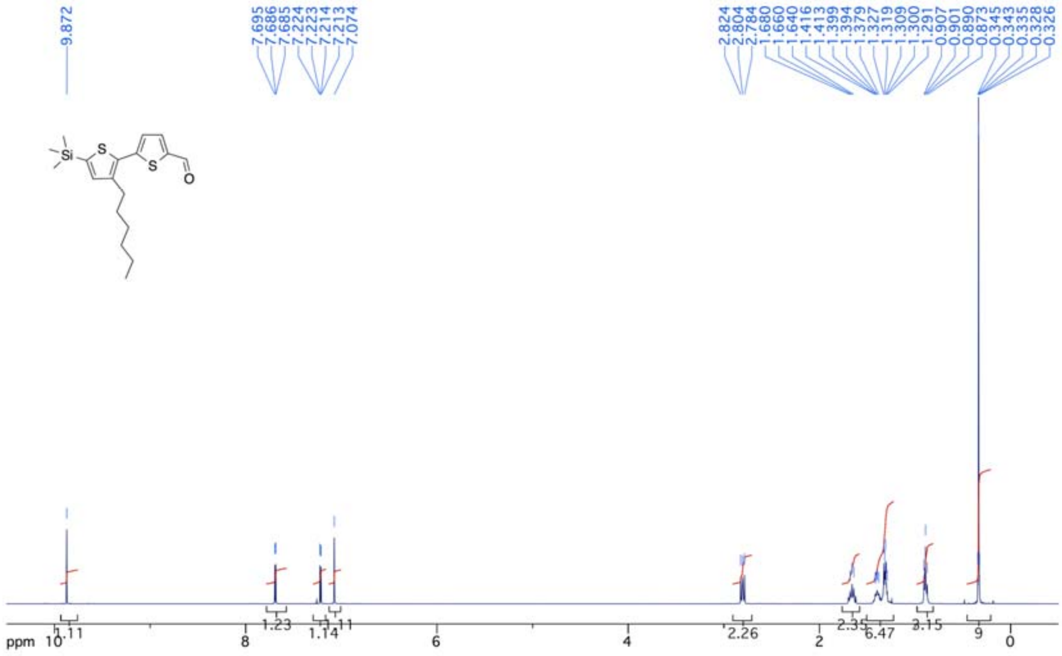

Figure S3.15. ^{1}H-NMR of 3'-hexyl-5'-(trimethylsilyl)-[2,2'-bithiophene]-5-carbaldehyde (4b)

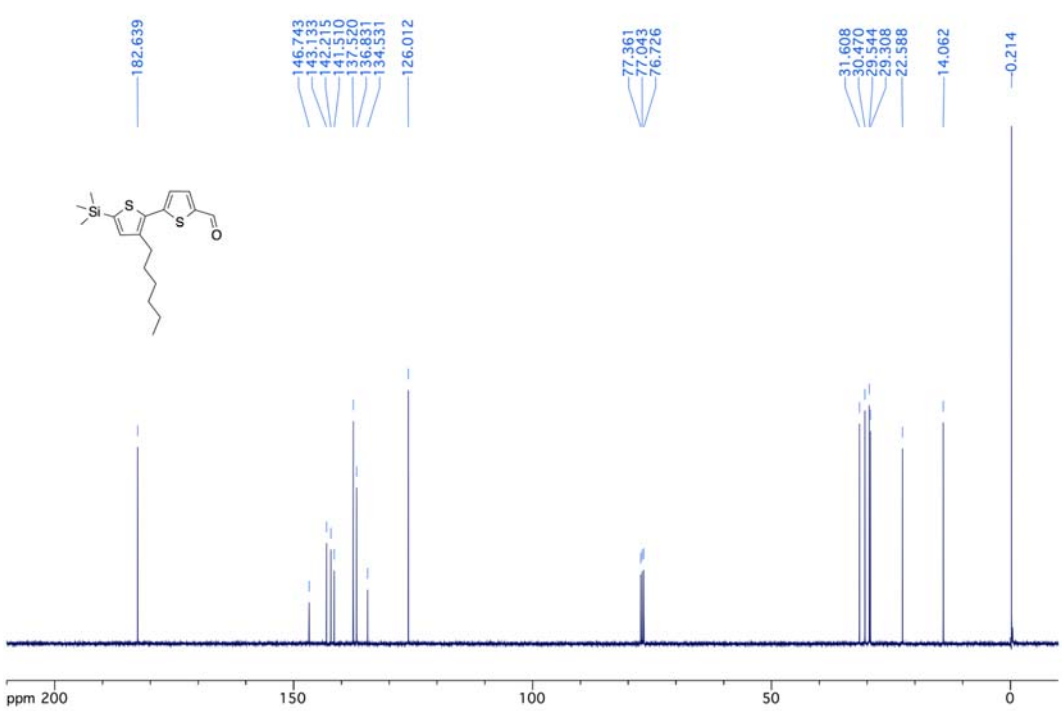

Figure S3.16. ^{13}C-NMR of 3'-hexyl-5'-(trimethylsilyl)-[2,2'-bithiophene]-5-carbaldehyde (4b)

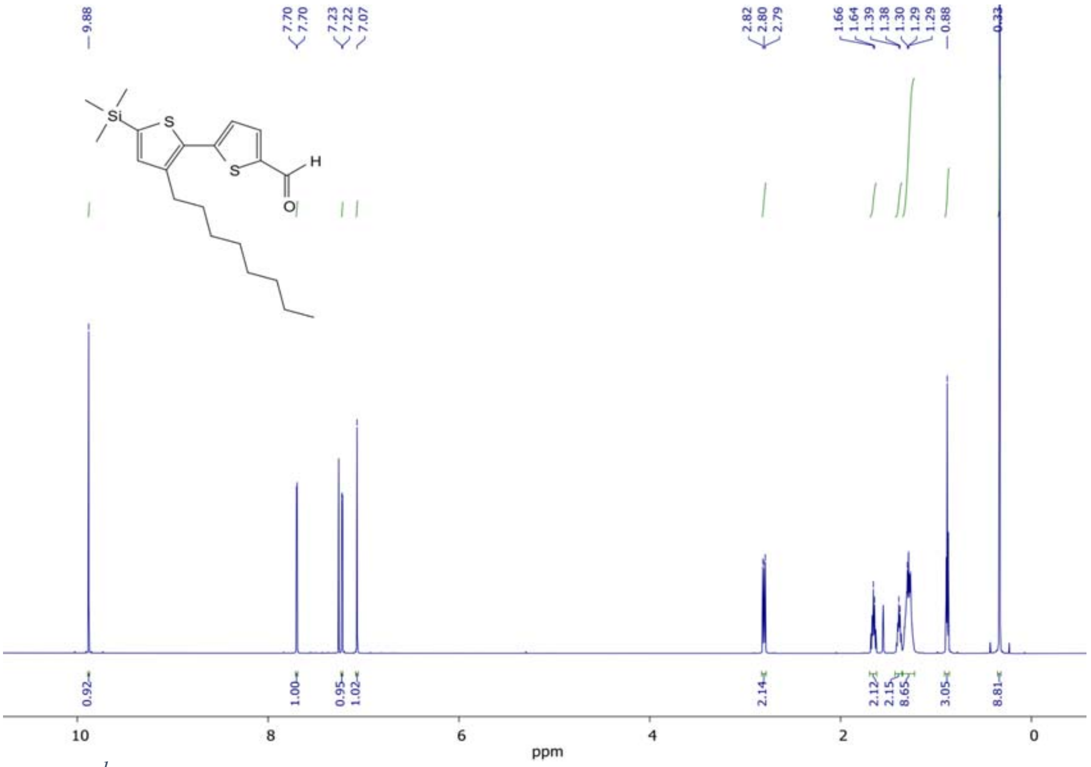

Figure S3.17. *^{1}H-NMR of 3'-octyl-5'-(trimethylsilyl)-[2,2'-bithiophene]-5-carbaldehyde (4c)*

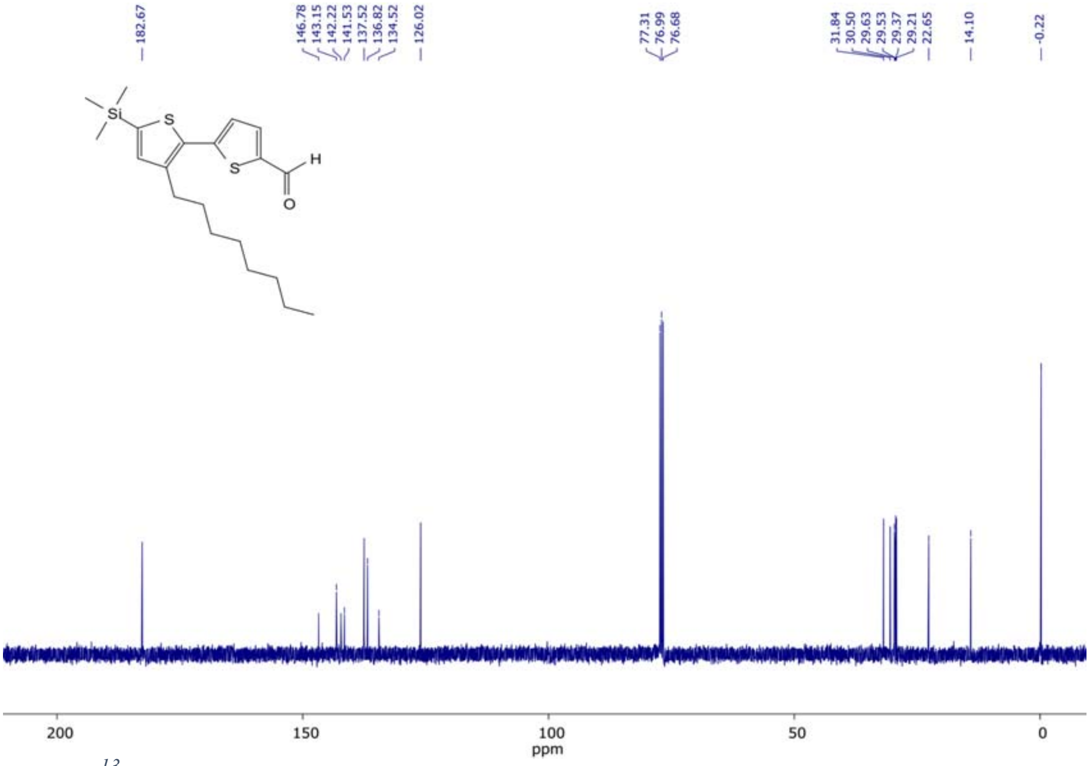

Figure S3.18. *^{13}C-NMR of 3'-butyl-5'-(trimethylsilyl)-[2,2'-bithiophene]-5-carbaldehyde (4c)*

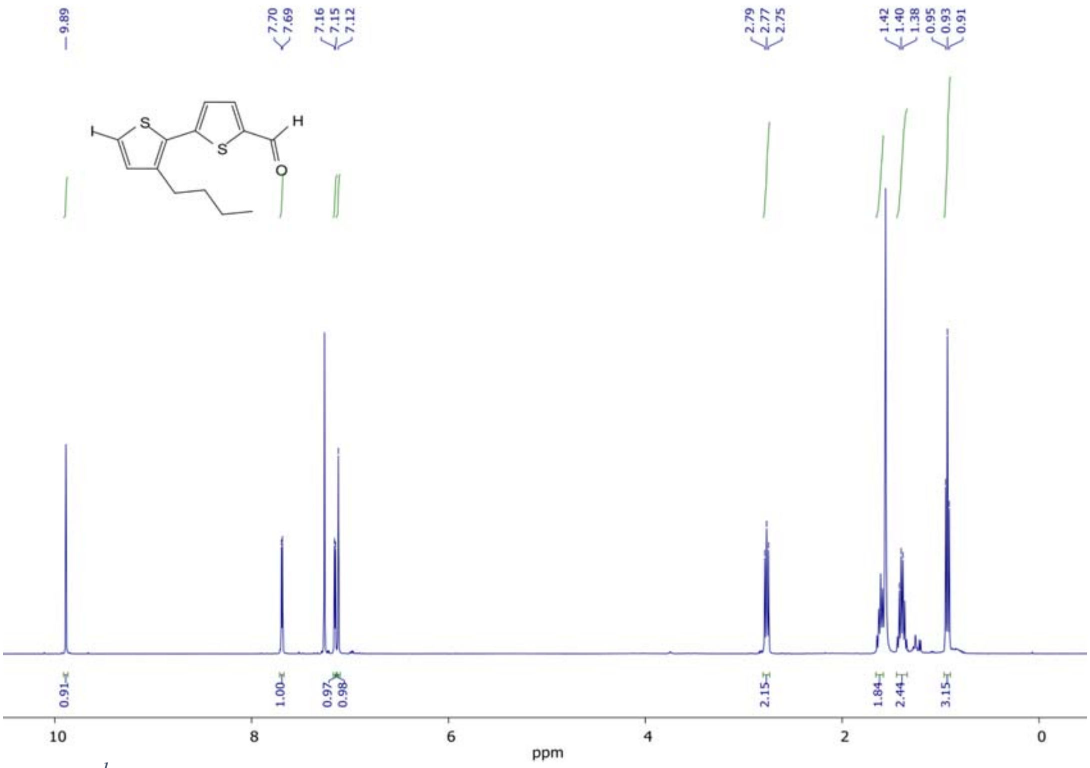

Figure S3.19. *^{1}H-NMR of 3'-butyl-5'-iodo-[2,2'-bithiophene]-5-carbaldehyde (5a)*

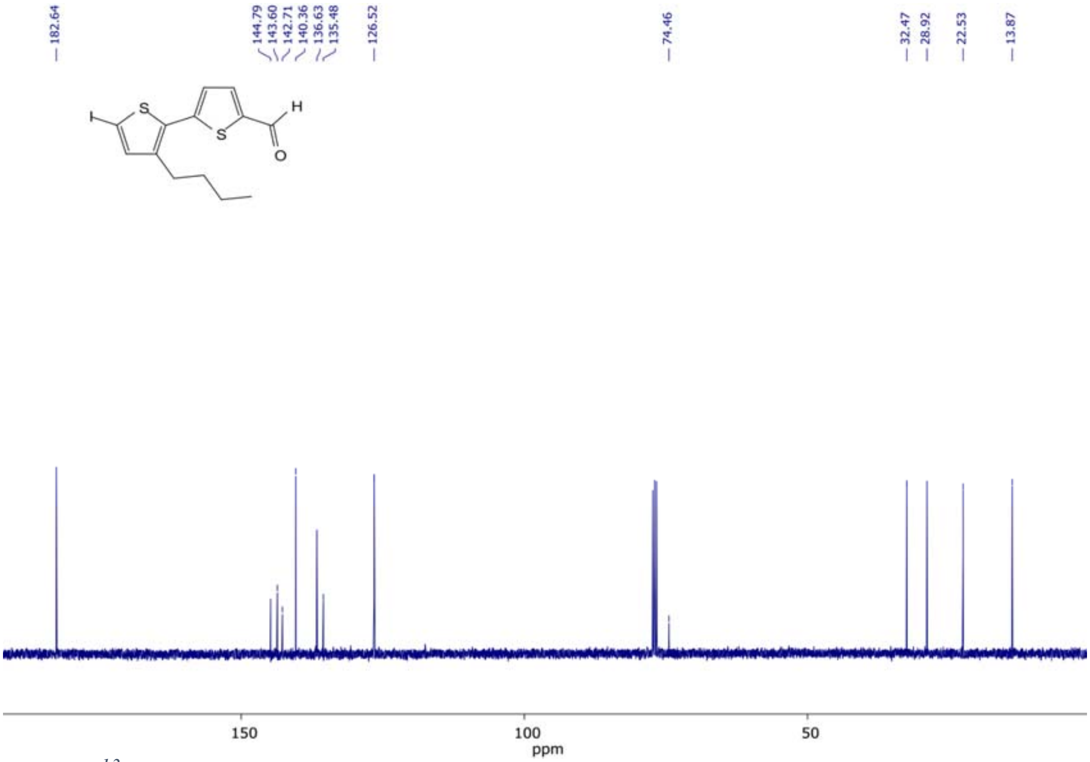

Figure S3.20. *^{13}C-NMR of 3'-butyl-5'-iodo-[2,2'-bithiophene]-5-carbaldehyde (5a)*

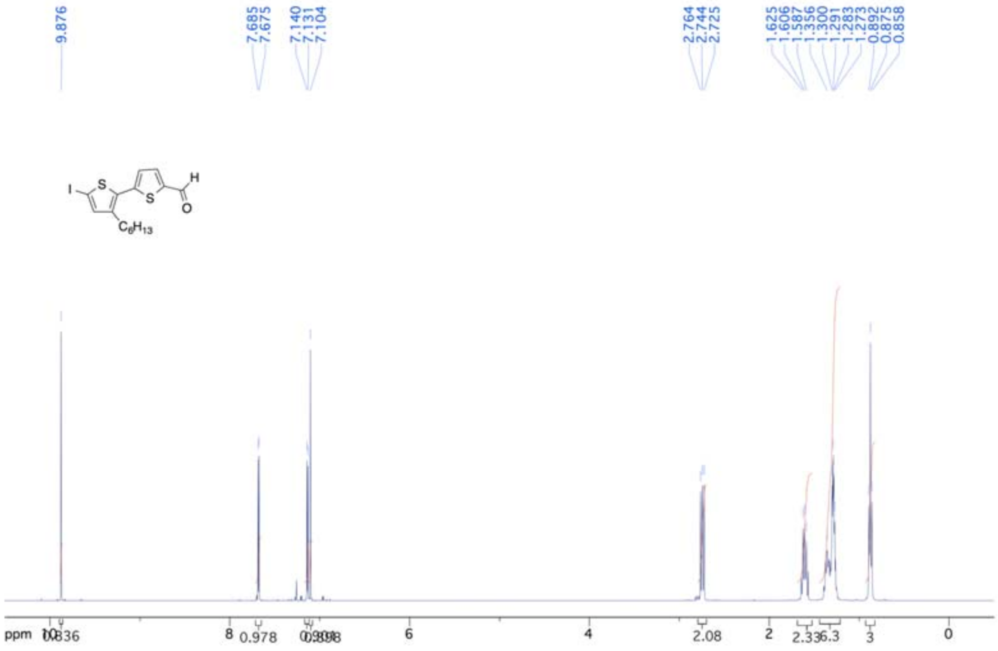

Figure S3.21. [1]*H-NMR of 3'-hexyl-5'-iodo-[2,2'-bithiophene]-5-carbaldehyde (5b)*

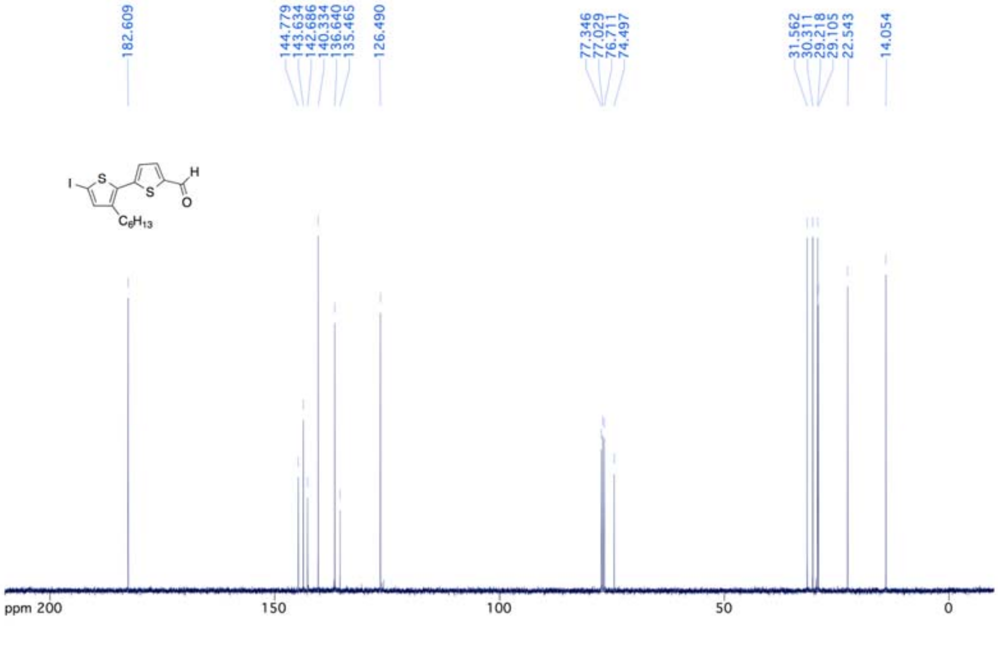

Figure S3.22. [13]*C-NMR of 3'-hexyl-5'-iodo-[2,2'-bithiophene]-5-carbaldehyde (5b)*

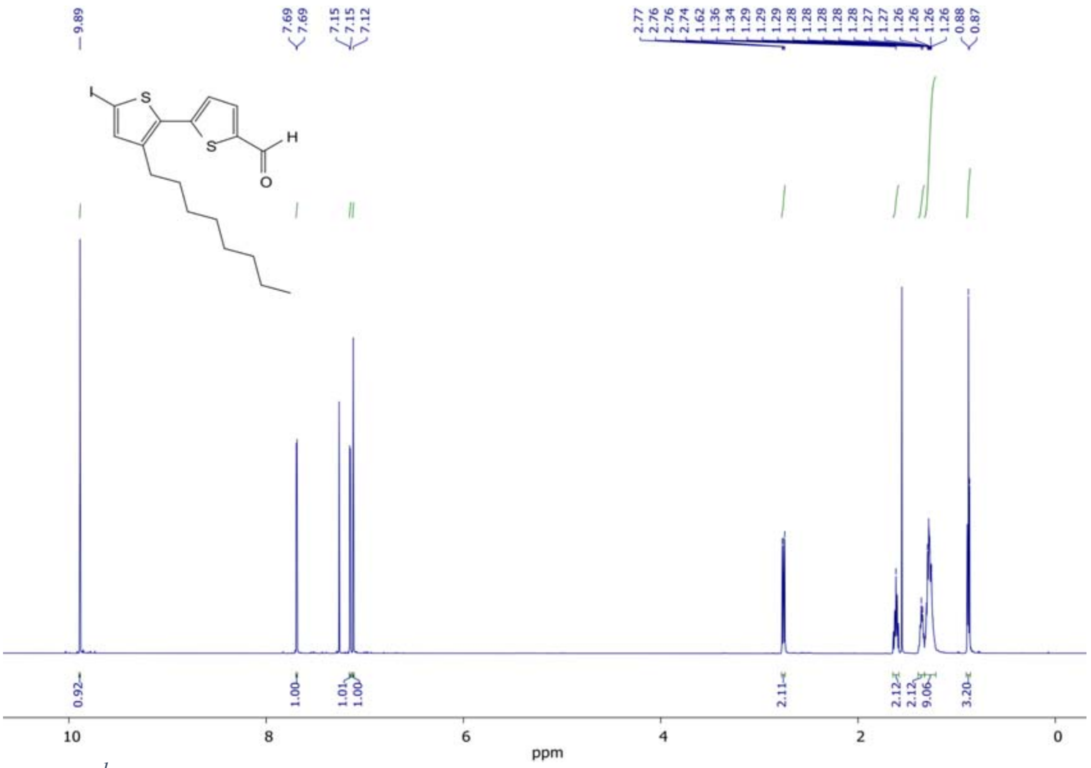

Figure S3.23. [1]*H-NMR of 3'-octyl-5'-iodo-[2,2'-bithiophene]-5-carbaldehyde (5c)*

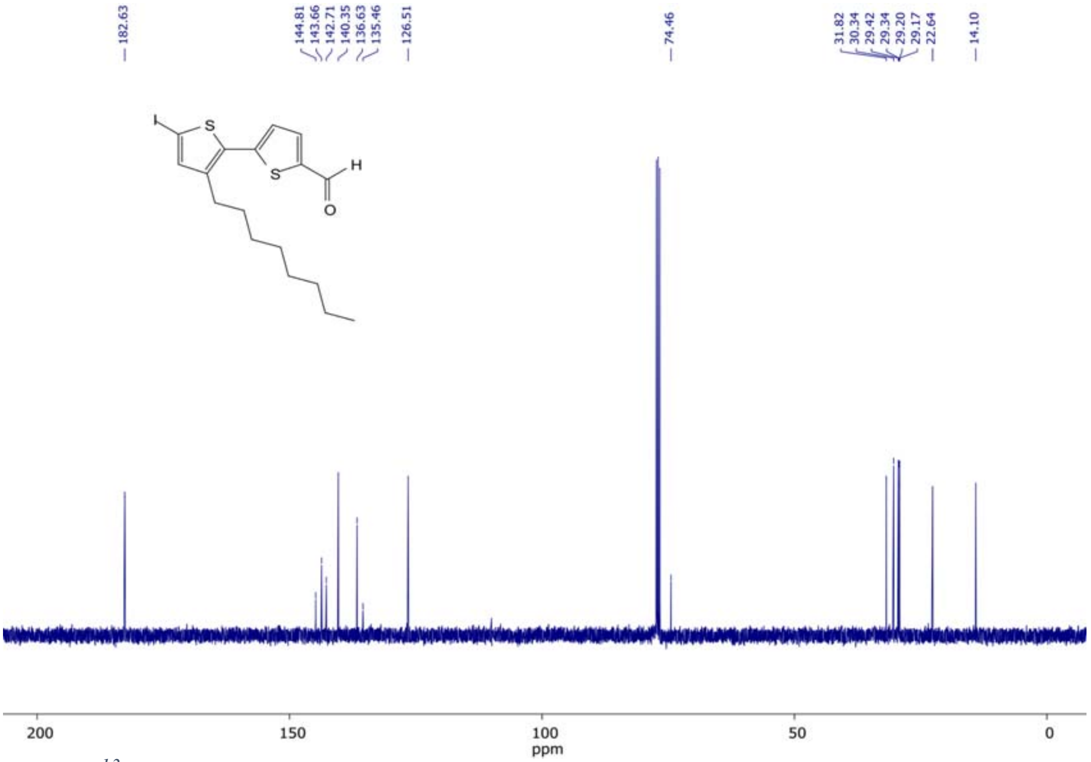

Figure S3.24. [13]*C-NMR of 3'-octyl-5'-iodo-[2,2'-bithiophene]-5-carbaldehyde (5c)*

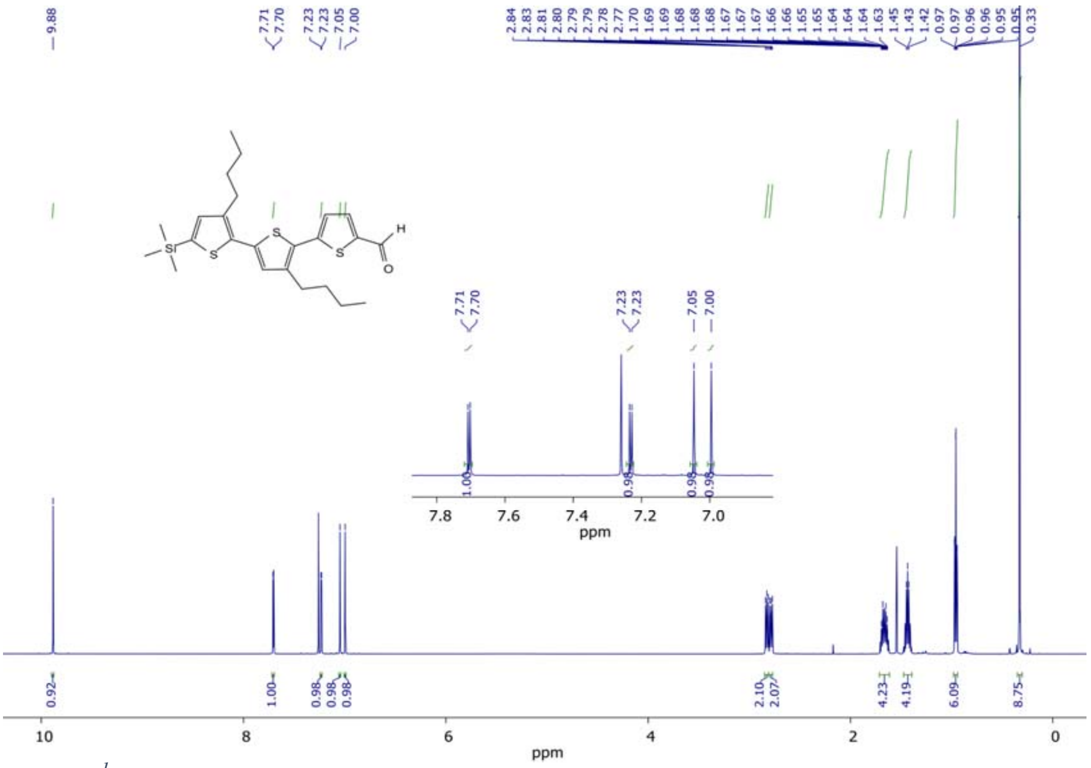

Figure S3.25. [1]*H-NMR of 3',3''-dibutyl-5''-(trimethylsilyl)-[2,2':5',2''-terthiophene]-5-carbaldehyde (6a)*

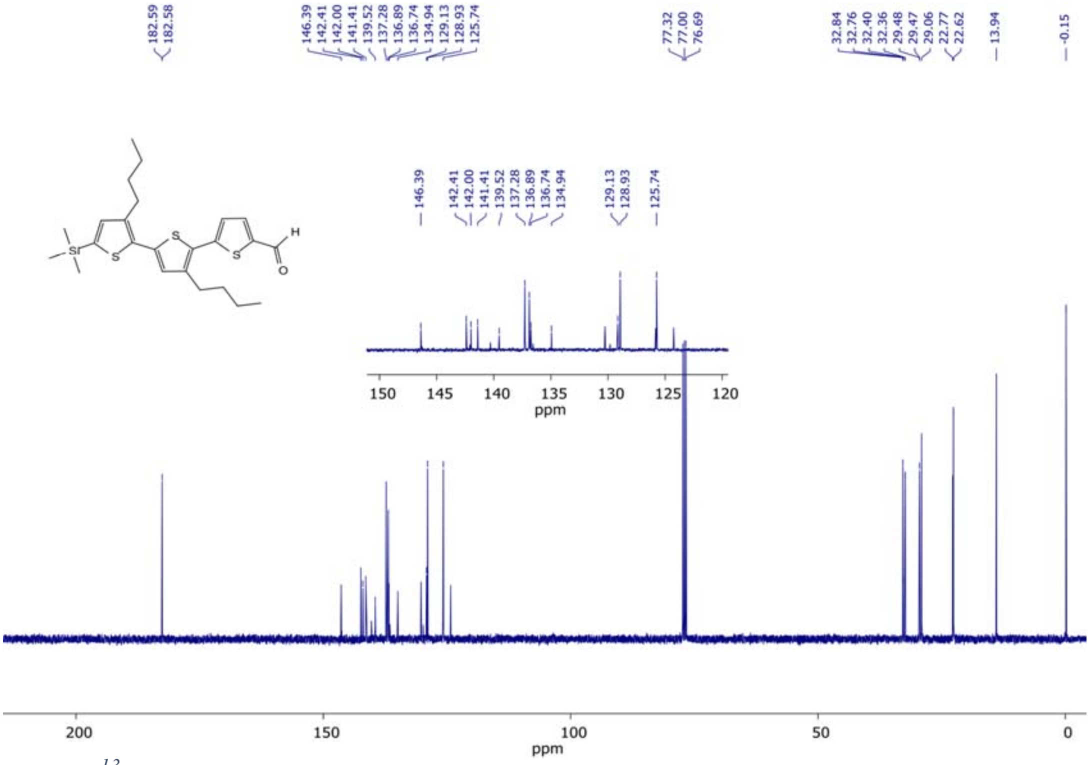

Figure S3.26. [13]*C-NMR of 3',3''-dibutyl-5''-(trimethylsilyl)-[2,2':5',2''-terthiophene]-5-carbaldehyde (6a)*

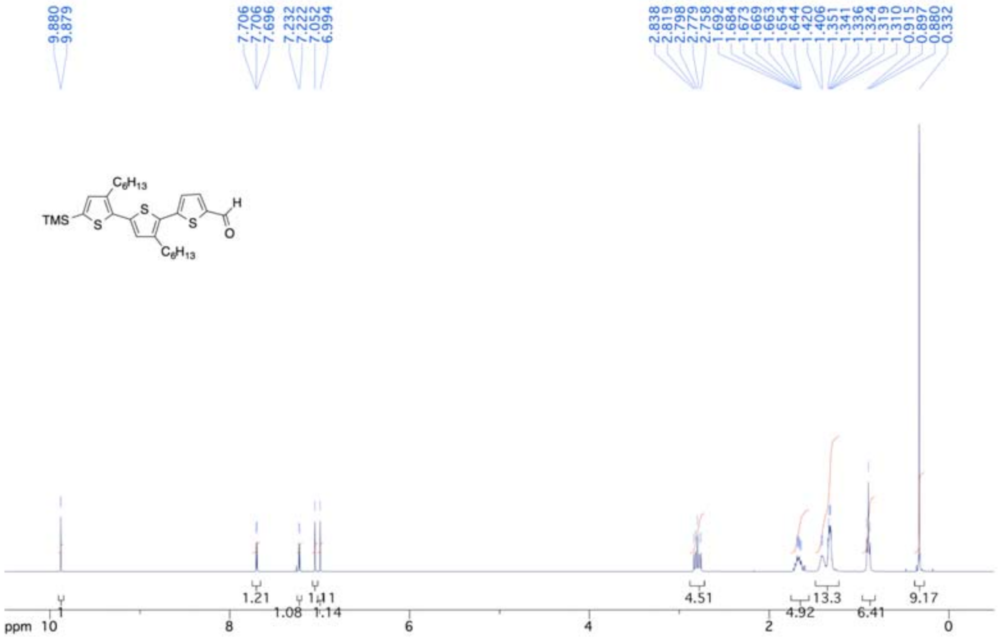

Figure S3.27. ^{1}*H-NMR of 3',3''-dihexyl-5''-(trimethylsilyl)-[2,2':5',2''-terthiophene]-5-carbaldehyde (6b)*

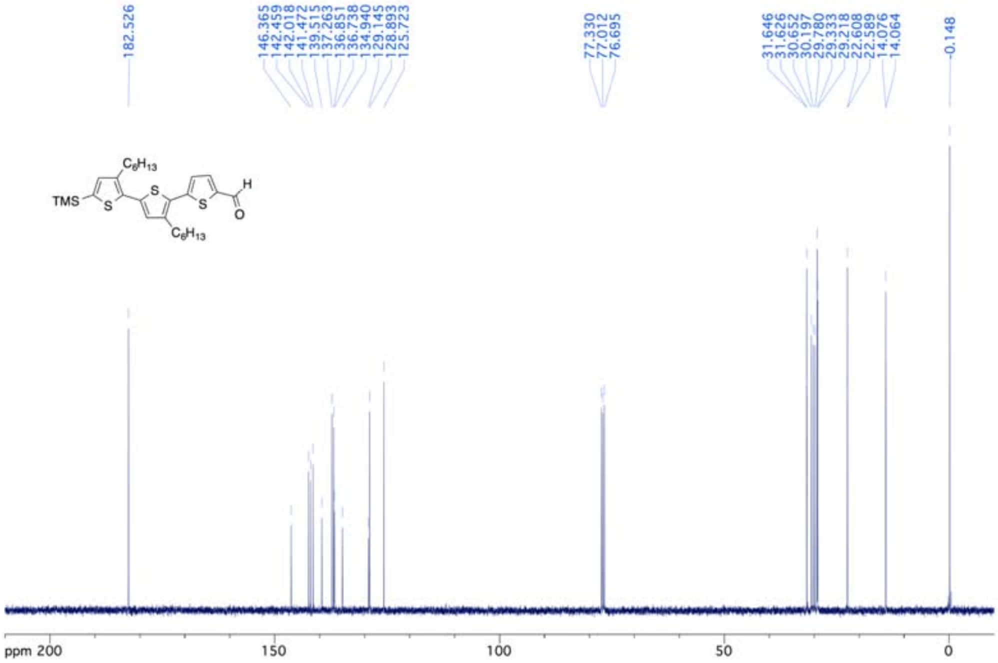

Figure S3.28. ^{13}*C-NMR of 3',3''-dihexyl-5''-(trimethylsilyl)-[2,2':5',2''-terthiophene]-5-carbaldehyde (6b)*

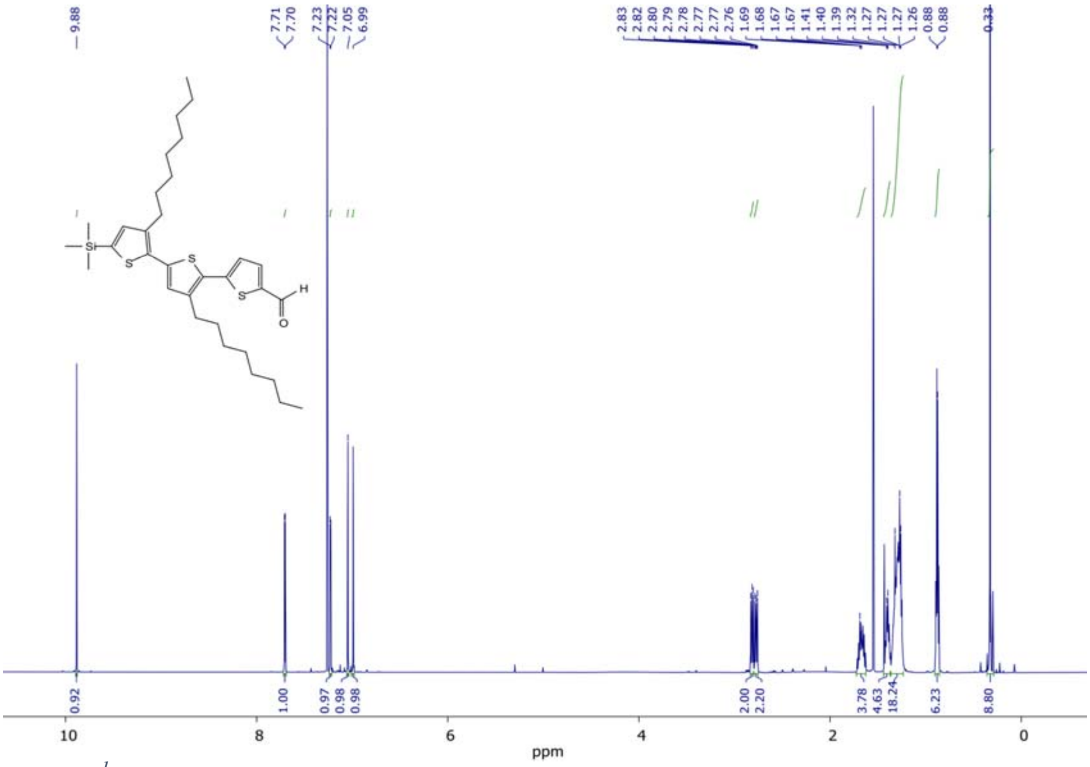

Figure S3.29. *^{1}H-NMR of 3',3''-dioctyl-5''-(trimethylsilyl)-[2,2':5',2''-terthiophene]-5-carbaldehyde (6c)*

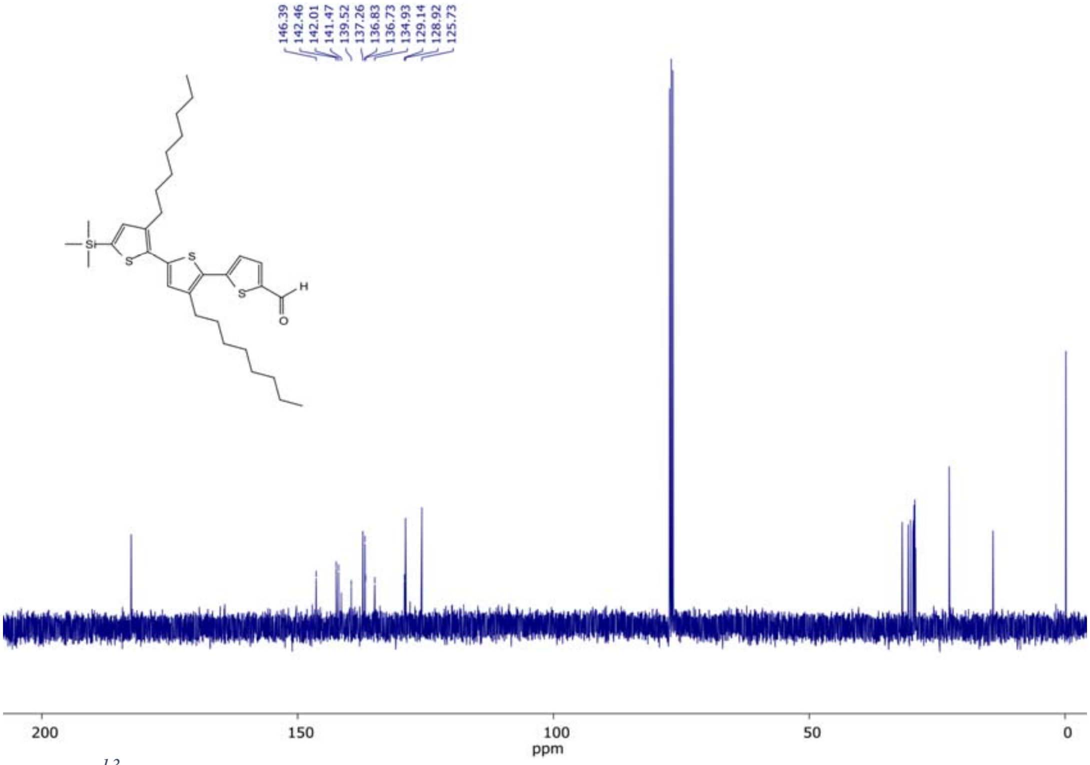

Figure S3.30. *^{13}C-NMR of 3',3''-dioctyl-5''-(trimethylsilyl)-[2,2':5',2''-terthiophene]-5-carbaldehyde (6c)*

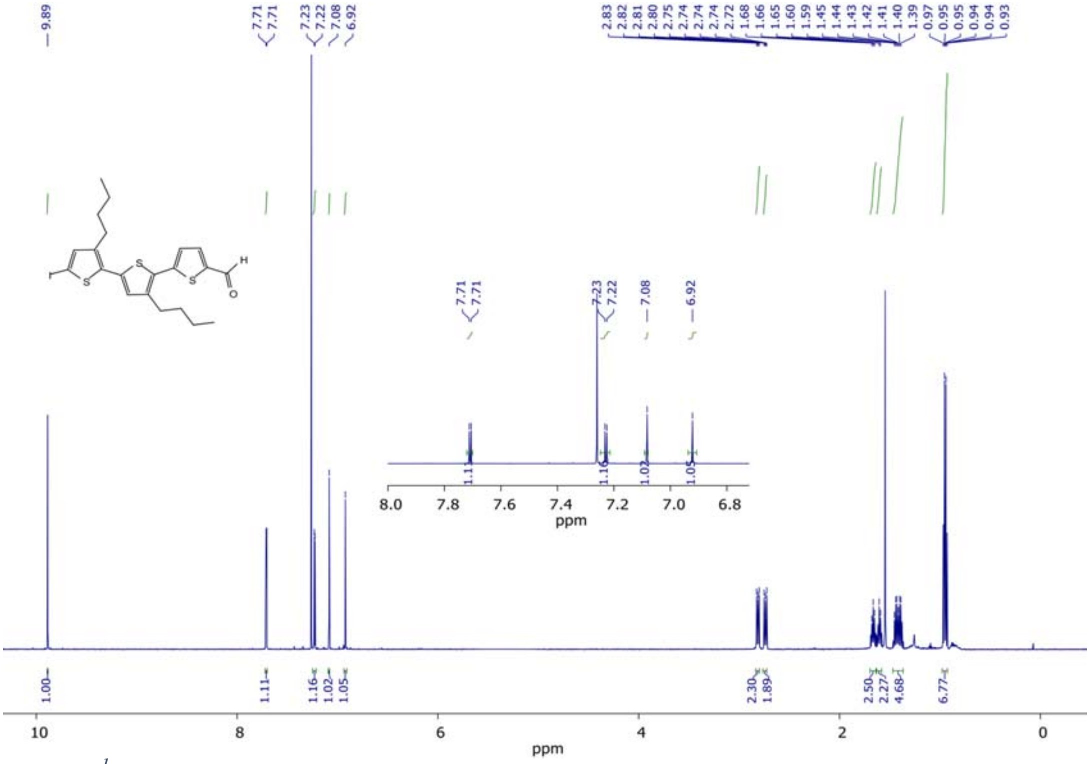

Figure S3.31. [1]*H-NMR of 3',3''-dibutyl-5''-iodo-[2,2':5',2''-terthiophene]-5-carbaldehyde (7a)*

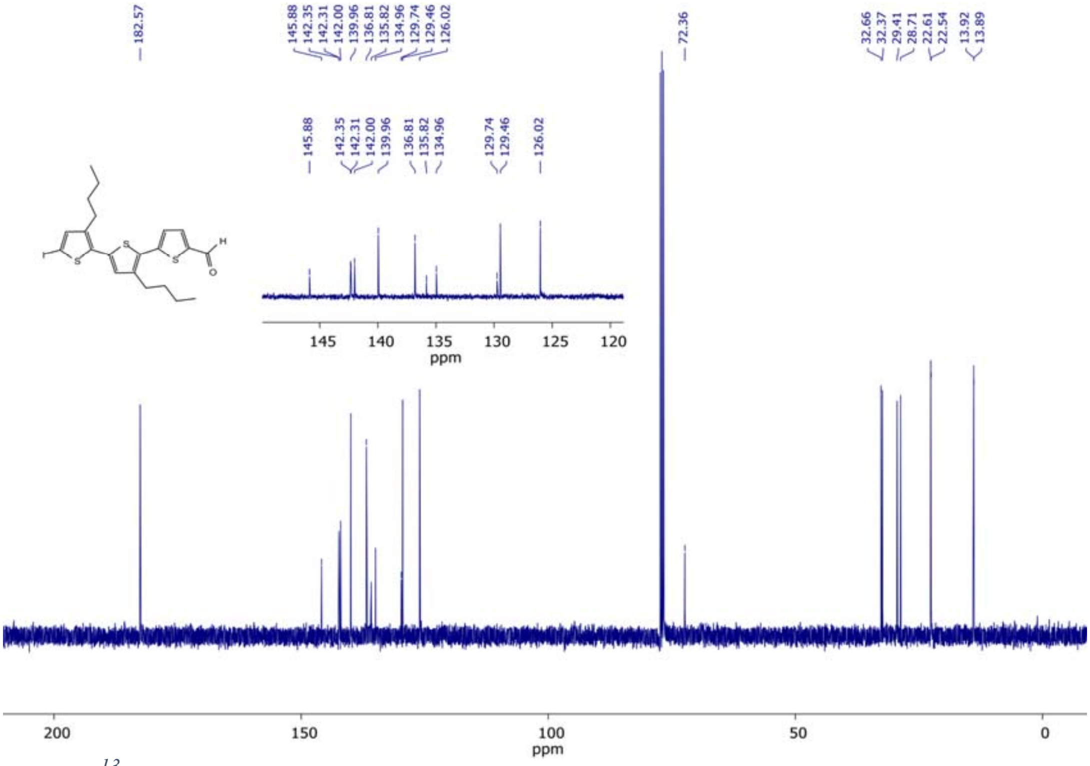

Figure S3.32. [13]*C-NMR of 3',3''-dibutyl-5''-iodo-[2,2':5',2''-terthiophene]-5-carbaldehyde (7a)*

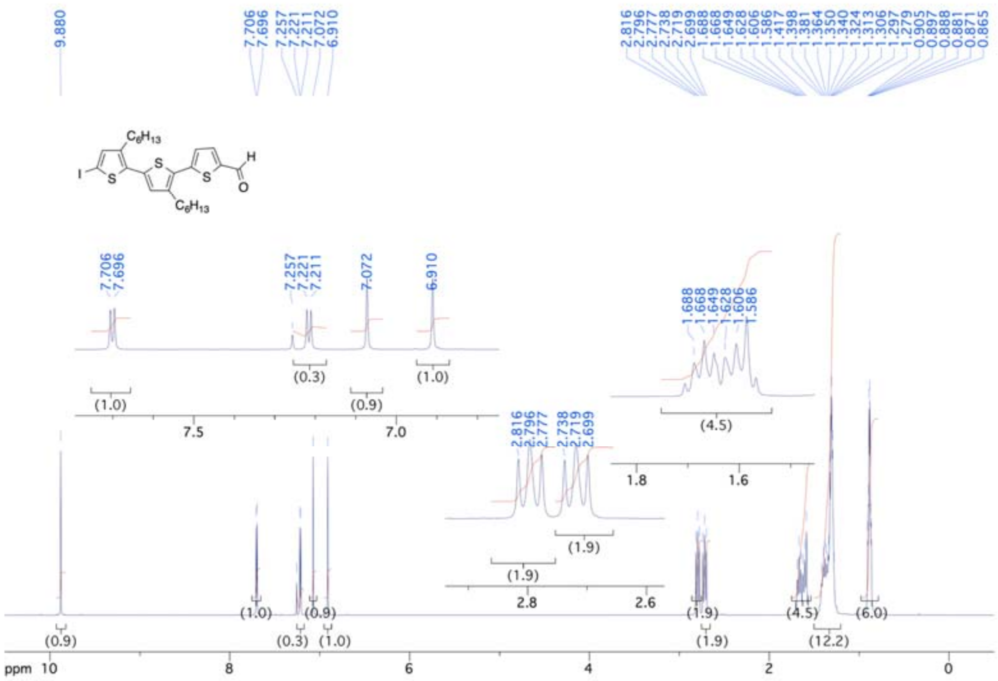

Figure S3.33. [1]*H-NMR of 3',3''-dihexyl-5''-iodo-[2,2':5',2''-terthiophene]-5-carbaldehyde (7b)*

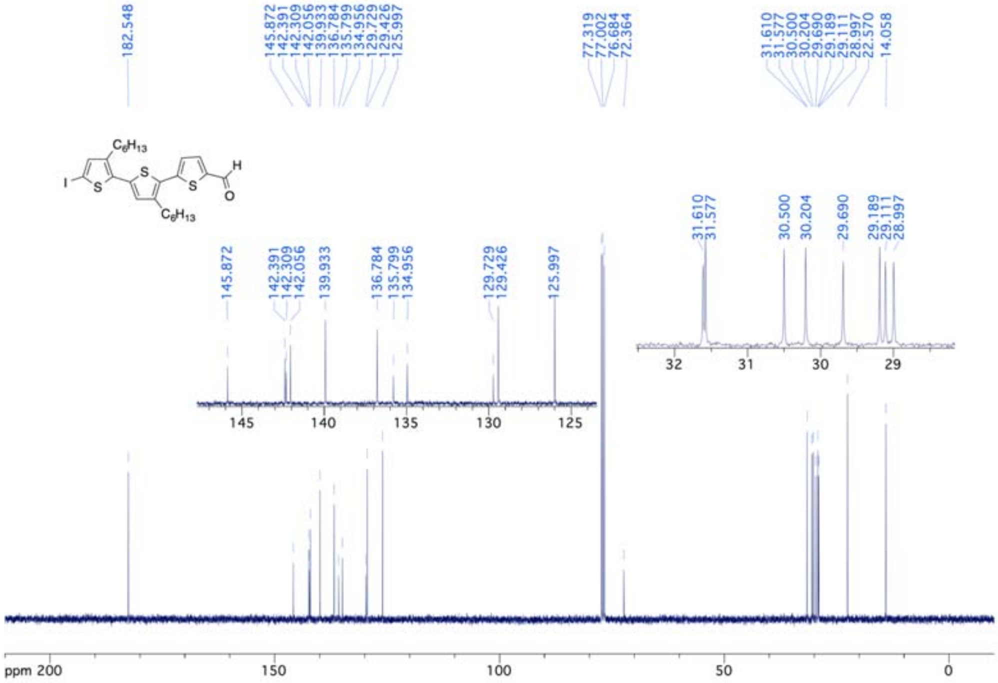

Figure S3.34. [13]*C-NMR of 3',3''-dihexyl-5''-iodo-[2,2':5',2''-terthiophene]-5-carbaldehyde (7b)*

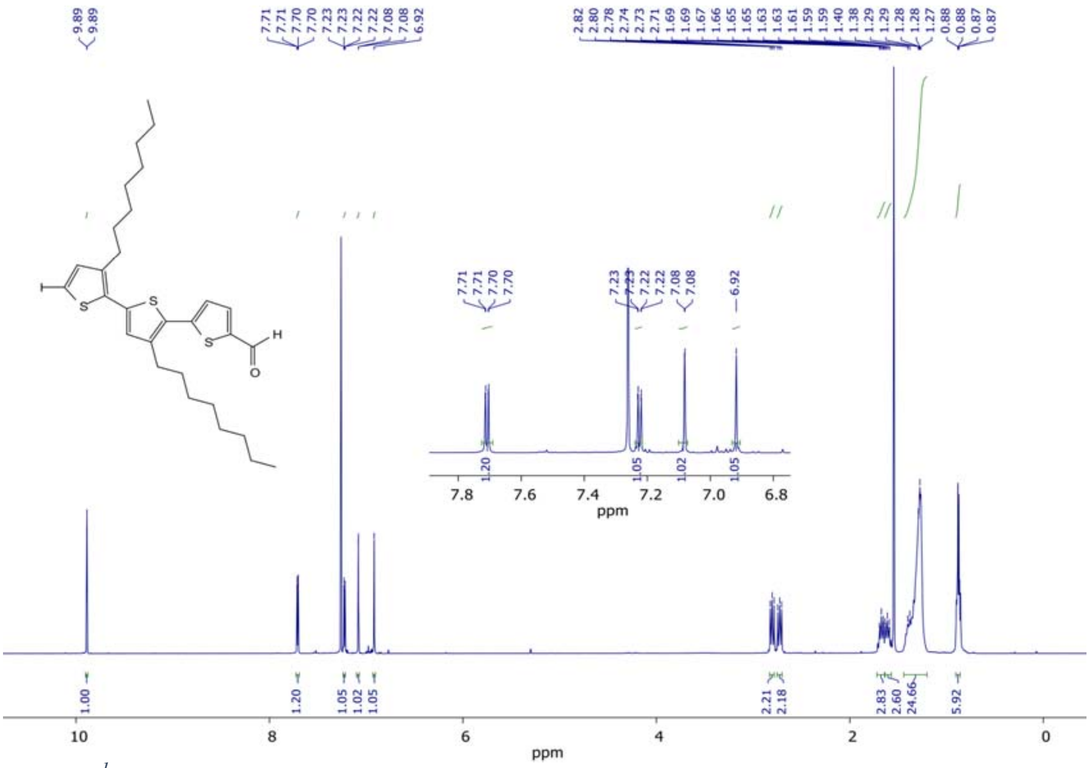

Figure S3.35. *^{1}H-NMR of 3',3''-dioctyl-5''-iodo-[2,2':5',2''-terthiophene]-5-carbaldehyde (7c)*

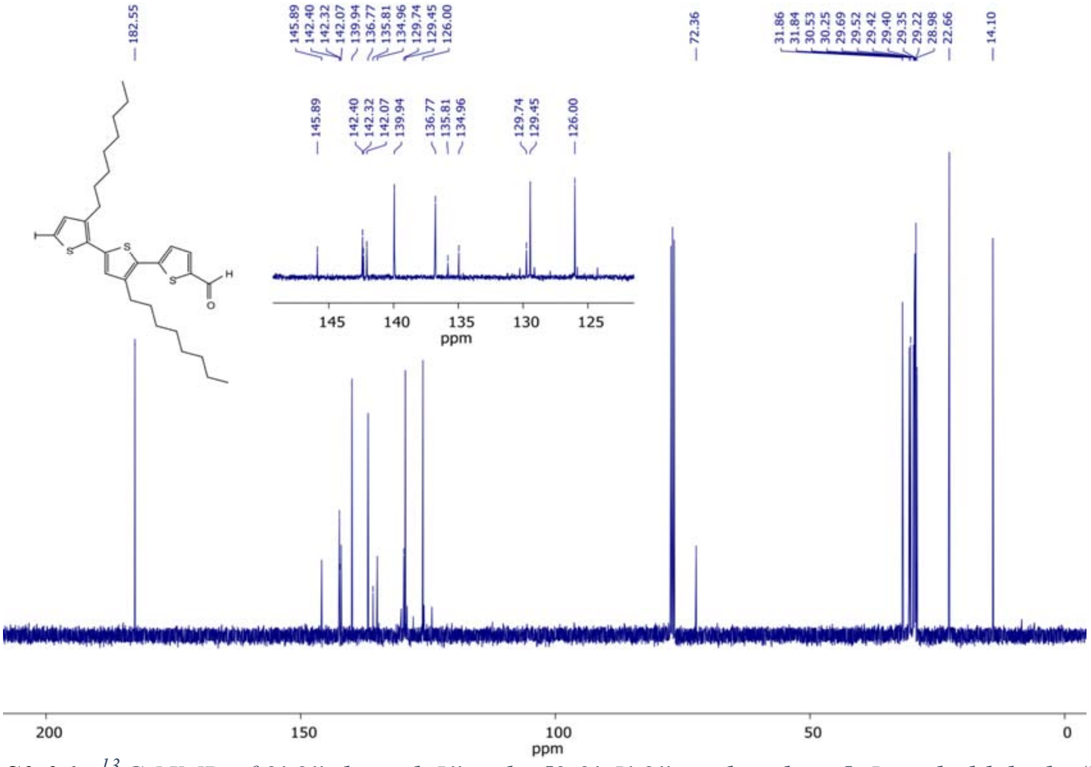

Figure S3.36. *^{13}C-NMR of 3',3''-dioctyl-5''-iodo-[2,2':5',2''-terthiophene]-5-carbaldehyde (7c)*

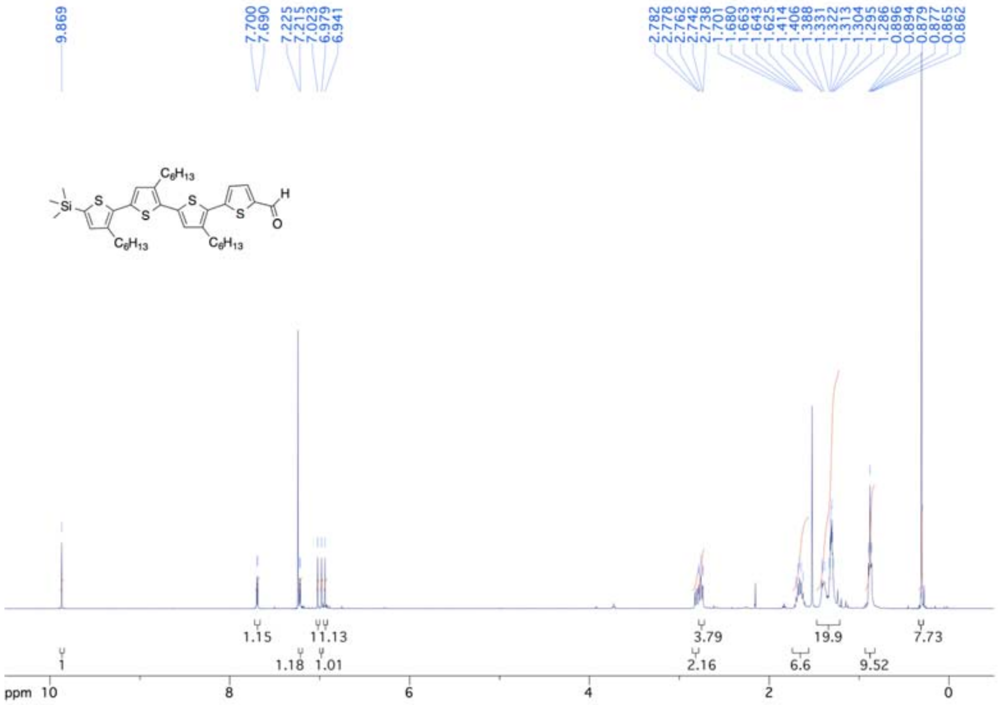

Figure S3.37. *^{1}H-NMR of 3',3'',3'''-trihexyl-5'''-(trimethylsilyl)-[2,2':5',2'':5'',2'''-quaterthiophene]-5-carbaldehyde (8b)*

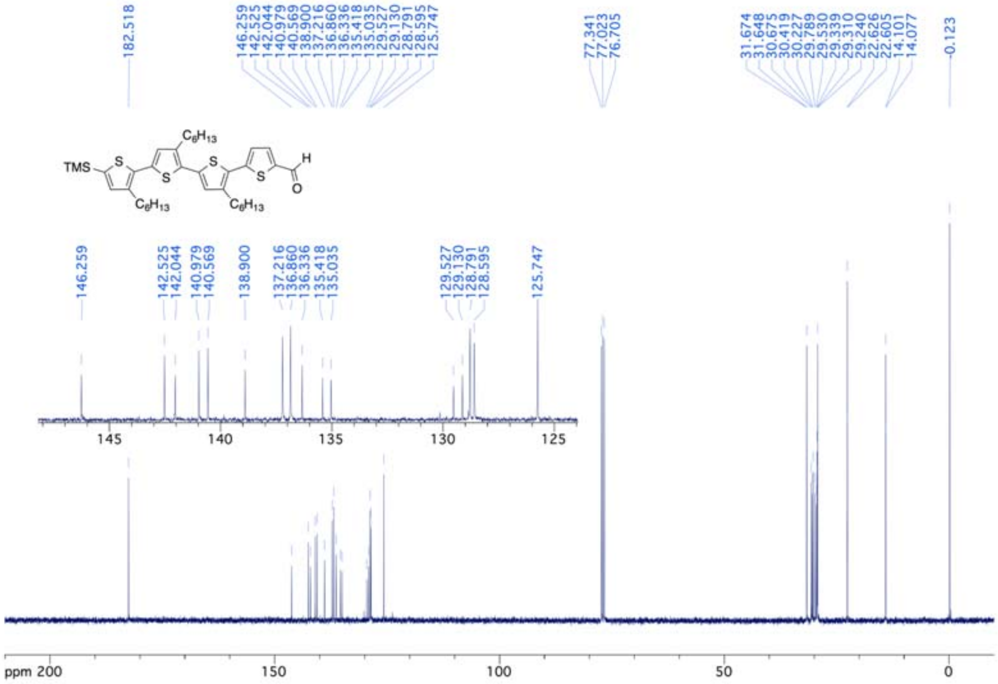

Figure S3.38. *^{13}C-NMR of 3',3'',3'''-trihexyl-5'''-(trimethylsilyl)-[2,2':5',2'':5'',2'''-quaterthiophene]-5-carbaldehyde (8b)*

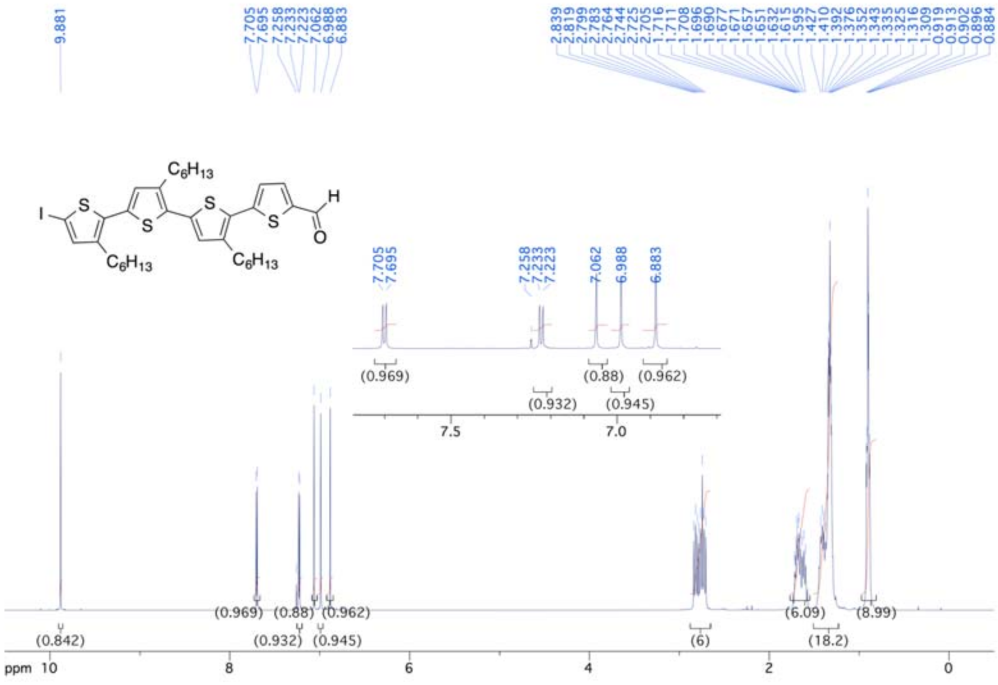

Figure S3.39. ^{1}*H-NMR of 3',3'',3'''-trihexyl-5'''-iodo-[2,2':5',2'':5'',2'''-quaterthiophene]-5-carbaldehyde (9b)*

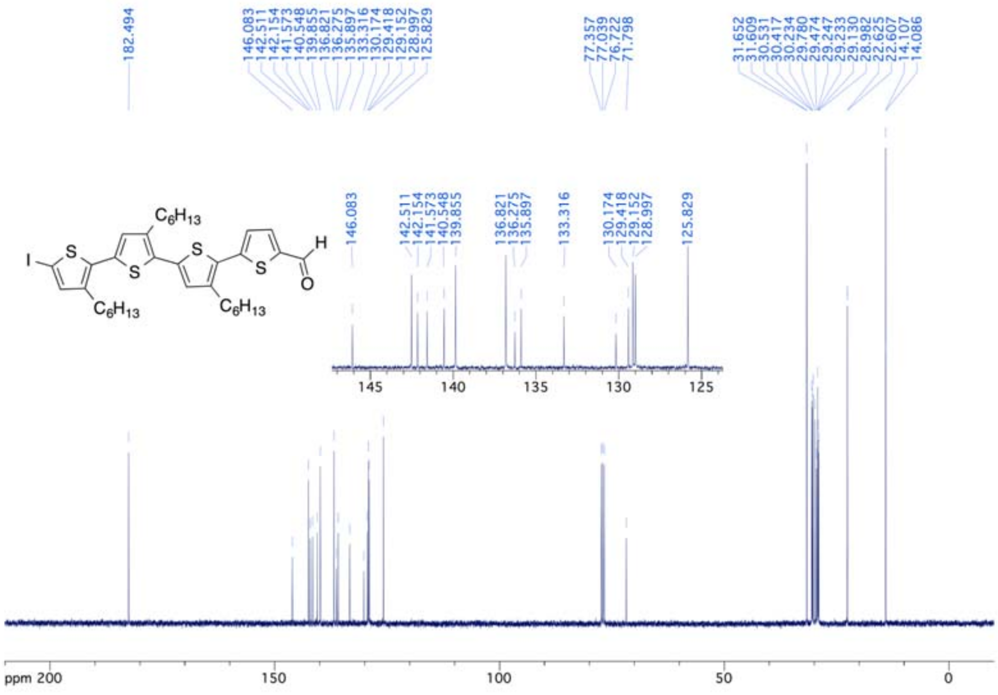

Figure S3.40. ^{13}*C-NMR of 3',3'',3'''-trihexyl-5'''-iodo-[2,2':5',2'':5'',2'''-quaterthiophene]-5-carbaldehyde (9b)*

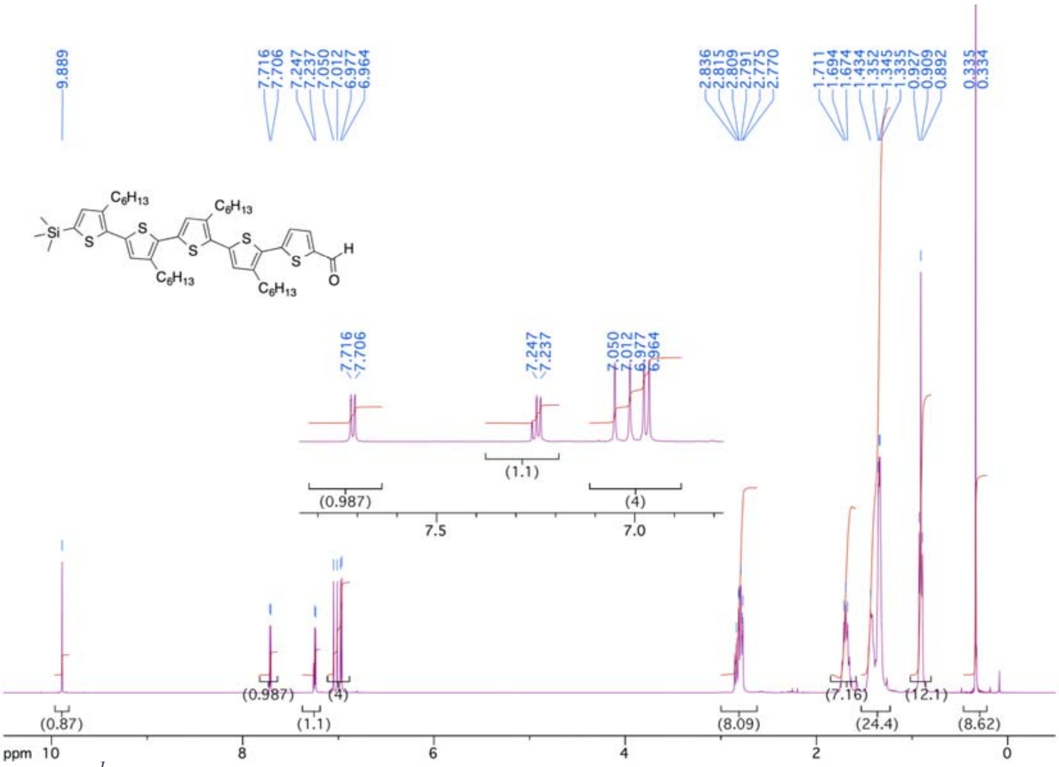

Figure S3.41. [1]*H-NMR of 3',3'',3''',3''''-tetrahexyl-5''''-(trimethylsilyl)-[2,2':5',2'':5'',2''':5''',2''''-quinquethiophene]-5-carbaldehyde (10b)*

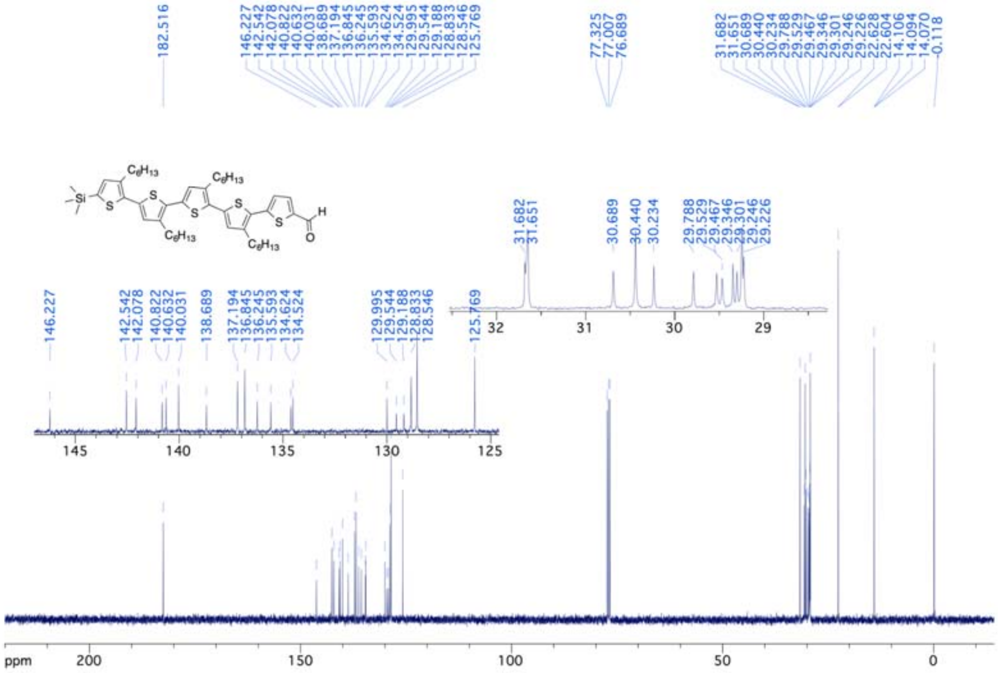

Figure S3.42. [13]*C-NMR of 3',3'',3''',3''''-tetrahexyl-5''''-(trimethylsilyl)-[2,2':5',2'':5'',2''':5''',2''''-quinquethiophene]-5-carbaldehyde (10b)*

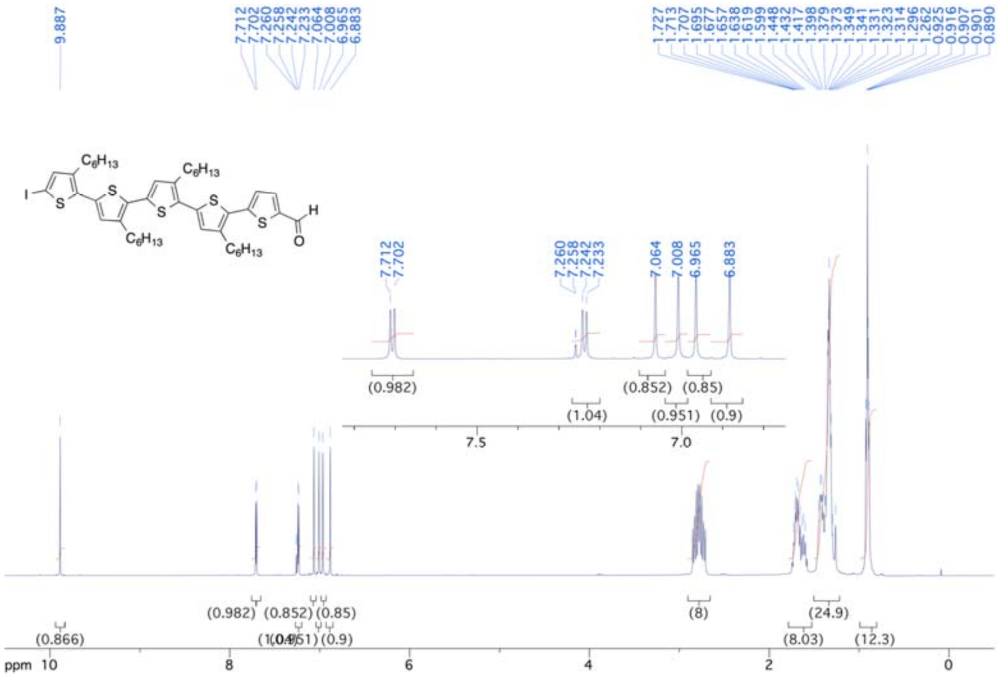

Figure S3.43. *^{1}H-NMR of 3',3'',3''',3''''-tetrahexyl-5''''-iodo-[2,2':5',2'':5'',2''': 5''',2''''-quinquethiophene]-5-carbaldehyde (11b)*

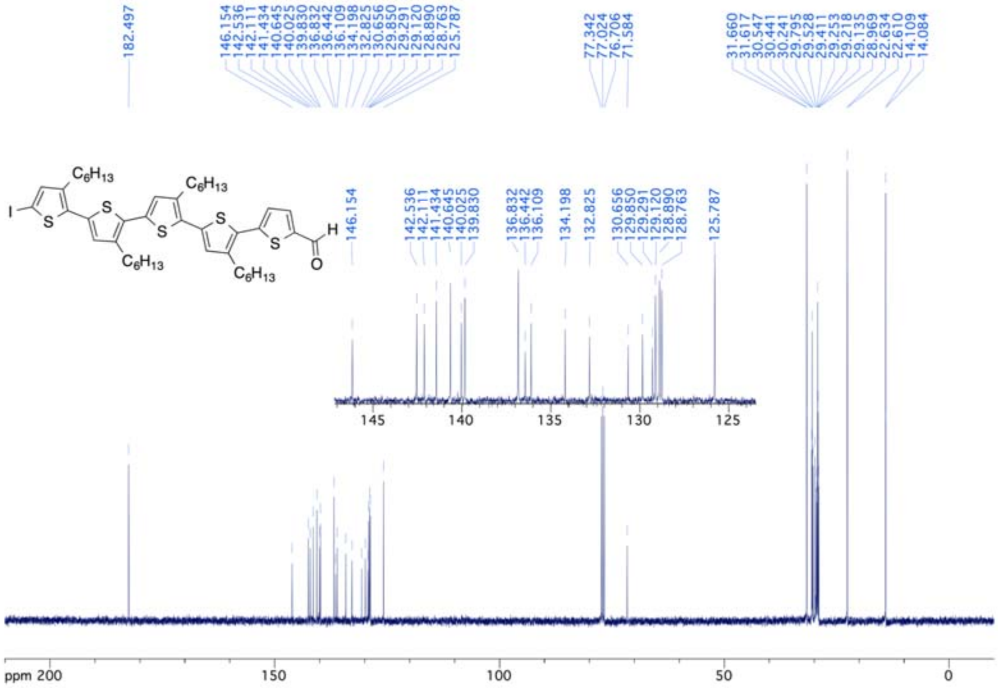

Figure S3.44. *^{13}C-NMR of 3',3'',3''',3''''-tetrahexyl-5''''-iodo-[2,2':5',2'':5'',2''': 5''',2''''-quinquethiophene]-5-carbaldehyde (11b)*

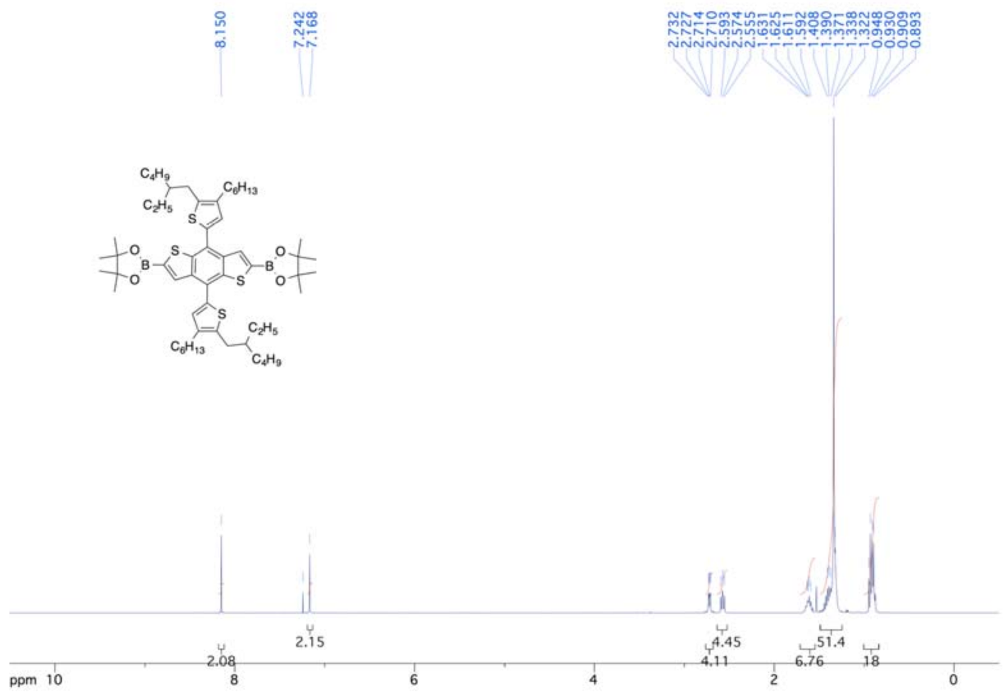

Figure S3.45. [1]*H-NMR of 2,2'-(4,8-bis(5-(2-ethylhexyl)-4-hexylthiophen-2-yl)benzo[1,2-b:4,5-b']dithiophene-2,6-diyl)bis(4,4,5,5-tetramethyl-1,3,2-dioxaborolane) (13)*

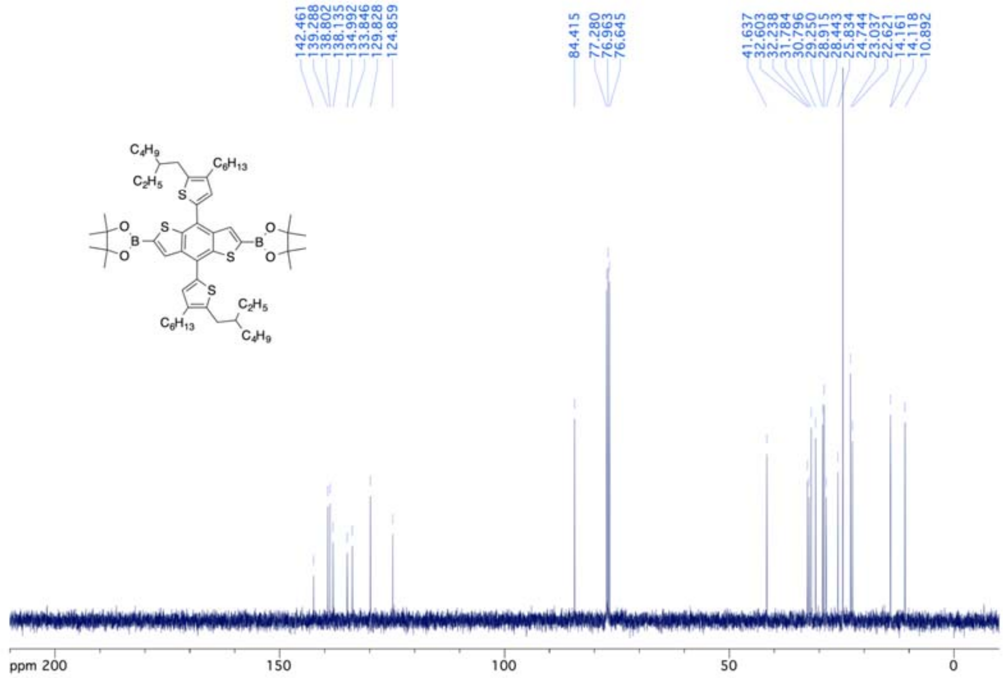

Figure S3.46. [13]*C-NMR of 2,2'-(4,8-bis(5-(2-ethylhexyl)-4-hexylthiophen-2-yl)benzo[1,2-b:4,5-b']dithiophene-2,6-diyl)bis(4,4,5,5-tetramethyl-1,3,2-dioxaborolane) (13)*

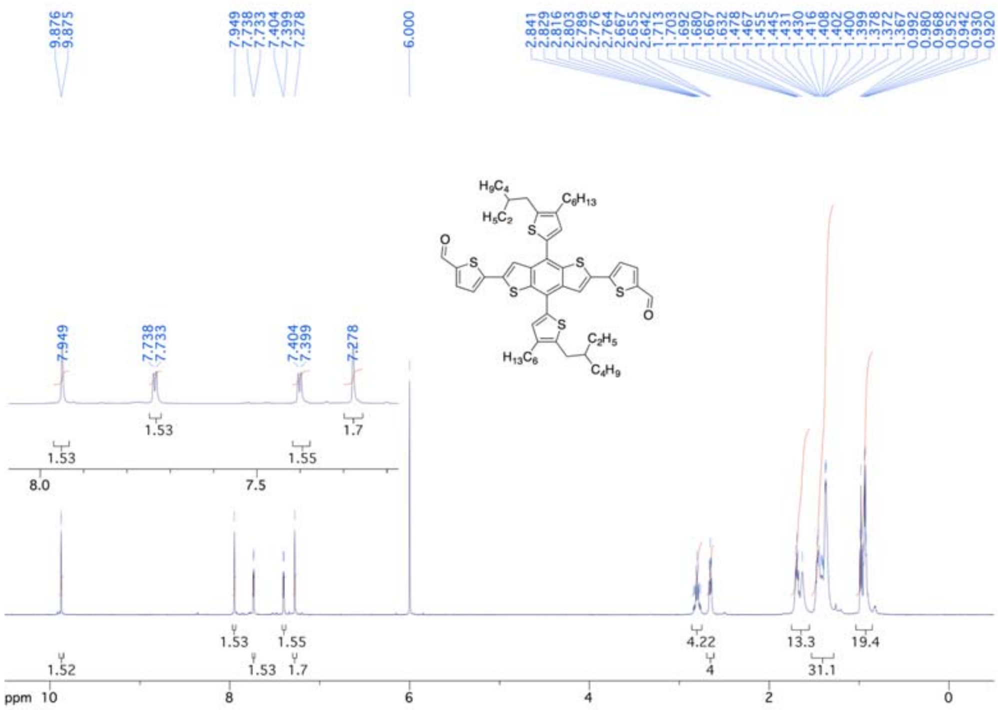

Figure S3.47. [1]*H-NMR of 5,5'-(4,8-bis(5-(2-ethylhexyl)-4-hexylthiophen-2-yl)benzo[1,2-b:4,5-b']dithiophene-2,6-diyl)bis(thiophene-2-carbaldehyde)) (BM-dialdehyde)*

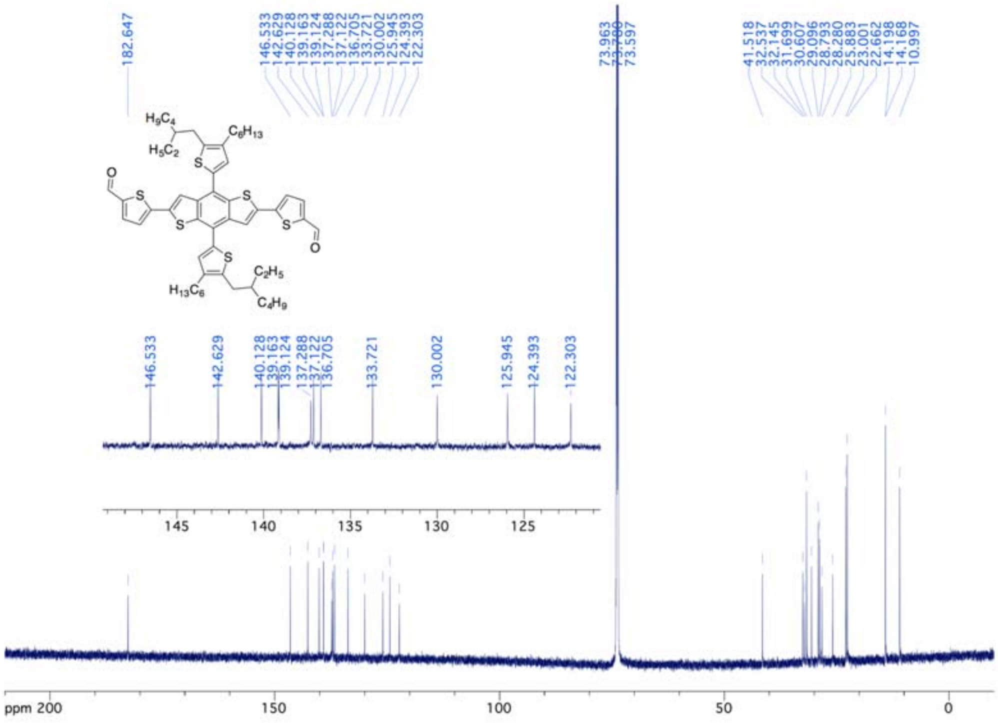

Figure S3.48. [13]*C-NMR of 5,5'-(4,8-bis(5-(2-ethylhexyl)-4-hexylthiophen-2-yl)benzo[1,2-b:4,5-b']dithiophene-2,6-diyl)bis(thiophene-2-carbaldehyde)) (BM-dialdehyde)*

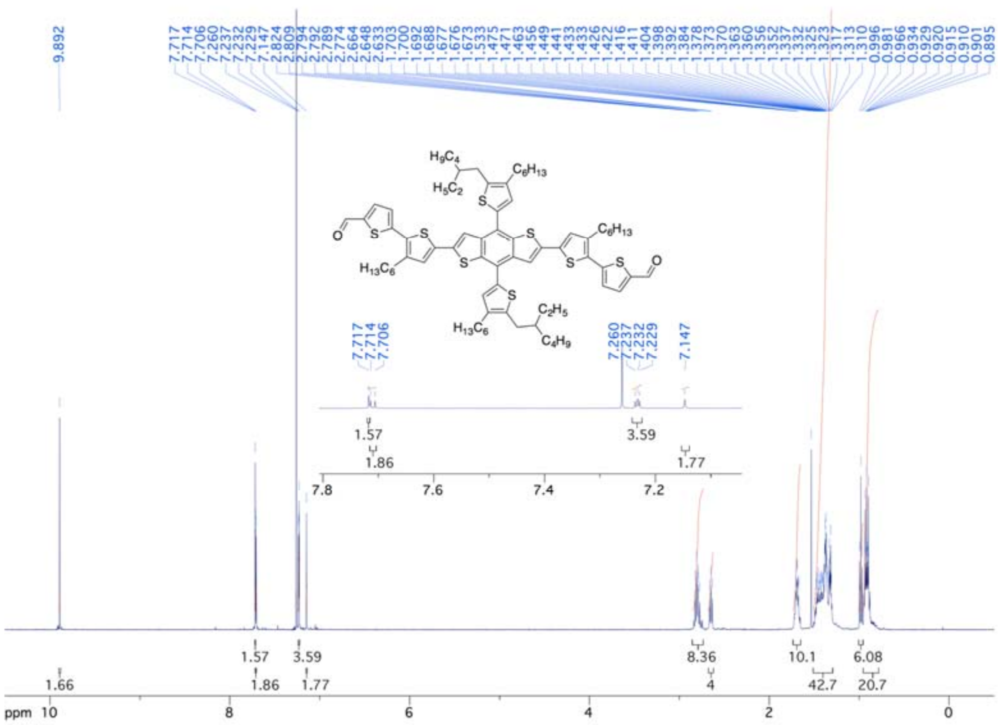

Figure S3.49. [1]*H-NMR of 5',5'''-(4,8-bis(5-(2-ethylhexyl)-4-hexylthiophen-2-yl)benzo[1,2-b:4,5-b']dithiophene-2,6-diyl)bis(3'-hexyl-[2,2'-bithiophene]-5-carbaldehyde) (BB-dialdehyde)*

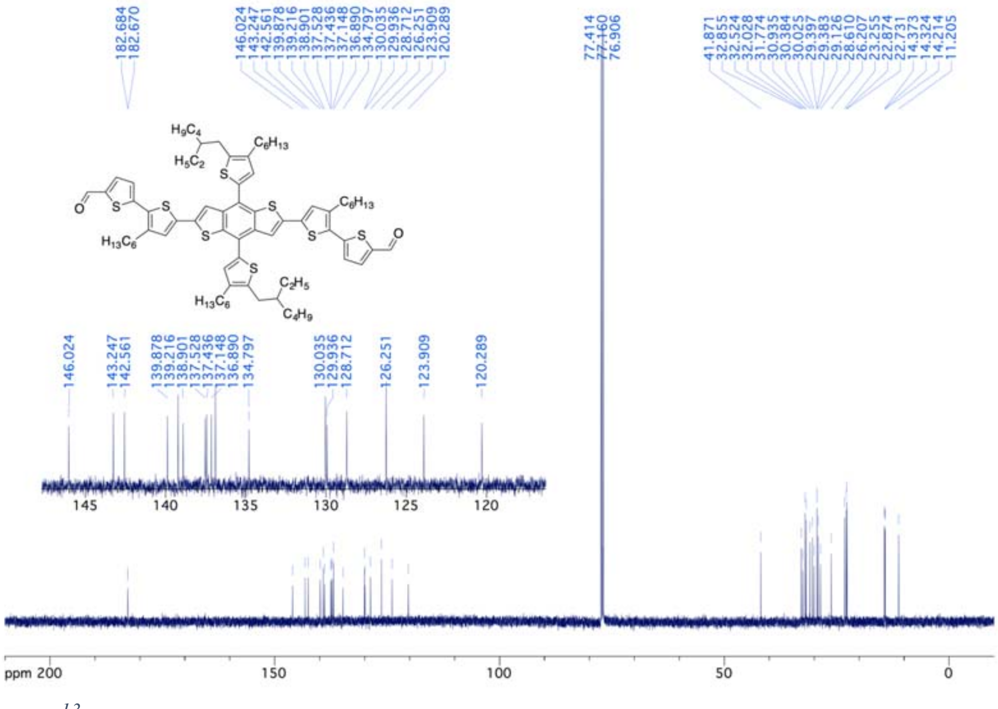

Figure S3.50. [13]*C-NMR of 5',5'''-(4,8-bis(5-(2-ethylhexyl)-4-hexylthiophen-2-yl)benzo[1,2-b:4,5-b']dithiophene-2,6-diyl)bis(3'-hexyl-[2,2'-bithiophene]-5-carbaldehyde) (BB-dialdehyde)*

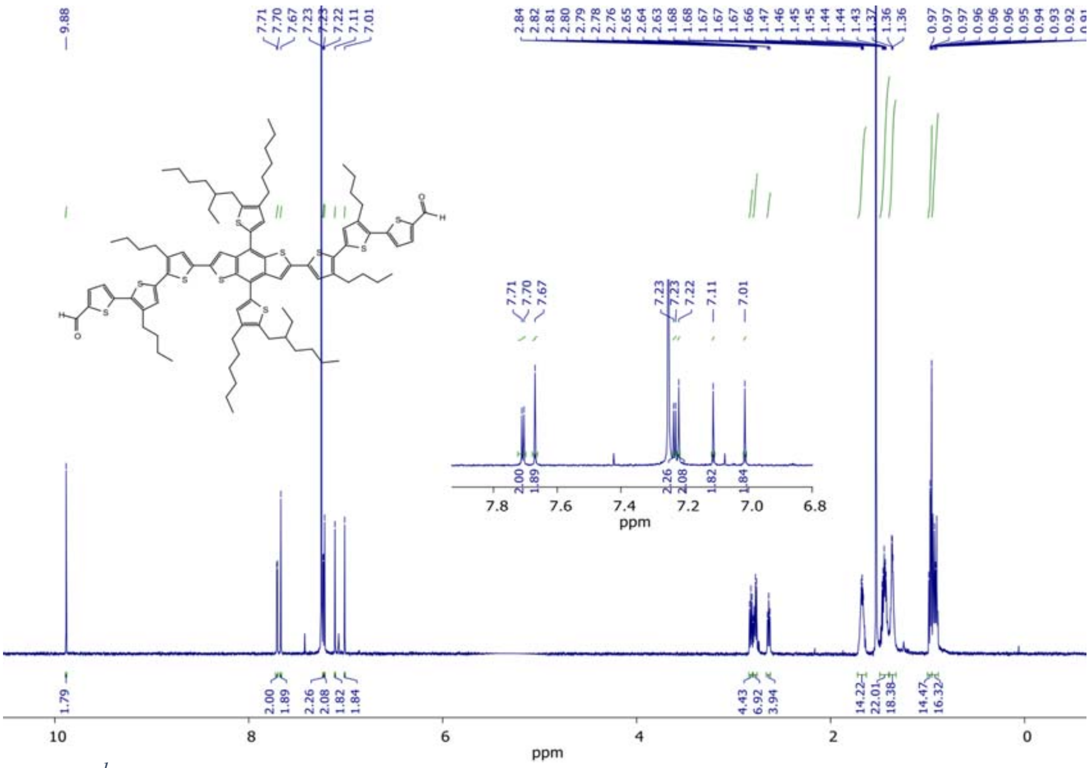

Figure S3.51. *^{1}H-NMR of 5'',5'''''-(4,8-bis(5-(2-ethylhexyl)-4-hexylthiophen-2-yl)benzo[1,2-b:4,5-b']dithiophene-2,6-diyl)bis(3',3''-dibutyl-[2,2':5',2''-terthiophene]-5-carbaldehyde) (BT^4-dialdehyde)*

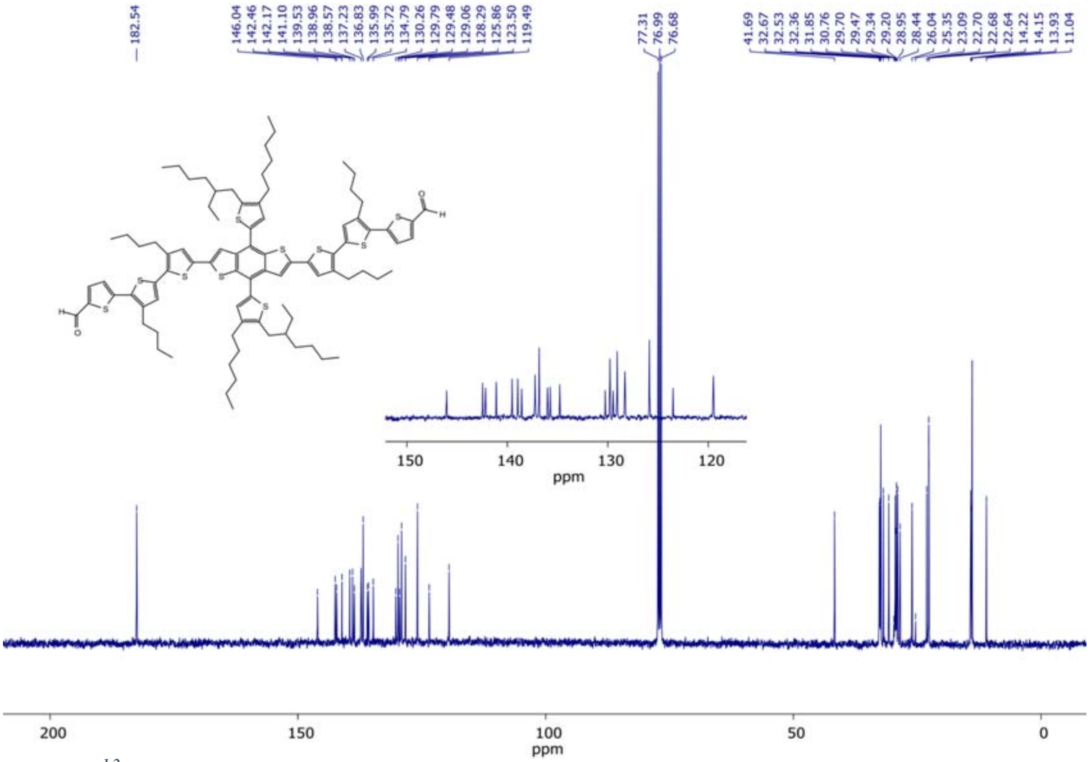

Figure S3.52. *^{13}C-NMR of 5'',5'''''-(4,8-bis(5-(2-ethylhexyl)-4-hexylthiophen-2-yl)benzo[1,2-b:4,5-b']dithiophene-2,6-diyl)bis(3',3''-dibutyl-[2,2':5',2''-terthiophene]-5-carbaldehyde) (BT^4-dialdehyde)*

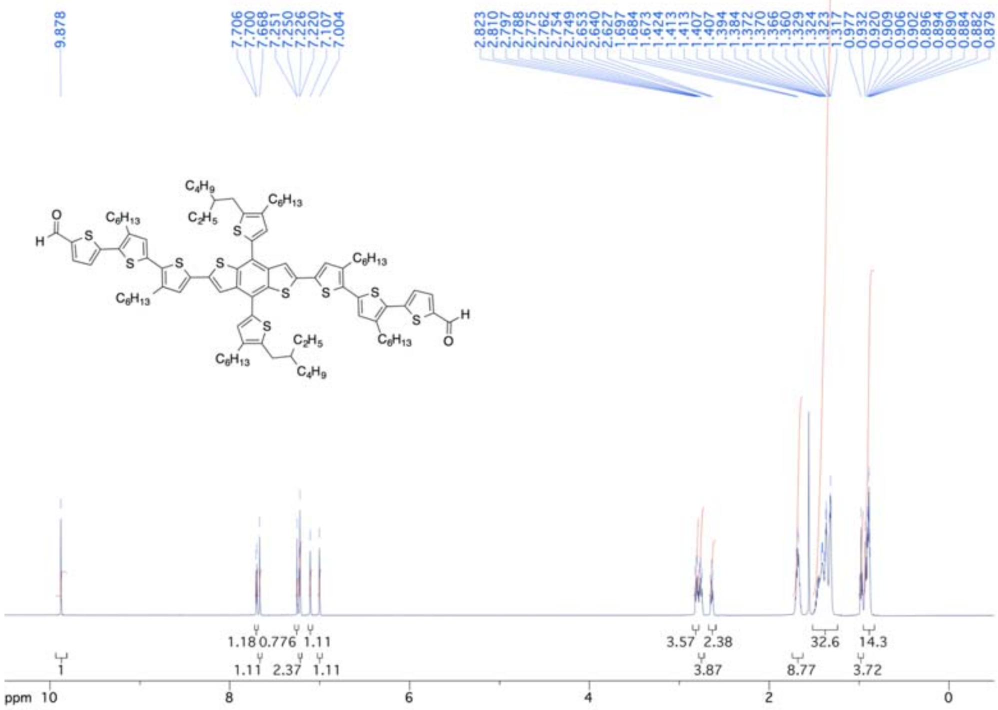

Figure S3.53. ^{1}H-NMR of 5'',5'''''-(4,8-bis(5-(2-ethylhexyl)-4-hexylthiophen-2-yl)benzo[1,2-b:4,5-b']dithiophene-2,6-diyl)bis(3',3''-dihexyl-[2,2':5',2''-terthiophene]-5-carbaldehyde) (BT-dialdehyde)

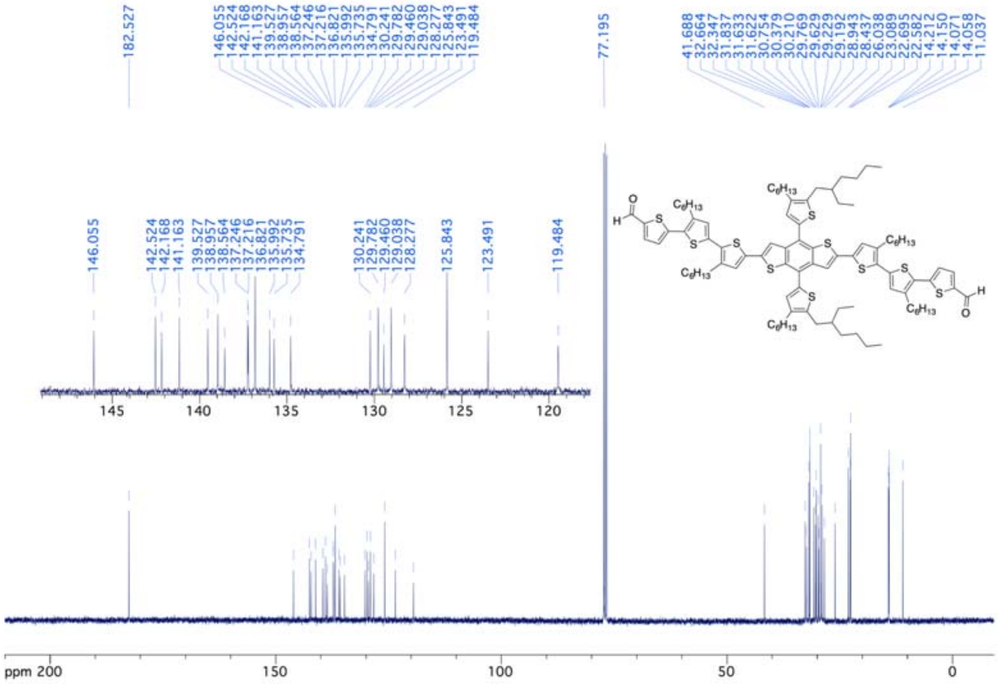

Figure S3.54. ^{13}C-NMR of 5'',5'''''-(4,8-bis(5-(2-ethylhexyl)-4-hexylthiophen-2-yl)benzo[1,2-b:4,5-b']dithiophene-2,6-diyl)bis(3',3''-dihexyl-[2,2':5',2''-terthiophene]-5-carbaldehyde) (BT-dialdehyde)

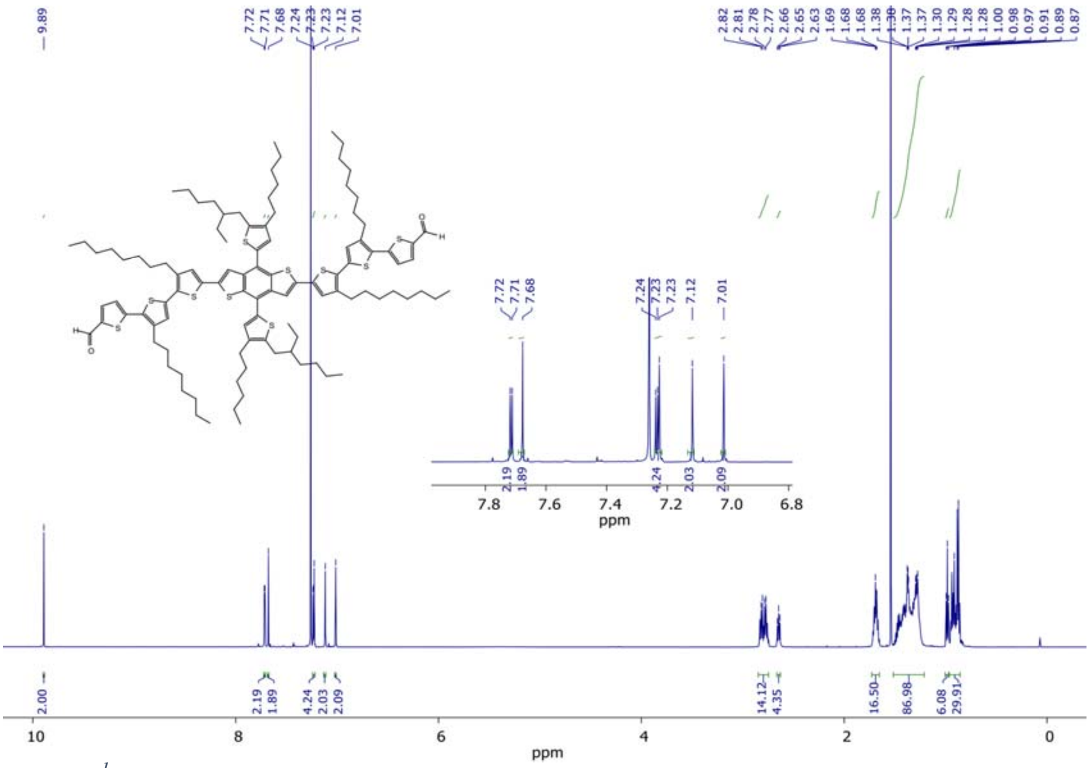

Figure S3.55. [1]*H-NMR of 5'',5''''-(4,8-bis(5-(2-ethylhexyl)-4-hexylthiophen-2-yl)benzo[1,2-b:4,5-b']dithiophene-2,6-diyl)bis(3',3''-dioctyl-[2,2':5',2''-terthiophene]-5-carbaldehyde) (BT*^{8}*-dialdehyde)*

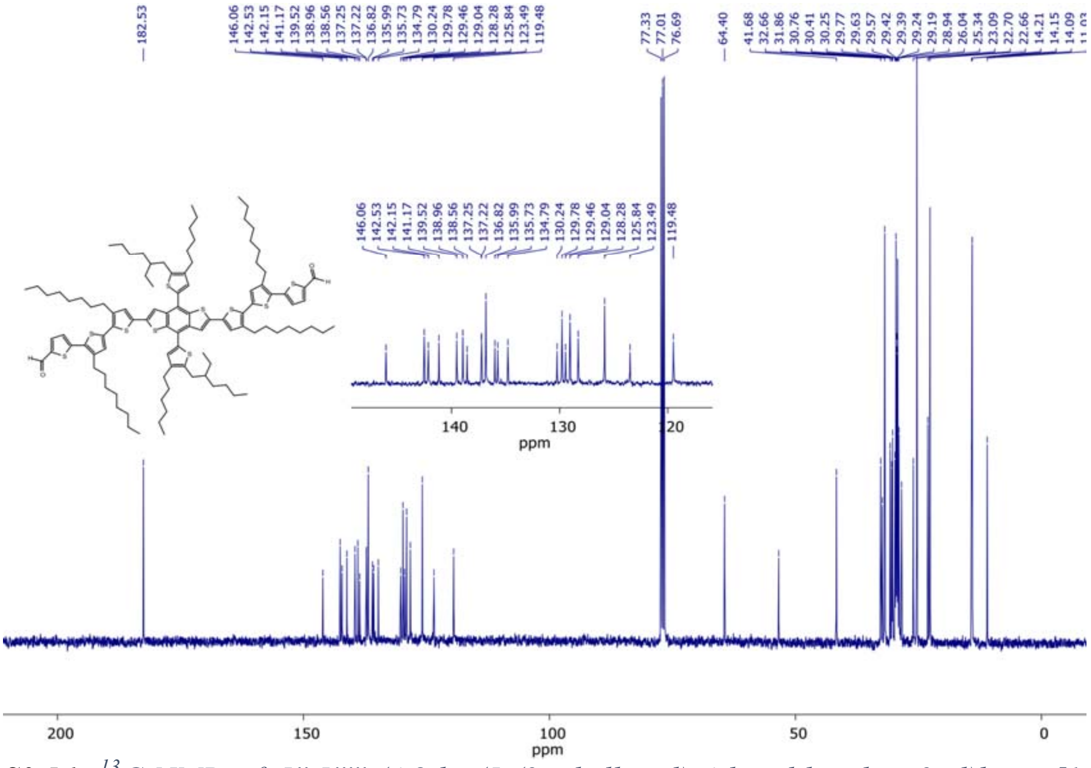

Figure S3.56. [13]*C-NMR of 5'',5''''-(4,8-bis(5-(2-ethylhexyl)-4-hexylthiophen-2-yl)benzo[1,2-b:4,5-b']dithiophene-2,6-diyl)bis(3',3''-dioctyl-[2,2':5',2''-terthiophene]-5-carbaldehyde) (BT*^{8}*-dialdehyde)*

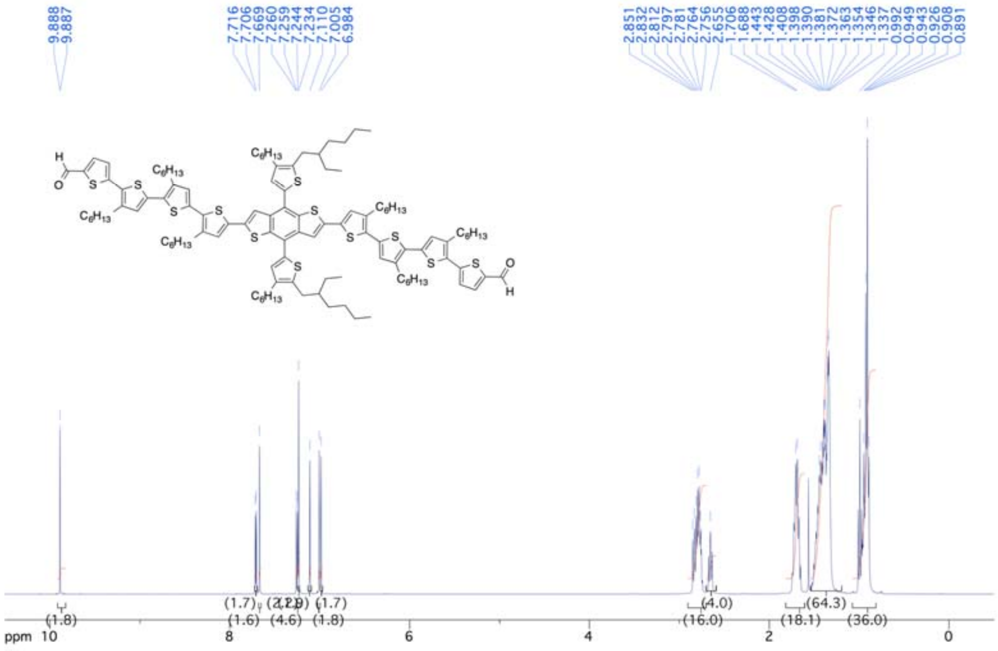

Figure S3.57. ^{1}H-NMR of 5''',5'''''''-(4,8-bis(5-(2-ethylhexyl)-4-hexylthiophen-2-yl)benzo[1,2-b:4,5-b']dithiophene-2,6-diyl)bis(3',3'',3'''-trihexyl-[2,2':5',2'':5'',2'''-quaterthiophene]-5-carbaldehyde) (BQ-dialdehyde)

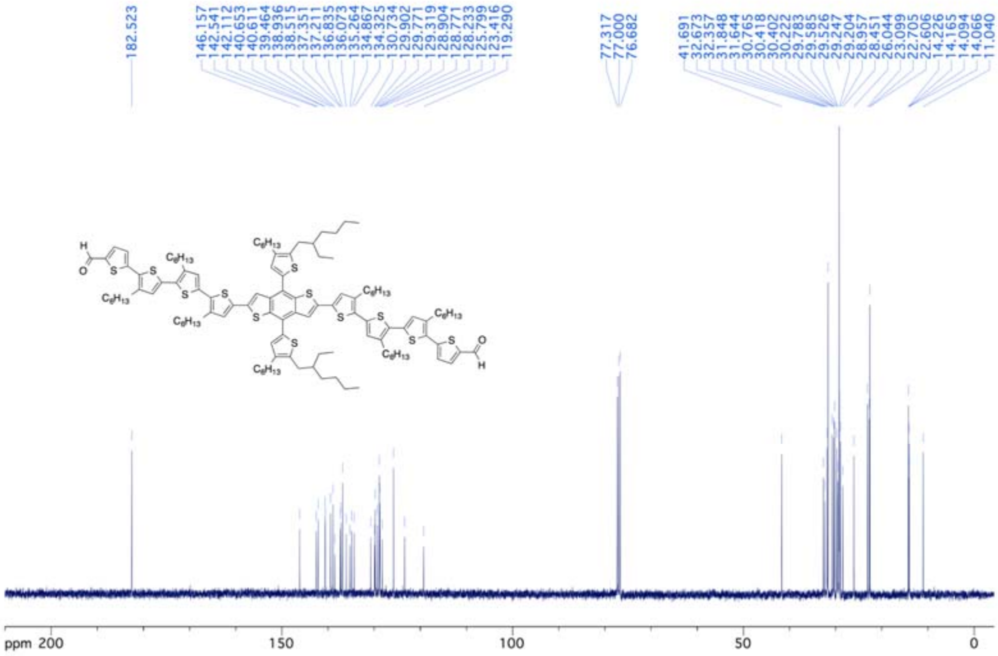

Figure S3.58. ^{13}C-NMR of 5''',5'''''''-(4,8-bis(5-(2-ethylhexyl)-4-hexylthiophen-2-yl)benzo[1,2-b:4,5-b']dithiophene-2,6-diyl)bis(3',3'',3'''-trihexyl-[2,2':5',2'':5'',2'''-quaterthiophene]-5-carbaldehyde) (BQ-dialdehyde)

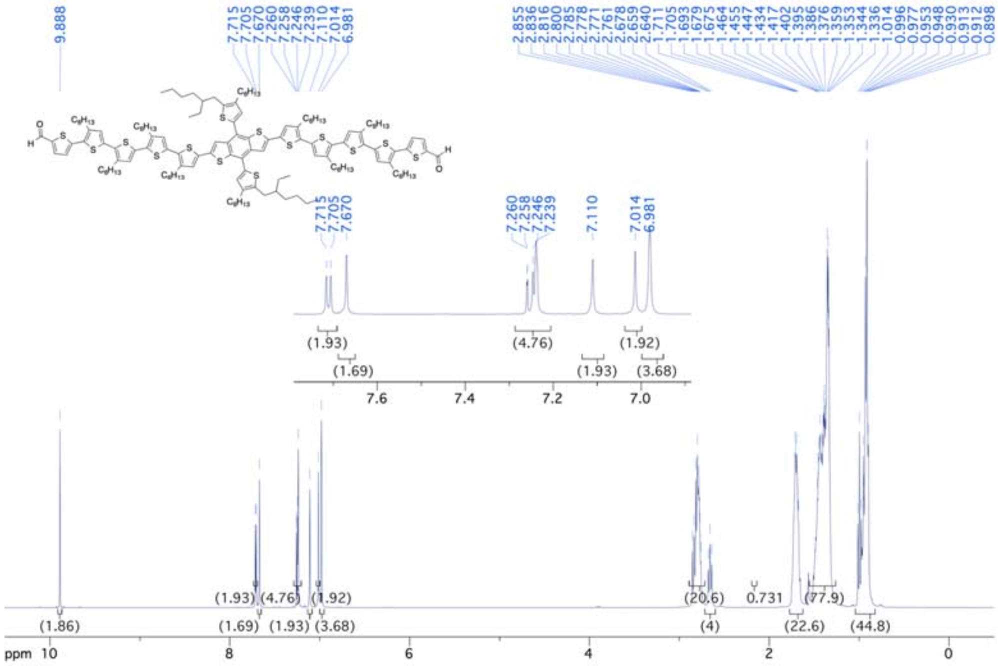

Figure S3.59. ^{1}H-NMR of 5'''',5''''''''-(4,8-bis(5-(2-ethylhexyl)-4-hexylthiophen-2-yl)benzo[1,2-b:4,5-b']dithiophene-2,6-diyl)bis(3',3'',3''',3''''-tetrahexyl-[2,2':5',2'':5'',2''':5''',2''''-quinquethiophene]-5-carbaldehyde) (BP-dialdehyde)

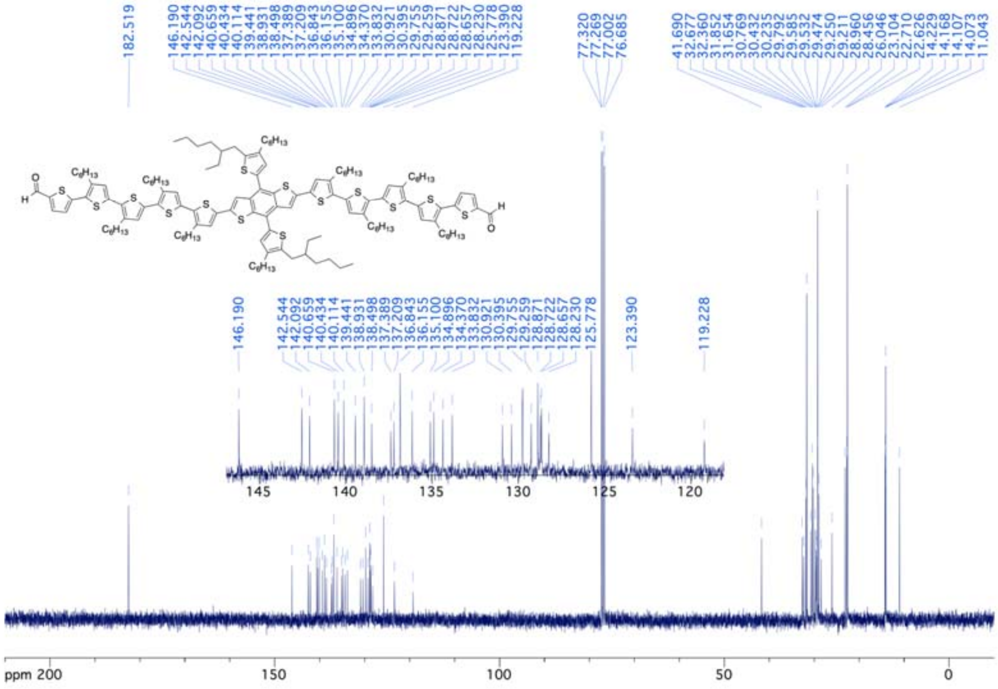

Figure S3.60. ^{13}C-NMR of 5'''',5''''''''-(4,8-bis(5-(2-ethylhexyl)-4-hexylthiophen-2-yl)benzo[1,2-b:4,5-b']dithiophene-2,6-diyl)bis(3',3'',3''',3''''-tetrahexyl-[2,2':5',2'':5'',2''':5''',2''''-quinquethiophene]-5-carbaldehyde) (BP-dialdehyde)

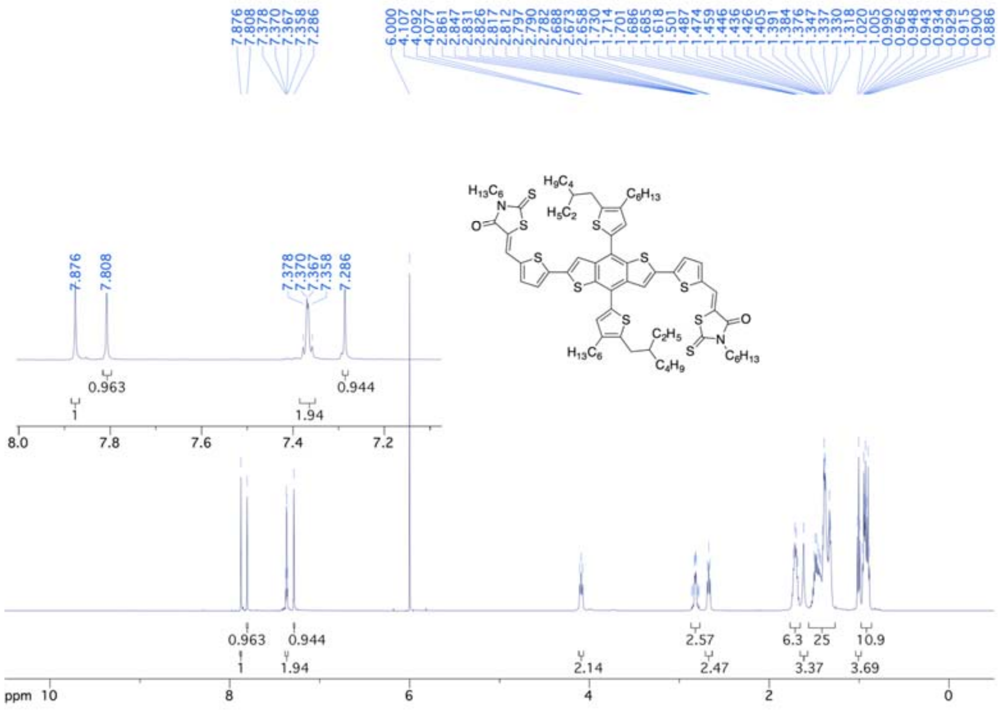

Figure S3.61. ^{1}H-NMR of BMR, (5Z,5'Z)-5,5'-((5,5'-(4,8-bis(5-(2-ethylhexyl)-4-hexylthiophen-2-yl)benzo[1,2-b:4,5-b']dithiophene-2,6-diyl)bis(thiophene-5,2-diyl))bis(methanylylidene))bis(3-hexyl-2-thioxothiazolidin-4-one)

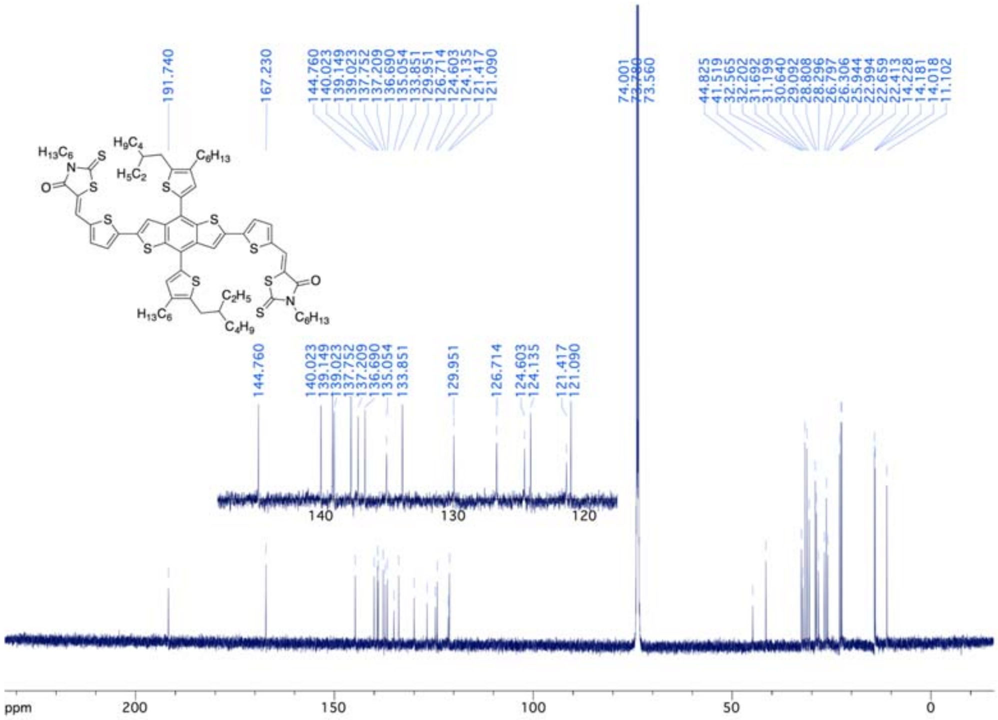

Figure S3.62. ^{13}C-NMR of BMR, (5Z,5'Z)-5,5'-((5,5'-(4,8-bis(5-(2-ethylhexyl)-4-hexylthiophen-2-yl)benzo[1,2-b:4,5-b']dithiophene-2,6-diyl)bis(thiophene-5,2-diyl))bis(methanylylidene))bis(3-hexyl-2-thioxothiazolidin-4-one)

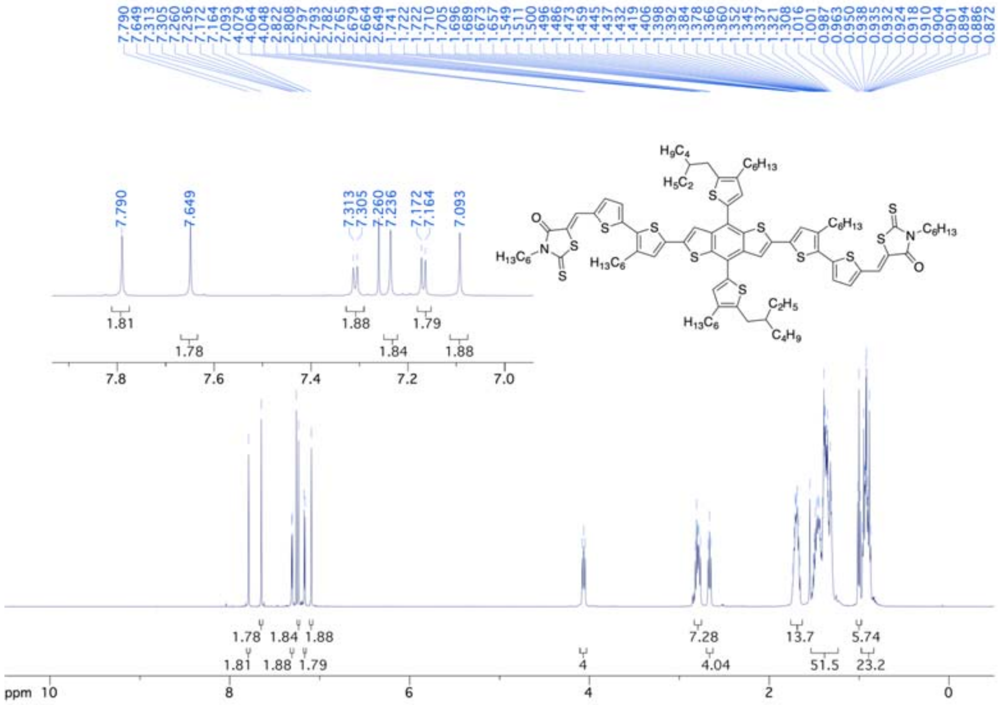

Figure S3.63. ^{1}H-NMR of BBR, (5Z,5'Z)-5,5'-((5',5'''-(4,8-bis(5-(2-ethylhexyl)-4-hexylthiophen-2-yl)benzo[1,2-b:4,5-b']dithiophene-2,6-diyl)bis(3'-hexyl-[2,2'-bithiophene]-5',5-diyl))bis(methanylylidene))bis(3-hexyl-2-thioxothiazolidin-4-one)

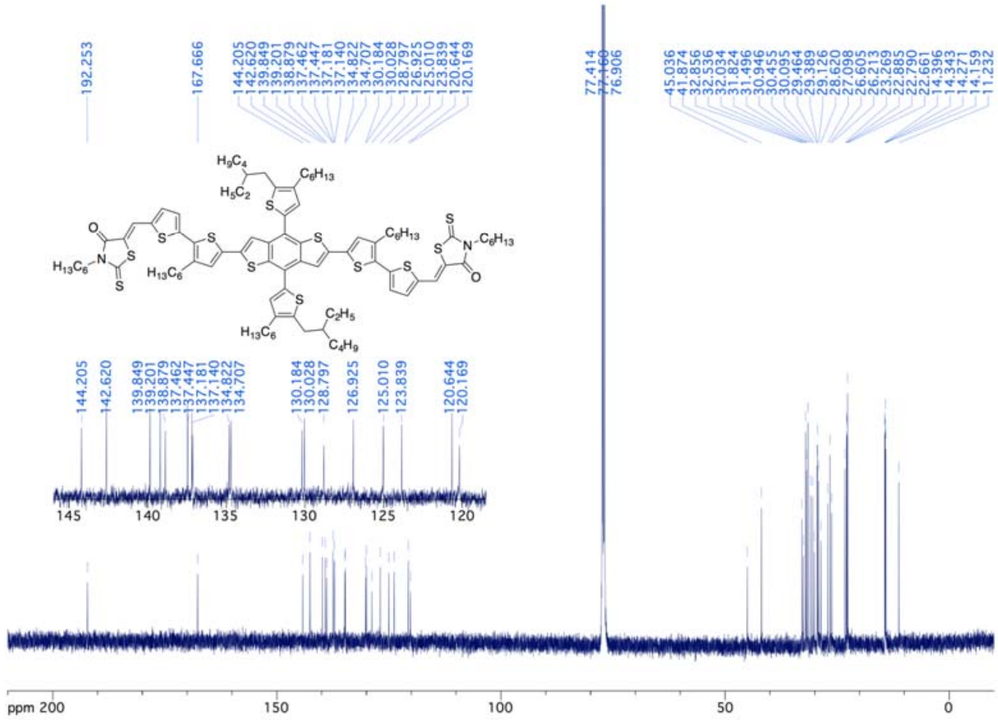

Figure S3.64. ^{13}C-NMR of BBR, (5Z,5'Z)-5,5'-((5',5'''-(4,8-bis(5-(2-ethylhexyl)-4-hexylthiophen-2-yl)benzo[1,2-b:4,5-b']dithiophene-2,6-diyl)bis(3'-hexyl-[2,2'-bithiophene]-5',5-diyl))bis(methanylylidene))bis(3-hexyl-2-thioxothiazolidin-4-one)

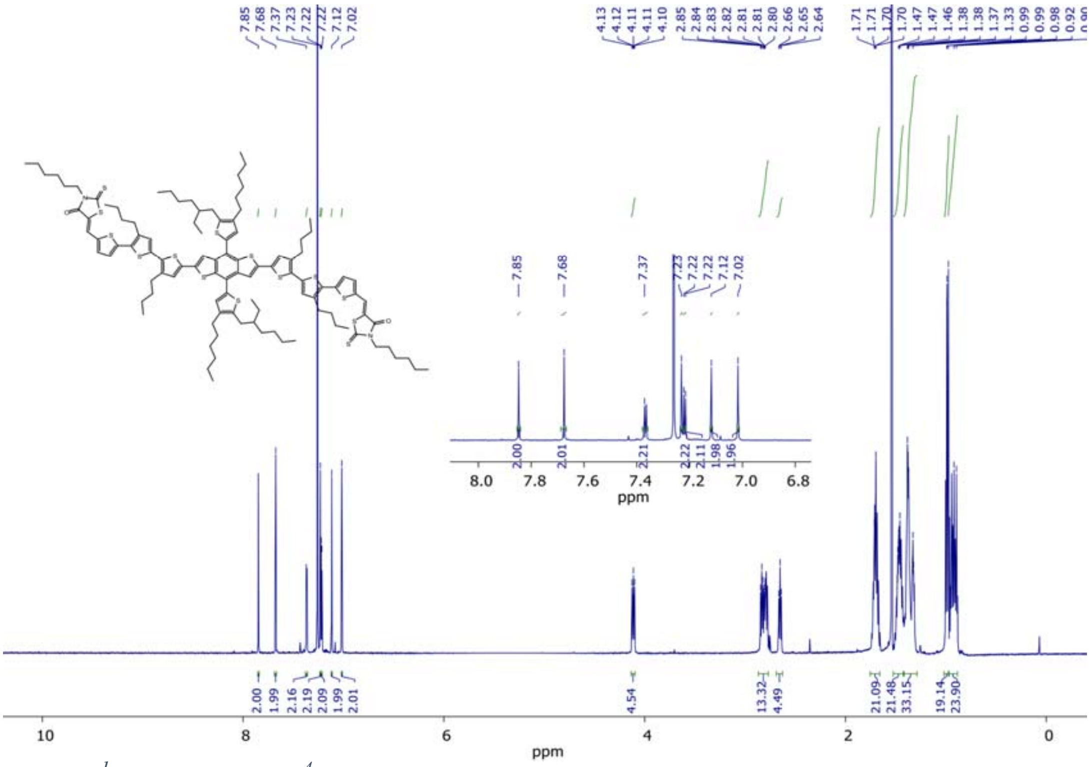

Figure S3.65. ^{1}H-NMR of BT[4]R, 5'',5'''''-(4,8-bis(5-(2-ethylhexyl)-4-hexylthiophen-2-yl)benzo[1,2-b:4,5-b']dithiophene-2,6-diyl)bis(3',3''-dibutyl-[2,2':5',2''-terthiophene]-5-hexylrhodanine)

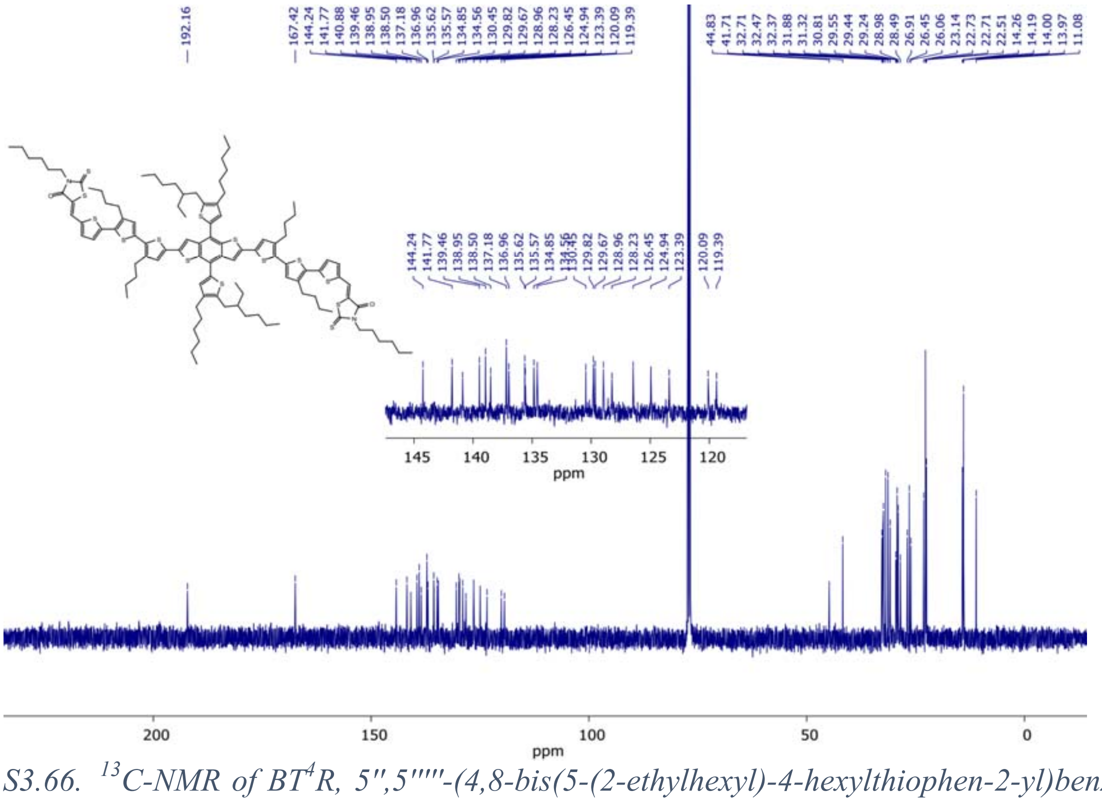

Figure S3.66. ^{13}C-NMR of BT[4]R, 5'',5'''''-(4,8-bis(5-(2-ethylhexyl)-4-hexylthiophen-2-yl)benzo[1,2-b:4,5-b']dithiophene-2,6-diyl)bis(3',3''-dibutyl-[2,2':5',2''-terthiophene]-5-hexylrhodanine)

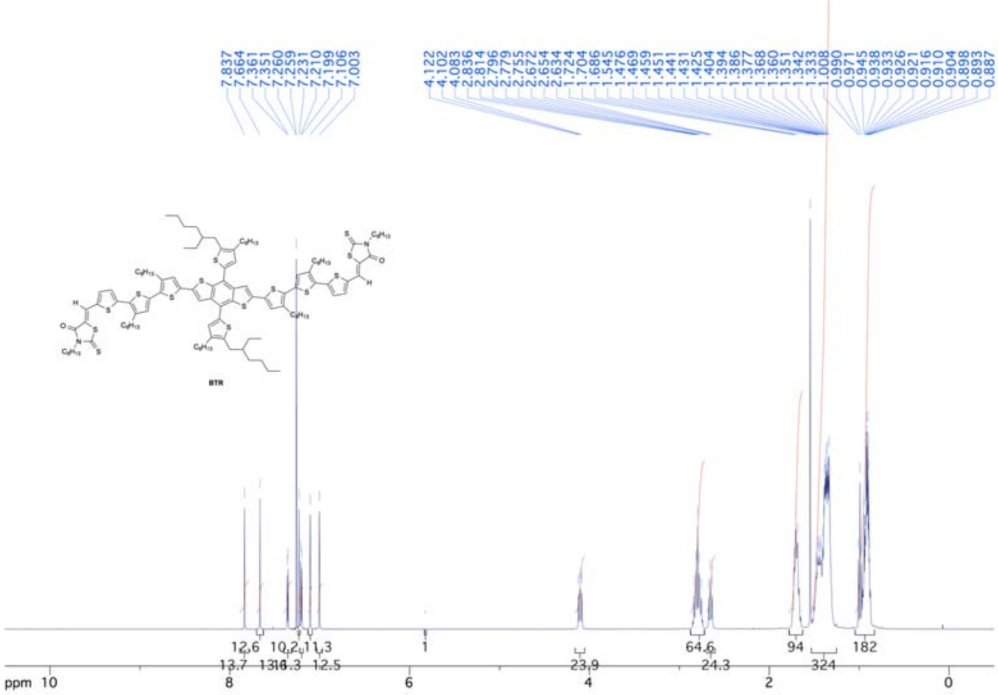

Figure S3.67. ^{1}H-NMR of BTR, (5Z,5'Z)-5,5'-((5'',5'''''-(4,8-bis(5-(2-ethylhexyl)-4-hexylthiophen-2-yl)benzo[1,2-b:4,5-b']dithiophene-2,6-diyl)bis(3',3''-dihexyl-[2,2':5',2''-terthiophene]-5'',5-diyl))bis(methanylylidene))bis(3-hexyl-2-thioxothiazolidin-4-one)

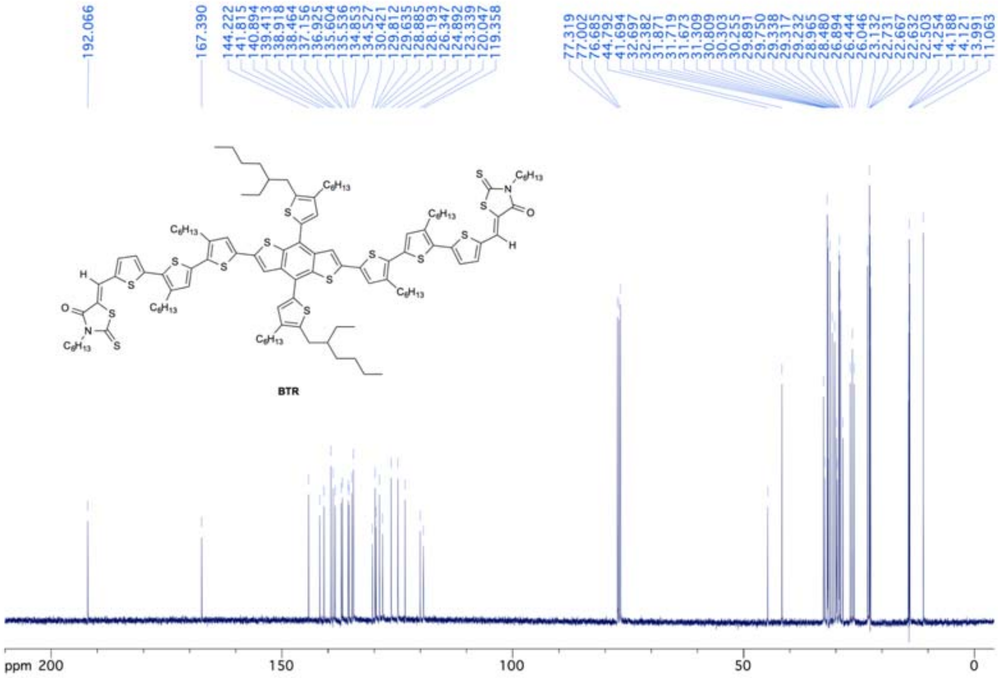

Figure S3.68. ^{13}C-NMR of BTR, (5Z,5'Z)-5,5'-((5'',5'''''-(4,8-bis(5-(2-ethylhexyl)-4-hexylthiophen-2-yl)benzo[1,2-b:4,5-b']dithiophene-2,6-diyl)bis(3',3''-dihexyl-[2,2':5',2''-terthiophene]-5'',5-diyl))bis(methanylylidene))bis(3-hexyl-2-thioxothiazolidin-4-one)

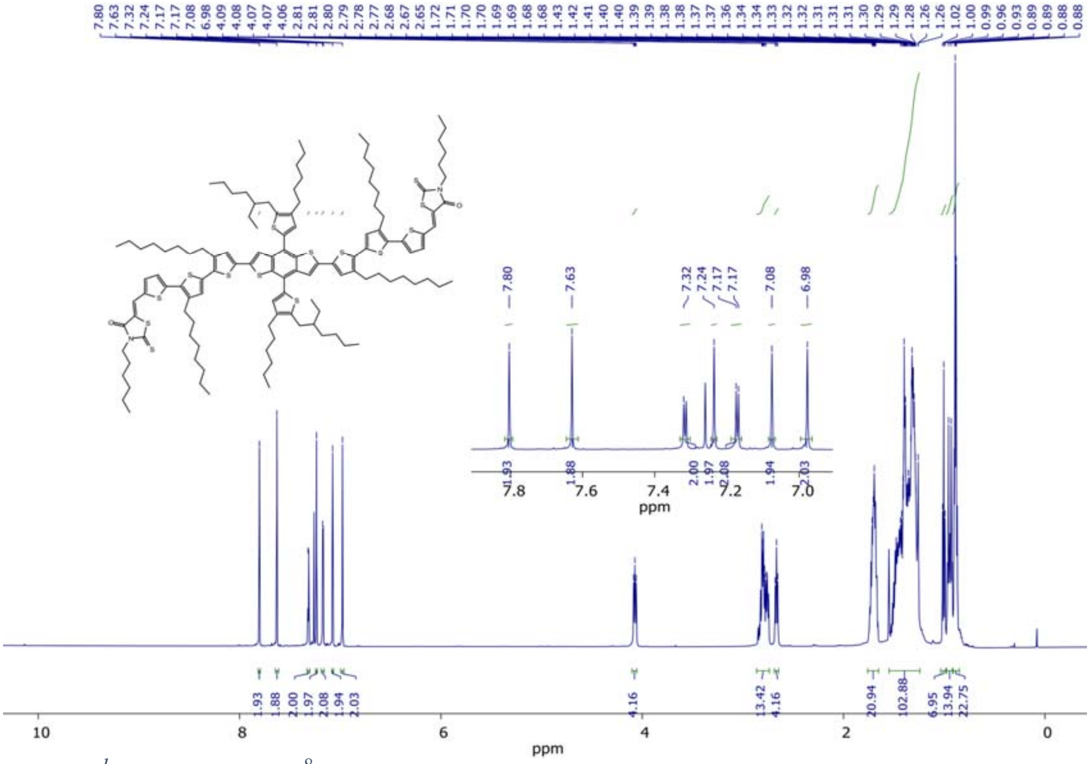

Figure S3.69. ^{1}H-NMR of BT[8]R, 5'',5'''''-(4,8-bis(5-(2-ethylhexyl)-4-hexylthiophen-2-yl)benzo[1,2-b:4,5-b']dithiophene-2,6-diyl)bis(3',3''-dioctyl-[2,2':5',2''-terthiophene]-5-hexylrhodanine)

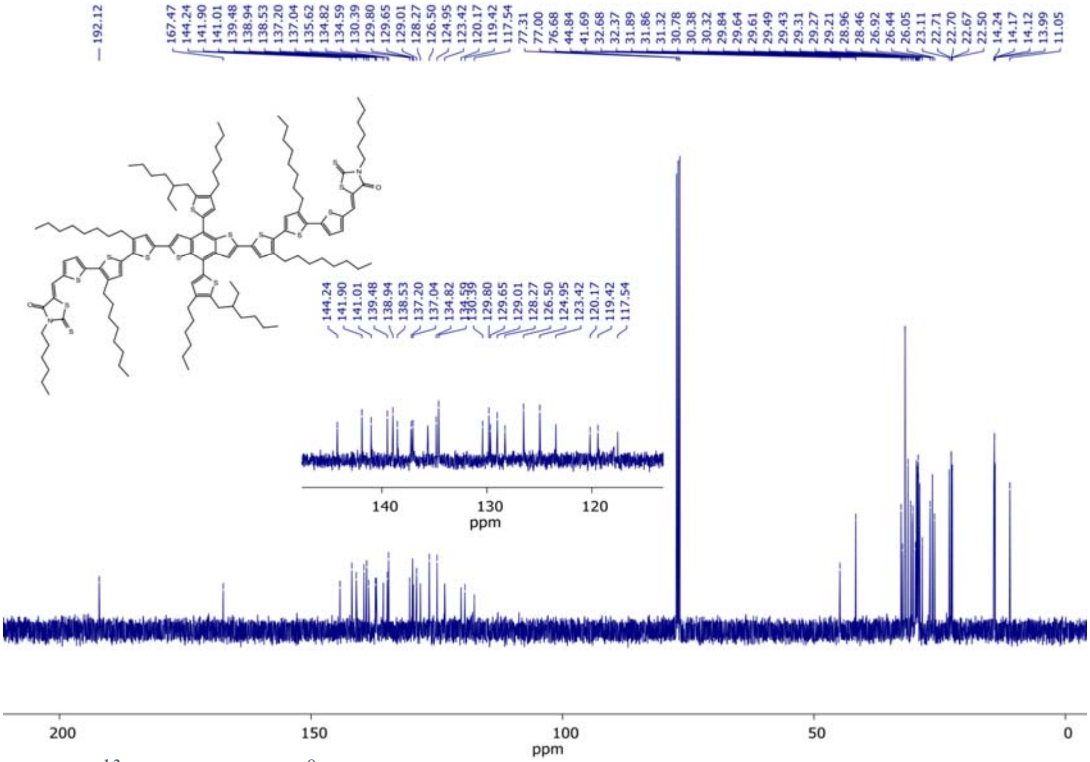

Figure S3.70. ^{13}C-NMR of BT[8]R, 5'',5'''''-(4,8-bis(5-(2-ethylhexyl)-4-hexylthiophen-2-yl)benzo[1,2-b:4,5-b']dithiophene-2,6-diyl)bis(3',3''-dioctyl-[2,2':5',2''-terthiophene]-5-hexylrhodanine)

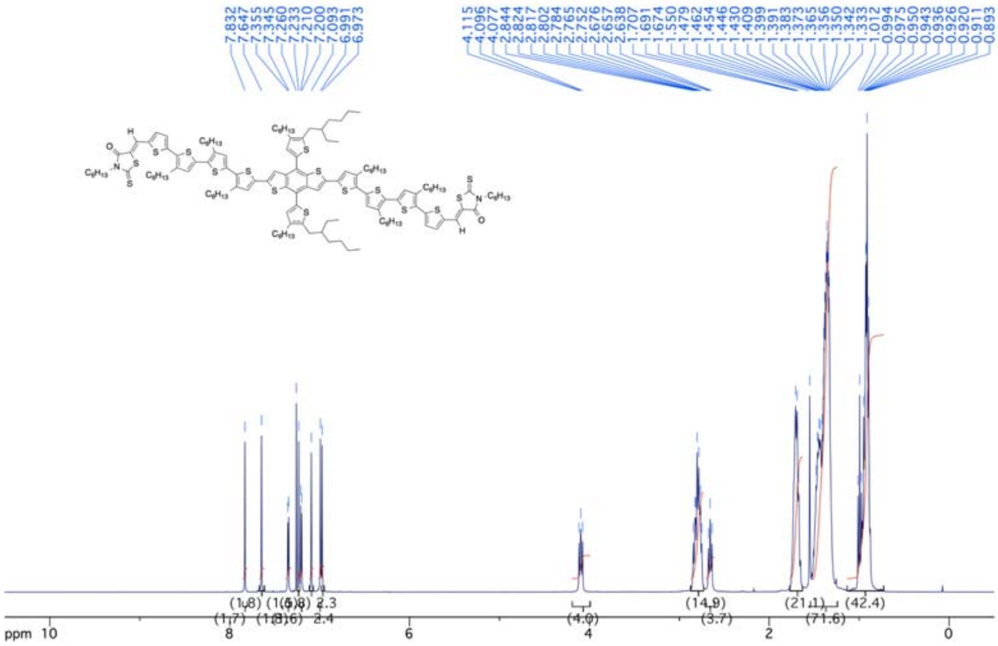

Figure S3.71. *^{1}H-NMR of BQR, (5Z,5'Z)-5,5'-((5''',5''''''-(4,8-bis(5-(2-ethylhexyl)-4-hexylthiophen-2-yl)benzo[1,2-b:4,5-b']dithiophene-2,6-diyl)bis(3',3'',3'''-trihexyl-[2,2':5',2'':5'',2'''-quaterthiophene]-5''',5-diyl))bis(methanylylidene))bis(3-hexyl-2-thioxothiazolidin-4-one)*

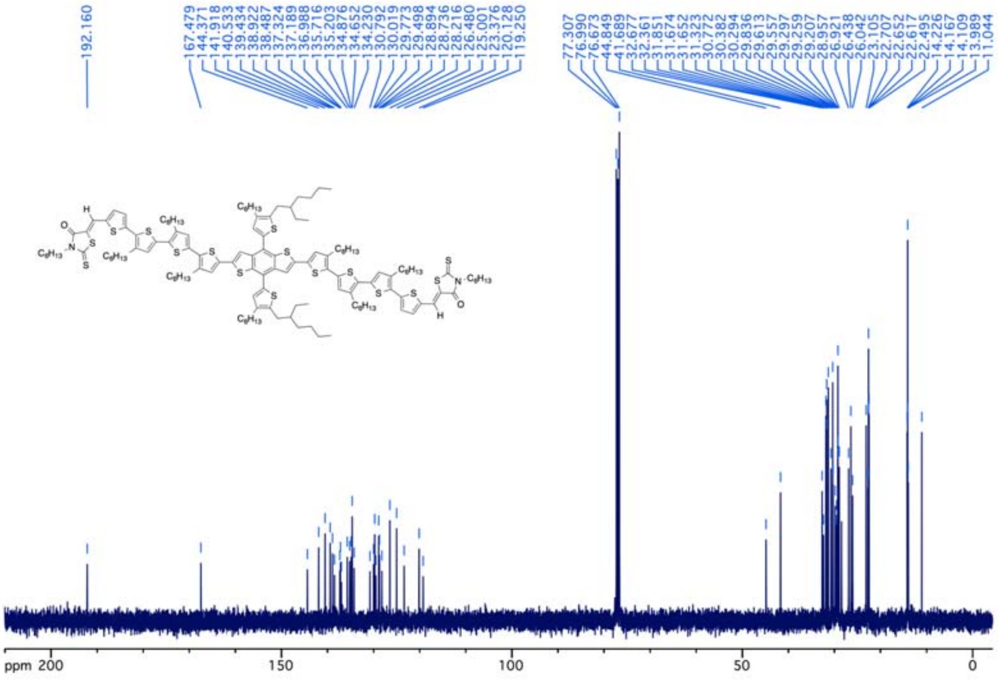

Figure S3.72. *^{13}C-NMR of BQR, (5Z,5'Z)-5,5'-((5''',5''''''-(4,8-bis(5-(2-ethylhexyl)-4-hexylthiophen-2-yl)benzo[1,2-b:4,5-b']dithiophene-2,6-diyl)bis(3',3'',3'''-trihexyl-[2,2':5',2'':5'',2'''-quaterthiophene]-5''',5-diyl))bis(methanylylidene))bis(3-hexyl-2-thioxothiazolidin-4-one)*

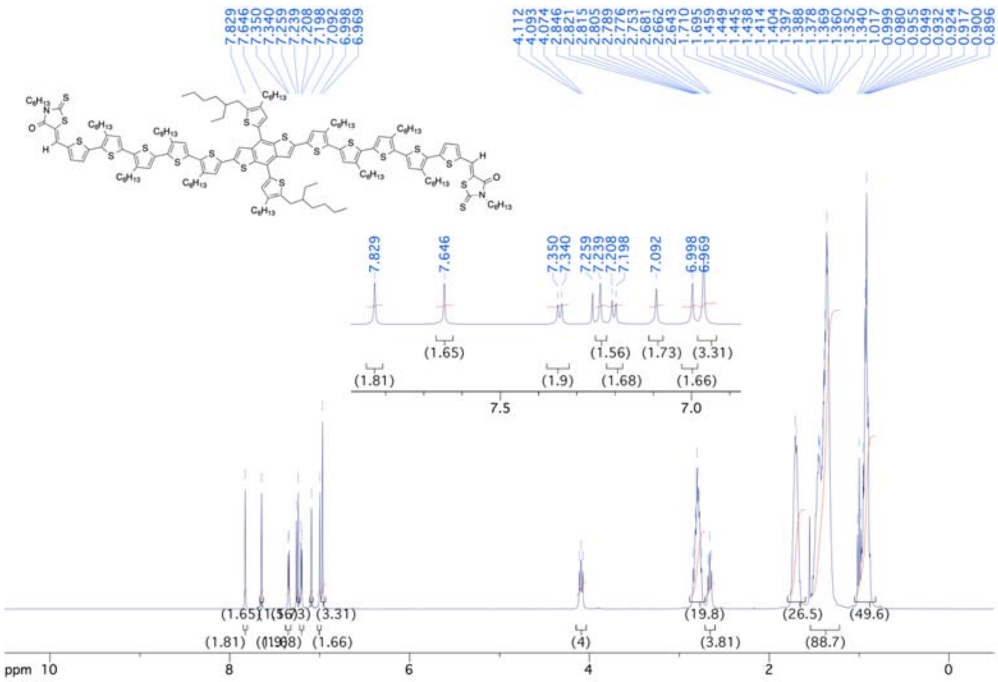

Figure S3.73. ^{1}H-NMR of BPR, (5Z,5'Z)-5,5'-((5'''',5''''''''-(4,8-bis(5-(2-ethylhexyl)-4-hexylthiophen-2-yl)benzo[1,2-b:4,5-b']dithiophene-2,6-diyl)bis(3',3'',3''',3''''-tetrahexyl-[2,2':5',2'':5'',2''':5''',2''''-quinquethiophene]-5'''',5-diyl))bis(methanylylidene))bis(3-hexyl-2-thioxothiazolidin-4-one)

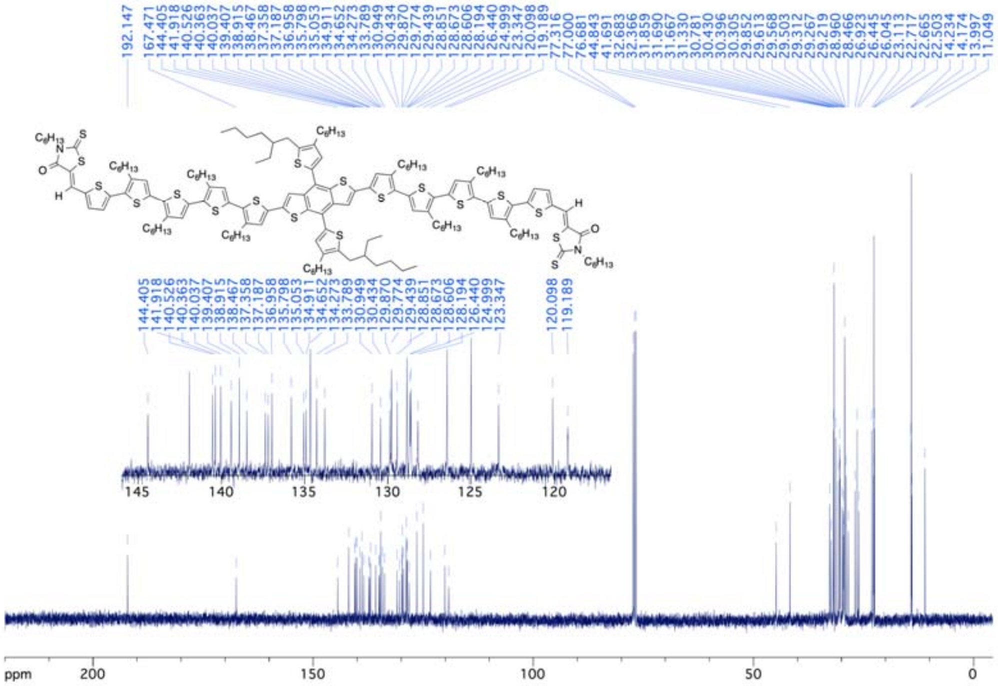

Figure S3.74. ^{13}C-NMR of BPR, (5Z,5'Z)-5,5'-((5'''',5''''''''-(4,8-bis(5-(2-ethylhexyl)-4-hexylthiophen-2-yl)benzo[1,2-b:4,5-b']dithiophene-2,6-diyl)bis(3',3'',3''',3''''-tetrahexyl-[2,2':5',2'':5'',2''':5''',2''''-quinquethiophene]-5'''',5-diyl))bis(methanylylidene))bis(3hexyl-2-thioxothiazolidin-4-one)

S4 MALDI spectra for the dialdehydes BX^{X}-dialdehyde and the BX^{X}R materials

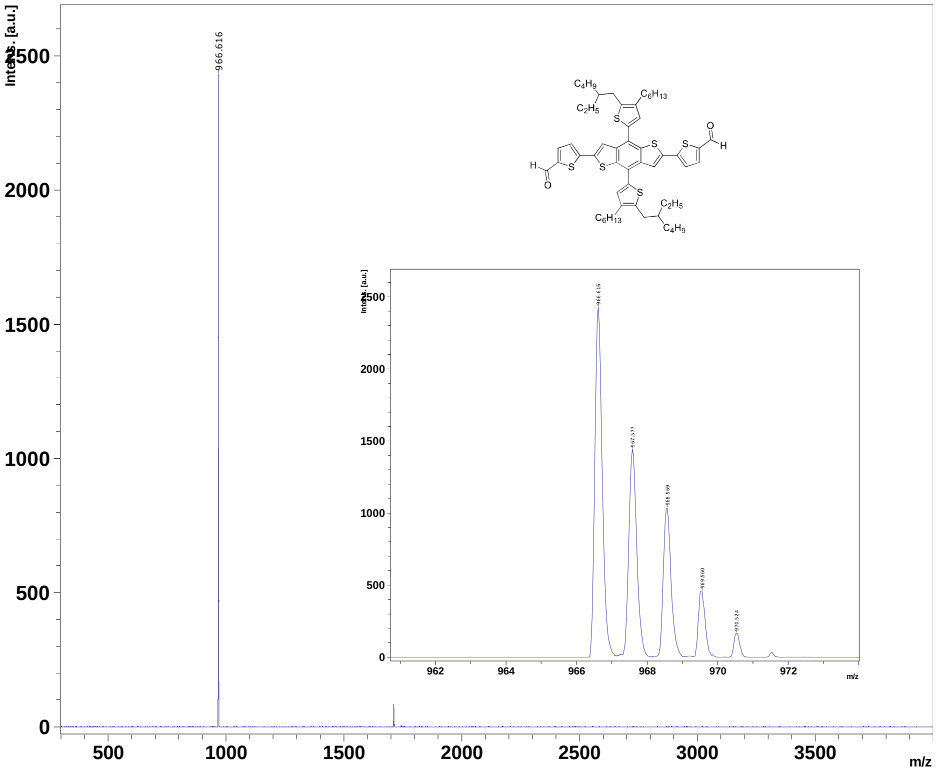

Figure S4.1. MALDI of ***BM****-dialdehyde, 5,5'-(4,8-bis(5-(2-ethylhexyl)-4-hexylthiophen-2-yl)benzo[1,2-b:4,5-b']dithiophene-2,6-diyl)bis(thiophene-2-carbaldehyde))*

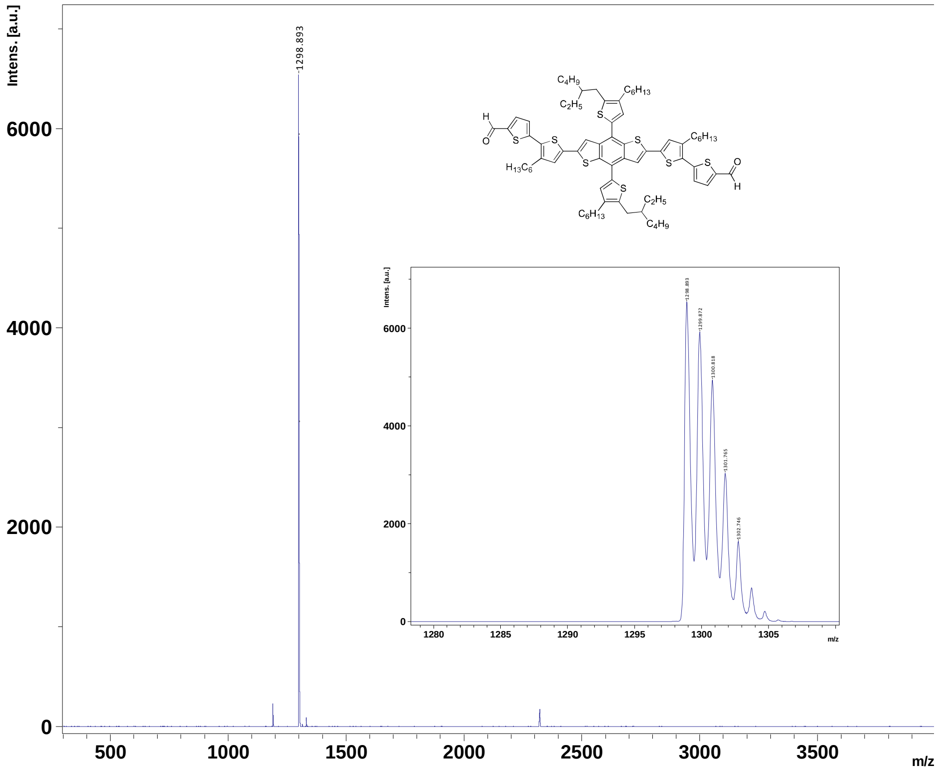

Figure S4.2. MALDI of ***BB****-dialdehyde, 5',5'''-(4,8-bis(5-(2-ethylhexyl)-4-hexylthiophen-2-yl)benzo[1,2-b:4,5-b']dithiophene-2,6-diyl)bis(3'-hexyl-[2,2'-bithiophene]-5-carbaldehyde)*

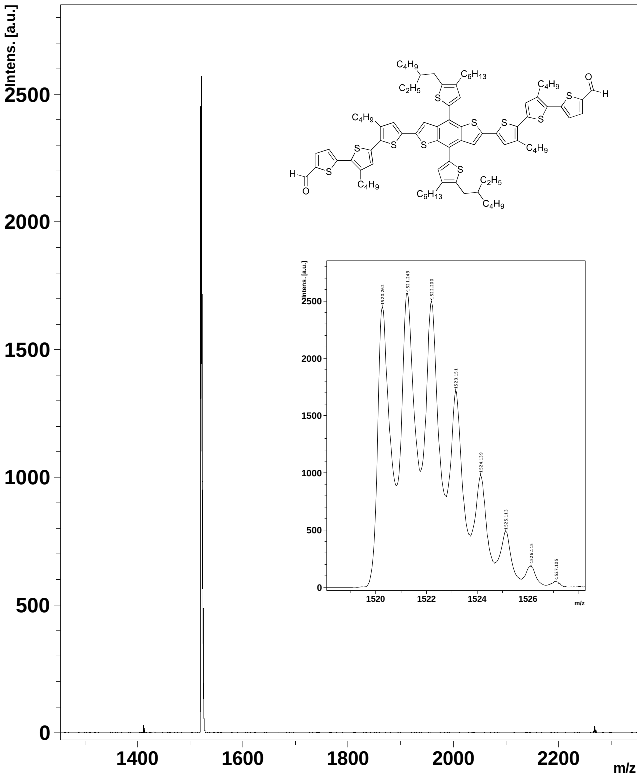

Figure S4.3. *MALDI of* ***BT***[4]*-dialdehyde, 5'',5''''-(4,8-bis(5-(2-ethylhexyl)-4-hexylthiophen-2-yl)benzo[1,2-b:4,5-b']dithiophene-2,6-diyl)bis(3',3''-dibutyl-[2,2':5',2''-terthiophene]-5-carbaldehyde)*

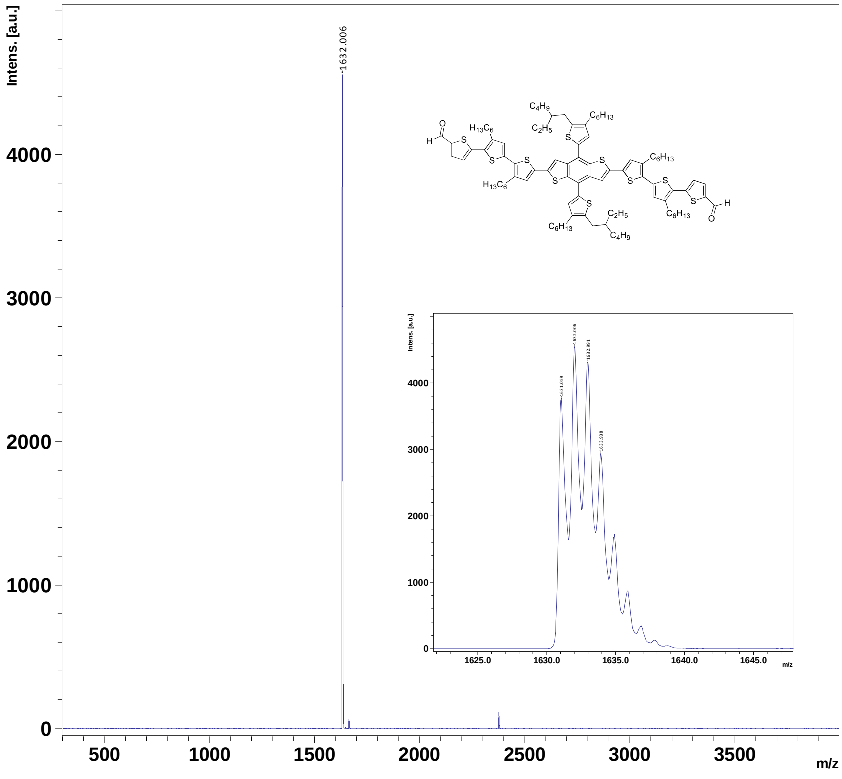

Figure S4.4. *MALDI of* ***BT****-dialdehyde, 5'',5''''-(4,8-bis(5-(2-ethylhexyl)-4-hexylthiophen-2-yl)benzo[1,2-b:4,5-b']dithiophene-2,6-diyl)bis(3',3''-dihexyl-[2,2':5',2''-terthiophene]-5-carbaldehyde)*

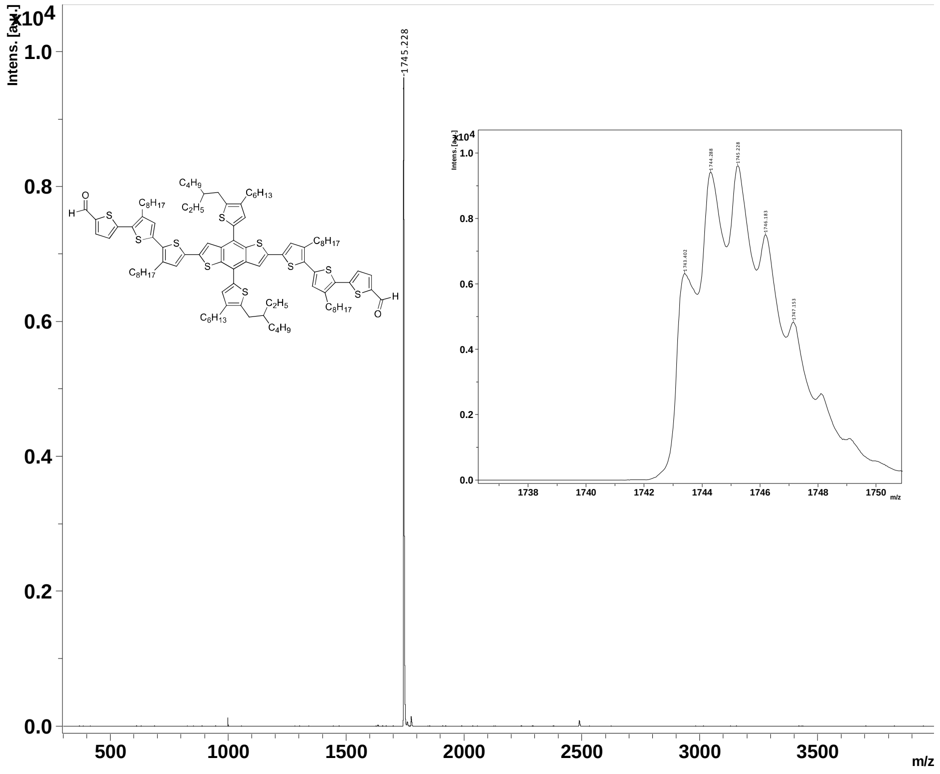

Figure S4.5. MALDI of ***BT^8^****-dialdehyde, 5'',5'''''-(4,8-bis(5-(2-ethylhexyl)-4-hexylthiophen-2-yl)benzo[1,2-b:4,5-b']dithiophene-2,6-diyl)bis(3',3''-dioctyl-[2,2':5',2''-terthiophene]-5-carbaldehyde)*

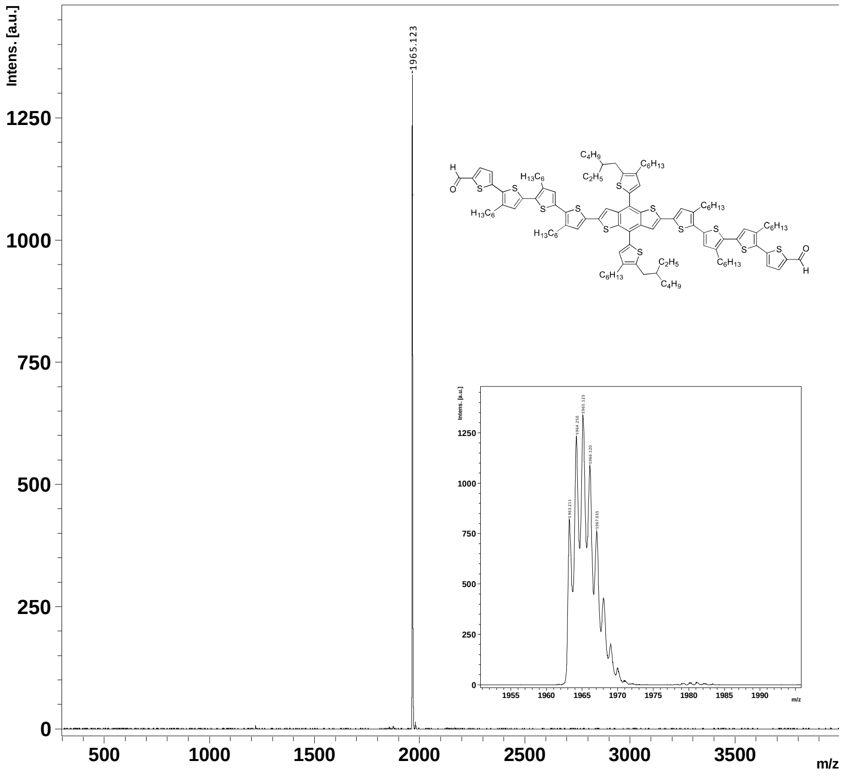

Figure S4.6. MALDI of ***BQ****-dialdehyde, 5''',5'''''''-(4,8-bis(5-(2-ethylhexyl)-4-hexylthiophen-2-yl)benzo[1,2-b:4,5-b']dithiophene-2,6-diyl)bis(3',3'',3'''-trihexyl-[2,2':5',2'':5'',2'''-quaterthiophene]-5-carbaldehyde)*

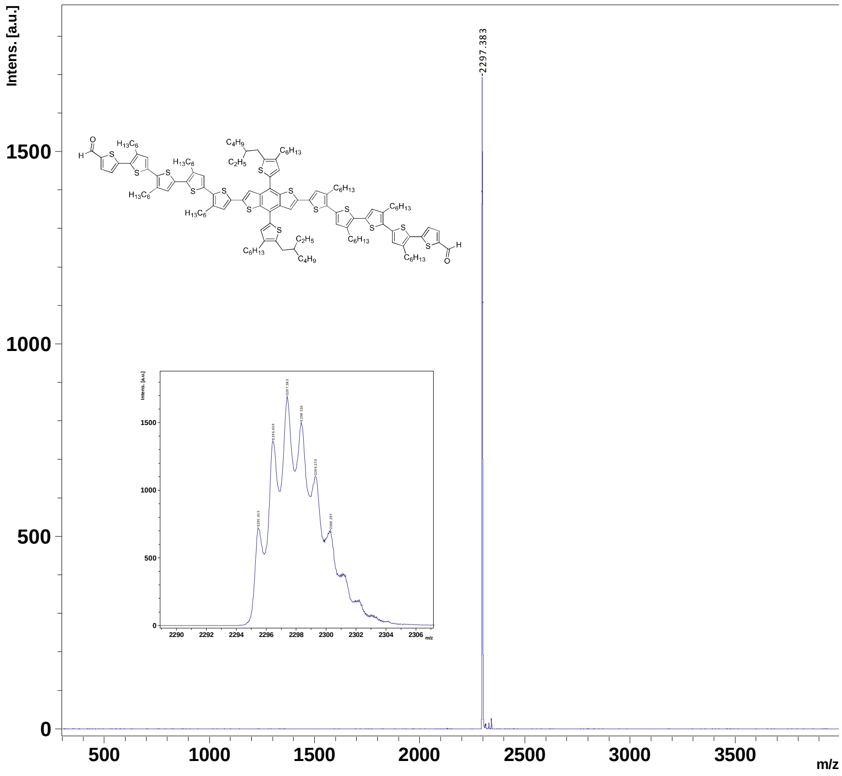

Figure S4.7. MALDI of ***BP****-dialdehyde, 5'''',5''''''''-(4,8-bis(5-(2-ethylhexyl)-4-hexylthiophen-2-yl)benzo[1,2-b:4,5-b']dithiophene-2,6-diyl)bis(3',3'',3''',3''''-tetrahexyl-[2,2':5',2'':5'',2''':5''',2''''-quinquethiophene]-5-carbaldehyde)*

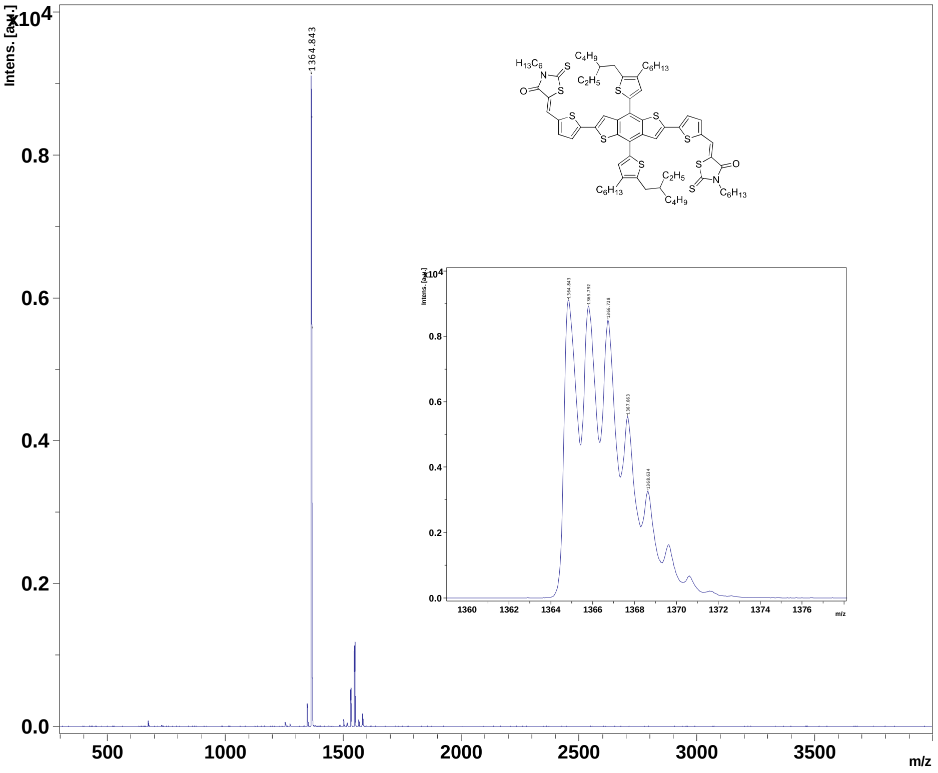

Figure S4.8. MALDI of ***BMR****, (5Z,5'Z)-5,5'-((5,5'-(4,8-bis(5-(2-ethylhexyl)-4-hexylthiophen-2-yl)benzo[1,2-b:4,5-b']dithiophene-2,6-diyl)bis(thiophene-5,2-diyl))bis(methanylylidene))bis(3-hexyl-2-thioxothiazolidin-4-one)*

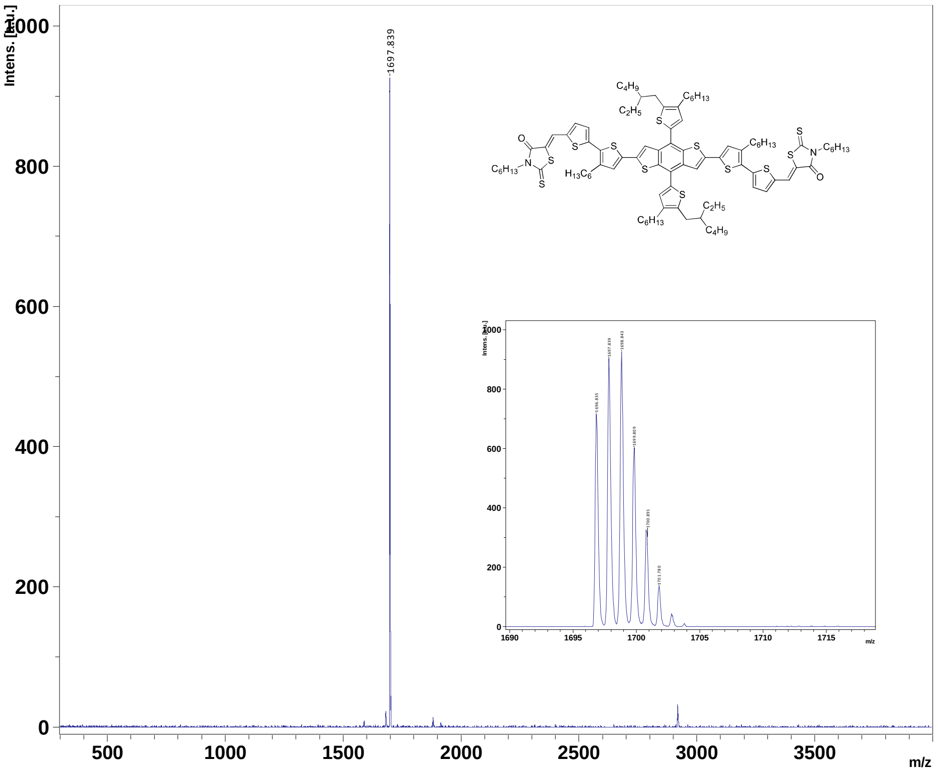

*Figure S4.9. MALDI of **BBR**, (5Z,5'Z)-5,5'-((5',5'''-(4,8-bis(5-(2-ethylhexyl)-4-hexylthiophen-2-yl)benzo[1,2-b:4,5-b']dithiophene-2,6-diyl)bis(3'-hexyl-[2,2'-bithiophene]-5',5-diyl))bis(methanylylidene))bis(3-hexyl-2-thioxothiazolidin-4-one)*

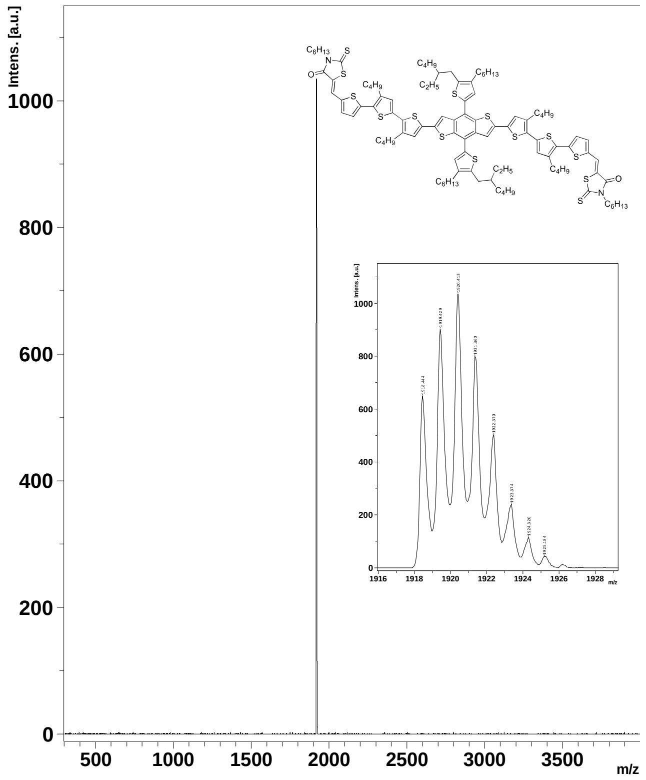

*Figure S4.10. MALDI of **BT^{4}R**, 5'',5'''''-(4,8-bis(5-(2-ethylhexyl)-4-hexylthiophen-2-yl)benzo[1,2-b:4,5-b']dithiophene-2,6-diyl)bis(3',3''-dibutyl-[2,2':5',2''-terthiophene]-5-hexylrhodanine)*

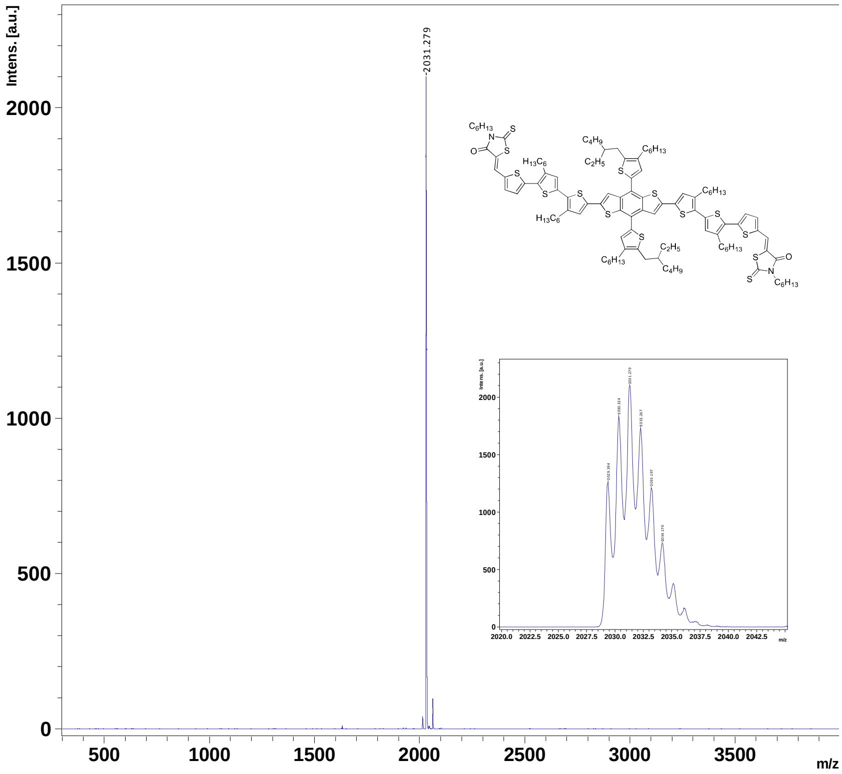

Figure S4.11. MALDI of ***BTR****, (5Z,5'Z)-5,5'-((5'',5''''-(4,8-bis(5-(2-ethylhexyl)-4-hexylthiophen-2-yl)benzo[1,2-b:4,5-b']dithiophene-2,6-diyl)bis(3',3''-dihexyl-[2,2':5',2''-terthiophene]-5'',5-diyl))bis(methanylylidene))bis(3-hexyl-2-thioxothiazolidin-4-one)*

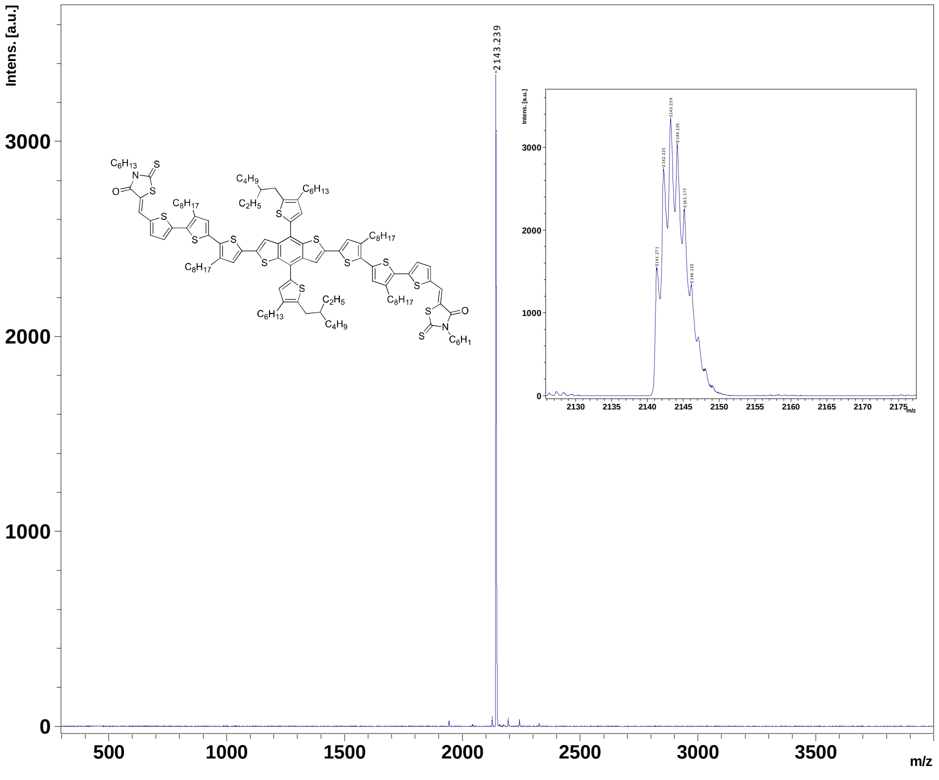

Figure S4.12. MALDI of ***BT⁸R****, 5'',5''''-(4,8-bis(5-(2-ethylhexyl)-4-hexylthiophen-2-yl)benzo[1,2-b:4,5-b']dithiophene-2,6-diyl)bis(3',3''-dioctyl-[2,2':5',2''-terthiophene]-5-hexylrhodanine)*

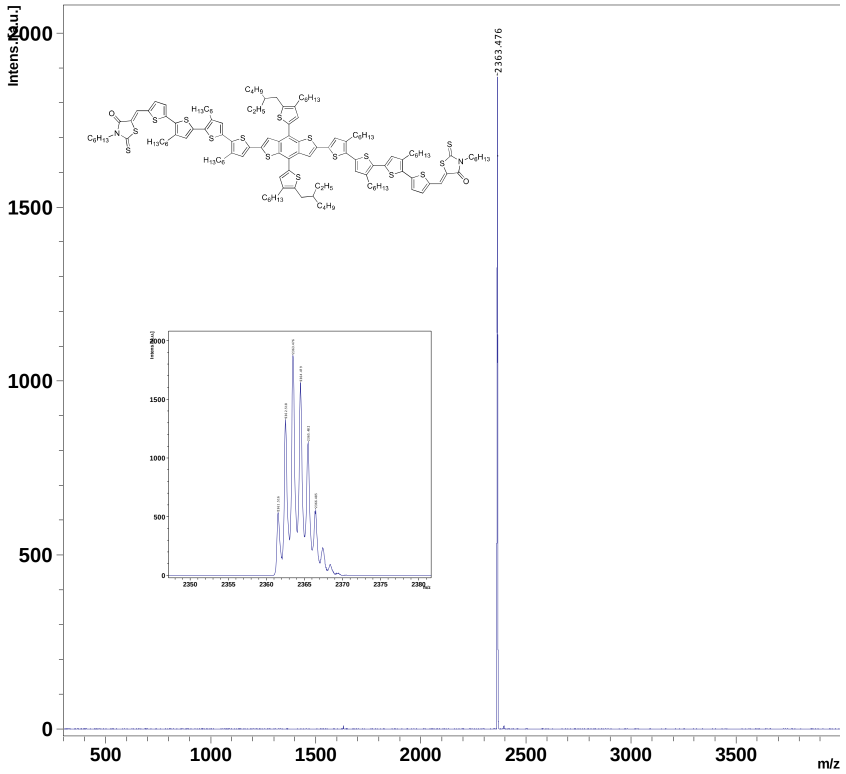

Figure S4.13. MALDI of BQR, (5Z,5'Z)-5,5'-((5''',5''''''-(4,8-bis(5-(2-ethylhexyl)-4-hexylthiophen-2-yl)benzo[1,2-b:4,5-b']dithiophene-2,6-diyl)bis(3',3'',3'''-trihexyl-[2,2':5',2'':5'',2'''-quaterthiophene]-5''',5-diyl))bis(methanylylidene))bis(3-hexyl-2-thioxothiazolidin-4-one)

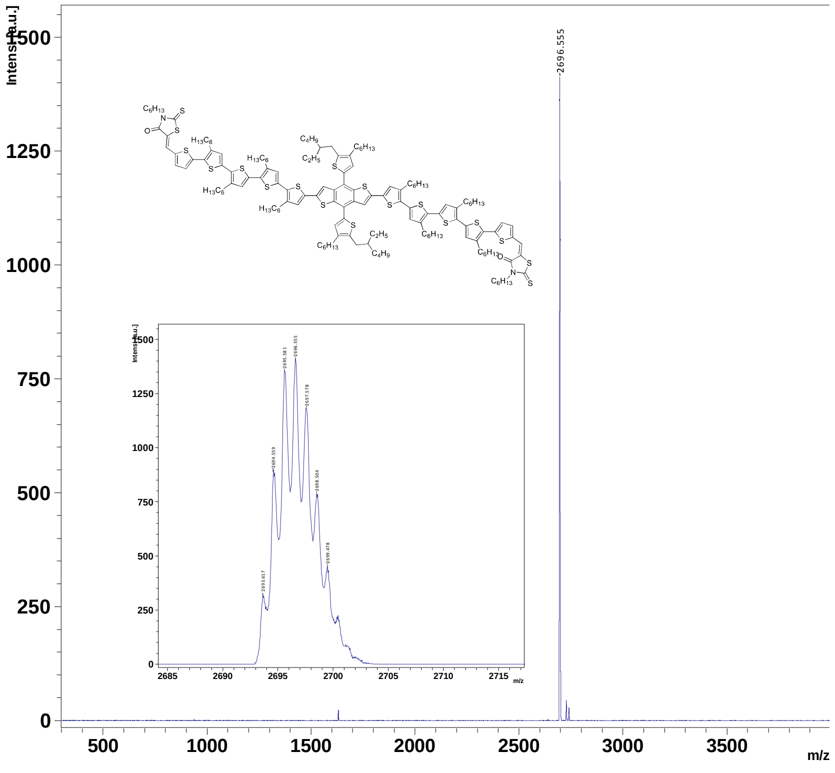

Figure S4.14. MALDI of ***BPR****, (5Z,5'Z)-5,5'-((5'''',5''''''''-(4,8-bis(5-(2-ethylhexyl)-4-hexylthiophen-2-yl)benzo[1,2-b:4,5-b']dithiophene-2,6-diyl)bis(3',3'',3''',3''''-tetrahexyl-[2,2':5',2'':5'',2''':5''',2''''-quinquethiophene]-5'''',5-diyl))bis(methanylylidene))bis(3-hexyl-2-thioxothiazolidin-4-one)*

S5 TGA Thermograms

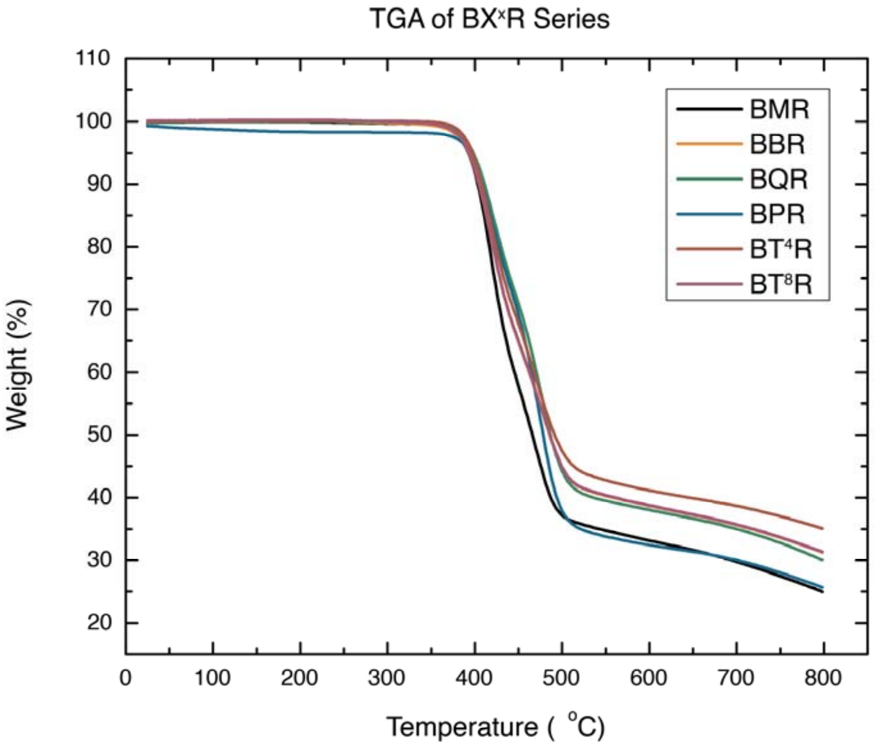

Figure S5.1. *TGA for a)* ***BMR****, and b)* ***BBR****, c)* ***BTR****, d)* ***BQR****, e)* ***BPR****, f)* ***BT^{4}R****, and g)* ***BT^{8}R****.*

S6 DSC thermograms for the BX^{x}R materials

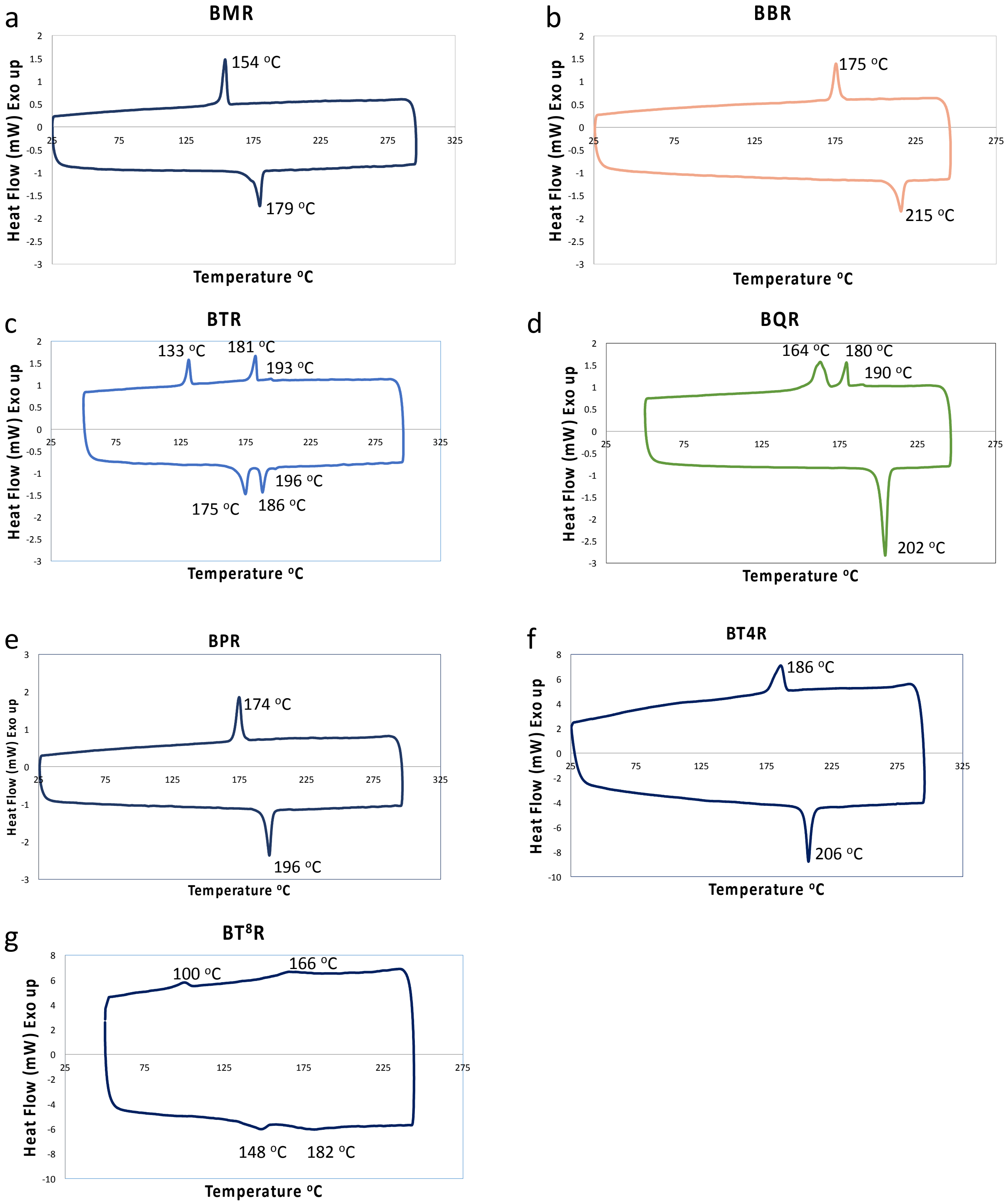

Figure S6.1. *DSC for a)* ***BMR****, and b)* ***BBR****, c)* ***BTR****, d)* ***BQR****, e)* ***BPR****, f)* ***BT^{4}R****, and g)* ***BT^{8}R****.*

S7 Cyclic Voltammograms for the BX^{x}R materials

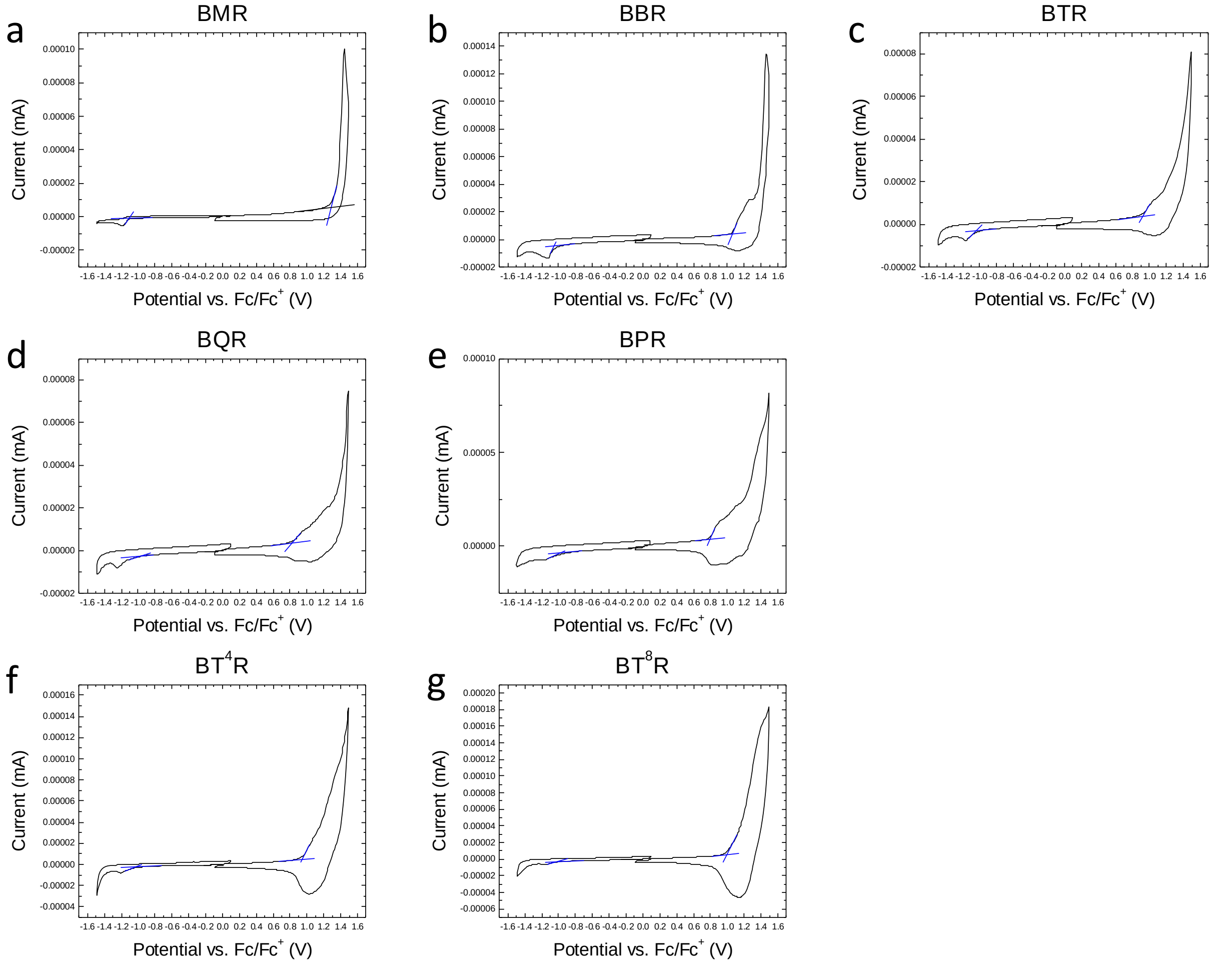

Figure S7.1. Thin film cyclic voltamograms for a) ***BMR****, and b)* ***BBR****, c)* ***BTR****, d)* ***BQR****, e)* ***BPR****, f)* ***BT^{4}R****, and g)* ***BT^{8}R****.*

S8 Polarising optical microscopy (POM) method and images

Methods

A Linkham TMS 94 variable temperature stage was mounted on an Olympus BH2 fluorescence microscope. Samples were mounted using standard glass microscope slides with a cover slip and located in the microscope focus. A high resolution Luminera Infinity 4-11C 10.7 megapixel CCD camera was mounted on the microscope for image capture using the transmission light source of the microscope. Heating profiles were optimised for each sample and are listed in Tables S1-4.

Table S8.1: **BTR sample [Transmission imaging]**					
**Heating process**			**Cooling Process**		
Temperature °C	Temperature °C.min^{-1}	Temperature hold/minutes	Temperature °C	Temperature °C.min^{-1}	Temperature hold/minutes
25	1	1	210	1	5
174	1	10	194	1	25
185	1	15	182	1	20
195	1	20	134	1	10
197	1	10	25	1	1
210	1	5			

Table S8.2: **BQR sample [Transmission imaging]**					
**Heating process**			**Cooling Process**		
Temperature °C	Temperature °C.min^{-1}	Temperature hold/minutes	Temperature °C	Temperature °C.min^{-1}	Temperature hold/minutes
25	1	1	210	1	5
200	1	1	190	1	15
203	1	15	180	0.1	15
210	1	5	174	0.1	15
			164	1	10
			25	1	1

Table S8.3: **BT^{4}R sample [Transmission imaging]**					
**Heating process**			**Cooling Process**		
Temperature °C	Temperature °C.min-1	Temperature hold/minutes	Temperature °C	Temperature °C.min^{-1}	Temperature hold/minutes
25	10	1	210	1	5
205	1	10	200	1	10
206	1	10	198.5	1	10
210	1	5	193.5	1	10
			25	10	1

Table S8.4: **BT^{8}R sample [Transmission imaging]**					
**Heating process**			**Cooling Process**		
Temperature ^{0}C	Temperature ^{0}C.min-1	Temperature hold/minutes	Temperature ^{0}C	Temperature ^{0}C.min-1	Temperature hold/minutes
25	10	1	210	1	5
145	1	10	172	1	10
150	1	10	100	1	10
182	1	5	25	1	1
198	1	10			
210	1	5			

Spectroscopic Measurements

To obtain variable temperature UV-Vis and fluorescence measurements an Ocean Optics HR2000 fibre-optic spectrometer was connected to the Olympus BH-2 fluorescence microscope at the c-mount adapter instead of the CCD colour camera. The spectra therefore integrate all the light transmitted (or emitted) through the field of view. The temperature profiles used for UV-Vis and fluorescence spectra are listed in Table S5 and S6. Emission images and spectra were recorded using the Hg fluorescence excitation source of the microscope.

Table S8.5: **BTR sample [Absorption measurements]**					
**Heating process**			**Cooling Process**		
Temperature ^{0}C	Temperature ^{o}C.min^{-1}	Temperature hold/min.	Temperature ^{0}C	Temperature ^{o}C.min^{-1}	Temperature hold/min.
25	1	1	210	1	1
134	1	1	197	1	1
174	1	1	195	1	1
182	1	1	194	1	1
185	1	1	185	1	1
194	1	1	182	1	1
195	1	1	174	1	1
197	1	1	134	1	1
210	1	1	25	1	1

Table S8.6: **BQR sample [Absorption measurements]**					
**Heating process**			**Cooling Process**		
Temperature ^{0}C	Temperature ^{o}C.min^{-1}	Temperature Hold min.	Temperature ^{0}C	Temperature ^{o}C.min^{-1}	Temperature Hold min.
25	1	1	210	1	1
164	1	1	203	1	1
174	1	1	200	1	1
180	1	1	190	1	1
190	1	1	180	0.1	1
200	1	1	174	0.1	1
203	1	1	164	1	1
210	1	1	25	1	1

The microscopy imaging and spectral experiments were preformed using a 20X 0.40 NA microscope objective lens (Olympus, ULWD MSPlan20), and the magnification was further increased 1.25X by the fluorescence reflection unit extension.

The settings for all experiments such as; microscopic power, imaging exposure time and spectrometric integration time and others, were set and can be seen in the Table S7.

Table S8.7: **BTR/BQR sample [Transmission Imaging settings]**			
Microscopic power	Exposure time	Gain	Others
8	300 milliseconds	1.5	Default
**BTR/BQR sample [Fluorescence Imaging settings]**			
Microscopic power	Exposure time	Gain	Others
0 blocked	300 milliseconds	1.5	Default
**BTR/BQR sample [Absorption measurement settings]**			
Microscopic power	Integration time	Scan to average	Boxcar width
< 3	100 milliseconds	25	10
**BTR/BQR sample [Emission measurement settings]**			
Microscopic power	Integration time	Scan to average	Boxcar width
0 blocked	100 milliseconds	50	10

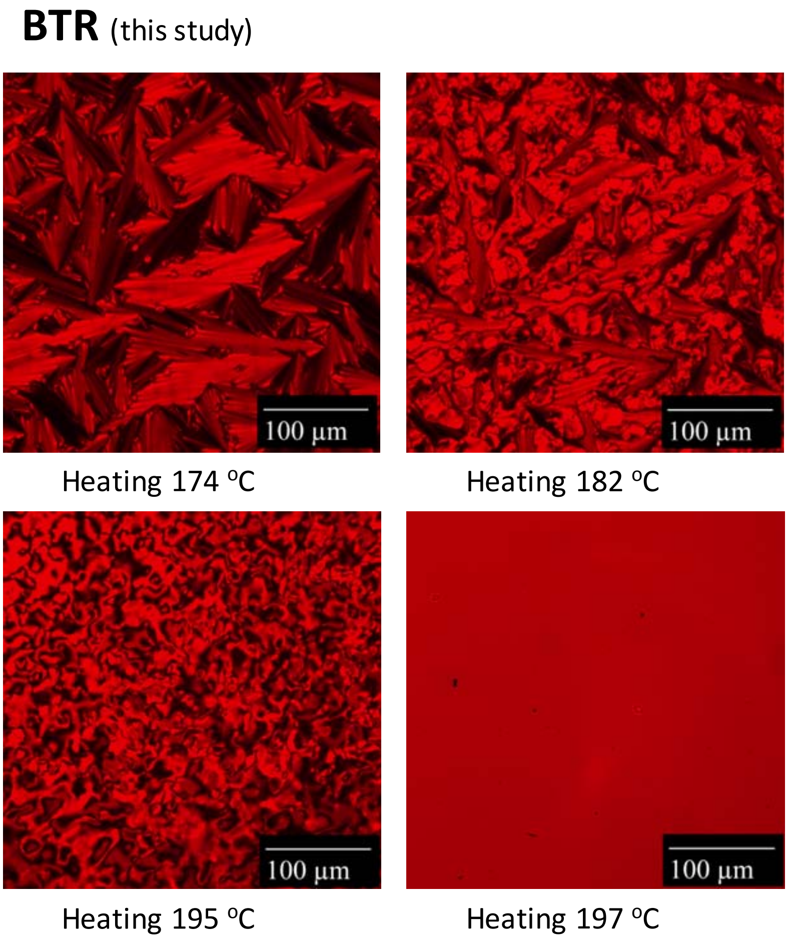

Figure S8.1. Selected POM images for BTR, Heating cycle.

BTR (this study)

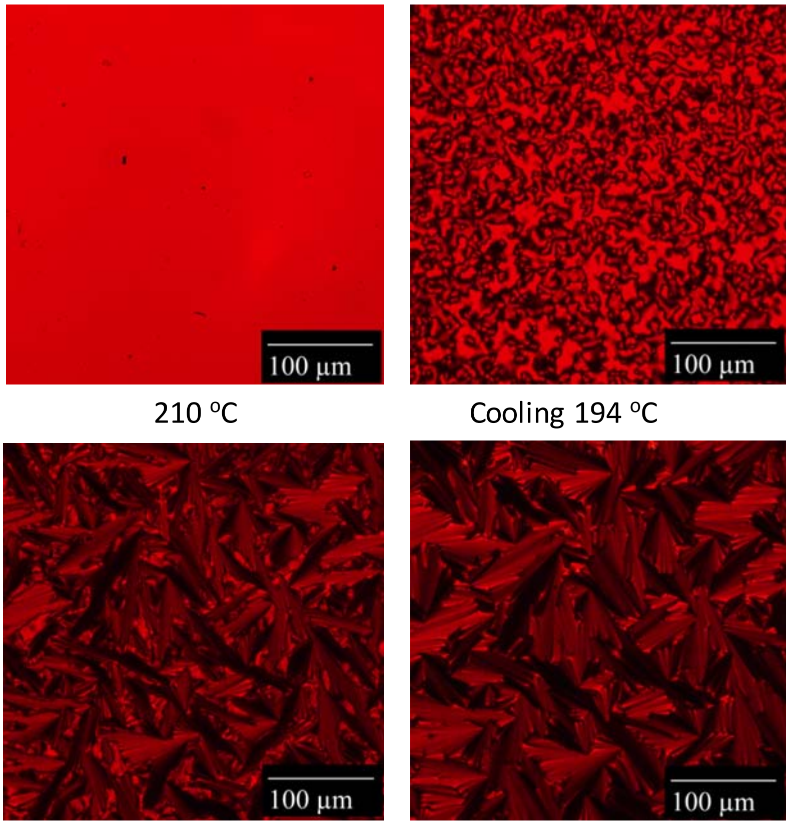

210 °C | Cooling 194 °C

Cooling 179.6 °C | Cooling 174 °C

Figure S8.2. Selected POM images for BTR, cooling cycle.

BQR

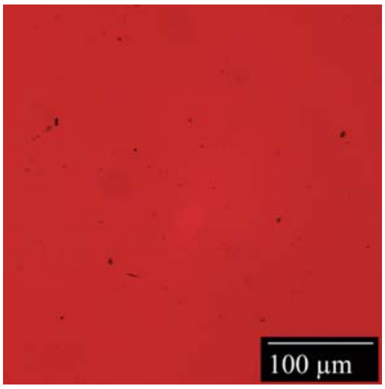

203 °C

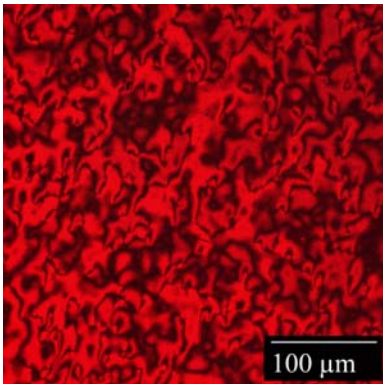

Cooling 190 °C

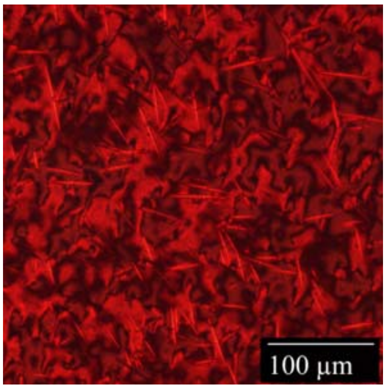

Cooling 183 °C

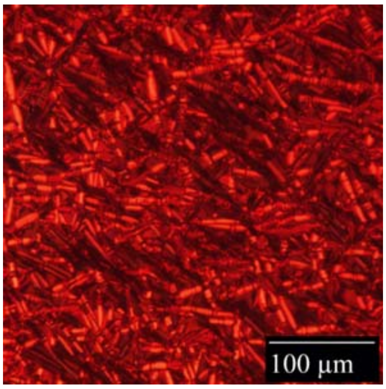

Cooling 183 °C

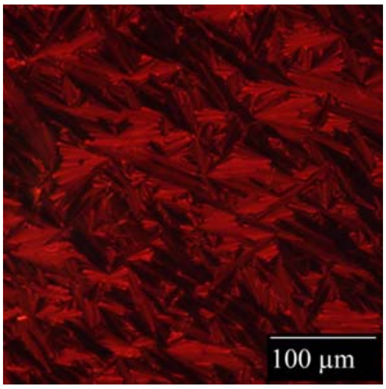

Cooling 179 °C

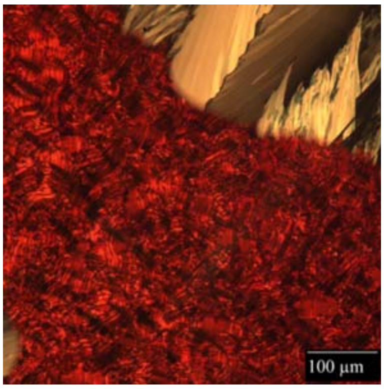

Cooling 173.5 °C

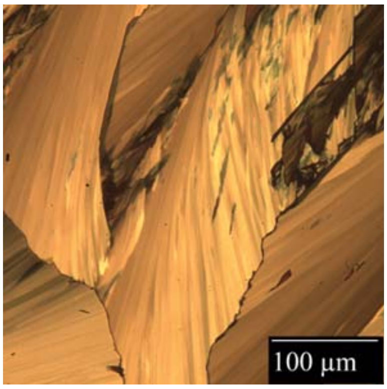

Cooling 172 °C

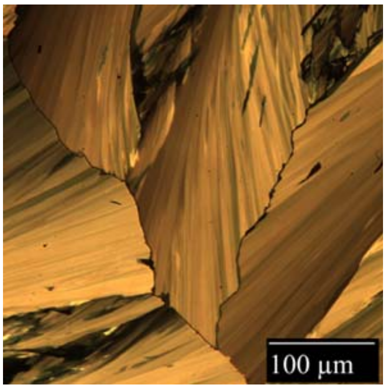

Cooling 164 °C

Figure S8.3. Selected POM images for BQR, cooling cycle.

BT^{4}R

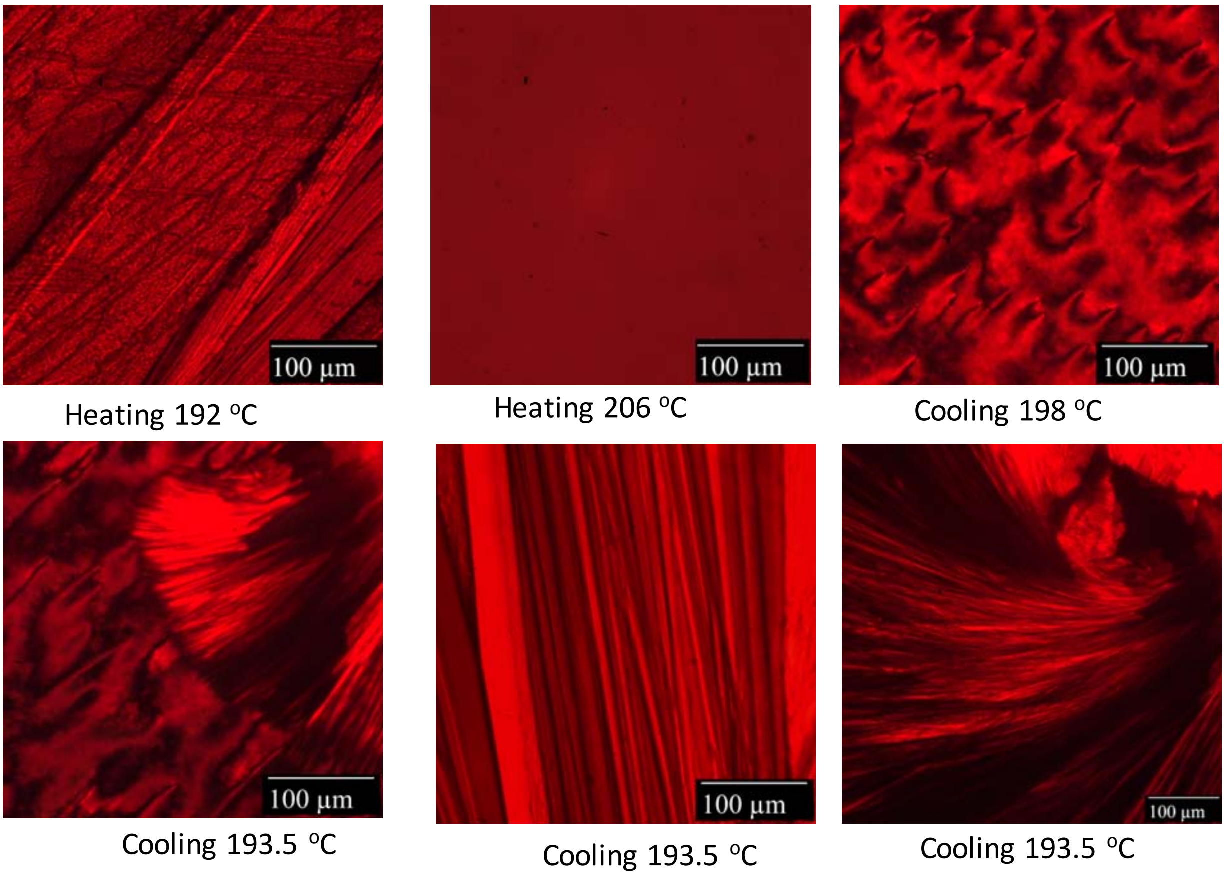

Figure S8.4. Selected POM images for BT^{4}R, heating and cooling cycles.

BT^8R

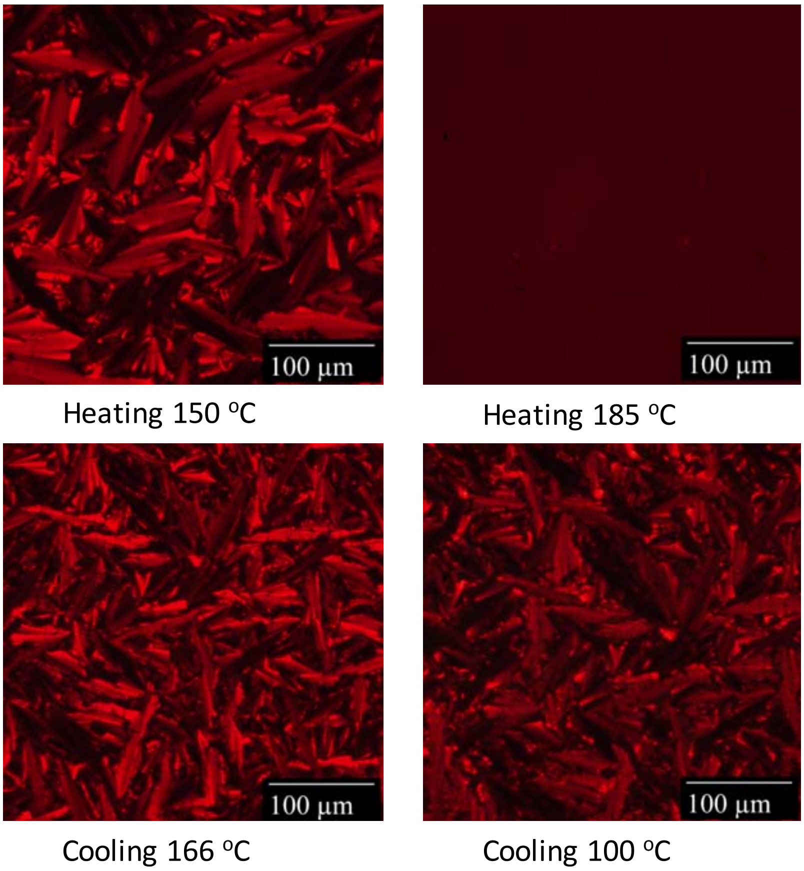

Figure S8.5. Selected POM images for BT^8R, heating and cooling cycles.

S9 UV–vis spectra for the BXR and BTxR series of materials

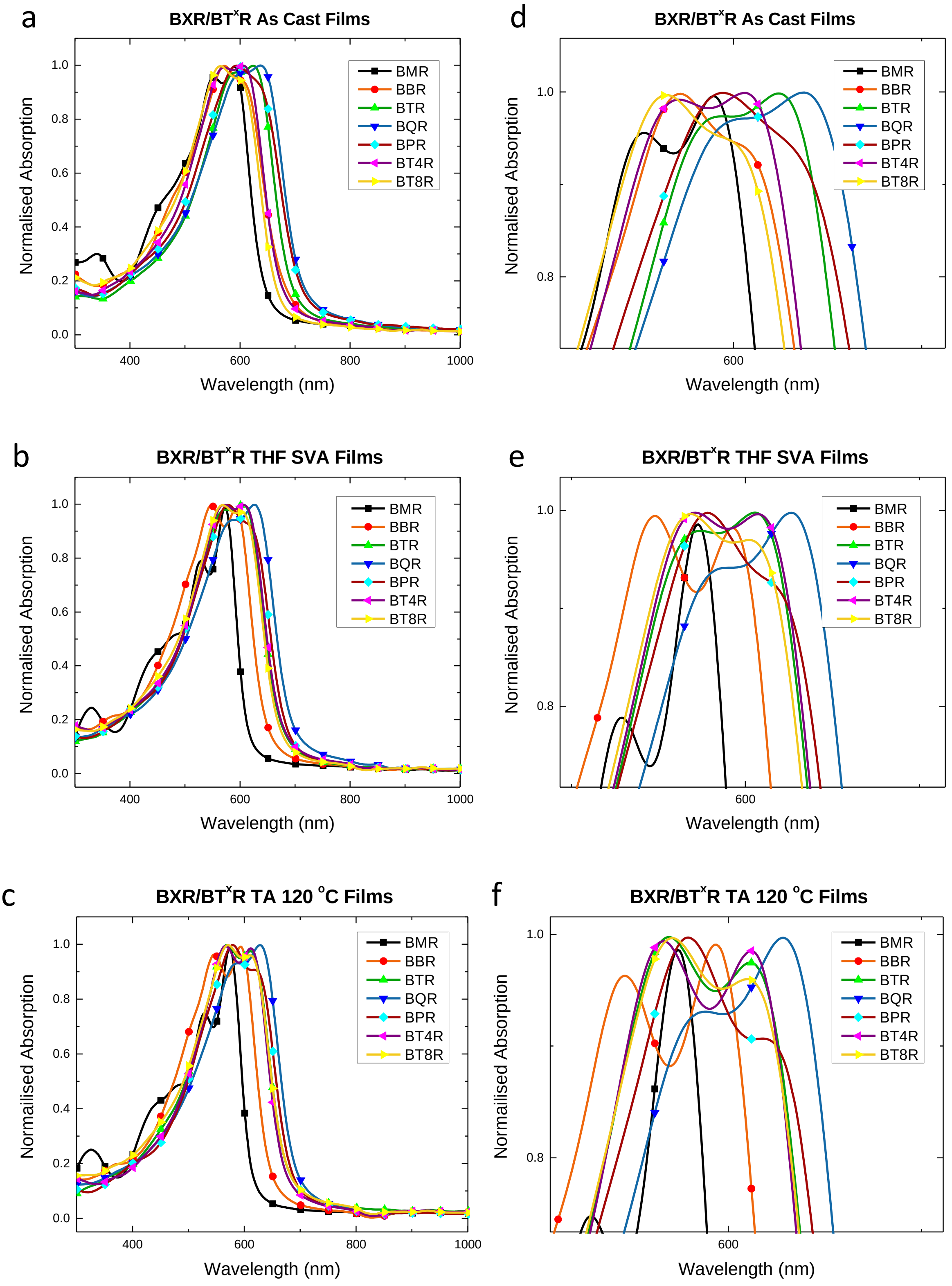

Figure S9.1. *Collated UV-Vis spectra for the* ***BX^{x}R*** *series, a) as-cast, b)solvent vapour annealed (SVA) with THF for 10 sec, c) thermally annealed (TA) at 120 °C for 10 minutes, d) as-cast expansion of peak area, e) SVA expansion of peak area, f) TA expansion of peak area.*

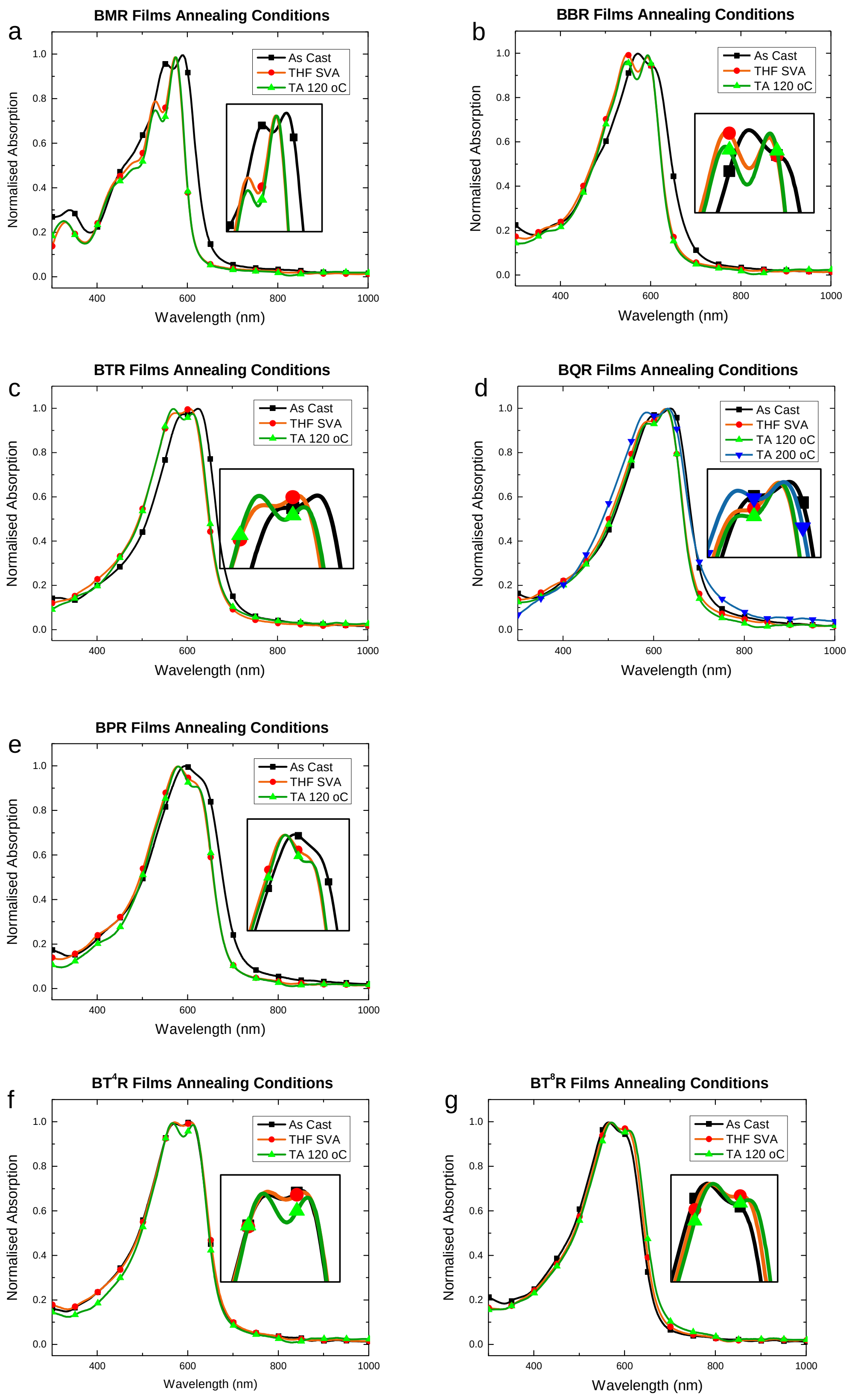

Figure S9.2. UV-Vis spectra for the individual ***BX^{x}R*** *material series showing as-cast, SVA and TA for each material with an inset showing an expansion of the peak area for, a)* ***BMR****, b)* ***BBR****, c)* ***BTR****, d)* ***BQR****, e)* ***BPR****, f)* ***BT^{4}R****, g)* ***BT^{8}R***

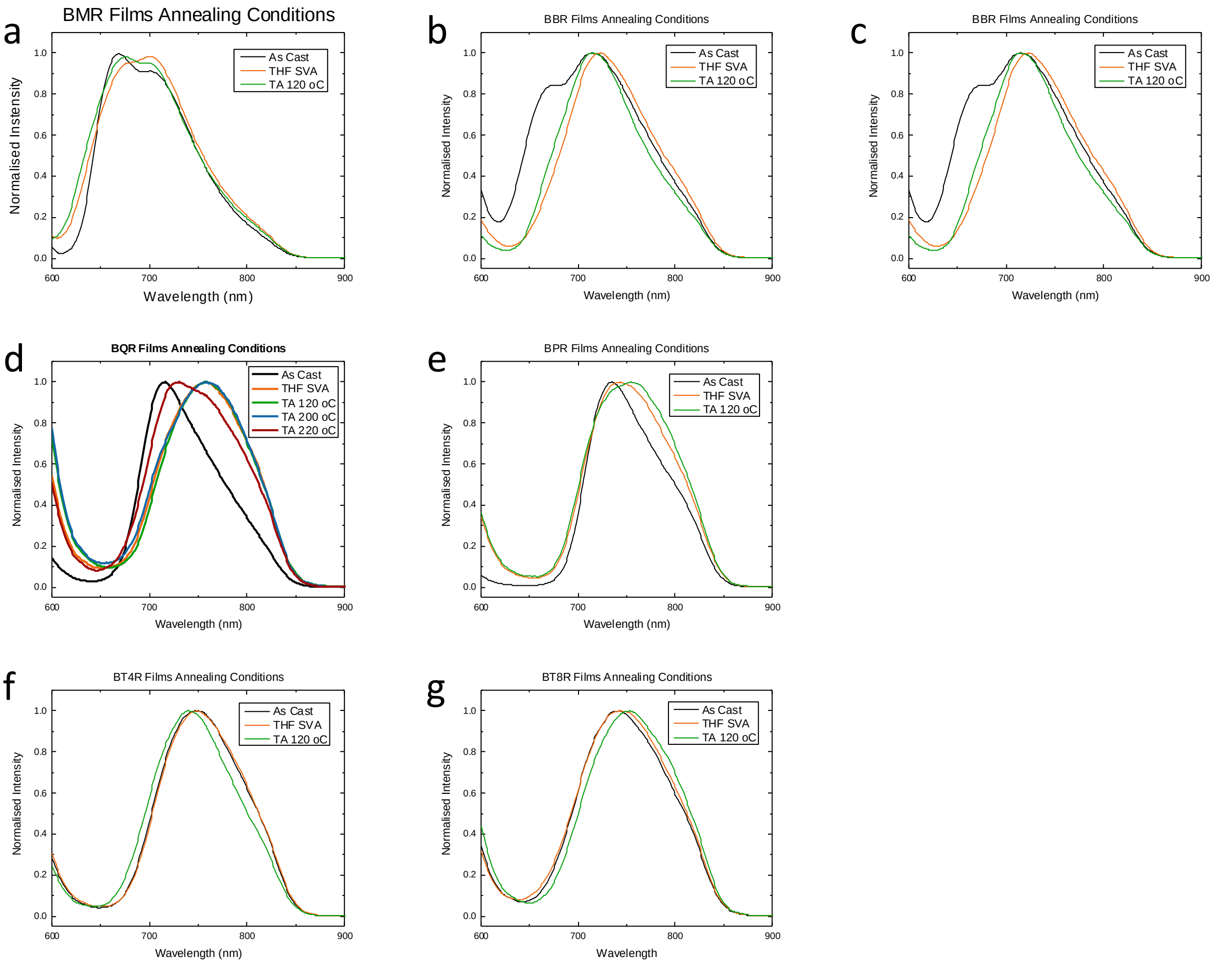

Figure S9.3. Fluorescence emission spectra for the individual ***BX^{x}R*** *material series showing as-cast, SVA and TA for each material, a)* ***BMR****, b)* ***BBR****, c)* ***BTR****, d)* ***BQR****, e)* ***BPR****, f)* ***BT^{4}R****, g)* ***BT^{8}R****.*

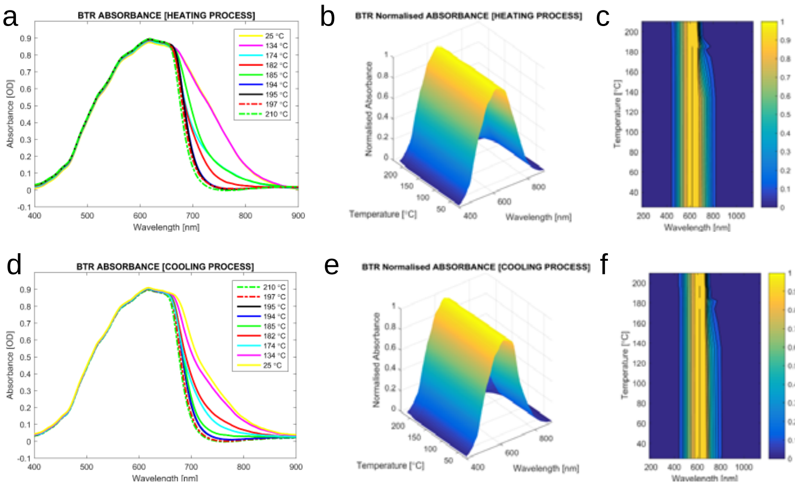

*Figure S8.1. Variable Temperature Uv-Vis spectra for **BTR**, Heating cycle, a) 2-D selected temperatures, b) 3-D temperature series, and c) 2-D intensity plot, and Cooling cycle d) 2-D selected temperatures, e) 3-D temperature series, and f) 2-D intensity plot.*

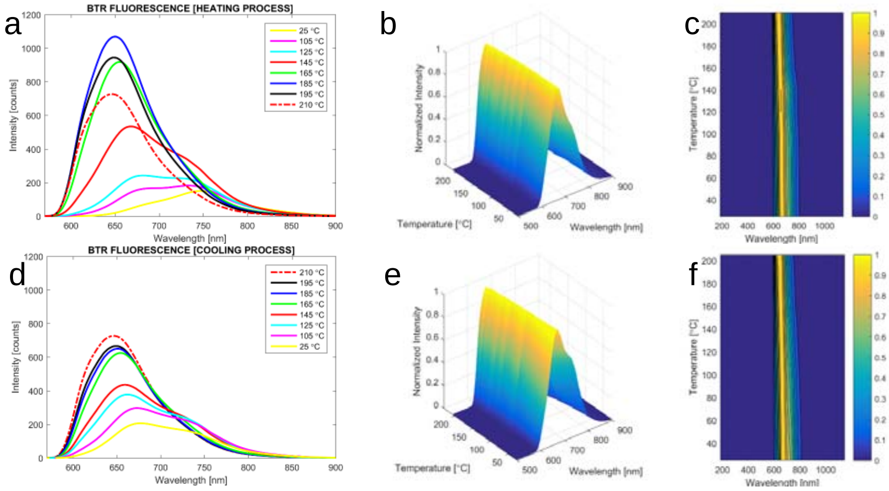

*Figure S8.2. Variable Temperature fluorescence spectra for **BTR**, Heating cycle, a) 2-D selected temperatures, b) 3-D temperature series, and c) 2-D intensity plot, and Cooling cycle d) 2-D selected temperatures, e) 3-D temperature series, and f) 2-D intensity plot.*

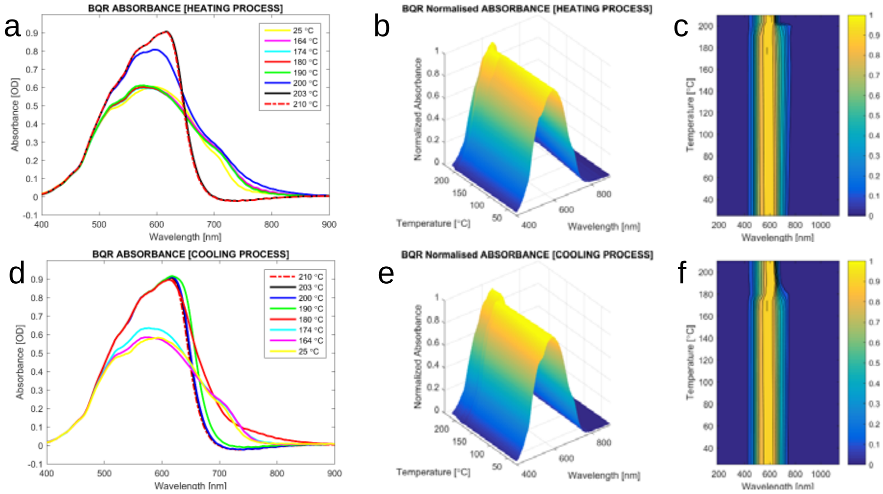

*Figure S8.3. Variable Temperature Uv-Vis spectra for **BQR**, Heating cycle, a) 2-D selected temperatures, b) 3-D temperature series, and c) 2-D intensity plot, and Cooling cycle d) 2-D selected temperatures, e) 3-D temperature series, and f) 2-D intensity plot.*

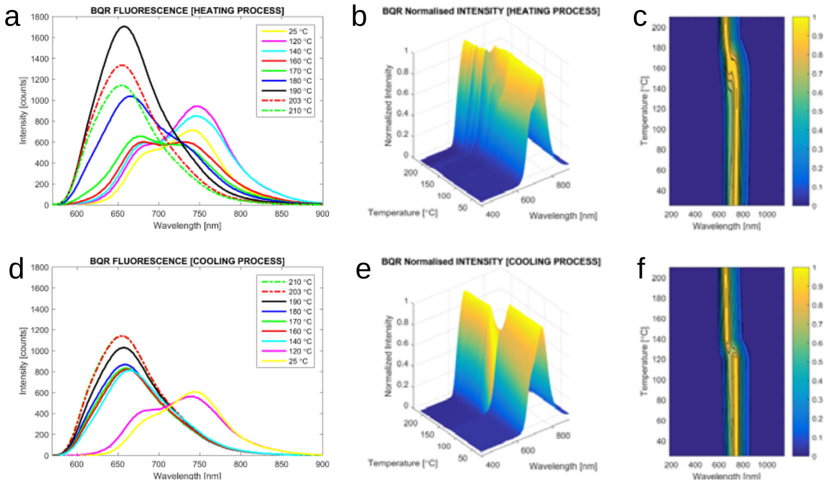

Figure S8.4. Variable Temperature fluorescence spectra for ***BQR****, Heating cycle, a) 2-D selected temperatures, b) 3-D temperature series, and c) 2-D intensity plot, and Cooling cycle d) 2-D selected temperatures, e) 3-D temperature series, and f) 2-D intensity plot.*

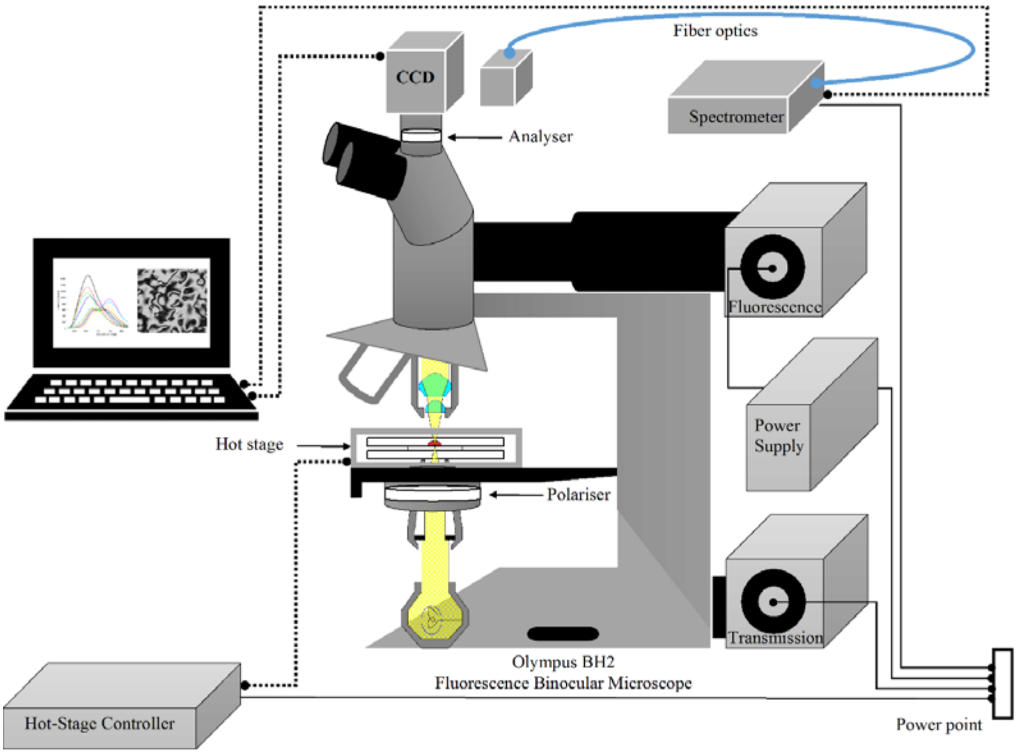

Figure S8.5. POM and variable temperature UV-Vis and fluorescence equipment setup used in this study.

S10 Photoelectron spectroscopy in air (PESA) measurements for the BXR series of materials

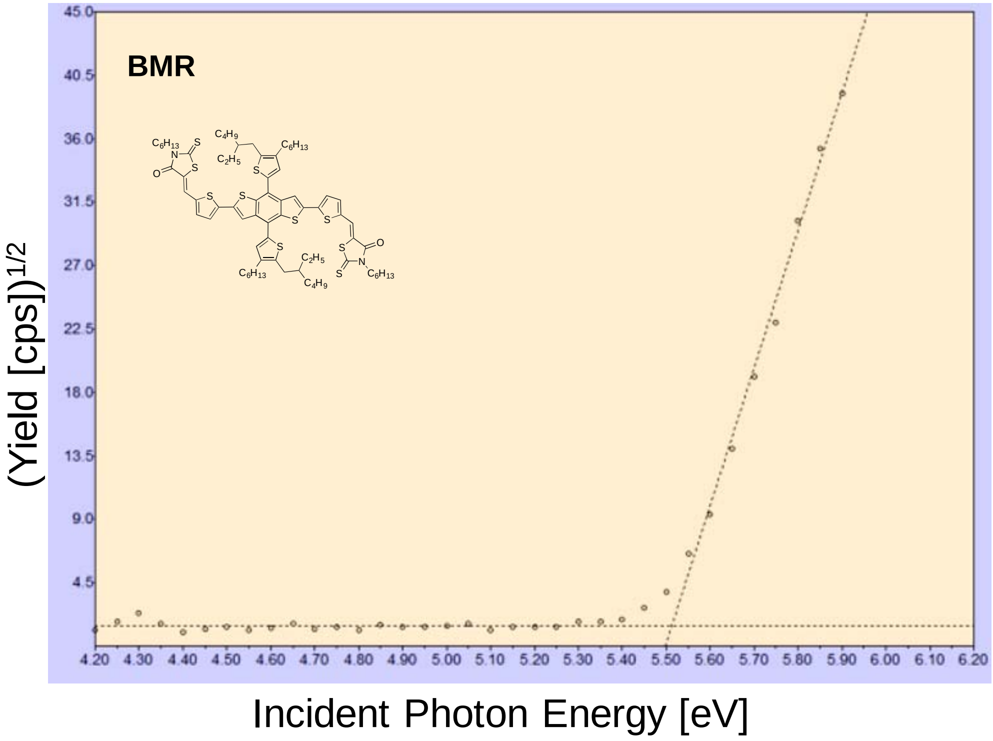

Figure S10.1. PESA of BMR

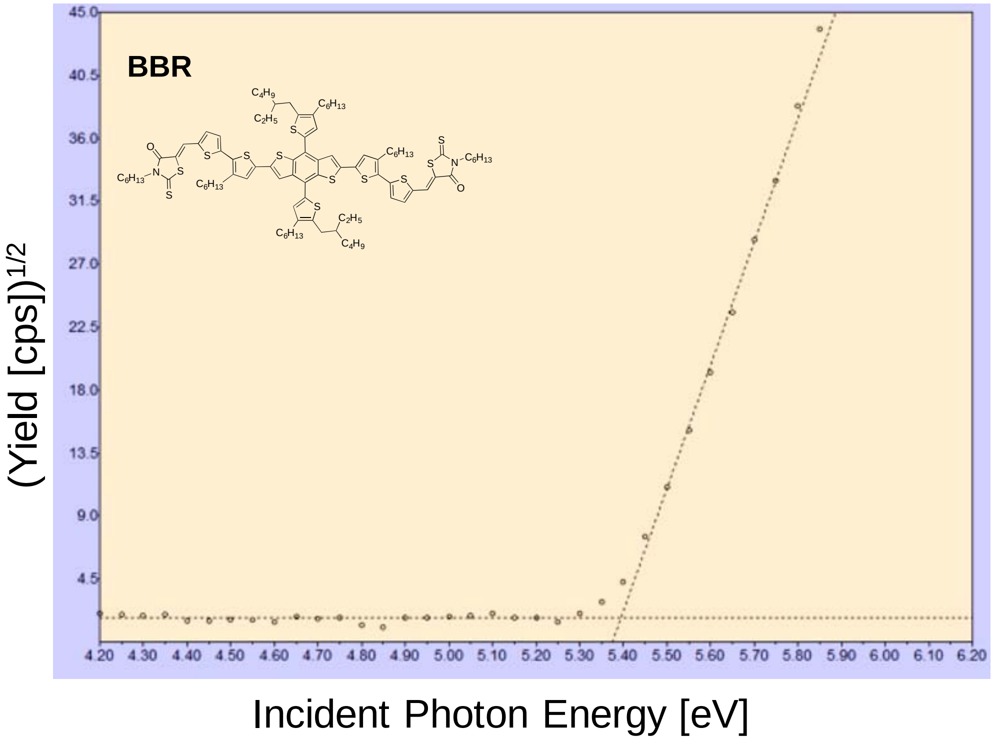

Figure S10.2. PESA of BBR

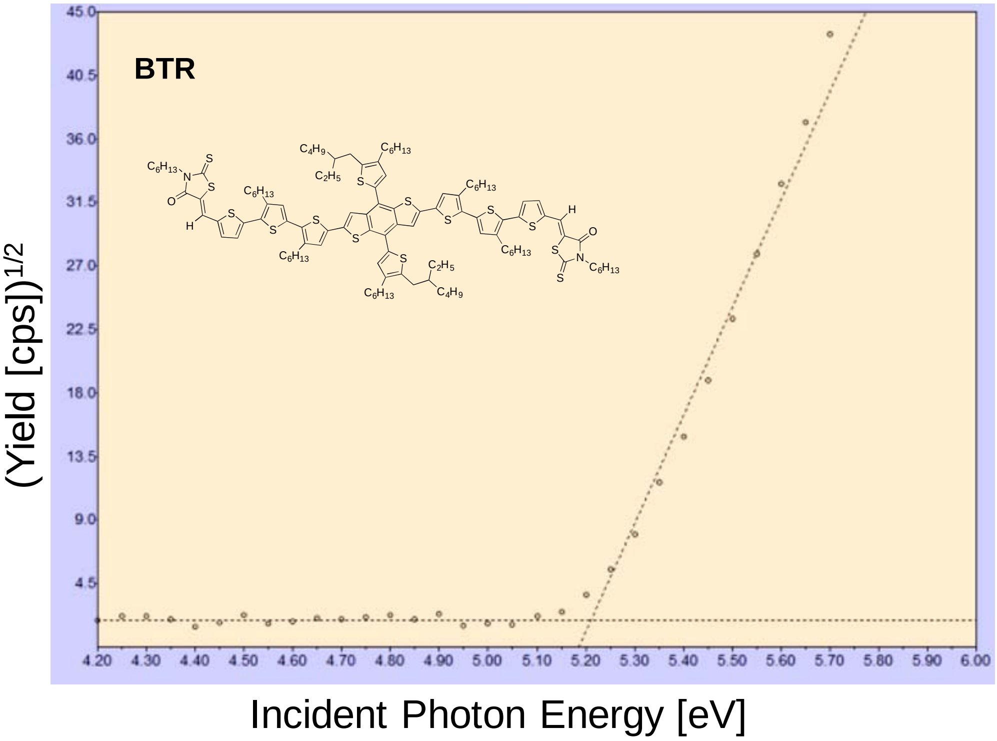

Figure S10.3. PESA of BTR

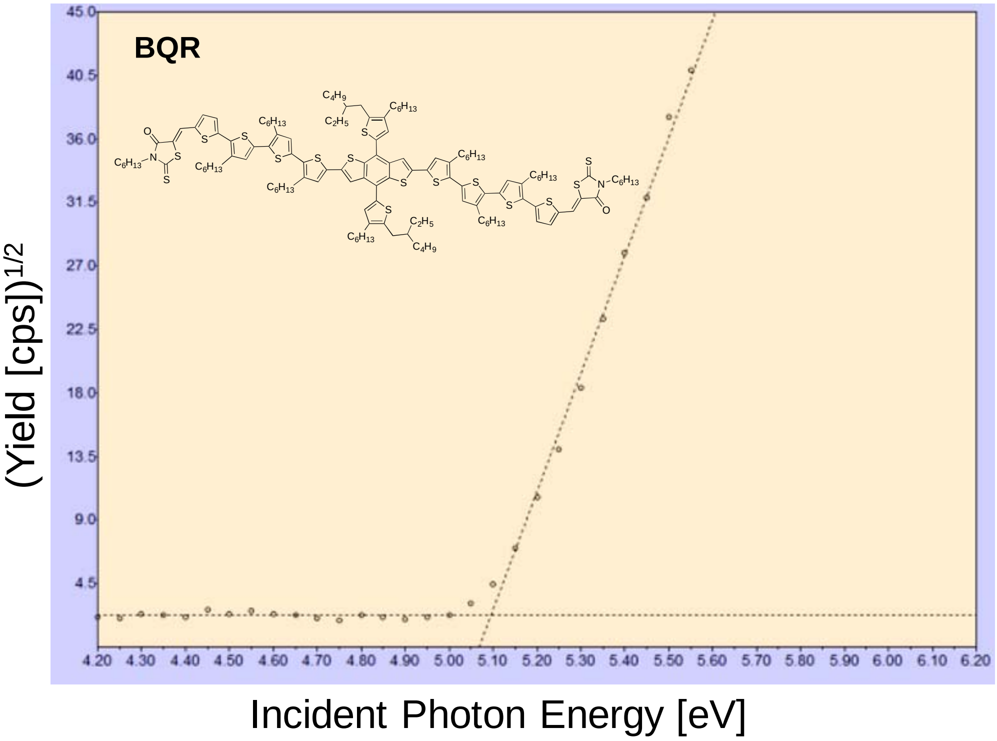

Figure S10.4. PESA of BQR

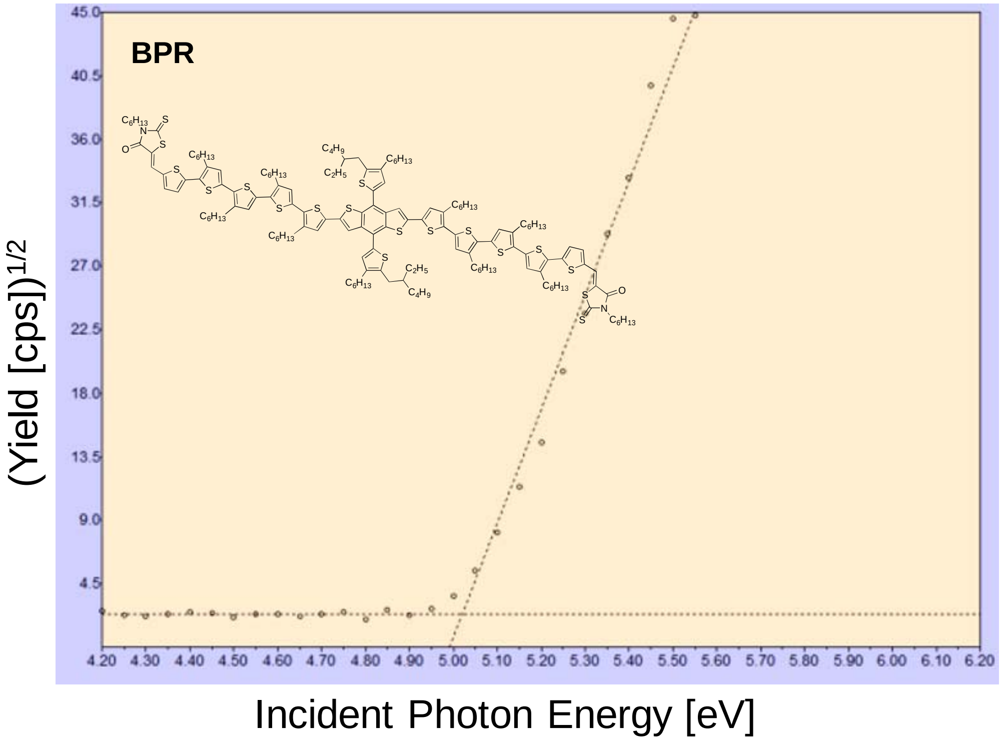

Figure S10.5. PESA of BPR

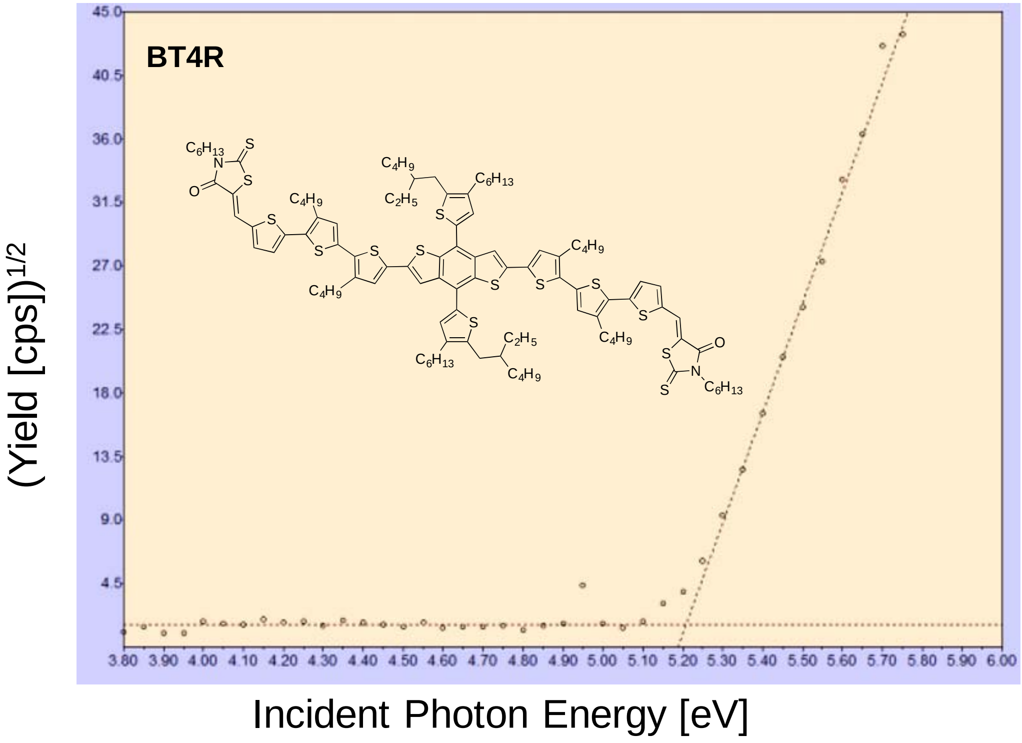

Figure S10.6. PESA of BT^4R

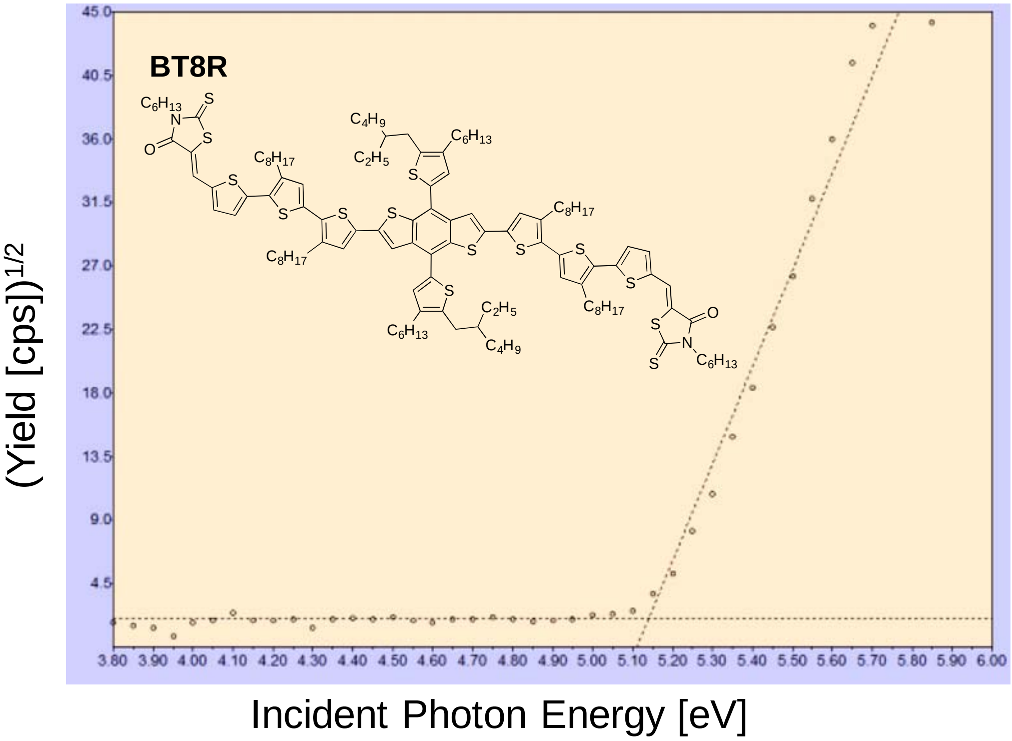

Figure S10.7. PESA of BT^{8}R

S11 DFT Calculations

All calculations were determined using using Gaussian 09 Rev. B.01.[1] The geometry optimization was performed using 6-311G(d,p) basis set and B3LYP functional with D2 correction to account for dispersion. The Cartesian coordinates are shown below for the BXR series.

Section S11.1 Cartesian Coordinates

Section S11.2 BMR

C	1.40131	0.02678	0.1131
C	0.67649	-1.1997	0.10995
C	-0.71683	-1.26386	0.10164
C	-1.40133	-0.02649	0.11196
C	-0.67652	1.19999	0.10993
C	0.71681	1.26416	0.10279
C	2.8114	-0.18774	0.13244
C	3.175	-1.50605	0.11493
S	1.76822	-2.57981	0.08343
C	-2.81144	0.18797	0.13009
C	-3.17507	1.50627	0.11283
S	-1.76827	2.58008	0.08317
C	1.42232	2.55107	0.08764
C	-1.42233	-2.55076	0.08538
C	1.24923	3.61187	0.93418
C	2.05822	4.74974	0.63036
C	2.84879	4.54964	-0.47197
S	2.6007	2.95658	-1.14349
C	-1.25028	-3.61172	0.93192
C	-2.05895	-4.7495	0.62693
C	-2.84822	-4.54916	-0.47629
S	-2.59928	-2.95596	-1.14721
C	3.85427	5.47618	-1.08782
C	2.04425	5.99863	1.4697
C	-3.85301	-5.47557	-1.09345
C	-2.04612	-5.99857	1.46603
C	-4.50662	2.05443	0.1284
C	4.50652	-2.05426	0.13156
C	4.88836	-3.38257	0.21436
C	6.27989	-3.56706	0.2112
C	7.00365	-2.38752	0.12607
S	5.91124	-1.015	0.04108
C	-4.88851	3.38293	0.20794
C	-6.28006	3.56736	0.20447
C	-7.00378	2.38759	0.12234

S	-5.91132	1.01489	0.04087
C	8.42619	-2.30764	0.10904
C	-8.42631	2.30761	0.10554
C	-9.25421	1.23694	0.03264
C	9.25414	-1.23718	0.03371
C	10.71943	-1.42883	0.0361
N	11.36649	-0.17801	-0.04886
C	10.57899	0.93843	-0.11574
S	8.86075	0.47949	-0.07441
C	-10.71951	1.42851	0.03458
N	-11.3665	0.17747	-0.04763
C	-10.57895	-0.93907	-0.11204
S	-8.86073	-0.47995	-0.07165
C	12.82594	-0.0916	-0.06457
O	11.31016	-2.48492	0.10044
S	11.08179	2.5034	-0.22087
S	-11.08167	-2.50429	-0.21374
O	-11.3103	2.48471	0.09657
C	-12.82595	0.09097	-0.0632
H	3.52819	0.6204	0.16909
H	-3.52821	-0.62025	0.16546
H	0.56506	3.57786	1.77291
H	-0.56704	-3.57793	1.77141
H	3.64364	6.50805	-0.79751
H	3.83634	5.42308	-2.18033
H	4.87261	5.23757	-0.75935
H	2.65175	5.87337	2.3737
H	2.4384	6.85841	0.92349
H	1.02506	6.23728	1.78824
H	-3.64284	-6.50746	-0.80288
H	-4.87174	-5.23688	-0.76628
H	-3.83368	-5.42244	-2.18594
H	-2.65607	-5.87404	2.36848
H	-2.43814	-6.85855	0.91859
H	-1.02756	-6.23645	1.78713
H	4.17148	-4.19039	0.28081
H	6.76705	-4.53183	0.27212
H	-4.17168	4.19098	0.27206
H	-6.76724	4.53228	0.26293
H	8.94031	-3.2644	0.1668
H	-8.94048	3.26446	0.16113
H	13.15312	0.38925	-0.98835
H	13.21061	-1.10822	0.00068
H	13.16517	0.50746	0.78259
H	-13.21066	1.10771	-0.00011
H	-13.16517	-0.50632	0.78521

H	-13.15309	-0.39185	-0.98598

Section S11.3 BBR

C	1.309	-0.50544	0.3395
C	1.04684	0.89427	0.34298
C	-0.24085	1.43131	0.35127
C	-1.30902	0.50548	0.33959
C	-1.04686	-0.89424	0.34312
C	0.24083	-1.43127	0.35132
C	2.70888	-0.78667	0.33026
C	3.50102	0.32723	0.34646
S	2.54551	1.81732	0.35874
C	-2.7089	0.7867	0.33042
C	-3.50104	-0.3272	0.34673
S	-2.54553	-1.81729	0.35904
C	0.45883	-2.88305	0.36973
C	-0.45885	2.88309	0.36962
C	-0.06777	-3.81998	-0.4762
C	0.28529	-5.16851	-0.16073
C	1.08453	-5.25313	0.94978
S	1.40792	-3.67001	1.6145
C	0.0677	3.81998	-0.47639
C	-0.28536	5.16852	-0.16099
C	-1.08455	5.2532	0.94956
S	-1.40789	3.67011	1.61439
C	1.68986	-6.47122	1.58137
C	-0.16389	-6.33653	-0.99645
C	-1.68983	6.47131	1.58116
C	0.16376	6.33648	-0.99682
C	-4.94031	-0.39379	0.34256
C	4.94029	0.39381	0.34223
C	5.74696	1.49543	0.5236
C	7.13603	1.23239	0.47176
C	7.40949	-0.11026	0.24732
S	5.91826	-1.0296	0.07561
C	-5.74697	-1.49543	0.52387
C	-7.13605	-1.2324	0.47203
C	-7.40952	0.11026	0.24766
S	-5.9183	1.02963	0.07601
C	8.66525	-0.80831	0.15166
C	-8.66529	0.8083	0.15207
C	8.87271	-2.17876	0.24321
C	10.209	-2.56227	0.07336
C	11.07665	-1.5037	-0.15196
S	10.1853	0.00853	-0.1597

C	-8.87281	2.17871	0.24397
C	-10.2091	2.56222	0.07406
C	-11.07668	1.50367	-0.15167
S	-10.18527	-0.00852	-0.15973
C	-12.48256	1.6312	-0.33648
C	-13.44167	0.69694	-0.55091
C	12.48254	-1.63125	-0.33669
C	13.44169	-0.69698	-0.5509
C	8.16123	2.31474	0.66899
C	-8.16124	-2.31477	0.66923
S	13.28993	1.05689	-0.67877
C	15.03735	1.25505	-0.94651
N	15.65647	0.03563	-0.93167
C	14.85104	-1.1035	-0.71724
S	-13.28985	-1.0569	-0.67905
C	-15.03726	-1.25509	-0.94685
N	-15.65641	-0.03569	-0.93185
C	-14.85102	1.10344	-0.71725
C	17.09896	-0.09655	-1.12946
S	15.74515	2.72555	-1.17293
O	15.28776	-2.23382	-0.68225
C	-17.09889	0.09648	-1.12968
S	-15.745	-2.72557	-1.17351
O	-15.28777	2.23375	-0.68215
H	3.10704	-1.79142	0.31914
H	-3.10707	1.79145	0.31924
H	-0.69122	-3.55213	-1.32014
H	0.69112	3.55208	-1.32034
H	1.13569	-7.3647	1.28476
H	1.67151	-6.41113	2.67363
H	2.73315	-6.60751	1.27273
H	-1.20308	-6.20601	-1.31308
H	-0.09295	-7.27881	-0.44863
H	0.44675	-6.43204	-1.90201
H	-1.13614	7.36486	1.28386
H	-1.67069	6.41159	2.67343
H	-2.73337	6.60717	1.27321
H	1.20284	6.2058	-1.31373
H	0.09311	7.27877	-0.449
H	-0.44711	6.43207	-1.90222
H	5.34922	2.4847	0.71255
H	-5.34922	-2.4847	0.71275
H	8.06804	-2.87328	0.44489
H	10.55972	-3.58531	0.1232
H	-8.06821	2.8732	0.44596
H	-10.55987	3.58523	0.12416

H	-12.8576	2.6517	-0.30306
H	12.85755	-2.65176	-0.3034
H	7.67557	3.23259	1.00727
H	8.69286	2.54029	-0.26204
H	8.91081	2.02916	1.41315
H	-7.6756	-3.23256	1.00768
H	-8.69272	-2.54044	-0.26186
H	-8.91094	-2.02913	1.41325
H	17.37372	0.32219	-2.09936
H	17.33548	-1.15854	-1.084
H	17.62719	0.45264	-0.34769
H	-17.62714	-0.45291	-0.34805
H	-17.3736	-0.32205	-2.09968
H	-17.33546	1.15845	-1.08398

Section S11.4 BTR

C	1.39247	-0.17821	-0.6155
C	0.80311	1.11797	-0.61968
C	-0.5756	1.33177	-0.62866
C	-1.39255	0.1784	-0.61571
C	-0.80319	-1.11777	-0.6197
C	0.57552	-1.33157	-0.62845
C	2.81929	-0.11662	-0.60746
C	3.32221	1.15412	-0.6245
S	2.03791	2.37266	-0.6337
C	-2.81937	0.11679	-0.60783
C	-3.32226	-1.15396	-0.6248
S	-2.03794	-2.37247	-0.63372
C	1.13216	-2.69025	-0.64876
C	-1.13222	2.69045	-0.6492
C	0.85866	-3.72026	0.20808
C	1.53308	-4.94255	-0.09969
C	2.32567	-4.83593	-1.21288
S	2.243	-3.22802	-1.89242
C	-0.85866	3.72065	0.20738
C	-1.53305	4.94289	-0.10063
C	-2.32569	4.83604	-1.21376
S	-2.24312	3.228	-1.89295
C	3.16333	-5.88819	-1.87687
C	1.35496	-6.18874	0.72453
C	-3.16336	5.88816	-1.87796
C	-1.35485	6.18922	0.72335
C	-4.70409	-1.5628	-0.62479
C	4.70405	1.56292	-0.62438
C	5.22746	2.81558	-0.85264

C	6.6402	2.89021	-0.79823
C	7.22242	1.66175	-0.52248
S	5.99116	0.42437	-0.30227
C	-5.22746	-2.81545	-0.85314
C	-6.6402	-2.89012	-0.79883
C	-7.22247	-1.66169	-0.52308
S	-5.99126	-0.42429	-0.30272
C	8.60903	1.28383	-0.41007
C	-8.60909	-1.28379	-0.41073
C	9.13827	0.00973	-0.47289
C	10.5359	-0.072	-0.28981
C	11.11658	1.17346	-0.08315
S	9.8909	2.43501	-0.09631
C	-9.13837	-0.00971	-0.47362
C	-10.536	0.072	-0.29056
C	-11.11665	-1.17347	-0.08384
S	-9.89094	-2.43499	-0.0969
C	12.49292	1.55052	0.10158
C	-12.49298	-1.55055	0.10089
S	13.7564	0.39162	0.4743
C	14.98122	1.64774	0.55002
C	14.40362	2.8847	0.30301
C	13.02708	2.83303	0.05337
S	-13.7564	-0.39172	0.47405
C	-14.98124	-1.64784	0.54953
C	-14.4037	-2.88474	0.30213
C	-13.02717	-2.83303	0.05238
C	16.36367	1.43294	0.80925
C	17.05927	0.29341	1.04976
C	-16.36367	-1.43308	0.80893
C	-17.0592	-0.29361	1.04987
C	-7.3832	-4.17424	-1.04622
C	-11.27148	1.38195	-0.35312
C	7.38324	4.17432	-1.0455
C	11.27134	-1.38198	-0.35229
C	18.51435	0.34613	1.28747
N	19.01136	-0.95676	1.50941
C	18.1169	-1.99049	1.4712
S	16.48398	-1.3737	1.13239
C	-18.51427	-0.34636	1.2877
N	-19.0112	0.95647	1.51017
C	-18.11671	1.99018	1.4722
S	-16.48385	1.37345	1.13301
S	18.4386	-3.59123	1.69612
C	20.43267	-1.17971	1.76814
O	19.21228	1.3376	1.29764

S	-18.43834	3.59086	1.69767
C	-20.43248	1.17939	1.76911
O	-19.21223	-1.3378	1.29758
H	3.44646	-0.99678	-0.5997
H	-3.44657	0.99693	-0.60023
H	0.20103	-3.60406	1.06057
H	-0.201	3.60462	1.05988
H	3.39176	-6.69049	-1.17155
H	4.11116	-5.47896	-2.23911
H	2.64438	-6.33385	-2.73361
H	1.40141	-5.95477	1.79279
H	2.12319	-6.93385	0.50764
H	0.3797	-6.65087	0.53178
H	-3.39163	6.69071	-1.17287
H	-4.11128	5.47889	-2.23992
H	-2.64451	6.3335	-2.73493
H	-1.40215	5.9556	1.79165
H	-2.12253	6.93468	0.50571
H	-0.37921	6.65077	0.53113
H	4.6074	3.67345	-1.08145
H	-4.60736	-3.6733	-1.08195
H	8.52879	-0.86292	-0.67058
H	-8.5289	0.86294	-0.67136
H	14.98889	3.79551	0.29707
H	12.42513	3.70346	-0.17145
H	-14.98898	-3.79554	0.29598
H	-12.42525	-3.70341	-0.17274
H	16.97338	2.33379	0.81638
H	-16.9734	-2.33391	0.81582
H	-6.7009	-4.9319	-1.43811
H	-8.19505	-4.03672	-1.76675
H	-7.82599	-4.56655	-0.12389
H	-10.59009	2.17968	-0.6563
H	-11.69398	1.65275	0.62059
H	-12.09786	1.34649	-1.06992
H	6.70097	4.93204	-1.43732
H	8.19508	4.03684	-1.76606
H	7.82607	4.56653	-0.12315
H	10.58987	-2.17975	-0.65521
H	11.69402	-1.65263	0.62139
H	12.09758	-1.34666	-1.06925
H	20.922	-0.2072	1.74359
H	20.84349	-1.84127	1.00308
H	20.55668	-1.65288	2.74405
H	-20.92178	0.20686	1.74467
H	-20.84345	1.84089	1.00408

H	-20.55635	1.6526	2.74502

Section S11.5 BQR

C	-1.36497	0.40479	0.46226
C	-0.97582	-0.96477	0.46395
C	0.35335	-1.38197	0.38492
C	1.32953	-0.36472	0.28695
C	0.94081	1.00493	0.29061
C	-0.38692	1.42241	0.38738
C	-2.78229	0.55811	0.55335
C	-3.46563	-0.62198	0.64084
S	-2.37958	-2.01923	0.5963
C	2.74423	-0.51853	0.16369
C	3.42989	0.66219	0.10467
S	2.34706	2.05996	0.19589
C	-0.73436	2.84912	0.40799
C	0.70379	-2.80763	0.40416
C	-0.37945	3.81235	-0.49552
C	-0.84272	5.12597	-0.17434
C	-1.55593	5.1568	0.99586
S	-1.65681	3.56682	1.71309
C	0.21617	-3.80147	-0.39886
C	0.72514	-5.10441	-0.10485
C	1.60921	-5.09577	0.94271
S	1.81678	-3.48113	1.57762
C	-2.178	6.33325	1.68775
C	-0.54731	6.31953	-1.04164
C	2.3314	-6.24938	1.57333
C	0.30236	-6.32661	-0.87373
C	4.85132	0.85775	-0.02969
C	-4.88957	-0.81853	0.74637
C	-5.57522	-1.96246	1.08614
C	-6.98454	-1.82447	1.12062
C	-7.39585	-0.53987	0.80031
S	-6.01412	0.48131	0.4239
C	5.53589	2.01652	-0.31713
C	6.94246	1.87322	-0.40038
C	7.35418	0.56877	-0.17251
S	5.97488	-0.46619	0.17789
C	-8.71636	0.03855	0.74772
C	8.67089	-0.01966	-0.19471
C	-9.04692	1.3776	0.7954
C	-10.42718	1.66115	0.68161
C	-11.1929	0.5123	0.54756
S	-10.16964	-0.91611	0.53319

C	8.98677	-1.36063	-0.27939
C	10.36772	-1.65864	-0.24023
C	11.15172	-0.52013	-0.12172
S	10.14517	0.92029	-0.06892
C	-12.62112	0.34017	0.4553
C	12.58259	-0.36622	-0.04396
S	-13.69955	1.60925	-0.08531
C	-15.11223	0.57058	0.06563
C	-14.73902	-0.69827	0.49249
C	-13.34743	-0.80726	0.70641
S	13.70655	-1.60681	-0.55986
C	15.10047	-0.60475	-0.17417
C	14.6913	0.63284	0.30759
C	13.28564	0.74604	0.37639
C	-16.40846	1.12088	-0.22845
C	16.4172	-1.15064	-0.36819
C	-16.72595	2.45757	-0.44277
C	-18.07577	2.6807	-0.73824
C	-18.84886	1.52888	-0.76071
S	-17.84625	0.13077	-0.40801
C	16.7478	-2.47494	-0.63324
C	18.11926	-2.69426	-0.80685
C	18.89632	-1.5519	-0.68012
S	17.87024	-0.1672	-0.34194
C	-20.24731	1.49374	-1.01893
C	20.31354	-1.51674	-0.79684
C	21.19382	-0.49062	-0.68362
C	-21.12171	0.4572	-1.05949
C	7.84605	3.02981	-0.72931
C	10.88157	-3.07004	-0.32016
C	15.6009	1.74689	0.74623
C	-7.89189	-2.96325	1.49845
C	-10.96351	3.06505	0.74465
C	-15.67398	-1.84874	0.7436
S	20.88957	1.21404	-0.33882
C	22.62178	1.60757	-0.41708
N	23.34754	0.4817	-0.69199
C	22.63915	-0.72902	-0.85531
S	-20.83043	-1.26493	-0.80113
C	-22.54197	-1.66302	-1.07258
N	-23.25244	-0.52555	-1.33954
C	-22.54688	0.69771	-1.3536
C	-24.68984	-0.56102	-1.60277
S	-23.12424	-3.20329	-0.9984
O	-23.07094	1.76794	-1.57817
C	24.80377	0.51639	-0.81365

S	23.20352	3.13175	-0.18161
O	23.17592	-1.78849	-1.1001
H	-3.2718	1.52152	0.56525
H	3.22866	-1.48266	0.10077
H	0.18779	3.58556	-1.38971
H	-0.48157	-3.6044	-1.20325
H	-2.34139	7.14701	0.97725
H	-1.53504	6.7145	2.48977
H	-3.14414	6.07478	2.13141
H	-0.66206	6.06522	-2.09989
H	0.48254	6.66648	-0.89645
H	-1.2127	7.15702	-0.82091
H	2.40477	-7.08102	0.86845
H	1.80761	-6.61259	2.46536
H	3.34638	-5.97304	1.87389
H	0.29024	-6.12147	-1.94882
H	-0.70845	-6.64044	-0.5879
H	0.97315	-7.17013	-0.69726
H	-5.07386	-2.88909	1.33702
H	5.03561	2.96107	-0.49138
H	-8.3045	2.1529	0.9374
H	8.23115	-2.12995	-0.37718
H	-12.88635	-1.71915	1.06401
H	12.79546	1.63524	0.75219
H	-15.98907	3.24669	-0.37349
H	-18.50371	3.65733	-0.926
H	16.00364	-3.25845	-0.68642
H	18.55911	-3.66213	-1.01167
H	-20.69985	2.46243	-1.21939
H	20.77811	-2.47576	-1.01547
H	7.2521	3.8938	-1.03587
H	8.5372	2.78268	-1.54094
H	8.44934	3.32752	0.1356
H	10.0548	-3.77594	-0.21373
H	11.61322	-3.2762	0.46686
H	11.36942	-3.26681	-1.2814
H	16.33447	1.40323	1.48231
H	15.01643	2.55181	1.19691
H	16.15752	2.1675	-0.09832
H	-7.30761	-3.78194	1.92488
H	-8.43076	-3.35428	0.62814
H	-8.63945	-2.65381	2.23476
H	-10.17926	3.75152	1.07167
H	-11.80313	3.14333	1.4417
H	-11.31829	3.40356	-0.23517
H	-16.48157	-1.57021	1.42797

H	-15.12773	-2.68597	1.18324
H	-16.13829	-2.20005	-0.18428
H	-25.20866	-0.98531	-0.74106
H	-24.88636	-1.18847	-2.47422
H	-25.01085	0.46392	-1.78254
H	25.24109	0.86474	0.12406
H	25.08717	1.20657	-1.61048
H	25.13308	-0.49621	-1.04181

Section S11.6 BPR

C	-1.39039	0.19931	-1.81744
C	-0.81968	-1.10491	-1.82057
C	0.55592	-1.33834	-1.83046
C	0.81937	1.10674	-1.82021
C	-0.55623	1.34018	-1.83065
C	-2.81823	0.15842	-1.80454
C	-1.09344	2.70674	-1.85075
C	1.09313	-2.70491	-1.85038
C	-0.80785	3.73238	-0.99277
C	-1.46502	4.96396	-1.30111
C	0.80716	-3.73056	-0.99253
C	1.46447	-4.96212	-1.30061
C	4.72718	1.49666	-1.7986
C	-4.72749	-1.49485	-1.80064
C	-6.68179	-2.8024	-1.92166
C	-7.24633	-1.55924	-1.68598
C	5.26734	2.74953	-1.98056
C	6.68149	2.80439	-1.91775
C	-8.62978	-1.16229	-1.57767
C	8.62947	1.16387	-1.57575
C	-11.12224	-1.00796	-1.17877
C	-10.5548	0.20018	-1.55318
C	1.39008	-0.19747	-1.8169
C	-3.34015	-1.10459	-1.81556
S	-2.07284	-2.34144	-1.82907
C	2.81791	-0.15658	-1.80338
C	3.33984	1.10642	-1.81415
S	2.07254	2.34327	-1.82822
C	-2.25683	4.86883	-2.41581
S	-2.19459	3.26035	-3.09616
C	2.25676	-4.86698	-2.41496
S	2.19482	-3.2585	-3.09531

C	-3.07893	5.93284	-3.08059
C	-1.27146	6.207	-0.47562
C	3.07906	-5.93108	-3.07934
C	16.27362	1.01434	0.10801
C	-5.26764	-2.74748	-1.98429
S	-6.00079	-0.33028	-1.51249
C	7.24601	1.56093	-1.68368
S	6.00049	0.33171	-1.51197
S	-9.89697	-2.26246	-1.07477
C	-9.16117	0.09519	-1.7739
S	9.89652	2.26335	-1.07104
C	11.12187	1.00908	-1.17672
C	10.55454	-0.19854	-1.55298
C	9.16094	-0.0933	-1.77374
C	-12.49492	-1.3589	-0.90561
C	12.49449	1.35971	-0.90292
S	-13.69277	-0.18792	-0.393
C	-14.93952	-1.41916	-0.26005
C	-14.42718	-2.66972	-0.57046
C	-13.06115	-2.61723	-0.93171
C	13.06071	2.61807	-0.92742
C	14.42666	2.67016	-0.56585
C	14.939	1.41923	-0.25683
S	13.69225	0.18814	-0.39149
C	11.30315	-1.48753	-1.75606
C	7.44475	4.08526	-2.11835
C	-11.30331	1.4895	-1.75448
C	-15.20884	-3.95497	-0.57644
C	23.55807	0.92973	3.03984
C	18.90611	-1.66167	0.42401
C	15.20822	3.95547	-0.57011
C	18.15047	-0.36323	0.48645
C	18.64462	0.83906	0.97848
S	17.4266	2.10331	0.84906
C	19.93258	1.1747	1.52414
S	21.13069	-0.03308	1.95556
C	22.25955	1.1882	2.52013
C	21.70118	2.44975	2.37262
C	20.41518	2.44478	1.82054
C	1.27056	-6.20527	-0.47535
C	-16.27424	-1.01471	0.105
C	-16.82931	0.24521	0.00102
C	-18.15155	0.36213	0.48364
C	-18.64499	-0.84023	0.97616
S	-17.4264	-2.10394	0.84685
C	-19.93269	-1.17633	1.52219

C	16.82814	-0.24586	0.00422
C	-18.90787	1.66017	0.42096
S	-21.13029	0.03117	1.95577
C	-22.25894	-1.19055	2.51979
C	-21.70086	-2.45202	2.37055
C	-20.41525	-2.44664	1.81756
C	-24.23072	0.23111	3.2229
C	-23.55711	-0.93247	3.04059
C	24.23184	-0.23397	3.22073
S	23.72596	-1.88801	2.8656
C	25.26947	-2.55478	3.44323
N	26.08621	-1.54557	3.87243
C	25.59381	-0.2247	3.78633
S	25.60521	-4.16866	3.43519
C	27.42255	-1.81086	4.40194
O	26.22825	0.74801	4.13547
S	-23.72503	1.88542	2.86878
C	-25.26815	2.55175	3.44794
N	-26.08464	1.54221	3.87685
C	-25.59232	0.22141	3.78939
S	-25.60386	4.16564	3.44141
O	-26.22655	-0.75157	4.13818
C	-27.42065	1.8071	4.40743
H	-3.43227	1.04776	-1.7928
H	-0.15325	3.60674	-0.13928
H	0.15222	-3.60493	-0.13931
C	-7.44505	-4.08299	-2.124
H	4.65918	3.62311	-2.18064
H	3.43192	-1.04594	-1.79132
H	-3.29967	6.73676	-2.37457
H	-2.55172	6.37351	-3.93491
H	-4.03066	5.53616	-3.44661
H	-1.32606	5.97341	0.59238
H	-0.28836	6.65416	-0.6639
H	-2.02729	6.9639	-0.69549
H	3.30014	-6.73456	-2.37293
H	2.55183	-6.37237	-3.93333
H	4.03061	-5.53432	-3.44575
H	-4.65946	-3.62077	-2.18555
H	-8.55885	0.93334	-2.10147
H	8.5587	-0.93099	-2.10264
H	-12.50553	-3.49625	-1.23348
H	12.50512	3.49744	-1.22823
H	10.67412	-2.20721	-2.28513
H	12.21621	-1.33363	-2.33855
H	11.59556	-1.93709	-0.80051

H	6.79039	4.84792	-2.54717
H	7.83529	4.47344	-1.17092
H	8.29624	3.94354	-2.78997
H	-10.67406	2.21002	-2.28214
H	-12.21613	1.33654	-2.33759
H	-11.59611	1.93753	-0.79834
H	-14.64402	-4.73512	-1.09199
H	-15.41192	-4.30579	0.44156
H	-16.17255	-3.83641	-1.08022
H	24.11245	1.81413	3.34622
H	18.31707	-2.41236	-0.10714
H	19.86266	-1.54754	-0.09591
H	19.12241	-2.05011	1.42519
H	14.64281	4.73659	-1.08354
H	15.41235	4.3043	0.44837
H	16.17142	3.838	-1.07515
H	22.23682	3.34631	2.65806
H	19.84217	3.34216	1.6286
H	1.32214	-5.97137	0.59272
H	0.28859	-6.65391	-0.66602
H	2.02804	-6.96113	-0.69317
H	-16.29719	1.07848	-0.44006
H	16.29553	-1.07907	-0.43637
H	-18.31979	2.4107	-0.11145
H	-19.86496	1.54515	-0.09776
H	-19.12322	2.04934	1.42207
H	-22.23645	-3.34881	2.65535
H	-19.84251	-3.34389	1.62415
H	-24.11132	-1.81711	3.3466
H	28.02877	-2.30015	3.63704
H	27.85476	-0.85179	4.68347
H	27.34739	-2.47308	5.26644
H	-28.02736	2.29695	3.64328
H	-27.85266	0.84782	4.68852
H	-27.34492	2.46868	5.27236
H	-6.7906	-4.84518	-2.55354
H	-8.29634	-3.94045	-2.79572
H	-7.8359	-4.47228	-1.17717

Section S11.7 TD-DFT Energy Level Calculation

The HOMO/LUMO energy levels were calculated using TD-DFT and benchmarked against experimentally determined orbital energy levels. Different functionals with varying Hartree-Fock contribution were investigated to determine the optimal functional (CAM-B3LYP, HF = 19%; PBE0, HF = 25%; BMK, HF = 42%; BH and HLYP, HF = 50%; M06-2X, HF = 54%), as shown in Table S1.

Table S1. Calculated HOMO/LUMO energy levels with different functionals

Orbital (eV)	BMR					
	Experimental (CV Film)	CAM-B3LYP	PBE0	BMK	BH and HLYP	M06-2X
LUMO	-3.42	-2.26	-3.14	-2.70	-2.36	-2.54
HOMO	-5.68	-6.25	-5.67	-6.10	-6.25	-6.53

The investigation was focused around the BMR material. This was due to the relative simplicity of the material in comparison to the larger derivatives that would be more expensive to calculate. The PBE0 functional shows the lowest mean absolute error compared to the experimental results concerning the HOMO energy level but the LUMO energy level is different by ~0.3 eV but still shares the highest agreement with experimental values. It is apparent that as the BXR molecular materials increase in size the overlap of the HOMO and LUMO decreases. The HOMO overlap does not shift significantly as the series extends from BMR to BPR with it delocalized out to the third and partially fourth thiophene along the bridging arm. However, the LUMO is isolated more on the N-hexyl-rhodanine acceptor moiety as the conjugation length increases. The energy levels of the HOMO calculated match closely to the experimental data determined from CV of the films with BQR showing a small exception. The LUMO levels are all consistently deviated from the experimental values but the trend in the energy levels holds true along with the energy gap.

[1] M. J. Frisch, G. W. Trucks, H. B. Schlegel, G. E. Scuseria, M. A. Robb, J. R. Cheeseman, G. Scalmani, V. Barone, B. Mennucci, G. A. Petersson, H. Nakatsuji, M. Caricato, X. Li, H. P. Hratchian, A. F. Izmaylov, J. Bloino, G. Zheng, J. L. Sonnenberg, M. Hada, M. Ehara, K. Toyota, R. Fukuda, J. Hasegawa, M. Ishida, T. Nakajima, Y. Honda, O. Kitao, H. Nakai, T. Vreven, J. A. Montgomery Jr., J. E. Peralta, F. Ogliaro, M. J. Bearpark, J. Heyd, E. N. Brothers, K. N. Kudin, V. N. Staroverov, R. Kobayashi, J. Normand, K. Raghavachari, A. P. Rendell, J. C. Burant, S. S. Iyengar, J. Tomasi, M. Cossi, N. Rega, N. J. Millam, M. Klene, J. E. Knox, J. B. Cross, V. Bakken, C. Adamo, J. Jaramillo, R. Gomperts, R. E. Stratmann, O. Yazyev, A. J. Austin, R. Cammi, C. Pomelli, J. W. Ochterski, R. L. Martin, K. Morokuma, V. G. Zakrzewski, G. A. Voth, P. Salvador, J. J. Dannenberg, S. Dapprich, A. D. Daniels, Ö. Farkas, J. B. Foresman, J. V. Ortiz, J. Cioslowski, D. J. Fox, Gaussian, Inc., Wallingford, CT, USA, **2010**.
